# Transcriptome and Metabolite Insights into Domestication Process of Cultivated Barley in China

**DOI:** 10.3390/plants11020209

**Published:** 2022-01-14

**Authors:** Yu Zhou, Guang Lu, Genlou Sun, Daokun Sun, Xifeng Ren

**Affiliations:** 1 College of Plant Science and Technology, Huazhong Agricultural University, Wuhan 430070, China; zhouyu@mail.hzau.edu.cn (Y.Z.); ab623413443@163.com (G.L.); ab173836094@163.com (D.S.); 2 Hubei Hongshan Laboratory, Wuhan 430070, China; 3 Saint Mary’s University, Halifax, NS B3H 3C3, Canada; genlou.sun@smu.ca

**Keywords:** domestication process, barley, feralization, alkaloid, phenylpropanoid

## Abstract

The domestication process of cultivated barley in China remains under debate because of the controversial origins of barley. Here, we analyzed transcriptomic and non-targeted metabolic data from 29 accessions together with public resequencing data from 124 accessions to explore the domestication process of cultivated barley in China (Cb-C). These analyses revealed that both Cb-C and Tibetan wild barley (Wb-T) were the descendants of wild barley from the Near East Fertile Crescent (Wb-NE), yielding little support for a local origin of Wb-T. Wb-T was more likely an intermediate in the domestication process from Wb-NE to Cb-C. Wb-T contributed more genetically to Cb-C than Wb-NE, and was domesticated into Cb-C about 3300 years ago. These results together seem to support that Wb-T may be a feralized or hybrid form of cultivated barley from the Near East Fertile Crescent or central Asia. Additionally, the metabolite analysis revealed divergent metabolites of alkaloids and phenylpropanoids and these metabolites were specifically targeted for selection in the evolutionary stages from Wb-NE to Wb-T and from Wb-T to Cb-C. The key missense SNPs in the genes *HORVU6Hr1G027650* and *HORVU4Hr1G072150* might be responsible for the divergence of metabolites of alkaloids and phenylpropanoids during domestication. Our findings allow for a better understanding of the domestication process of cultivated barley in China.

## 1. Introduction

Barley is one of the earliest domesticated crops and is regarded as one of the founders of the Neolithic transition in the Near East Fertile Crescent [1]. Unlike wheat and other founder crops, the widely eastward dispersal of wild barley (*Hordeum vulgare* ssp. *spontaneum*), the progenitor of cultivated barley (*H. vulgare* ssp. *vulgare*), extends from the Near East Fertile Crescent into central Asia and even the Tibetan Plateau [1,2,3,4,5]. Since the discovery of Tibetan wild barley (*H. vulgare* ssp. *spontaneum* and *H. agriocrithon* Åberg), an increasing number of studies proposed that Tibet was one of the centers of barley domestication [6,7,8,9,10]. Nevertheless, some results rejected or questioned this hypothesis that Tibet was a center of origin or domestication for barley [11,12], and favored that barley has a monophyletic origin in the Fertile Crescent, against multi-geographic origins for this crop [13,14,15].

Tibetan wild barley does not exist as a wild population in the Qinghai Tibet Plateau but occurs as a weed at the edges of fields in the region [9,10,11]. Meanwhile, a large number of studies suggested that *H. agriocrithon* has a highly heterogeneous genetic structure, and originated from six-rowed barley landraces [11,12,15,16,17,18]. Thus, the existence of Tibetan wild barley only provided weak evidence for the hypothesis that Tibet was one of the centers of barley origin or domestication. Tibetan cultivated barley (qingke) was derived from the Fertile Crescent, and was first introduced to southern Tibet most likely via north Pakistan, India, and Nepal between 3500 and 4500 years ago [11].

During domestication, the selection process drives the improvement of adaptation for plants and animals to be more suitable for cultivation or rearing [19]. The untangling domestication process of barley is fundamental to understanding the origins and early diffusion of agrarian culture [20]. So far, the undisputed research result about cultivated barley in east Asia is that cultivars and landraces of barley in east Asia have significantly diverged in genetic compositions from those in West [11,13,20,21]. However, the evidence is far from completion in depicting the domestication landscape of cultivated barley in east Asia.

Compared with wild barley, cultivated barley underwent dramatic morphological transformations during domestication, such as in the rachis, seed dormancy, seed size (Table S1) and number, and flowering time [22,23]. Additionally, there is growing evidence showing that morphological changes were frequently accompanied by coordinated metabolite alterations during crop domestication [24,25,26,27,28]. However, there is no investigation on the alterations in metabolism from barley domestication so far.

The objectives of this study were to untangle the controversial domestication process of cultivated barley in China and to explore metabolic changes associated with barley domestication. Specifically, we analyzed transcriptomic and metabolic data from 29 accessions (nine wild barley from the Near East Fertile Crescent (Wb-NE), ten wild barley from the Tibetan Plateau (Wb-T), and ten cultivated barley from China (Cb-C)) together with published resequencing data of 124 accessions from Zeng et al. [11]. We showed that both Cb-C and Wb-T were the descendants of Wb-NE. Wb-T played an important intermediate role in the domestication process of Cb-C, and contributed more genetically to Cb-C than Wb-NE. Additionally, an evolutionary metabolomics method was employed to assess metabolite divergence in seedlings between Wb-NE and Wb-T, and between Wb-T and Cb-C. We revealed that distinct sets of metabolites were selective during the evolution from Wb-NE to Wb-T to Cb-C. Our findings provide genetic and metabolic insights into domestication process of barley.

## 2. Results

### 2.1. Both Cb-C and Wb-T Are the Descendants of Wb-NE

RNA sequencing of 29 barley accessions (Table S2, Figure S1) generated a total of 185.60 gigabase (Gb) of high-quality cleaned sequences, with an average of 6.19 Gb per accession (Table S2). Bioinformatics analysis detected 1,182,394 raw SNPs and 176,741 high-quality SNPs after stringent filtering for subsequent analyses.

The genetic diversity analysis for the three groups of barley accessions revealed that Wb-NE had the highest nucleotide diversity (π = 0.23036), highest Watterson’s estimator (θ_W_ = 0.21255), highest minor allele frequency (MAF = 0.1745), and greatest polymorphism information content (PIC = 0.2176) (Table 1). This was followed by the Wb-T group with π = 0.18906, θ_W_ = 0.15944, MAF = 0.1453, and PIC = 0.1796, while Cb-C had the lowest in those indexes (Table 1).

**Figure 1:**
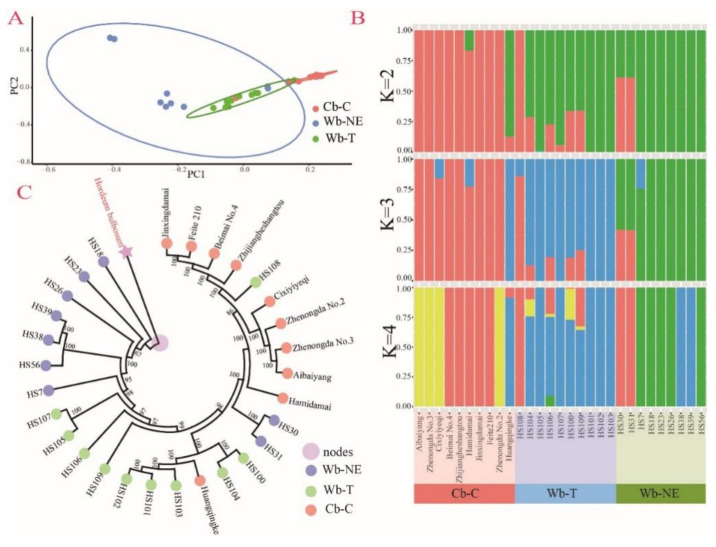
PCA scatter plot, population structure and rooted phylogenetic tree of 29 barley accessions representing three typical groups. Wb-NE, wild barley from the Near East Fertile Crescent. Wb-T, wild barley from the Tibetan Plateau. Cb-C, cultivated barley from China. (**A**) PCA scatter plot was visualized based on the PC1 (the first principal component) and PC2 (the second principal component). (**B**) Population structure of three barley groups when K was 2, 3, and 4, respectively. (**C**) Rooted phylogenetic tree of 29 barley accessions with *H. bulbosum* as an outgroup.

The PCA scatter plot, population structure (K = 2~4), and rooted phylogenetic tree are shown in Figure 1. The PCA scatter plot revealed that cultivated accessions were clustered together and obviously separated from wild accessions (Figure 1A). An optimal population division (cv error = 0.82356) appeared when the K was 2 (Table S3), indicating that all accessions were separated into wild accessions and cultivated accessions (Figure 1B). The results were consistent with the results of PCA. When K was 3, Wb-T was separated from Wb-NE (Figure 1B). To assess the evolutionary relationships of the three groups, we constructed rooted phylogenetic trees based on 165,848 high-quality SNPs using the bulbous barley (*H. bulbosum*) as an outgroup that was differentiated from barley about 5.80 MYA (million years ago) (http://www.timetree.org/, accessed on 18 October 2021). Wb-NE was found to be located at the basal position of the tree (Figure 1C), and differentiated from the bulbous barley. Both Wb-T and Cb-C were derived from Wb-NE (Figure 1C), supporting that the Wb-NE group was the oldest clade. Similar results were also revealed by the rooted tree constructed using Tajima–Nei model (Figure S2).

### 2.2. Wb-T Contributed More to Cb-C Than Wb-NE

**Figure 2:**
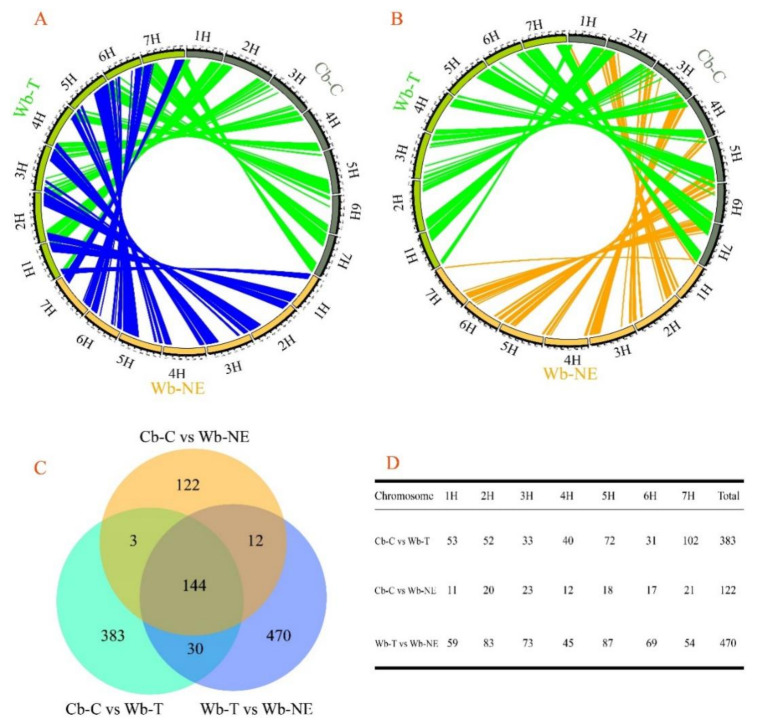
Genomic similarity between three groups of barley accessions. Wb-NE, wild barley from the Near East Fertile Crescent. Wb-T, wild barley from the Tibetan Plateau. Cb-C, cultivated barley from China. (**A**) Circos diagram with lines shows genomic similarity regions between Wb-T and Wb-NE (blue lines) and between Wb-T and Cb-C (green lines). The similar regions were connected with lines and each line represented one unique window (150 kb) of the genome with the highest similarity between two populations. (**B**) Circos diagram with lines showing genomic similarity regions between Cb-C and Wb-NE (orange lines) and between Wb-T and Cb-C (green line). The similar regions are connected with lines and each line represents one unique window (150 kb) of the genome with the highest similarity between two populations. The green line was consistent with the green line of sub-figure (**A**). (**C**) Venn diagram exhibiting the numbers of genomic similarity regions among the three groups. (**D**) The numbers of unique genomic similarity regions on seven chromosomes.

ABBA-BABA test and Treemix analysis detected the presence of the gene flow occurred between Cb-C and Wb-NE and between Cb-C and Wb-T (Figure S3). Based on the results of population structure inference and rooted phylogenetic tree (Figure 1B,C), four accessions (Huangqingke, HS30, HS31 and HS108) were removed to minimize the effect of admixture between groups, and further genomic similarity analyses was made of 25 accessions (nine accessions in Cb-C, nine accessions in Wb-T and seven accessions in Wb-NE). The 150-kb windows and 75-kb overlapping slide windows were used for the better coverage along the reference genome of barley. These analyses detected a total of 1155 windows with high similarity, which covered roughly 3.58% of barley reference genome. The 1155 windows were illustrated in Circos diagrams (Figure 2A,B). Compared with the numbers of windows between Wb-NE and Cb-C (281), more windows were detected between Wb-T and Cb-C (560) (Figure 2A,B), indicating that Wb-T shared more genomic regions to Cb-C than Wb-NE.

There were only 122 unique genomic regions between Wb-NE and Cb-C (Figure 2C). In contrast, 383 and 470 unique genomic regions were detected between Wb-T and Cb-C, and between Wb-T and Wb-NE, respectively (Figure 2C). Meanwhile, unique genomic regions on each chromosome were summarized in Figure 2D. Compared with the numbers of unique windows between Cb-C and Wb-NE, more unique windows between Cb-C and Wb-T were detected across the seven chromosomes, particularly on 5H and 7H (Figure 2D).

### 2.3. Wb-T Was the Product of Feralization or Hybridization of Cultivated Barley

**Figure 3:**
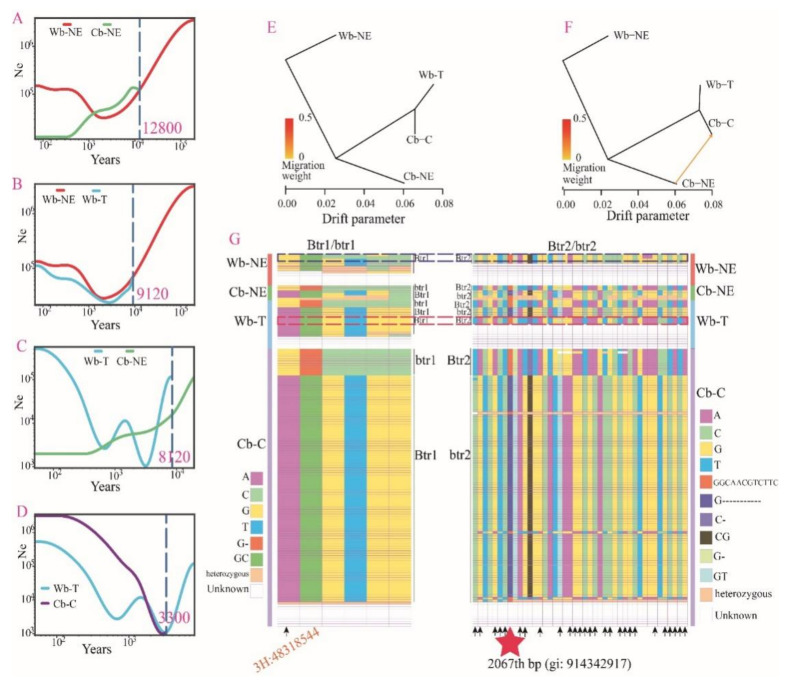
Origin of Wb-T and its genetic relationship with Cb-C. Wb-NE, wild barley from the Near East Fertile Crescent. Wb-T, wild barley from the Tibetan Plateau. Cb-C, cultivated barley from China. Cb-NE, cultivated barley from the Near East Fertile Crescent. (**A**–**D**) Effective population size (Ne) changed for the four groups and estimated divergence time between Wb-NE and Cb-NE (**A**), between Wb-NE and Wb-T (**B**), between Cb-NE and Wb-T (**C**), and between Wb-T and Cb-C (**D**) were inferred by SMC++. Estimated divergence time is denoted by the blue dotted line and was assumed one generation per year. The mutation rate was 6.5 × 10^−9^ per site per generation. (**E**,**F**) Inference of population splits with 0 mixture events (**E**) and 1 mixture event (**F**) by TreeMix. Admixture events are indicated by arrows and the arrow color denotes the migration weight. (**G**) Haplotype heatmaps at *Btr1*/*btr1* and *Btr2*/*btr2* loci in 153 accessions (124 resequencing + 29 RNA sequencing). Key variations are denoted by red font in the *Btr1*/*btr1* heatmap and the star in the *Btr2*/*btr2* heatmap. Accessions in the *Btr1*/*btr1* heatmap were corresponded with those in the *Btr2*/*btr2* heatmap. Arrows below the accessions indicate that the accessions show differences in SNP or InDel sites in the Wb-NE accessions (blue dashed box) and the Wb-T accessions (red dashed box), though both of them exhibit the *Btr1Btr2* genotype.

Wild barley from the Tibetan Plateau and surrounding areas is unique and makes the domestication process of Cb-C complicated and confused. In order to gain more insights into the domestication process of Cb-C, we downloaded 124 barley accessions resequencing data from Zeng et al. [11] together with our 29 RNA sequencing data for further study. These 153 barley accessions were assigned into four groups: Wb-NE (thirteen accessions), Cb-NE (cultivated barley from the Near East Fertile Crescent, six accessions), Wb-T (twenty accessions), and Cb-C (one hundred and fourteen accessions). A demographic analysis showed that the effective population size (Ne) of Wb-NE has declined until ~2100 years ago, suggesting that a genetic bottleneck lasted for a long time (Figure 3A). Since ~12,800 years ago, Cb-NE was differentiated from Wb-NE in the Near East Fertile Crescent (Figure 3A). Wb-T was differentiated from Wb-NE ~9120 years ago (Figure 3B), and from Cb-NE ~8120 years ago (Figure 3C). This suggested that Wb-T was likely to be the product of feralization or hybridization of Cb-NE. Cb-C was differentiated from Wb-T ~3300 years ago (Figure 3D), suggesting that Cb-C is a descendant of Wb-T. After differentiation, they had similar patterns in the Ne that fluctuated (Figure 3D).

The TreeMix software enables to calculate chronological population splits and infer population mixture events, and was used to examine the relationships among the four groups. As a result, the topology of the maximum likelihood tree was robust when a 0 or 1 migration event was allowed in the model (Figure 3E,F and Figure S4A,B). Wb-T and Cb-C consistently formed a clade and both were differentiated from Cb-NE. Additionally, both the evolutionary branches of Wb-T and Cb-C were relatively short, suggesting that they appeared relatively late (Figure 3E,F). In the maximum likelihood tree, one migration event was observed from Cb-NE to Cb-C (Figure 3F). This suggests that gene flow had taken place between eastern and western barley accessions during the domestication process of barley.

These 153 barley accessions were also used to search for molecular variation in Btr1/btr1 and Btr2/btr2 loci. The btr1 and btr2 were different from Btr1 and Btr2 by a 1-bp (GC/G-) and an 11-bp deletion (GGCAACGTCTTC/G-----------), respectively (Figure 3G). All Wb-NE accessions with known SNPs or InDels showed the Btr1Btr2 genotype (Figure 3G). Two Cb-NE barley accessions showed the btr1Btr2 genotype, while the remaining four showed the Btr1btr2 genotype (Figure 3G). Three Wb-T barley accessions had the btr1Btr2 genotype, three had the Btr1btr2 genotype, and three were discovered with Btr1Btr2 and exhibited brittle rachis (Figure 3G). Although the three Wb-T accessions had the Btr1Btr2 genotype, 27 SNPs (1 in the Btr1/btr1 locus and 26 in the Btr2/btr2 locus) and 1 InDels (in the Btr2/btr2 locus) are inconsistent with Wb-NE, but consistent with Cb-NE and Cb-C. These results indicate that the Btr1Btr2 of Wb-T was a new haplotype that was different from Wb-NE, and could be the result of recombination by hybridization of cultivated barley accessions with the Btr1btr2 and btr1Btr2 genotypes.

PCA and population structure inference of the four barley groups revealed that 14 Wb-T accessions showed closer genetic relationships with Cb-C (Figure S5). However, the remaining Wb-T accessions were clustered into Cb-NE or Wb-NE (Figure S5). Additionally, Wb-T seemed to present earlier than Cb-C (Figure 3D), and the genetic diversity of Wb-T was higher than that of Cb-C, even though Wb-T contained only 20 accessions (Figure S5). These results do not suggest that Wb-T was the descendant of Cb-C, although it may have been the product of feralization or hybridization of cultivated barley (Figure 3G). When K was 3 or 4, nearly half of the accessions of Wb-T showed a close genetic relationship with Cb-NE and Wb-NE (Figure S6). The result was consistent with the result of PCA (Figure S5), questioning the local origin of Wb-T. It is more likely that Wb-T was a feralized or hybrid form of cultivated barley from the Near East Fertile Crescent or Central Asia.

### 2.4. Different Evolutionary Stages Were Accompanied by Different Sets of Divergent Metabolites

**Figure 4:**
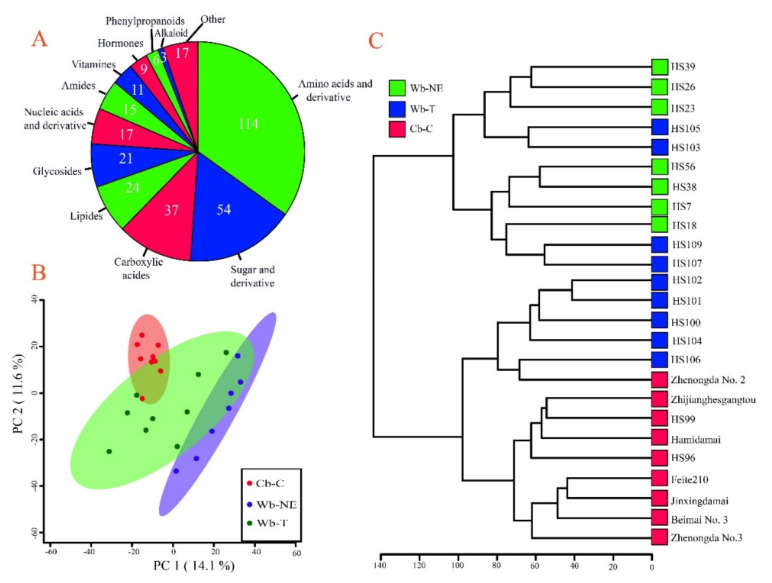
Metabolome divergence between Cb-C and its wild relatives. Wb-NE, wild barley from the Near East Fertile Crescent. Wb-T, wild barley from the Tibetan Plateau. Cb-C, cultivated barley from China. (**A**) Classification of metabolites with annotated structures. (**B**) PCA of the Cb-C, Wb-T, and Wb-NE accessions with all metabolites. PC1, the first principal component. PC2, the second principal component. (**C**) Hierarchical cluster of Cb-C, Wb-T, and Wb-NE accessions. The clustering tree was drawn based on Euclidean distance.

To determine the difference in the metabolomes among the three groups of barley accessions, the seedling metabolomes of three pure groups including 25 accessions described in genomic similarity analysis were quantified using a non-targeted metabolomics approach. In total, 8828 non-redundant metabolites were detected (Table S4), including 328 structure-annotated metabolites (Table S5). These annotated metabolites covered 12 metabolite classes, including 114 amino acids and derivatives, 54 sugar and derivatives, 37 carboxylic acids, 24 lipides, 21 glycosides, 17 nucleic acids and derivative, 15 amines, 11 vitamins, 9 hormones, 6 phenylpropanoids, 3 alkaloids, and 17 unclassified metabolites (Figure 4A and Table S5).

PCA and hierarchical clustering were performed based on the converted contents of all metabolites. Interestingly, the 25 barley accessions were also separated into three clusters (Figure 4B). Moreover, Wb-T resided between Wb-NE and Cb-C, indicating that metabolic divergence might have occurred during the evolution from Wb-NE to Wb-T and from Wb-T to Cb-C. Similar results of PCA were obtained by the hierarchical cluster tree (Figure 4C).

**Figure 5:**
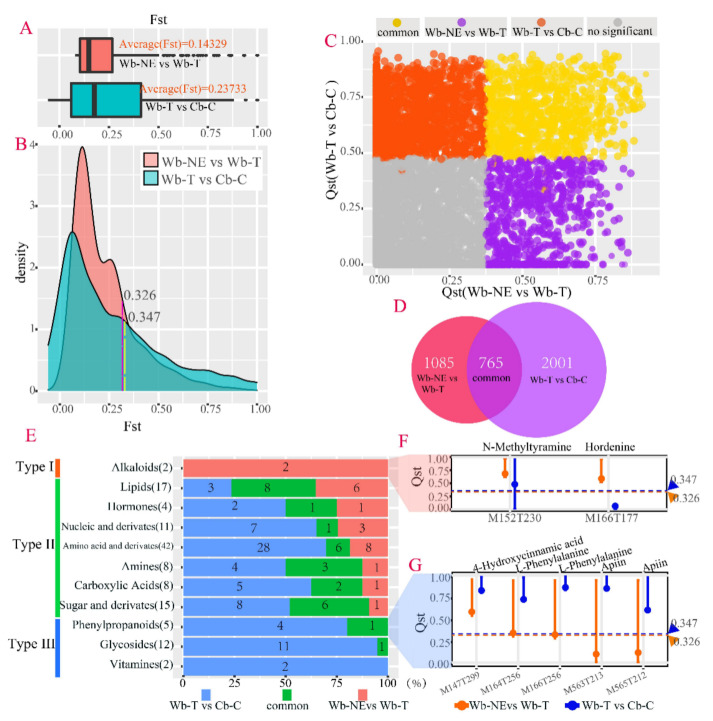
Identification of selective metabolites in barley accessions of Wb-NE vs. Wb-T and Wb-T vs. Cb-C. (**A**) Box plots of Fst in Wb-NE vs. Wb-T and in Wb-T vs. Cb-C. Boxes represented the inter-quartile ranges. The lines across the boxes, the whiskers, and the black circles represent the median values, the 10th, and 90th percentiles and outliers, respectively. (**B**) Density diagrams of Fst in Wb-NE vs. Wb-T and in Wb-T vs. Cb-C. Fst values were higher than 0.326 (the purple dotted line) and higher than 0.347 (the yellow dotted line), indicating that SNPs were under natural or artificial selection in the evolutionary stage from Wb-NE to Wb-T and from Wb-T to Cb-C, respectively. (**C**) Qst distribution associated with Wb-NE vs. Wb-T and Wb-T vs. Cb-C. For each metabolite, Qst was calculated separately for Wb-NE vs. Wb-T and Wb-T vs. Cb-C. The metabolites that exhibited selective status in Wb-NE vs. Wb-T, Wb-T vs. Cb-C, and both are represented by purple circles, red circles, and orange circles, respectively. (**D**) Venn diagram showing the number of selective metabolites in Wb-NE vs. Wb-T, Wb-T vs. Cb-C, and common. (**E**) Different metabolite classes exhibited distinct divergence patterns. The bar chart shows the number of selective metabolites in the corresponding evolutionary process. (**F**,**G**) Illustration of the divergent pattern using alkaloids (**F**) and phenylpropanoids (**G**). Bars and dots represent 99% CIs of Qst and observed Qst, respectively. Red bars with above 0.326 indicate that corresponding metabolites were selective in Wb-NE vs. Wb-T, and blue bars with above 0.347 indicate that corresponding metabolites were selective in Wb-T vs. Cb-C.

The Qst–Fst comparison strategy was used to identify the specific metabolites that were targeted by selection or by neutral processes in domesticated stages from Wb-NE to Wb-T and from Wb-T to Cb-C. Qst and Fst were used to estimate the divergence for metabolic level and for molecular level between groups, respectively. At the molecular level, the averaged Fst from Wb-T to Cb-C (0.23733) was much higher than that from Wb-NE to Wb-T (0.14329) (Figure 5A). The SNPs were removed with Fst > 0.326 in the stage from Wb-NE to Wb-T, and with Fst > 0.347 from Wb-T to Cb-C because the SNPs exhibited selective status (*p* value < 0.01) (Figure 5B). After removing SNPs that deviated from neutrality process, 51,097 and 96,208 neutral SNPs were retained in domesticated stages from Wb-NE to Wb-T and from Wb-T to Cb-C, respectively (data not shown). At the metabolic level, 99% confidence intervals (CIs) of Qst at each metabolite was compared with Fst of neutral SNPs to determine divergent metabolites driven by selection. As a result, of the 8828 non-redundant metabolites (Table S4), 3851 metabolites showed significant selection signals in Wb-NE vs. Wb-T and Wb-T vs. Cb-C (Figure 5C,D), including 1850 metabolites in evolutionary stages from Wb-NE to Wb-T and 2766 metabolites from Wb-T to Cb-C (Figure 5D, Tables S6 and S7). Among the 8828 metabolites, only 765 (8.67%) metabolites were commonly targeted in both domesticated stages (Figure 5C,D), suggesting that different sets of metabolites were targeted for selection in different evolutionary stages of Cb-C.

Among the 328 structure-annotated metabolites (Table S5), there were 132 metabolites (126 classified and 6 unclassified metabolites) showing selective status or divergence in evolutionary stages from Wb-NE to Wb-T and from Wb-T to Cb-C (Table S8 and Figure S7), including 55 metabolites between Wb-NE and Wb-T (Table S9) and 108 metabolites between Wb-T and Cb-C (Table S10). To determine which class of metabolites was involved in a specific domesticated stage, we analyzed the distribution of 11 metabolite classes from 126 classified metabolites. As a result, one metabolite class (alkaloids) was specially targeted in Wb-NE vs. Wb-T. Seven metabolite classes (lipids, hormones, nucleic acids and derivatives, amino acids and derivatives, amines, carboxylic acids, sugar and derivatives) were targeted in both Wb-NE vs. Wb-T and Wb-T vs. Cb-C. Three metabolite classes (phenylpropanoids, glycosides, and vitamins) were specially targeted in Wb-T vs. Cb-C (Figure 5E). The results suggest that alkaloids and phenylpropanoids played an important role in the evolutionary stage from Wb-NE to Wb-T and from Wb-T to Cb-C, respectively.

### 2.5. Positive Selective Genes and SNPs Influencing the Metabolic Divergence

**Figure 6:**
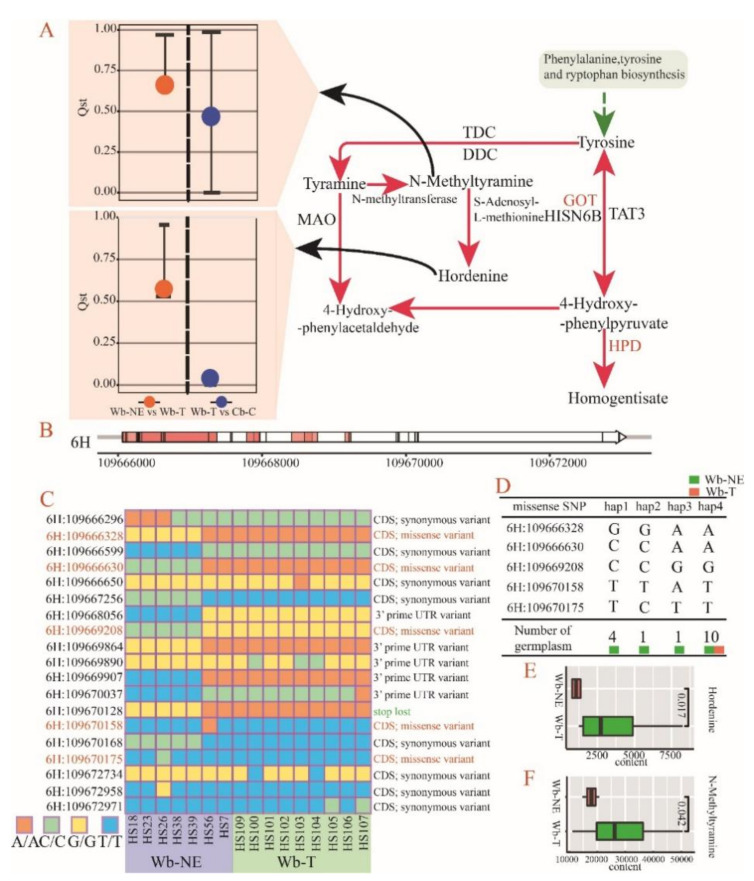
*HORVU6Hr1G027650*, a key gene regulating 4-hydroxyphenylpyruvate dioxygenase (HPD), and its haplotype distribution. Wb-NE, wild barley from the Near East Fertile Crescent. Wb-T, wild barley from the Tibetan Plateau. Cb-C, cultivated barley from China. (**A**) biosynthesis of N-Methyltyramine and Hordenine. HPD, 4-hydroxyphenylpyruvate dioxygenase. GOT, aspartate aminotransferase. DDC, aromatic-L-amino-acid/L-tryptophan decarboxylase. Bars and dots represent 99% CIs of Qst and observed Qst, respectively. (**B**) Gene structure of *HORVU6Hr1G027650*, the red regions represent the coding region of the gene. The vertical lines in the gene indicate the positions of SNPs. The ruler under the gene indicates the position of the gene on the 6H chromosome. (**C**) Haplotype diversity of Wb-NE and Wb-T in *HORVU6Hr1G027650*. (**D**) Four detected haplotypes in accessions of Wb-NE and Wb-T. Haplotypes were composed of five missense SNPs. (**E**,**F**) Comparison of contents of Hordenine (**E**) and N-Methyltyramine (**F**) between Wb-NE with haplotype 1 (four accessions) and Wb-T with haplotype 4 (nine accessions).

The three pure barley groups described in the genomic similarity analysis were also used to calculate a sliding windowed fixation index (Fst) between groups. Genomic regions with the top 5% Fst thresholds (0.4314 in Wb-NE vs. Wb-T and 0.7205 in Wb-T vs. Cb-C) were selected to conduct selective sweep analysis and locate genes influenced by natural selection and/or artificial selection (Figure S8). As a result, 1010 and 930 selective genes were detected in Wb-NE vs. Wb-T and in Wb-T vs. Cb-C (Tables S11 and S12), respectively. Two alkaloid compounds (N-Methyltyramine and Hordenine) exhibited specific divergence in the evolutionary stage from Wb-NE to Wb-T (Figure 5F and Figure 6A). Furthermore, four of 1010 positive selective genes were involved in the same regulatory pathway with the two divergent compounds (Table 2). The four selective genes regulate alcohol dehydrogenase, acylpyruvate hydrolase (FAHD1), aspartate aminotransferase (GOT1), and 4-hydroxyphenylpyruvate dioxygenase (HPD). Among the four genes, *HORVU3Hr1G073220* and *HORVU6Hr1G027650* directly influence N-Methyltyramine and Hordenine (Figure 6A).

The gene *HORVU6Hr1G027650* exhibits close regulatory relationship with N-Methyltyramine and Hordenine, and 19 SNPs associated with this gene were detected (Figure 6B). Five missense mutations in the coding region of *HORVU6Hr1G027650* were discovered with 4 haplotypes in 16 accessions from Wb-NE and Wb-T (Figure 6C,D). Hap1 to hap4 were observed in Wb-NE, whereas only the hap4 was found in Wb-T (Figure 6D). Moreover, hap1 contained the most accessions (four accessions) of Wb-NE, whereas hap4 contained all accessions (nine accessions) of Wb-T (Figure 6D). A “*t*-test” between the four Wb-NE accessions with hap1 and nine Wb-T accessions with hap4, showed that both N-Methyltyramine and Hordenine in Wb-T exhibited significantly more content than in Wb-NE (Figure 6E,F). These results indicate that the five missense SNPs of *HORVU6Hr1G027650* could have affected the divergence of alkaloid metabolites at the evolutionary stage from Wb-NE to Wb-T.

**Figure 7:**
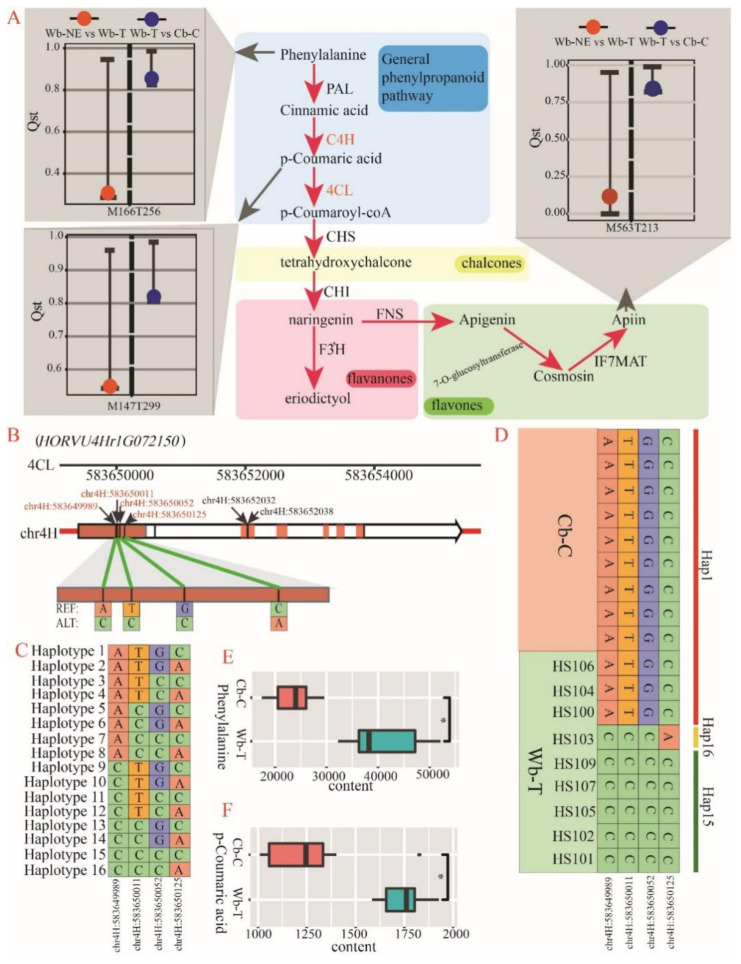
*HORVU4Hr1G072150*, a key gene regulating phenylpropanoid metabolites, and its haplotype distribution. Wb-NE, wild barley from the Near East Fertile Crescent. Wb-T, wild barley from the Tibetan Plateau. Cb-C, cultivated barley from China. (**A**) Phenylpropanoid metabolic pathway. PAL, phenylalanine ammonia-lyase. C4H, trans-cinnamate 4-monooxygenase. 4CL, 4-coumarate-CoA ligase. Bars and dots represent 99% CIs of Qst and observed Qst, respectively. (**B**) Gene structure of *HORVU4Hr1G072150*, the red regions represent the coding region of the gene. The vertical lines in the gene indicate the positions of SNPs. The ruler above the gene indicates the position of the gene on 4H chromosome. (**C**) 16 haplotypes composed of 4 missense SNPs in *HORVU6Hr1G027650*. (**D**) Three detected haplotypes in accessions of Cb-C and Wb-T. (**E**,**F**) Comparison of contents of Phenylalanine (**E**) and 4-Hydroxycimamic (**F**) between Cb-C with haplotype 1 (nine accessions) and Wb-T with haplotype 15 (five accessions).

We also found that three phenylpropanoids compounds (4-Hydroxycimamic, L-Phenylalanine, and Apiin) exhibited specific divergence in the evolutionary stage from Wb-T to Cb-C (Figure 5G and Figure 7A). There were two positive selective genes that were found to directly regulate these three divergent compounds (especially 4-Hydroxycimamic and L-Phenylalanine). The two selective genes, *HORVU3Hr1G080830* and *HORVU4Hr1G072150*, regulate trans-cinnamate 4-monooxygenase (C4H) and 4-coumarate-CoA ligase (4CL), respectively (Table 3 and Figure 7A).

There are six SNPs in the gene structure of *HORVU4Hr1G072150*, which exhibits close regulatory relationship with L-Phenylalanine and 4-Hydroxycimamic (Figure 7B). All six SNPs in the structure of *HORVU4Hr1G072150* were found in the coding region, and four of them were missense SNPs (Figure 7B). The four missense SNPs generated 16 haplotypes (Figure 7C), but only three haplotypes (hap1, hap15 and hap16) were found in 18 accessions containing Wb-T and Cb-C (Figure 7D). The hap1 contained all accessions (accession number = 9) of Cb-C and three accessions of Wb-T, while the hap15 contained five accessions of Wb-T (Figure 7D). The hap16 only contained one accession of Wb-T (Figure 7D). For the two divergent compounds (L-Phenylalanine and 4-Hydroxycimamic), the “t test” between the nine Cb-C accessions with hap1 and five Wb-T accessions with hap15 indicated that both L-Phenylalanine and 4-Hydroxycimamic exhibited more content in Wb-T than in Cb-C (Figure 7E,F). These findings indicated that the four missense SNPs of *HORVU6Hr1G027650* could have affected the divergence of phenylpropanoid metabolites at the evolutionary stage from Wb-T to Cb-C.

## 3. Discussion

### 3.1. Origin and Domestication Process of Cultivated Barley in China

The domestication of crops and the expansion of agriculture fundamentally reshaped the historical process of human beings [29,30,31]. The Near East Fertile Crescent is the primary habitat of wild barley [32]. However, its isolated populations have spread as far as to North African and European shores of the Mediterranean Basin and the Tibetan Plateau [1]. So far, the domestication process of cultivated barley in China is still under debate [33], because of the controversial origin centers of barley [6,7,8,9,10]. Two hypotheses have been proposed. One suggests that the cultivated barley in China was introduced from the Near East Fertile Crescent [14,34,35], and another proposes that cultivated barley in China might have locally derived from two-rowed or six-rowed wild barley from the Qinghai Tibet Plateau [36,37]. We used transcriptomic and non-targeted metabolic data from 29 accessions and resequencing data from 124 accessions to explore the domestication process of cultivated barley in China. The transcriptomic data revealed that among the three assayed barley groups, Wb-NE has the highest genetic diversity, followed by Wb-T and Cb-C (Table 1). The rooted phylogenetic trees showed that both Wb-T and Cb-C originated from the Near East Fertile Crescent (Figure 1C and Figure S2), which was consistent with the results reported by Zeng et al. [11]. These results favor the hypothesis that cultivated barley in China originated from the Near East Fertile Crescent [11,13]. The PCA scatter plot and inference of population structure showed a separation of wild barley and cultivated barley (Figure 1A,B), which was consistent with the results of previous research [11]. Furthermore, the results of PCA, rooted phylogenetic trees, and demographic history seem to suggest that Wb-T is more likely an intermediate subgroup in the domestication process from Wb-NE to Cb-C (Figure 1A,C and Figure 3B–D). The higher genomic similarity regions were found in Wb-T vs. Cb-C compared with Wb-NE vs. Cb-C (Figure 2A,B), suggesting that Wb-T contributed more genetically to Cb-C than Wb-NE. The result was consistent with results reported by Dai et al. [38].

Since the discovery of Tibetan wild barley, many studies have argued that Tibet was one of the barley domestication centers [6,7,8,9,10], and Tibetan hulless barley (qingke) might have existed in the early stage of barley domestication [38]. However, recent studies found that contemporary qingke was derived from eastern domesticated barley and gained little for local origin in the Tibetan Plateau [11]. Our study seems to suggest that feralization or hybridization might be a primary mechanism for the origin of Tibetan wild barley if we consider the results of demographic history and haplotype detection in *Btr1/btr1* and *Btr2/btr2* loci (Figure 3C,G). These findings together are not in support of a local origin of Wb-T. Additionally, the *Vrs1/vrs1* locus was also detected by other studies, such as Zeng et al. and Pourkheirandish et al., offering additional information that Tibetan wild barley was the product of feralization or hybridization [11,12]. In fact, feralization or de-domestication is common in crops, such as in wheat [39] and rice [40]. In wheat, a 0.8-Mb deletion region in *Brt1/2* homologs on 3D and a genetic locus containing the *TaQ-5A* gene on 5A correlated with a de-domestication episode [39]. Latin American weedy rice originated through extensive hybridization [40]. Our results on wild barley from Tibet seem to follow the same scenarios in wheat [39] and weedy rice [40]. Tibetan wild barleys belonged to pseudo-agriocrithon, and were derived from hybridization of domesticated barley between the *btr1Btr2* and *Btr1btr2* genotypes, followed by recombination between *Btr1* and *Btr2* [11,12]. However, our data offer limited possibilities to explore where the cultivated barley feralized first, nor to infer the origin of Wb-T. Further studies are also needed to infer divergent times among the groups, as the assayed samples were admixed. These efforts will enhance our understanding of the role that Wb-T played in barley domestication.

### 3.2. Metabolic Divergence in the Domestication Process of Cultivated Barley in China

The diffusion process of barley from the Near East Fertile Crescent to far beyond the original range of its ancestors is in the process of constantly adapting to new environments [41]. The adaptation to different environments led to different evolutionary directions of barley, resulting in different ecotypes of wild barley and widely distributed landraces, and finally forming various types of modern cultivated barley [41,42,43]. Throughout the domestication process, significant changes have taken place in the morphology of barley, such as seed shattering (Figure S1), seed dormancy, and seed size (Figure S1 and Table S1) and amount [23]. Morphological changes during domestication were frequently accompanied by coordinated metabolite alterations [24,25]. Our study revealed significant metabolic divergence among the three barley groups (Figure 5B,C). Additionally, Wb-T resided between Wb-NE and Cb-C (Figure 5B,C), further suggesting that Wb-T played an important intermediate role during domestication for barley in China. Interestingly, similar results were obtained in a metabolic study of the domestication process from teosinte to temperate maize [28].

Tibetan wild barley was a rich reservoir of many novel alleles for better adaptation to the severe ecological environment of Tibet, such as high levels of UV-B radiation (about 65 kJ m^−2^ in the summer) [44], low temperatures (average yearly temperature of 7.6 °C) [45], and low barometric pressure (about 650 mbar) [46]. The genetic difference between Wb-T and Wb-NE has been reported previously [38]. Our study generated the first evidence on the metabolic differences between the two groups. Two specific alkaloid metabolites (Hordenine and its precursor N-Methyltyramine) were found to be strongly selected from Wb-NE to Wb-T (Figure 6A). N-Methyltyramine, Hordenine, and Gramine are alkaloids that play a key role in the allelopathy of barley [47], and the mixture of Hordenine and Gramine has the function of phytotoxin, which impacts weeds, insects, and pathogens [48]. Lovett and Hoult found that the continuous breeding and domestication process of cultivated barley reduced the synthesis of Gramine, while it increased the synthesis of Hordenine [49]. Similar results were found in our study, namely that the content of Hordenine and N-Methyltyramine in Wb-T is higher than that in Wb-NE (Figure 6E). These results show that missense mutation of a selective gene, *HORVU6Hr1G027650*, which regulates the two metabolites, might be the genetic cause of the metabolic difference. However, further functional studies of the gene would help verify the hypothesis.

In the domestication process of cultivated barley in China, from Wb-T to Cb-C, three specific phenylpropanoids compounds (4-Hydroxycimamic, L-Phenylalanine, and Apiin) were found to be strongly selected (Figure 7A). The metabolites of the phenylpropanoids metabolism pathway, such as flavonoids, phenols, and lignin, play an important role in plant life activities, are widely involved in biological and abiotic stress, and help plants resist external diseases and insect pests [50]. A recent study pointed out that the excessive accumulation of phenylpropanoids metabolites not only protects against UV-B damage, but also may help resist freezing injury of Tibetan hulless barley [51]. Our results show that phenylpropanoid metabolite content in Wb-T was higher than that in Cb-C (Figure 7E,F). We speculate that the difference in 4-Hydroxycimamic and L-Phenylalanine might be caused by different ecological environments. The selective gene, *HORVU4Hr1G072150*, regulates the two metabolites. Our results show that the missense mutations of the key gene might be responsible for the metabolic difference. Phenylpropanoid metabolites including the three compounds have the function of regulating the absorption of UV by plant leaves and preventing plants from being burned [52]. The accumulation of these metabolites provided chaperone for the survival of wild barley in Tibet under strong UV-B radiation.

## 4. Materials and Methods

### 4.1. Plant Growth Conditions

A total of 29 barley accessions were used in this study, and they were provided by the United States Department of Agriculture (USDA, Washington, DC, USA) and the Huazhong Agricultural University (Wuhan, China) barley germplasm collection (See Table S2 and Figure S1 for details). Each barley accession was seeded in plots (three individuals per plot) for germination with three replications in a controlled greenhouse environment with 16 h of light (at 5000 lux illumination intensity and mixed wavelength ranging from 450 to 660 nm) and 8 h of darkness. The third fully unfolded leaves of the growing plants for each accession were collected for RNA sequencing (one replication per accession) and metabolite profiling (three replications per accession).

### 4.2. RNA Sequencing and Single Nucleotide Polymorphisms (SNPs) Calling

Total RNA of each barley accession was extracted from the mixed third fully unfolded leaves using TRIzol^®^ (Waltham, MA, USA) reagent following the manufacturer’s instructions (Invitrogen). Twenty-nine RNA-Seq libraries were constructed following the TruSeq™ RNA sample preparation kit (Illumina, San Diego, CA, USA) and paired-end libraries were sequenced by an Illumina X platform (2 × 151 bp read length). The raw paired-end reads (FASTQ format) were quality controlled by Trimmomatic (0.38 version) with default parameters [53]. Then, the index of the reference genome of Morex v2 (*H. vulgare* ssp. *vulgare*) [24] was built and clean reads of each accession were separately aligned to the reference genome using hisat2 (2.2.1 version) with default parameters [54]. The SAM files generated by aligning were sorted by SAMtools with the “SortSam” command [55] and duplicates were further marked by picard. The generated BAM file after marking duplicates was used to detect SNPs and short insertions/deletions (InDels) by the SAMtools [55]. The “mpileup” command was used to identify SNPs and InDels with the parameters as “-q 10 -Q 20 -C 50”. After excluding SNP calling errors caused by incorrect mapping and all Indels, only high-quality SNPs (with a coverage depth ≥ 3, mapping quality ≥ 30 and missing ratio = 0) were retained for subsequent analysis.

### 4.3. Genetic Diversity Analysis

Based on 176,741 high-quality SNPs from three barley groups, the nucleotide diversity (π) and Watterson estimator (θ_W_) were calculated using Dnasp5.0 software [56]. The minor allele frequency (MAF) was calculated by combined VCFtools v0.1.16 [57] and Plink (1.90b6.18 version) [58]. The polymorphism information content (PIC) was calculated using the following formula:

$$PIC=1-(\sum_{i=1}^{n}{p_{i}}^{2})-\sum_{i=1}^{n-1}\sum_{j=i+1}^{n}2\;{p_{i}}^{2}{p_{j}}^{2}$$

where p_i_ and p_j_ are the frequencies of the i and j alleles at a locus within the population, respectively. n is the number of alleles at the corresponding locus.

### 4.4. PCA and Population Structure Inference Based on SNPs of RNA-Seq

A principal component analysis (PCA) was performed to evaluate the genetic relationship for samples by the software plink (1.90b6.18 version) based on the 176,741 high-quality SNPs [58]. Then the distribution and 95% confidence interval of populations on PC1 and PC2 were visualized by the ggplot2 package in R. The population genetic structure was inferred using the software ADMIXTURE (1.23 version) [59]. All 29 accessions were used to estimate the genetic ancestry, specifying a K ranging from 2 to 4.

In order to infer the origin of Cb-C, a rooted neighbor-joining tree was constructed based on the sets of 165,848 high-quality SNPs using the software MEGAX with the Tajima–Nei model and 1000 bootstrap estimates [60]. *H. bulbosum* was used as an outgroup [61]. The genotype of *H. bulbosum* in each corresponding SNP site was identified by the following two steps. First, the reference sequences consisting of 200 bp with the SNP site in the 100th bp were mapped to contigs of *H. bulbosum* by the “blastn” command in the blast software packages. Second, the genotype of *H. bulbosum* in each corresponding SNP site was identified by our custom shell script. We obtained 165,848 SNPs for the inference of rooted phylogenetic trees.

### 4.5. Genomic Similarity Analysis

Based on the results of PCA and population structure inference, 25 barley accessions were divided into 3 pure barley groups for genomic similarity analysis. The three groups included the wild barley from the Near East Fertile Crescent (Wb-NE, including HS7, HS18, HS23, HS26, HS38, HS39, and HS56), the wild barley from the Tibetan Plateau (Wb-T, including HS100, HS101, HS102, HS103, HS104, HS105, HS106, HS107, and HS109) and the cultivated barley of China (Cb-C, all of the cultivated barley except for Huangqingke).

The set of 114,120 SNPs in the 25 barley accessions was used for genomic similarity analysis. The analysis had four steps. First, three barley gene pools for the three groups were constructed according to the published method [62]. Briefly, for all sites with two or more variants, the minority variants were treated as errors rather than using the reference base. If the variants in a certain site had the same frequency, they were selected randomly. Second, to maximize the genomic coverage and maintain high accuracy, we used 150 kb windows and 75 kb overlapping slide windows along the barley genome to study the genomic similarity. The number of SNPs was counted for each window, and the windows were removed when the widow had ≤10 SNPs. Third, the number of identical SNPs in each window was counted between Cb-C and Wb-NE, between Cb-C and Wb-T, and between Wb-NE and Wb-T. Then, the genomic similarity (number of identical SNPs/total number of SNPs) of each window was calculated. Lastly, the windows with a similarity ≥95% were retained and were visualized using the Circlize package in R [63].

### 4.6. Demographic History, Migration Event, and Haplotype Detection

In order to gain more insights into the domestication process of Cb-C, we downloaded the published resequencing data of 124 barley accessions from Zeng et al. [11]. These data, along with our 29 RNA sequencing data, were used for the following analysis (Table S13). We obtained 56,349,359 high-quality SNPs in 124 barley accessions. The effective population size (Ne) and divergence time between groups were inferred using SMC++ v1.15.3 [64]. Three distinguished individuals in each group were randomly selected to perform the estimation of effective population size and divergence time. The mutation rate was assumed as μ = 6.5 × 10^−9^ mutations per site per generation. The maximum likelihood trees were constructed when 0 or 1 migration events were allowed using TreeMix 1.13 [65]. Haplotype detection in *Btr1/btr1* and *Btr2/btr2* loci was performed for 153 accessions (29 RNA sequencing and 124 resequencing) and the data were visualized using the heatmap package of R.

### 4.7. Extraction and Detection of Untargeted Metabolites

Untargeted metabolites were extracted from the 25 accessions, with 3 replicates per accession. Analyses were performed using an UHPLC (1290 Infinity LC, Agilent Technologies, Santa Clara, CA, USA) coupled to a quadrupole time-of-flight (AB Sciex TripleTOF 6600) (see Supplementary File S1). The raw data were converted into mzXML format by Proteowizard, and then peak alignment, retention time correction, and peak area extraction were performed using the XCMS program. The structure of metabolites was identified by accurate mass number matching (<25 ppm) and secondary spectrum matching. For each metabolite, the area of the mass spectrum peak was calculated and corrected for preliminary quantification. After that, we searched the self-built database (with standard products) in order to annotate the metabolites.

### 4.8. PCA and Hierarchical Clustering Analysis Based on Metabolites

After filtering, 8828 non-redundant metabolites were retained. Among these non-redundant metabolites, 328 metabolites had annotated structures (Figure S5). All metabolite contents were converted in log2 to enhance data homogeneity for further analysis. PCA and hierarchical cluster analysis were performed using the online tools of metaboanalyst (https://www.metaboanalyst.ca, accessed on 18 October 2021) based on all non-redundant metabolites.

### 4.9. Q_st_–F_st_ Comparison

We used a “Q_st_–F_st_ Comparison” strategy to identify the selective SNPs and divergent metabolites caused by selection rather than neutral processes [66]. The method was as follows:

First, the F_st_ of SNP between Wb-NE and Wb-T and between Wb-T and Cb-C was calculated by using Arlequin3.5.1.3 according to 114,120 SNP sets of 25 samples [67]. Before calculating F_st_, monomorphic SNPs were excluded, the retained SNPs were used to calculate F_st_ according to the method of molecular variance (AMOVA) [29] using the following formula:

F_st_ = б_B_^2^/б_T_^2^

where б_B_^2^ is the variance between groups and б_T_^2^ is the total variance. After calculating, SNPs that deviated from neutral expectations using an F_st_-based outlier test were excluded [29]. The outliers were identified by permuting individuals across groups (the number of permutations was 20,000). The outlier SNPs were discarded (with *p* value < 0.01), and neutral SNPs were retained and used to calculate neutral Fst.

Second, Q_st_ between Wb-NE and Wb-T and between Wb-T and Cb-C was calculated based on metabolites data according to the following formula:

Q_st_ = б_B_^2^/(б_B_^2^ + 2б_W_^2^)

where б_B_^2^ is the variance between groups of converted metabolites content and б_W_^2^ is the variance within groups of converted metabolites content. б_B_^2^ and б_W_^2^ were calculated using the R software package of lme4r with the linear mixed model. The 99% confidence interval (CI) of Q_st_ was calculated by resampling 1000 times [68]. CIs of Q_st_ were compared with the F_st_ value of a neutral SNP, and those metabolites with CIs greater than the neutral F_st_ value were considered as the selective or divergent metabolites.

## Figures and Tables

**Table 1:** Estimate of genetic diversity per base pair for three groups (Wb-NE, Wb-T, and Cb-C).

Group	π	θ_W_	MAF	PIC
Wb-NE	0.23036	0.21255	0.1745	0.2176
Wb-T	0.18906	0.15944	0.1453	0.1796
Cb-C	0.11034	0.10183	0.1120	0.1048

Note: π, nucleotide diversity. θ_W_, Watterson’s estimator. MAF, Minor allele frequency. PIC, Polymorphism information content. Wb-NE, wild barley from the Near East Fertile Crescent. Wb-T, wild barley from the Tibetan Plateau. Cb-C, cultivated barley from China.

**Table 2:** Positive selective genes related to alkaloid compounds in the evolutionary stage from Wb-NE to Wb-T.

Gene ID	KEGG ID	Function
*HORVU1Hr1G010130*	K00001	alcohol dehydrogenase
*HORVU4Hr1G013370*	K01557	FAHD1, acylpyruvate hydrolase
*HORVU3Hr1G073220*	K14454	GOT1, aspartate aminotransferase
*HORVU6Hr1G027650*	K00457	HPD, 4-hydroxyphenylpyruvate dioxygenase

**Table 3:** Positive selective genes involved in phenylpropanoid metabolites.

Gene ID	KEGG ID	Function
*HORVU3Hr1G080830*	K00487	C4H, CYP73A, trans-cinnamate 4-monooxygenase
*HORVU4Hr1G072150*	K01904	4CL, 4-coumarate-CoA ligase

## Data Availability

The sequences data that support the findings of this study have been deposited in NCBI under the BioProject PRJNA744021 with the Sequence Read Archive (SRA) number SRP327777.

## Supplementary Materials

The following materials are available online at https://www.mdpi.com/article/10.3390/plants11020209/s1, Figure S1: The geographic distribution of three sampled barley groups and morphology of grains and spikes; Figure S2: Original tree based on Tajima–Nei model; Figure S3: Gene flow between barley groups; Figure S4: Residual likelihood plots of the maximum-likelihood trees for the four barley groups; Figure S5: PCA scatter plot of the four barley groups; Figure S6: Population structure of the four groups; Figure S7: Venn diagrams of metabolites annotated by selection during two evolutionary stages; Figure S8: Positive selection signals in evolutionary stages from Wb-NE to Wb-T and from Wb-T to Cb-C; Table S1: Eight morphological traits of grain in three barley groups; Table S2: Code, taxon, origin, raw reads, clean reads, Q20, Q30, and GC content of 29 barley accessions; Table S3: The optimal number of population structure judged by admixture software; Table S4: The 8828 non-redundant metabolites; Table S5: Metabolites with an annotated structure; Table S6: Divergent metabolites between Wb-NE and Wb-T; Table S7: Divergent metabolites between Wb-T and Cb-C; Table S8: Selective annotated metabolites; Table S9. Selective annotated metabolites between Wb-NE and Wb-T; Table S10. Selective annotated metabolites between Wb-T and Cb-C; Table S11: Selective genes between Wb-NE and Wb-T; Table S12: Selective genes between Wb-T and Cb-C; Table S13: All accessions used in this study, Supplementary File S1: Supplementary Materials and Methods [57,58,59,69,70].
