# Supplementary material for: Transcriptome and Metabolite Insights into Domestication Process of Cultivated Barley in China
Source: Plants (Basel). 2022 Jan 14;11(2):209. doi: 10.3390/plants11020209 (PMC8779797; doi:10.3390/plants11020209)
Supplement: Supplementary file 1 [file plants-11-00209-s001.zip › supplementary information/supplementary information.pdf]

Transcriptome and Metabolite Insights into Domestication Process of Cultivated Barley in China

Yu Zhou [1,2,†], Guang Lu [1,†], Genlou Sun[3], Daokun Sun[1] and Xifeng Ren[1,2,*]

Supplementary Materials and methods

1. Determination of Eight Grain Morphology Traits

The 29 barley accessions used in this study included nine wild accessions from the Near East Fertile Crescent (Wb-NE), ten wild accessions from Tibetan Plateau (Wb-T) and ten cultivated accessions from China (Wb-C) (Figure S1), which were provided by the United States Department of Agriculture (USDA) and the Huazhong Agricultural University barley germplasm collection (See Table S2 for details). Eight grain morphological traits (grain area, grain perimeter, grain length, grain width, length-width ratio, roundness of grain, density factor and grain diameter) were measured by using a camera-assisted phenotyping system (SC-G, Wanshen Detection Technology Co., Ltd., Hangzhou, China) (Yin et al 2015). SPSS 24.0 software was used for one way ANOVA analysis of all measured traits in the three groups.

2. ABBA-BABA Analysis and Treemix analysis

The VCF file containing 165,848 high-quality SNPs was used to perform ABBA-BABA analysis using the software Dsuite (0.4 version) with "Dtrios" command (Malinsky et al 2020). The ABBA-BABA statistics (D statistics) were applied to biallelic SNPs across four groups or taxa: P1, P2, P3, and O based onthe rooted tree (((P1,P2),P3),O) (Malinsky et al 2020). According to the results of rooted neighbor-joining tree (Figure 1C), HS18 (a wild barley accession from the Near East Fertile Crescent) was selected as outgroup because it occupied the "O" position in the rooted tree (((P1,P2),P3),O). *H. bulbosum* was not used as an outgroup because after adding *H. bulbosum*, many SNPs were not biallelic but triallelic, which violated the requirements of ABBA-BABA test. The treemix1.13 was used to test mixture events of the three groups based on the 165,848 high quality SNPs.

3. PCA and Population Structure Inference based on SNPs of All Accessions

The 153 barley accessions (29 RNA-sequencing and 124 re-sequencing) were used to infer the genetic relationship of barley groups. These accessions were assigned into four groups: Wb-NE (13 accessions), Cb-NE (cultivated barley from the Near East Fertile Crescent, six accessions), Wb-T (20 accessions) and Cb-C (114 accessions representing landraces from China, specially from Tibet). The 78,861 SNPs common identified in both the VCF files of the 29 RNA-sequencing accessions and the 124 re-sequencing accessions were used to perform PCA and population structure inference. PCA and population structure inference were performed by plink (1.90b6.18 version) (Purcell et al 2007) and ADMIXTURE (1.23 version) (Alexander et al 2009), respectively. Nucleotide diversity (π) of four groups was analyzed using VCFtools v0.1.16 (Danecek et al 2011).

4. Selective Sweep Analysis and Identification of Selective Genes

The 114,120 high-quality SNPs randomly distributed on chromosomes were used for the selective sweep analysis for two of the three pure groups (25 accessions) by measuring Fst in each 15-kb genomic fragment with a step of 5 kb along all chromosomes. Genomic regions under selective sweeps were measured by the sliding windowed Fst using VCFtools v0.1.16 (Danecek et al 2011). The windows with the top 5% Fst value were considered as putative selection regions. The selective genes were considered to be in the windows with the top 5% Fst values (0.4314 in Wb-NE vs Wb-T and 0.7205 in Wb-T vs Cb-C), and were annotated by KEGG database.

5. Extraction and Detection of Untargeted Metabolites

Untargeted metabolites were extracted from the 25 accessions, with 3 replicates per accession. Analyses were performed using an UHPLC (1290 Infinity LC, Agilent Technologies) coupled to a quadrupole time-of-flight (AB Sciex TripleTOF 6600). For HILIC separation, samples were analyzed using a 2.1 mm × 100 mm ACQUIY UPLC BEH 1.7 µm column (waters, Ireland). In both ESI positive and negative modes, the mobile phase contained A = 25 mM ammonium acetate and 25 mM ammonium hydroxide in water, and B = acetonitrile. The gradient was 85% B for one min and was linearly reduced to 65% in 11 min, and then was reduced to 40% in 0.1 min and kept for four min, and then increased to 85% in 0.1 min, with a five min re-equilibration period employed. The ESI source conditions were set as follows: Ion Source Gas1 (Gas1) as 60, Ion Source Gas2 (Gas2) as 60, curtain gas (CUR) as 30, source temperature: 600℃, IonSpray Voltage Floating (ISVF) ±5500 V. In MS only acquisition, the instrument was set to acquire over the m/z range of 60-1000 Da, and the accumulation time for TOF MS scan was set at 0.20 s/spectra. In auto MS/MS acquisition, the instrument was set to acquire over the m/z range of 25-1000 Da, and the accumulation time for product ion scan was set at 0.05 s/spectra. The product ion scan was acquired using information dependent acquisition (IDA) with high sensitivity mode selected. The collision energy (CE) was fixed at 35 V with ±15 eV. Declustering potential (DP) was set as ±60V.

After detection, AB Sciex TripleTOF 6600 was used to collect the primary and secondary spectra. The raw data was converted into mzXML format by Proteowizard, and then peak alignment, retention time correction and peak area extraction were performed by XCMS program. The structure of metabolites was identified by accurate mass number matching (< 25 ppm) and secondary spectrum matching. For each metabolite, the area of mass spectrum peak was calculated and corrected for preliminary quantification. After that, we searched the self-built database (with standard products) for annotating the metabolites.

Supplementary Figures and Tables

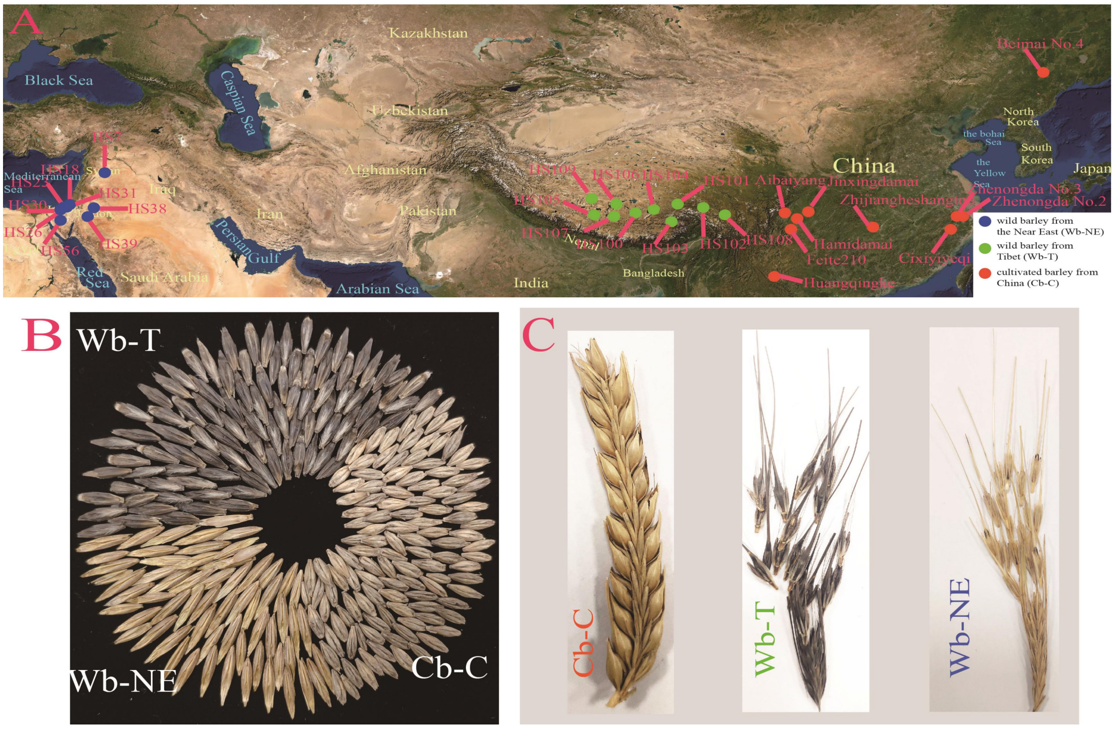

Figure S1. The geographic distribution of sampled three barley groups and morphology of grains and spikes. The satellite map was provided by software ShuiJingWeiTu.

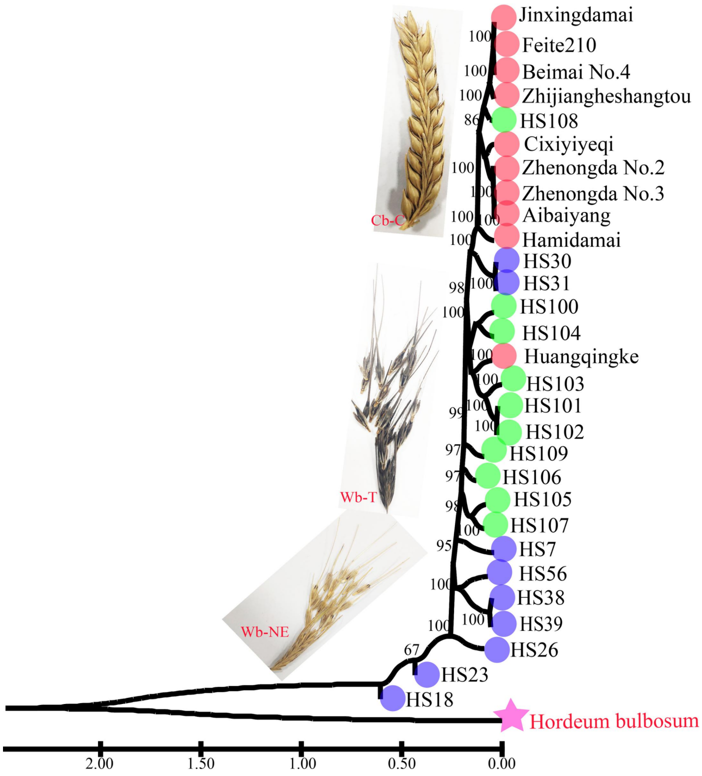

Figure S2. Original tree based on Tajima-Nei model. The tree was drawn to scale with branch lengths in the same units as those of the evolutionary distances used to infer the phylogenetic tree.

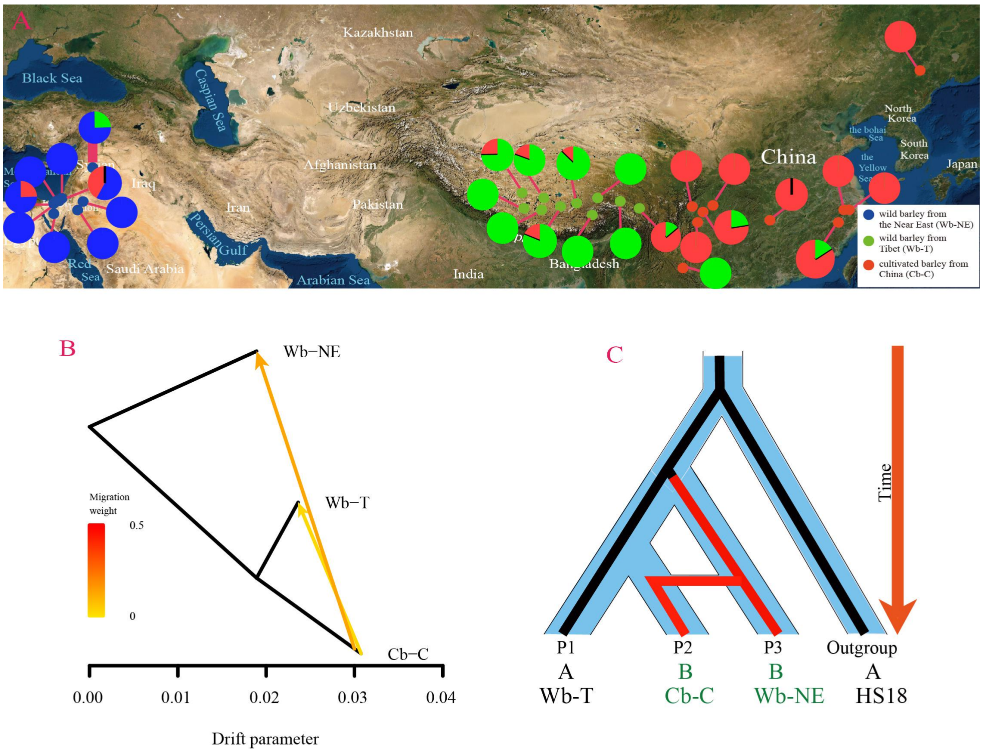

D

P1	P2	P3	Outgroup	Dstatistic	Z-score	p-value
Wb-T	Cb-C	Wb-NE	HS18	0.167869	6.0212	$8.65662e^{-10}$
Wb-NE	Cb-C	Wb-T	HS18	0.314726	12.6609	0
Wb-NE	Wb-T	Cb-C	HS18	0.155049	4.60531	$2.0593e^{-06}$

Figure S3. Gene flow between barley groups. Wb-NE, wild barley from the Near East Fertile Crescent. Wb-T, wild barley from Tibetan Plateau. Cb-C, cultivated barley from China. (A) Membership coefficient of each accession when K=3. The small red circles, small green circles and small blue circles denoted accessions of Cb-C, Wb-T and Wb-NE, respectively. The colors of the pie charts corresponded to the ADMIXTURE results with K = 3 in Figure 1B. The satellite map was provided by software ShuiJingWeiTu. (B) The maximum likelihood tree with two mixture event. (C) The optimal dtrios model based on the sets of 165,848 high-quality SNPs and HS18 as the outgroup revealed by software Dsuite (0.4 version). P1, P2 and P3 denoted the population 1, the population 2 and the population 3, respectively. (D) three trios model based on HS18 as the outgroup.

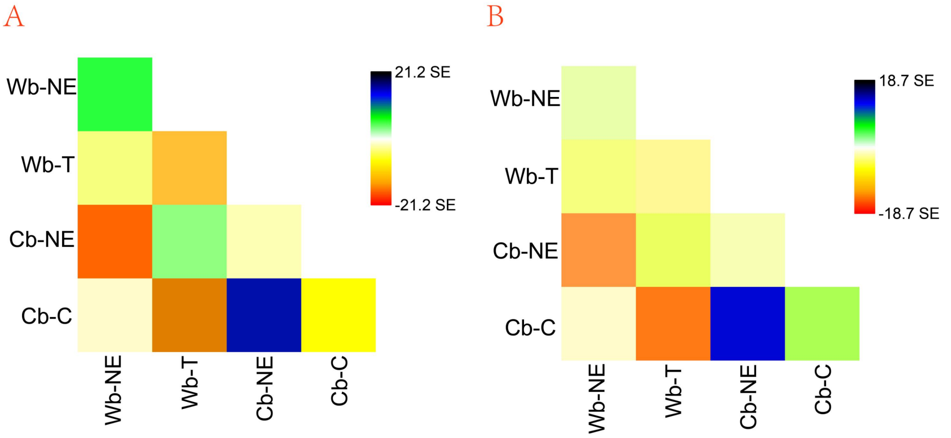

Figure S4. Residual likelihood plots of the maximum-likelihood trees for the four barley groups. Wb-NE, wild barley from the Near East Fertile Crescent. Wb-T, wild barley from Tibetan Plateau. Cb-C, cultivated barley from China. Cb-NE, cultivated barley from the Near East Fertile Crescent. (A) and (B) denoted residual likelihood plots when 0 and 1 migration events were allowed, respectively.

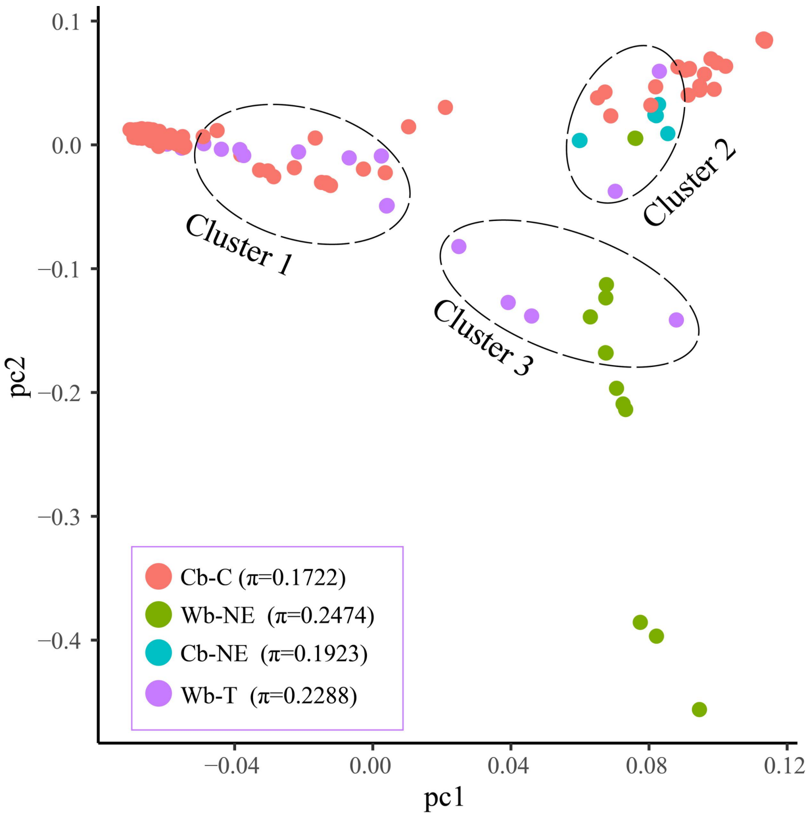

Figure S5. PCA scatter plot of the four barley groups. Wb-NE, wild barley from the Near East Fertile Crescent. Wb-T, wild barley from Tibetan Plateau. Cb-C, cultivated barley from China. Cb-NE, cultivated barley from the Near East Fertile Crescent. pc1, the first principal component. pc2, the second principal component. The Cluster 1, Cluster 2 and Cluster 3 showed close genetic relationship of Wb-T accessions with Cb-C, Cb-NE and Wb-NE, respectively.

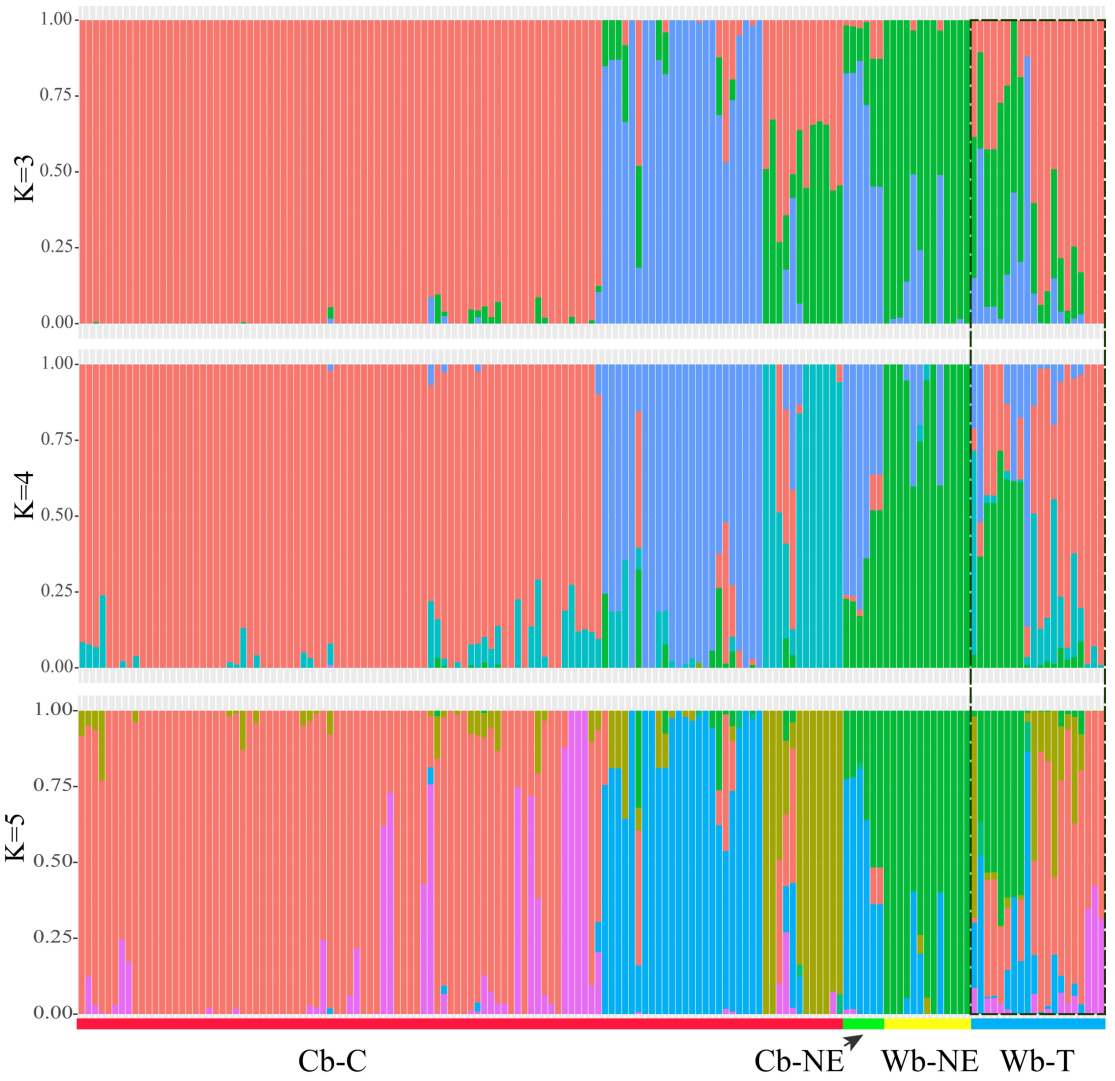

Figure S6. Population structure of the four barley groups. Wb-NE, wild barley from the Near East Fertile Crescent. Wb-T, wild barley from Tibetan Plateau. Cb-C, cultivated barley from China. Cb-NE, cultivated barley from the Near East Fertile Crescent.

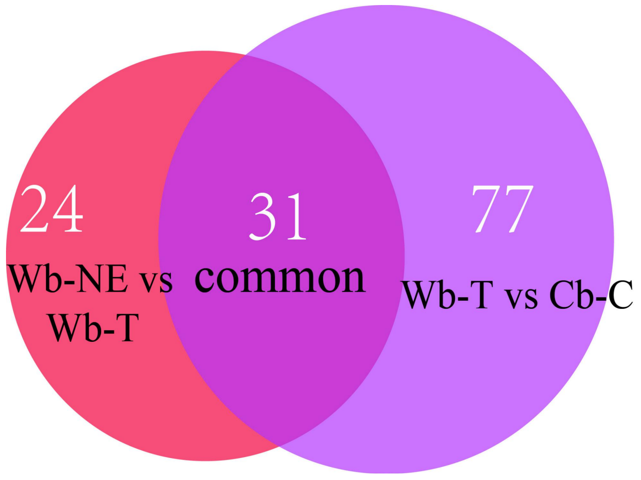

Figure S7. Venn diagrams of metabolites annotated by selection during two evolutionary stages.

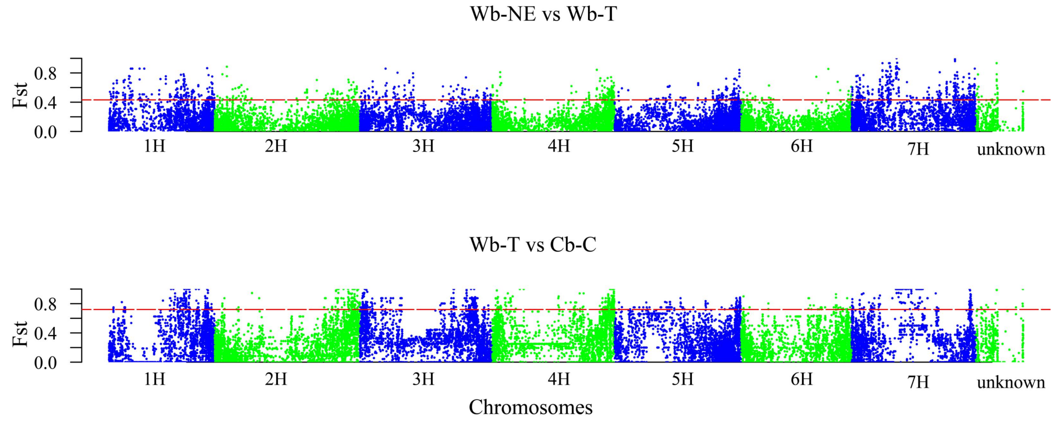

Figure S8. Positive selection signals in evolutionary stages from Wb-NE to Wb-T and from Wb-T to Cb-C. Wb-NE, wild barley from the Near East Fertile Crescent. Wb-T, wild barley from Tibetan Plateau. Cb-C, cultivated barley from China. Genome-wide distribution of genetic differentiation regions identified by Fst analysis between Wb-NE and Wb-T, and between Wb-T and Cb-C, respectively. Each point indicated a weighted Fst value with a 15-kb window. Red dotted lines indicated the thresholds of Fst value (0.4314 in Wb-NE vs Wb-T and 0.7205 in Wb-T vs Cb-C) in the top 5% windows.

Table S1. Eight morphological traits of grain in three barley groups.

**Group**	**Grain area** (mm^2)	**Grain perimeter** (mm)	**Length width ratio**	**Grain length** (mm)	**Grain width** (mm)	**Roundness of grain**	**Density factor** (mg/mm^2)	**Grain diameter** (mm)
Wb-NE	26.20[a]	27.56[a]	4.05[a]	12.04[a]	3.00[b]	0.25[c]	0.90[b]	5.74[a]
Wb-T	24.70[a]	24.66[b]	3.20[b]	10.51[b]	3.30[a]	0.31[b]	1.10[a]	5.57[a]
Cb-C	22.62[a]	22.05[b]	2.78[b]	9.20[c]	3.34[a]	0.35[a]	1.12[a]	5.35[a]

Note: Wb-NE, wild barley from the Near East Fertile Crescent. Wb-T, wild barley from Tibetan Plateau. Cb-C, cultivated barley from China.

Table S2. Code, Taxon, Origin, Raw reads, Clean reads, Q20, Q30 and GC content of 29 barley accessions.

Group	Accessions ID	NCBI ID	Code	Taxon	Origin	Raw reads	Clean reads	Clean bases (G)	Q20(%)	Q30(%)	GC content (%)
Cb-C	Aibaiyang	SAMN20065704	381（Aibaiyang）	*Hordeum vulgare*	Sichuan, China	44,167,002	43,963,564	5.99	97.95	94	55.14
	Cixiyiyeqi	SAMN20065705	385（Cixiyiyeqi）	*Hordeum vulgare*	Zhejiang, China	43,699,046	43,500,878	7.14	97.99	94.14	53.94
	Beimai No.4	SAMN20065706	Beimai no.4	*Hordeum vulgare*	Jilin,China	43,103,444	42,838,678	5.95	97.68	93.57	55.56
	Huangqingke	SAMN20065707	Huangqingke	*Hordeum vulgare*	Yunnan,china	42937834	42,701,836	5.83	97.71	93.56	55.43
	Zhijiangheshangtou	SAMN20065708	Zhijiangheshangtou	*Hordeum vulgare*	Hunan, China	43,948,882	43,728,622	5.88	97.95	94.07	54.97
	Hamidamai	SAMN20065709	Hamidamai	*Hordeum vulgare*	Sichuan, China	43,537,552	43,343,810	5.96	98	94.18	54.93
	Jinxingdamai	SAMN20065710	Jinxingdamai	*Hordeum vulgare*	Sichuan, China	42,103,686	41,912,218	5.89	97.99	94.16	54.97
	Feite210	SAMN20065711	Feite210	*Hordeum vulgare*	Sichuan, China	45,487,962	45,251,466	5.67	97.82	93.71	54.41
	Zhenongda No.2	SAMN20065712	Zhenongda No.2	*Hordeum vulgare*	Zhejiang, China	44,143,792	43,935,392	6.21	97.94	94.1	53.48
	Zhenongda No.3	SAMN20065713	Zhenongda No.3	*Hordeum vulgare*	Zhejiang, China	52,555,774	52,302,084	5.87	97.92	94.06	53.81
Wb-NE	HS7	SAMN20065695	PI236386	*Hordeum spontaneum*	Syria	56,614,596	56,307,586	7.66	97.85	93.94	55.32
	HS18	SAMN20065696	PI284752	*Hordeum spontaneum*	Israel	57,939,324	57,653,976	7.81	97.89	93.98	54.69
	HS23	SAMN20065697	PI296792	*Hordeum spontaneum*	Israel	40,438,926	40,264,256	5.43	98.08	94.39	54.7
	HS26	SAMN20065698	PI296878	*Hordeum spontaneum*	Israel	53,673,492	53,431,814	7.18	97.99	94.2	54.8
	HS30	SAMN20065699	PI356209	*Hordeum spontaneum*	Israel	49,060,986	48,852,090	6.47	98.11	94.46	54.31
	HS31	SAMN20065700	PI391100	*Hordeum spontaneum*	Israel	54,550,880	54,254,452	7.17	97.87	93.97	54.04
	HS38	SAMN20065701	PI420916	*Hordeum spontaneum*	Jordan	42,894,626	42,672,602	5.81	97.88	93.98	55.02
	HS39	SAMN20065702	PI420917	*Hordeum spontaneum*	Jordan	40,180,374	40,007,502	5.48	98.04	94.31	54.85
	HS56	SAMN20065703	PI466524	*Hordeum spontaneum*	Israel	42,473,328	42,293,454	5.79	98.05	94.32	55.76
Wb-T	HS100	SAMN20065685	01	*Hordeum spontaneum*	Tibet, China	42,180,230	41,971,336	5.66	97.94	94.13	54.91
	HS101	SAMN20065686	03	*Hordeum spontaneum*	Tibet, China	47,238,978	46,998,118	6.35	97.93	94.1	55.19

HS102	SAMN20065687	04	*Hordeum spontaneum*	Tibet, China	54,444,286	54,135,064	7.31	97.73	93.67	56.25
HS103	SAMN20065688	13	*Hordeum spontaneum*	Tibet, China	50,074,954	49,815,964	6.78	97.91	94.07	55.17
HS104	SAMN20065689	18	*Hordeum spontaneum*	Tibet, China	57,520,602	57,239,464	7.87	97.91	94	54.07
HS105	SAMN20065690	22	*Hordeum spontaneum*	Tibet, China	47,252,228	47,025,550	6.32	98.01	94.29	55.32
HS106	SAMN20065691	23	*Hordeum spontaneum*	Tibet, China	43,197,490	42,989,676	5.87	97.89	93.9	54.86
HS107	SAMN20065692	26	*Hordeum spontaneum*	Tibet, China	41,936,398	41,676,472	5.64	97.67	93.53	54.97
HS108	SAMN20065693	27	*Hordeum spontaneum*	Tibet, China	43,112,326	42,918,498	5.82	97.96	94.13	56.31
HS109	SAMN20065694	28	*Hordeum spontaneum*	Tibet, China	65,689,076	65,320,088	8.79	97.88	94.01	55.89
Sum		-	-		1,376,158,074	1,369,306,510	185.6	-	-	-
Average		-	-		45,871,935.80	45,643,550.30	6.19	-	-	-

Note: Wb-NE, wild barley from the Near East Fertile Crescent. Wb-T, wild barley from Tibetan Plateau. Cb-C, cultivated barley from China.

Table S3. The optimal number of population structure judged by admixture software.

K	CV error
2	0.82365
3	0.83402
4	0.95663

Table S4. The 8,828 non redundant metabolites (See "Table S4.xls" for detail).

.

Table S5. Metabolites had annotated structure.

Metabolite ID	Classification	Description	KEGG.ID
M166T177	Alkaloid	Hordenine	C06199
M152T230	Alkaloid	N-Methyltyramine	
M163T79	Alkaloid	Nicotine	C00745
M159T178	Amines	Allantoin	C01551
M157T181	Amines	Allantoin	C01551
M131T401	Amines	Agmatine	C00179
M177T242	Amines	Serotonin	C00780
M150T167	Amines	Triethanolamine	C06771
M102T335	Amines	Betaine aldehyde	C00576
M104T405	Amines	Choline	C00114
M100T59	Amines	Cyclohexylamine	C00571
M184T496	Amines	Phosphorylcholine	C00588
M318T162	Amines	Phytosphingosine	C12144
M242T389	Amines	Phosphorylcholine	C00588
M118T2722	Amines	Betaine	C00719
M136T299	Amines	Dopamine	C03758
M152T100	Amines	Norepinephrine	C00547
M138T234	Amines	Tyramine	C00483
M148T92	Amino acid and derivative	O-Acetyl-L-serine	C00979
M232T452	Amino acid and derivative	N-（omega）-Hydroxyarginine	C05933
M143T240	Amino acid and derivative	N6-Methyl-L-lysine	C02728
M193T84	Amino acid and derivative	N-Methylanthranilic Acid	
M205T258	Amino acid and derivative	L-Tryptophan	C00078
M203T258	Amino acid and derivative	L-Tryptophan	C00078
M116T309	Amino acid and derivative	D-Proline	C00763
M134T392	Amino acid and derivative	L-Aspartate	C00049
M102T513	Amino acid and derivative	1-Aminocyclopropanecarboxylic acid	C01234
M201T335	Amino acid and derivative	5-L-Glutamyl-L-alanine	C03740
M161T316	Amino acid and derivative	D-Alanyl-D-alanine （D-Ala-D-Ala）	C00993
M166T366	Amino acid and derivative	DL-Methionine sulfoxide	C02989
M308T393	Amino acid and derivative	Glutathione	C00051
M613T481	Amino acid and derivative	Glutathione disulfide	C00127
M175T529	Amino acid and derivative	L-Arginine	C00062
M176T391	Amino acid and derivative	L-Citrulline	C00327
M148T386	Amino acid and derivative	L-Glutamate	C00025
M156T450	Amino acid and derivative	L-Histidine	C00135
M132T168	Amino acid and derivative	L-Leucine	C00123
M106T375	Amino acid and derivative	L-Serine	C00065
M189T544	Amino acid and derivative	N6,N6,N6-Trimethyl-L-lysine	C03793
M203T512	Amino acid and derivative	NG,NG-dimethyl-L-arginine（ADMA）	C03626

M132T392	Amino acid and derivative	D-Aspartic acid	C00402
M178T36	Amino acid and derivative	3,4-Dihydroxy-L-phenylalanine （L-DOPA）	C00355
M172T318	Amino acid and derivative	Acetyl-DL-Leucine	C02710
M175T346	Amino acid and derivative	Allantoate/Allantoic acid	C00499
M159T317	Amino acid and derivative	D-Alanyl-D-alanine （D-Ala-D-Ala）	C00993
M275T439	Amino acid and derivative	gamma-L-Glutamyl-L-glutamic acid	C05282
M245T365	Amino acid and derivative	gamma-L-Glutamyl-L-valine	C03740
M88T345	Amino acid and derivative	L-Alanine	C00041
M146T385	Amino acid and derivative	L-Glutamate	C00025
M145T371	Amino acid and derivative	L-Glutamine	C00064
M130T272	Amino acid and derivative	L-Isoleucine	C00407
M128T307	Amino acid and derivative	L-Pipecolic acid	C00408
M114T310	Amino acid and derivative	L-Proline	C00148
M104T375	Amino acid and derivative	L-Serine	C00065
M116T299	Amino acid and derivative	L-Valine	C00183
M130T363	Amino acid and derivative	N-Acetyl-L-alanine	
M188T371	Amino acid and derivative	N-Acetyl-L-glutamate	C00624
M206T307	Amino acid and derivative	N-Acetyl-L-phenylalanine	C03519
M133T374	Amino acid and derivative	L-Asparagine	C00152
M120T365	Amino acid and derivative	L-Threonine	C00188
M182T299	Amino acid and derivative	L-Tyrosine	C00082
M131T374	Amino acid and derivative	L-Asparagine	C00152
M118T352	Amino acid and derivative	L-Threonine	C00188
M217T370	Amino acid and derivative	N-.alpha.-Acetyl-L-arginine	
M158T335	Amino acid and derivative	Acetyl-DL-Valine	
M215T371	Amino acid and derivative	N-.alpha.-Acetyl-L-arginine	
M190T189	Amino acid and derivative	N-Acetyl-DL-methionine	
M218T446	Amino acid and derivative	Ala-Lys	
M246T426	Amino acid and derivative	Arg-Ala	
M219T262	Amino acid and derivative	Ser-Ile	
M219T399	Amino acid and derivative	Ala-Glu	
M203T258_2	Amino acid and derivative	Ala-Leu	
M237T197	Amino acid and derivative	Ala-Phe	
M189T279	Amino acid and derivative	Ala-Val	
M304T443	Amino acid and derivative	Arg-Glu	
M274T384	Amino acid and derivative	Arg-Val	
M280T332	Amino acid and derivative	Asn-Phe	
M269T292	Amino acid and derivative	His-Ile	
M203T227	Amino acid and derivative	Ile-Ala	
M288T337	Amino acid and derivative	Ile-Arg	
M246T300	Amino acid and derivative	Ile-Asn	

M245T178	Amino acid and derivative	Ile-Ile	
M279T169	Amino acid and derivative	Ile-Phe	
M229T241	Amino acid and derivative	Ile-Pro	
M260T281	Amino acid and derivative	Ile-Ser	
M233T222	Amino acid and derivative	Ile-Thr	
M318T176	Amino acid and derivative	Ile-Trp	
M260T361	Amino acid and derivative	Leu-Lys	
M262T448	Amino acid and derivative	Lys-Asp	
M557T304	Amino acid and derivative	Lys-Leu	
M234T444	Amino acid and derivative	Lys-Ser	
M248T444	Amino acid and derivative	Lys-Thr	
M246T403	Amino acid and derivative	Lys-Val	
M322T313	Amino acid and derivative	Phe-Arg	
M281T332	Amino acid and derivative	Phe-Asp	
M294T254	Amino acid and derivative	Phe-Gln	
M253T238	Amino acid and derivative	Phe-Ser	
M265T176	Amino acid and derivative	Phe-Val	
M287T137	Amino acid and derivative	Prilocaine	
M187T335	Amino acid and derivative	Pro-Ala	
M272T415	Amino acid and derivative	Pro-Arg	
M245T407	Amino acid and derivative	Pro-Glu	
M177T321	Amino acid and derivative	Ser-Ala	
M303T445	Amino acid and derivative	Ser-Arg	
M235T418	Amino acid and derivative	Ser-Glu	
M207T326	Amino acid and derivative	Ser-Thr	
M269T285	Amino acid and derivative	Ser-Tyr	
M205T286	Amino acid and derivative	Ser-Val	
M191T306	Amino acid and derivative	Thr-Ala	
M249T389	Amino acid and derivative	Thr-Glu	
M233T241	Amino acid and derivative	Thr-Leu	
M248T427	Amino acid and derivative	Thr-Lys	
M267T220	Amino acid and derivative	Thr-Phe	
M221T310	Amino acid and derivative	Thr-Thr	
M386T155	Amino acid and derivative	Tyr-Gln	
M295T196	Amino acid and derivative	Tyr-Ile	
M330T255	Amino acid and derivative	Tyr-Met	
M283T95	Amino acid and derivative	Tyr-Thr	
M281T209	Amino acid and derivative	Tyr-Val	
M189T255	Amino acid and derivative	Val-Ala	
M274T363	Amino acid and derivative	Val-Arg	
M232T318	Amino acid and derivative	Val-Asn	
M246T274	Amino acid and derivative	Val-Gln	

M247T366	Amino acid and derivative	Val-Glu	
M231T192	Amino acid and derivative	Val-Ile	
M219T368	Amino acid and derivative	Val-Thr	
M298T499	Amino acid and derivative	Val-Tyr	
M217T206	Amino acid and derivative	Val-Val	
M317T32	Amino acid and derivative	Zearalenone	C09981
M93T210	Amino acid and derivative	Phenol	C00146/C15584
M403T52	Amino acid and derivative	（-）-Usnic acid	
M219T19	Amino acid and derivative	BHT	
M160T77	Carboxylic Acids	Indole-3-carboxylic acid	C19837
M133T517	Carboxylic Acids	L-Malic acid	C00149
M147T539	Carboxylic Acids	D-Pipecolinic acid	
M187T303	Carboxylic Acids	alpha-N-Acetyl-L-glutamine	
M103T258	Carboxylic Acids	Malonic acid	C00383
M121T173	Carboxylic Acids	Urocanic acid	C00785
M135T319	Carboxylic Acids	L-Threonate	C01620
M191T327	Carboxylic Acids	Quinate	C00296
M173T344	Carboxylic Acids	Shikimate	C00493
M104T374	Carboxylic Acids	4-Aminobutyric acid	C00334
M149T292	Carboxylic Acids	Dihydroxyfumarate	C00975
M173T262	Carboxylic Acids	L-Norleucine	C01933
M147T418	Carboxylic Acids	L-Pyroglutamic acid	C01879
M89T301	Carboxylic Acids	DL-lactate	C00256/C00186
M141T381	Carboxylic Acids	2-Oxoadipic acid	C00322
M173T413	Carboxylic Acids	cis-Aconitate	C00417
M191T499	Carboxylic Acids	Isocitrate	C00311
M128T289	Carboxylic Acids	L-Pyroglutamic acid	C01879
M137T386	Carboxylic Acids	Maleic acid	C01384
M117T180	Carboxylic Acids	Methylmalonic acid	C02170
M117T369	Carboxylic Acids	Succinate	C00042
M118T88	Carboxylic Acids	2-Methyl-3-hydroxybutyric acid	
M175T311	Carboxylic Acids	2-Isopropylmalic acid	C02504
M235T103	Carboxylic Acids	3-Hydroxyisovaleric acid	
M187T328	Carboxylic Acids	Azelaic acid	C08261
M147T335	Carboxylic Acids	Citramalic acid	C00815
M129T442	Carboxylic Acids	Mesaconic acid	C01732
M295T103	Carboxylic Acids	Mevalonic acid	C00418
M136T286	Carboxylic Acids	Anthranilic acid （Vitamin L1）	C00108
M162T398	Carboxylic Acids	DL-2-Aminoadipic acid	C00956
M160T397	Carboxylic Acids	DL-2-Aminoadipic acid	C00956
M149T31	Carboxylic Acids	1,2-Benzenedicarboxylic acid	
M101T371	Carboxylic Acids	DL-2,4-Diaminobutyric acid	

M176T269	Carboxylic Acids	N-Carboxyethyl-.gamma.-aminobutyric acid	
M171T268	Carboxylic Acids	3-Dehydroshikimic acid	
M114T374	Carboxylic Acids	Maleamic acid	
M383T327	Carboxylic Acids	Quinic acid	
M298T284	Glycosides	8-hydroxy Guanosine	
M282T294	Glycosides	1-Methyladenosine	C02494
M298T319	Glycosides	1-methylguanosine	
M364T428	Glycosides	3'-O-methylguanosine	
M346T312	Glycosides	3',5'-Cyclic guanosine monophosphate	C00942
M268T167	Glycosides	Adenosine	C00212
M244T238	Glycosides	Cytidine	C00475
M306T311	Glycosides	Cytidine 5'-monophosphate （CMP）	C00055
M284T260	Glycosides	Guanosine	C00387
M282T129	Glycosides	N6-methyladenosine	
M298T98	Glycosides	S-Methyl-5'-thioadenosine	C00170
M245T157	Glycosides	Uridine	C00299
M344T313	Glycosides	3',5'-Cyclic guanosine monophosphate	C00942
M282T260	Glycosides	Guanosine	C00387
M267T213	Glycosides	Inosine	C00294
M257T140	Glycosides	Ribothymidine	
M303T158	Glycosides	Uridine	C00299
M323T402	Glycosides	Uridine 5'-monophosphate （UMP）	C00105
M362T427	Glycosides	Guanosine 5'-monophosphate （GMP）	C00144
M549T388	Glycosides	Gemcitabine	
M336T68	Glycosides	Isopentenyladenosine	
M454T201	Lipids	1-Palmitoyl-2-hydroxy-sn-glycero-3-phosphoethanolamine	
M330T39	Lipids	Eicosapentaenoic Acid ethyl ester	
M748T78	Lipids	1-Palmitoyl-2-oleoyl-phosphatidylglycerol	
M355T113	Lipids	5-Oxo-ETE	
M452T201	Lipids	1-Palmitoyl-2-hydroxy-sn-glycero-3-phosphoethanolamine	
M357T261	Lipids	5（S）-HpETE	
M339T31	Lipids	Norethindrone Acetate	
M275T56	Lipids	Stearidonic Acid	
M263T342	Lipids	Linoleic acid	C01595
M255T45	Lipids	Palmitic acid	C00249
M283T44	Lipids	Stearic acid	C01530
M311T43	Lipids	Arachidic acid	C06425
M339T402	Lipids	Behenic acid	C08281
M253T37	Lipids	cis-9-Palmitoleic acid	C08362
M269T442	Lipids	Heptadecanoic acid	

M227T46	Lipids	Myristic acid	C06424
M281T101	Lipids	Oleic acid	C00712
M313T158	Lipids	1-Palmitoylglycerol	
M343T34	Lipids	20-Hydroxyarachidonic acid	
M338T2	Lipids	Erucamide	
M337T244	Lipids	MG（18:2（9Z,12Z）/0:0/0:0）[rac]	
M277T45	Lipids	all cis-（6,9,12）-Linolenic acid	C06426
M787T147	Lipids	1,2-dioleoyl-sn-glycero-3-phosphatidylcholine	
M819T136	Lipids	2-Oleoyl-1-palmitoyl-sn-glycero-3-phosphocholine（PC（16:0/18:1（9Z）））	
M152T2602	Nucleic acids and derivative	2-Hydroxyadenine	
M112T237	Nucleic acids and derivative	Cytosine	C00380
M137T213	Nucleic acids and derivative	Hypoxanthine	C00262
M113T158	Nucleic acids and derivative	Uracil	C00106
M127T372	Nucleic acids and derivative	Dihydrothymine	C00906
M113T374	Nucleic acids and derivative	Dihydrouracil	C00429
M111T159	Nucleic acids and derivative	Uracil	C00106
M282T92	Nucleic acids and derivative	2'-O-methyladenosine	C04779
M348T398	Nucleic acids and derivative	3'-O-methyladenosine	
M330T253	Nucleic acids and derivative	Adenosine 2',3'-cyclic monophosphate	C02353
M611T311	Nucleic acids and derivative	Cytidine 2',3'-cyclic phosphate	C02354
M328T256	Nucleic acids and derivative	Adenosine 3',5'-cyclic phosphate（cAMP）	C00575
M304T312	Nucleic acids and derivative	Cytidine 2',3'-cyclic phosphate	C02354
M323T423	Nucleic acids and derivative	5'-CMP	
M258T404	Nucleic acids and derivative	D-Glucosamine 1-phosphate（Glucosamine-1P）	C06156
M136T99	Nucleic acids and derivative	Adenine	C00147
M324T424	Nucleic acids and derivative	3'-O-methylcytidine	
M277T363	Other	2-C-Methyl-D-erythritol 2,4-cyclodiphosphate	
M145T364	Other	Pyruvaldehyde	C00546
M133T181	Other	Ribitol	C00474
M233T33	Other	Confertifoline	
M72T446	Other	Pyrrolidine	
M325T415	Other	3.alpha.-Mannobiose	
M391T247	Other	RU-0211	
M213T1041	Other	m-Chlorohippuric acid	
M173T72	Other	Lawsone	
M241T59	Other	Lumichrome	C01727
M327T212	Other	Coumestrol	
M146T267	Other	Oxyquinoline	

M867T79	Other	Dioscin	
M329T100	Other	Acetylvalerenolic acid	
M128T386	Other	Ammelide	
M129T259	Other	Cyanuric acid	
M301T23	Other	Diosmetin	
M147T299	Phenylpropanoids	4-Hydroxycinnamic acid	C00811
M565T212	Phenylpropanoids	Apiin	C04858
M163T54	Phenylpropanoids	4-Hydroxycinnamic acid	C00811
M563T213	Phenylpropanoids	APIIN	C04858
M166T256	Phenylpropanoids	L-Phenylalanine	C00079
M164T256	Phenylpropanoids	L-Phenylalanine	C00079
M173T181	Plant hormone	3-Methylindole	C08313
M118T165	Plant hormone	Indole	C00463
M173T182	Plant hormone	1-Methyltryptamine	C06213
M188T258	Plant hormone	DL-Indole-3-lactic acid	
M87T374	Plant hormone	4-Hydroxybutanoic acid lactone	
M323T295	Plant hormone	Beta-d-lactose	
M147T76	Plant hormone	D-Arabinono-1,4-lactone	
M220T117	Plant hormone	trans-Zeatin	C00371
M170T258	Plant hormone	Indoleacrylic acid	
M89T260	Sugar and derivative	Dihydroxyacetone	C00184
M147T180	Sugar and derivative	Hydroxyacetone	C05235
M359T2592	Sugar and derivative	D-Fructose	C00095/C05003/C10906
M191T109	Sugar and derivative	D-Galactarate	C00879
M255T261	Sugar and derivative	D-gluconate	C00257
M105T293	Sugar and derivative	Glyceric acid	C00258
M195T373	Sugar and derivative	Galactonic acid	C00880
M177T181	Sugar and derivative	D-Glucono-1,5-lactone	C00198
M237T356	Sugar and derivative	2-Dehydro-3-deoxy-D-gluconate	C00204
M757T39	Sugar and derivative	PC（16:0/16:0）	C00157
M173T334	Sugar and derivative	Glycerol 3-phosphate	C00093
M258T389	Sugar and derivative	Glycerophosphocholine	C00670
M153T261	Sugar and derivative	Glycerol 3-phosphate	C00093
M216T394	Sugar and derivative	sn-Glycerol 3-phosphoethanolamine	C01233
M759T391	Sugar and derivative	Thioetheramide-PC	
M522T189	Sugar and derivative	1-Oleoyl-sn-glycero-3-phosphocholine	
M496T196	Sugar and derivative	1-Palmitoyl-sn-glycero-3-phosphocholine	
M524T192	Sugar and derivative	1-Stearoyl-2-hydroxy-sn-glycero-3-phosphocholine	
M435T243	Sugar and derivative	1-Oleoyl-L-.alpha.-lysophosphatidic acid	
M409T251	Sugar and derivative	1-Palmitoyl Lysophosphatidic Acid	
M343T406	Sugar and derivative	Maltitol	

M259T448	Sugar and derivative	alpha-D-Galactose 1-phosphate	C00446
M259T470	Sugar and derivative	alpha-D-Glucose 1-phosphate	C00103
M297T469_2	Sugar and derivative	Beta-D-Fructose 6-phosphate	C05345
M177T105	Sugar and derivative	L-Gulonic gamma-lactone	C01040
M327T250	Sugar and derivative	D-Quinovose	C02522
M149T290	Sugar and derivative	L-Ribulose	
M383T258	Sugar and derivative	N-Acetyl-D-lactosamine	
M261T464	Sugar and derivative	alpha-D-Glucose 1-phosphate	C00103
M343T445	Sugar and derivative	D-Lactose	C00243
M505T445	Sugar and derivative	Raffinose	C00492
M667T485	Sugar and derivative	Stachyose	C01613
M360T437	Sugar and derivative	Sucrose	C00089
M179T260	Sugar and derivative	Alpha-D-Glucose	C00267
M193T311	Sugar and derivative	2'-Deoxy-D-ribose	C01801
M341T463	Sugar and derivative	Cellobiose	C00185
M341T414	Sugar and derivative	D-Lactose	C00243
M239T301	Sugar and derivative	D-Mannose	C00159
M223T255	Sugar and derivative	L-Fucose	C01019
M503T425	Sugar and derivative	Melezitose	C08243
M151T228	Sugar and derivative	Xylitol	C00379
M198T259	Sugar and derivative	D-Mannose	C00159
M243T387	Sugar and derivative	D-Mannose-6-phosphate	C00275
M360T389	Sugar and derivative	Isomaltose	C00252
M522T425	Sugar and derivative	Maltotriose	C01835
M401T389	Sugar and derivative	Galactinol	C01235
M300T432	Sugar and derivative	N-Acetylglucosamine 1-phosphate	C04256
M503T445	Sugar and derivative	Raffinose	C00492
M665T485	Sugar and derivative	Stachyose	C01613
M341T363	Sugar and derivative	Sucrose	C00089
M341T389	Sugar and derivative	Trehalose	C01083
M579T206	Sugar and derivative	Apigenin 7-O-neohesperidoside	
M149T151	Sugar and derivative	D-Lyxose	C00476
M193T182	Sugar and derivative	Perseitol	
M124T206	Vitamins	Nicotinate	C00253
M123T59	Vitamins	Nicotinamide	C00153
M168T91	Vitamins	Pyridoxal （Vitamin B6）	C00250
M265T369	Vitamins	Thiamine	C00378
M122T207	Vitamins	Nicotinate	C00253
M375T210	Vitamins	Riboflavin	C00255
M786T382	Vitamins	Flavin adenine dinucleotide （FAD）	C00016
M220T258	Vitamins	Pantothenate	C00864
M218T2612	Vitamins	Pantothenate	C00864

M170T101	Vitamins	Pyridoxine	C00314
M175T54	Vitamins	L-Ascorbic acid	C00072

Table S6. Divergent metabolites between Wb-NE and Wb-T.

Metabolism	Qst		
	0.05%	99.50%	Estimated Qst
M454T201	0.865288982	0.994603238	0.923953506
M633T37	0.8456384	0.993599597	0.910601158
M379T204	0.832029728	0.99286139	0.900926142
M251T186	0.831107127	0.992809975	0.900256821
M465T154	0.829386106	0.992713585	0.899003573
M483T154	0.826444959	0.992547261	0.896845244
M307T203	0.82639634	0.992544626	0.896811671
M481T40	0.822179295	0.992303019	0.893688241
M480T196	0.81740926	0.99202494	0.89010893
M398T203	0.812771262	0.991749545	0.886580567
M363T203	0.807569424	0.991434595	0.882565208
M447T154	0.807561003	0.991434078	0.882558632
M365T285	0.807541656	0.991432893	0.882543559
M481T153	0.80652227	0.99137038	0.881749019
M554T196	0.806514185	0.991369902	0.881743023
M522T189	0.799950499	0.990961248	0.876569674
M553T93	0.793830584	0.990570205	0.871651732
M496T196	0.791507767	0.990419162	0.869760539
M483T37	0.791007832	0.990386462	0.869351728
M311T154	0.783926746	0.989915612	0.863488457
M337T244	0.78353318	0.989889201	0.863161631
M251T55	0.782139015	0.989794685	0.861990674
M275T55	0.778151028	0.989521152	0.858611941
M452T201	0.776944268	0.989437441	0.857580893
M353T371	0.775276191	0.989320996	0.856148963
M221T111	0.774711444	0.989281395	0.855662663
M471T124	0.773572519	0.989201181	0.854678365
M328T33	0.772026336	0.989091656	0.853336533
M774T70	0.770144396	0.988957325	0.85169398
M381T263	0.768205668	0.988817752	0.849991042
M478T200	0.767992992	0.988802326	0.849802897
M876T97	0.766210992	0.988672872	0.848227333
M366T240	0.765696739	0.988635308	0.847770699
M275T33	0.764616373	0.988556104	0.846808778
M711T26_2	0.764402816	0.9885404	0.846618207

M507T156	0.76270959	0.988415357	0.845102398
M390T91	0.762139423	0.988373031	0.844589994
M417T248	0.760612734	0.988259151	0.843213017
M652T235	0.760303918	0.988236015	0.842933562
M731T348	0.75954553	0.988179067	0.842246156
M631T36	0.757837259	0.988050047	0.84069104
M618T155	0.757733807	0.988042199	0.840596548
M520T193	0.757162667	0.987998814	0.840074385
M433T246	0.75558686	0.987859458	0.838325236
M823T214	0.753276065	0.987700459	0.836493067
M215T371	0.751386283	0.987553394	0.834733882
M576T92	0.750722379	0.987501081	0.834107782
M321T98	0.748074409	0.987292429	0.831622028
M799T199	0.747598314	0.987254575	0.831171702
M532T109	0.745491904	0.987086023	0.829169628
M269T81	0.745096395	0.987054193	0.828792168
M339T27	0.744472982	0.987003883	0.828195894
M465T37	0.743834842	0.986952187	0.827583539
M753T175	0.740803619	0.986703536	0.824641658
M240T63	0.736203354	0.98632161	0.820154608
M293T117	0.733110157	0.986059036	0.817082009
M407T34	0.731817333	0.985947659	0.815780896
M339T31	0.731647232	0.985931832	0.81559205
M476T199	0.730440371	0.985795459	0.813885931
M466T169_2	0.730027521	0.98579318	0.813983308
M312T28_2	0.725389173	0.985385117	0.809251304
M284T44	0.725161727	0.985364814	0.809016484
M261T225	0.724946056	0.985345607	0.808794653
M539T153	0.724742933	0.985327463	0.808585026
M313T469	0.722477385	0.985123786	0.806235836
M411T35	0.720510925	0.984944986	0.80417939
M397T77	0.720443665	0.984938797	0.804108155
M897T93	0.718995411	0.984805889	0.802583337
M435T243	0.718735644	0.984781934	0.802308791
M311T28	0.718425759	0.984753313	0.801980909
M245T217	0.718150539	0.984727843	0.801689211
M666T23	0.717192723	0.984638933	0.800671898
M696T212	0.716789924	0.984601399	0.800242815
M512T132	0.716565028	0.984580421	0.800003144
M282T198	0.713813808	0.98432153	0.797050959
M228T301	0.712974571	0.984241754	0.796143455
M818T202	0.712205654	0.984168358	0.795309526
M285T32	0.711752582	0.984124959	0.794816837

M678T161	0.711244819	0.984076161	0.794263133
M180T83	0.709522211	0.983909683	0.792377437
M483T416	0.708344188	0.98379487	0.791079498
M450T26_1	0.708171719	0.983778004	0.790889042
M475T181	0.706640135	0.983627434	0.789190582
M431T123	0.706139872	0.983577966	0.788633373
M200T61	0.705025991	0.983467289	0.787388076
M297T28	0.704343242	0.983399126	0.786622206
M422T304	0.704099821	0.983374751	0.786348493
M821T39	0.703259464	0.983290338	0.785401328
M390T44	0.701417789	0.983103883	0.783313234
M417T34	0.700786943	0.983039532	0.782593792
M837T211	0.699396731	0.982896921	0.781001936
M291T541	0.698688682	0.982823825	0.780187244
M229T198	0.696933008	0.98264123	0.778155774
M957T34	0.696528858	0.982598925	0.77768586
M164T114	0.696502846	0.982596164	0.777655082
M445T373	0.69626256	0.982570973	0.777375467
M505T28	0.69625555	0.982570252	0.777367504
M333T246	0.695418343	0.982482136	0.776390014
M223T59	0.695363406	0.982476341	0.776325777
M217T370	0.695363307	0.982476324	0.776325562
M253T64	0.695299536	0.982469579	0.776250748
M829T78	0.694803892	0.982417182	0.775670236
M515T92	0.69475722	0.982412235	0.775615436
M545T116	0.69469785	0.982405939	0.77554568
M315T181_2	0.694621452	0.98239784	0.775455997
M322T131	0.69365321	0.982294611	0.774312859
M497T212	0.693589697	0.9822881	0.774241755
M315T35_1	0.693507218	0.982279299	0.774144463
M302T193	0.692780511	0.982201536	0.773285234
M803T55	0.692761086	0.982199459	0.773262324
M342T163	0.691088365	0.982019124	0.771273413
M497T91	0.691060391	0.982016091	0.771240004
M291T273	0.690938528	0.982002404	0.771087689
M366T34_1	0.689603757	0.981857466	0.769494659
M597T34	0.688886438	0.981778839	0.768631018
M795T199	0.688790173	0.981768264	0.768514944
M225T321	0.688096883	0.981691871	0.767676805
M702T34	0.687286361	0.981602103	0.766692962
M629T37_2	0.686735946	0.981540936	0.766023466
M735T214	0.686693895	0.981536229	0.765971891
M253T52	0.686041625	0.981463423	0.76517571

M431T250	0.685294875	0.981379669	0.764260715
M668T22	0.68430651	0.981268167	0.763044096
M879T80	0.683387123	0.981163861	0.761907858
M409T33	0.682107778	0.981017609	0.760317197
M509T157	0.682107706	0.981017562	0.760316572
M180T110	0.682085304	0.98101505	0.760289472
M646T82	0.681123197	0.980904251	0.759086585
M205T59	0.680823099	0.980869546	0.758710192
M269T56	0.680628805	0.98084704	0.758466187
M275T219	0.68029782	0.980808635	0.758049993
M794T71	0.679404876	0.980704597	0.756923604
M220T26	0.679324182	0.980695159	0.756821479
M581T36	0.679307393	0.980692744	0.756793901
M187T328	0.67903194	0.980660876	0.756450437
M1023T172	0.678361442	0.980582236	0.755600849
M462T243	0.677609056	0.980305424	0.752019393
M860T49	0.677495225	0.980480008	0.754497421
M267T259	0.677343534	0.980462022	0.754303371
M675T214_2	0.676573942	0.980370305	0.753313989
M294T294	0.676349607	0.980343877	0.753030376
M1025T78	0.675545639	0.980247709	0.751995598
M604T209	0.675476703	0.980239435	0.751906628
M276T319	0.675189749	0.980204961	0.75153604
M417T28	0.673179282	0.979961541	0.748924228
M476T317	0.672175893	0.979838776	0.747610185
M981T81	0.672160848	0.979836928	0.747590425
M516T187	0.672003471	0.979817594	0.747383673
M535T165	0.671672468	0.979776851	0.746948148
M282T107	0.671297525	0.979730597	0.746454026
M433T34_2	0.67083297	0.97967311	0.745840297
M329T318	0.669463754	0.979502571	0.744022368
M687T34	0.669458997	0.979501975	0.744016015
M353T38	0.667795596	0.97929253	0.741788897
M247T269	0.667600495	0.979267793	0.74152625
M515T213	0.66747616	0.979252018	0.74135881
M199T260	0.666939079	0.9791837	0.740634067
M395T208	0.666601547	0.979140621	0.740177367
M289T234	0.665914475	0.979052604	0.739245066
M399T259	0.665258854	0.978968021	0.738349589
M303T51	0.664726427	0.978899389	0.737624783
M398T77	0.66435553	0.978851278	0.737116642
M273T262	0.664113835	0.978819854	0.736784916
M408T76	0.663899243	0.978791906	0.736489997

M532T92	0.663085744	0.978685527	0.735368332
M847T101	0.662990728	0.978673083	0.735237288
M915T126	0.662620777	0.978624477	0.734725424
M538T108	0.662565632	0.978617219	0.734649019
M277T108	0.661880692	0.978526817	0.733697914
M474T375	0.661624051	0.978492825	0.733340575
M952T33	0.661539668	0.978481405	0.733219873
M199T91	0.660294903	0.978315661	0.731480472
M773T200	0.660070647	0.978285652	0.731165992
M296T53	0.659333846	0.978186523	0.730127502
M689T191	0.659275368	0.978178611	0.73004461
M270T32	0.659207999	0.978169535	0.729949664
M242T106	0.658999499	0.978141355	0.729654749
M237T59_2	0.657306445	0.977910826	0.727246072
M339T337	0.657055839	0.977876442	0.726887414
M173T423	0.657009571	0.977870089	0.726821159
M323T40	0.656884064	0.977852836	0.726641261
M407T246	0.656727919	0.977831349	0.72641727
M195T59	0.656633879	0.977818393	0.726282233
M375T28	0.65654424	0.977806042	0.72615354
M253T129	0.655598264	0.977675119	0.724790487
M308T88	0.655440603	0.977653202	0.724562513
M347T61	0.654446979	0.977514416	0.723120333
M543T109	0.654273305	0.977488933	0.722852597
M537T93	0.653863474	0.977432442	0.722269786
M375T155	0.653496069	0.977379585	0.721718829
M153T371	0.652987275	0.977308575	0.72098609
M343T301	0.651974473	0.977164301	0.719493369
M222T204	0.651795279	0.977138649	0.719228243
M845T80	0.651428939	0.977086056	0.718684825
M497T188	0.650705414	0.976981673	0.7176072
M361T141	0.650637759	0.976972037	0.717508235
M435T116	0.650386727	0.976935619	0.717132542
M185T386	0.650330528	0.976927544	0.717049501
M348T230	0.650288541	0.976921454	0.716986725
M1128T144	0.650256005	0.976916736	0.716938112
M507T160	0.649954609	0.976872947	0.716486926
M381T205	0.649483894	0.976804348	0.715780624
M118T27	0.649425456	0.976795812	0.71569278
M469T202	0.648836818	0.9767096	0.714806046
M62T13	0.648337984	0.976636203	0.714051843
M847T131	0.648228566	0.976620062	0.713886097
M273T242	0.647679622	0.976538858	0.713052608

M433T250	0.647130038	0.976457168	0.712214933
M974T34	0.646826286	0.976411868	0.711750812
M731T26	0.646157616	0.976311707	0.710725448
M226T114	0.646026502	0.976291998	0.710523821
M409T251	0.645860938	0.976267075	0.710268908
M391T35	0.645256004	0.976175731	0.709335389
M553T230	0.644884626	0.976119412	0.708760319
M368T44	0.644182742	0.976012466	0.707669345
M1029T400_2	0.643907704	0.975970379	0.707240391
M310T249	0.643888686	0.975967465	0.707210697
M801T596	0.643669981	0.975933821	0.706867684
M557T304	0.64364859	0.975930635	0.706835506
M1001T80	0.64361571	0.975925584	0.706784065
M235T263_2	0.643502141	0.975908127	0.706606285
M309T58	0.643411851	0.975894241	0.706464918
M1162T423_1	0.643355252	0.97588553	0.706376242
M251T585	0.643335846	0.975882515	0.706345485
M485T242	0.643294345	0.97587615	0.706280768
M357T35	0.643200435	0.975861667	0.706133343
M853T87	0.643163057	0.975855912	0.706074802
M551T314	0.642996524	0.97583021	0.705813298
M237T44	0.64285588	0.975808473	0.7055922
M452T123	0.642697877	0.975784018	0.70534353
M402T230	0.642484764	0.975750959	0.705007404
M408T33	0.642456066	0.975746502	0.704962112
M281T254	0.641789268	0.975642726	0.703908177
M505T203	0.641762066	0.975638479	0.703865065
M163T111	0.64138706	0.975579801	0.703269668
M98T106	0.64123772	0.975556392	0.703032285
M284T114	0.640842676	0.975494178	0.702401366
M297T35	0.640835749	0.975493192	0.702391652
M447T99	0.640786381	0.975485416	0.702312869
M112T329	0.640551661	0.975448394	0.701937866
M496T107	0.64037038	0.975419745	0.701647767
M647T36	0.640282345	0.975405813	0.701506731
M179T59	0.640056589	0.97537004	0.701144702
M598T346	0.639922985	0.975348554	0.70092658
M142T242	0.639906865	0.975346271	0.700904234
M323T199	0.639729729	0.975318107	0.700619381
M464T308	0.639367939	0.975260442	0.700036454
M373T395	0.6393266	0.975253849	0.699969852
M97T111	0.638736433	0.975159337	0.699015368
M588T114	0.63869417	0.975152549	0.698946852

M215T259	0.638529154	0.975126022	0.698679175
M465T289	0.638504738	0.97512209	0.698639493
M922T422_1	0.638380603	0.975102091	0.698437713
M593T392	0.638226517	0.975077256	0.698187263
M460T317	0.638153716	0.975065488	0.698068562
M381T53	0.638090121	0.975055233	0.697965217
M722T26	0.637969019	0.975035659	0.697767899
M339T138	0.637451213	0.974951686	0.696921888
M678T500	0.637260487	0.974920657	0.696609497
M697T199	0.636843063	0.974852566	0.695924406
M476T119	0.636587191	0.974810685	0.695503256
M459T26	0.636487458	0.974794247	0.695337801
M157T93	0.636486512	0.974794177	0.695337319
M321T294	0.636338307	0.974769841	0.69509272
M262T112	0.63596514	0.974708437	0.69447593
M294T105	0.635963083	0.974708098	0.694472526
M634T346	0.635835486	0.974687049	0.694261176
M362T104	0.635811581	0.974683103	0.694221573
M397T223	0.635565084	0.974642157	0.693810106
M466T124	0.635494447	0.974630644	0.693695062
M437T299	0.635288957	0.974596593	0.69335354
M945T465_1	0.635101443	0.974565417	0.693040851
M799T53	0.635007088	0.974549676	0.692882931
M374T115	0.63478355	0.974512518	0.69251076
M221T61	0.634654409	0.974490967	0.692294857
M618T34	0.634579816	0.974478507	0.692170049
M1008T80	0.634326419	0.974436112	0.691745546
M743T342	0.633977522	0.974377567	0.691159659
M587T32	0.63385187	0.974356433	0.690948257
M304T108	0.633815433	0.974350299	0.6908869
M295T176	0.63381317	0.97434986	0.690882362
M434T84	0.633696443	0.974330244	0.690686322
M150T225	0.633468475	0.974291785	0.69030189
M293T274	0.633435377	0.974286189	0.690245951
M409T34_1	0.633428565	0.974285005	0.690234029
M174T264	0.632783076	0.974175599	0.689141413
M150T119	0.632090753	0.974057326	0.687961373
M449T358	0.632067968	0.97405352	0.687923729
M455T443	0.631939246	0.974033879	0.687734119
M451T115	0.631784431	0.974004875	0.687438919
M1003T80	0.631710082	0.973992095	0.687311607
M390T56	0.631694515	0.973989418	0.68728494
M707T241	0.631329018	0.97392642	0.686657562

M188T97	0.631260086	0.973914536	0.686539325
M323T84_1	0.631096769	0.973886307	0.686258436
M273T33	0.631090522	0.973885216	0.686247554
M381T23	0.63103837	0.973876174	0.686157552
M284T83	0.630958572	0.973862379	0.686020387
M279T506	0.63079141	0.97383339	0.685732078
M255T262	0.630400901	0.973765469	0.685056917
M321T103	0.630117699	0.973716049	0.684566005
M392T44	0.630004557	0.973696262	0.684369509
M410T274	0.62994184	0.973685284	0.684260522
M697T212	0.629936345	0.973684319	0.684250938
M372T336	0.629488337	0.973605689	0.683470661
M921T52	0.629288578	0.973570496	0.683121607
M549T91	0.628763008	0.973477429	0.682198894
M912T34_2	0.628622866	0.973452738	0.681954789
M206T375	0.628550449	0.97343985	0.681827107
M540T114	0.628487247	0.973428646	0.68171625
M633T247	0.628397775	0.973412731	0.681558701
M603T26	0.628115657	0.973362453	0.681061167
M131T257	0.627988499	0.973339738	0.68083647
M541T35	0.627891631	0.973322415	0.680665154
M220T56	0.627700759	0.973288234	0.680327211
M440T114	0.627604664	0.973270996	0.680156837
M459T24	0.627469673	0.973246754	0.679917279
M100T57	0.627388499	0.973232159	0.679773085
M310T431	0.627259809	0.973208983	0.679544128
M841T56	0.627177302	0.973194126	0.679397429
M662T174	0.62697101	0.97315689	0.679029792
M209T333	0.626800066	0.973124549	0.678707087
M603T201	0.626571343	0.973084491	0.678315346
M724T36	0.62631535	0.973037999	0.677856963
M542T111	0.626225465	0.973021633	0.677695633
M423T232	0.626223469	0.973021262	0.677691947
M963T39	0.62593712	0.97296838	0.67716929
M395T222	0.625659496	0.972918215	0.676676812
M133T324	0.625635984	0.972913905	0.676634381
M467T169	0.625269433	0.972846533	0.67597123
M569T101	0.625004636	0.972797452	0.67548783
M364T110	0.624932838	0.972784492	0.675361129
M279T84	0.624830704	0.972765583	0.675175166
M375T98	0.624339875	0.972674566	0.674280945
M707T118	0.624128201	0.972635111	0.673893474
M1004T170	0.624051709	0.972620879	0.673753852

M318T81	0.624020829	0.972615115	0.673697283
M816T32	0.623686246	0.972552526	0.673083164
M348T116	0.622958771	0.972415646	0.671741589
M293T301	0.622790329	0.972383812	0.671429903
M163T55	0.622597325	0.972347237	0.67107187
M553T33	0.622417436	0.972313072	0.670737564
M396T101	0.622369814	0.972304005	0.670648824
M496T319	0.62232194	0.972294907	0.670559848
M301T114	0.622189423	0.972269662	0.670312931
M258T107	0.622132827	0.972258844	0.670207082
M109T111	0.621896381	0.972213714	0.669765975
M550T36_2	0.621843446	0.972203588	0.669667005
M639T32	0.621693691	0.972174902	0.66938672
M192T505	0.621276298	0.972094683	0.668603315
M207T304	0.621143355	0.972069037	0.668352976
M244T114	0.621134514	0.972067346	0.668336513
M227T318	0.621077378	0.97205636	0.668229406
M336T170	0.620998388	0.972041057	0.668079992
M161T82	0.620922453	0.972026373	0.667936744
M449T26	0.620908623	0.972023586	0.667909292
M195T266	0.62066206	0.971975919	0.667444699
M183T85	0.620352081	0.97191388	0.666835847
M482T243	0.620239755	0.971893763	0.666644039
M188T83	0.620216691	0.971889225	0.666599735
M251T139	0.619805403	0.971808777	0.665816374
M615T92	0.619724102	0.971792848	0.665661395
M242T59	0.619590717	0.971766685	0.665406906
M223T87	0.61945685	0.97174035	0.665150722
M595T148	0.619311748	0.971711752	0.664872596
M705T181	0.61919891	0.971689489	0.664656151
M867T102	0.619121276	0.97167353	0.664499578
M446T243	0.618889245	0.971628215	0.664060667
M142T113	0.618854956	0.971621416	0.663994605
M163T34_2	0.61881405	0.971613291	0.663915654
M471T34_2	0.618757396	0.971601909	0.663804785
M306T83_1	0.618738617	0.971598325	0.663770312
M255T271	0.618662812	0.971583261	0.663624004
M285T219	0.618461434	0.971543174	0.663234781
M359T114	0.618212594	0.971493516	0.662752883
M699T214	0.617952292	0.971440872	0.662241036
M321T320	0.617332781	0.971316617	0.661037921
M306T114	0.617322756	0.971314654	0.661019062
M185T37	0.617308829	0.971311755	0.660990785

M259T104	0.617275725	0.971305119	0.660926662
M841T127	0.617269494	0.971303886	0.660914779
M373T336_1	0.617218678	0.971293603	0.660815201
M205T39	0.617099526	0.97126947	0.66058155
M145T112	0.617000576	0.9712494	0.660387278
M731T34	0.616935815	0.971236232	0.660259781
M576T40	0.616543364	0.971156337	0.659486942
M274T169	0.616089974	0.971063528	0.658589911
M219T263	0.616089169	0.971063359	0.658588268
M223T39	0.616083414	0.971062181	0.658576891
M437T33	0.616065241	0.971058446	0.658540799
M927T422_1	0.615859548	0.971016145	0.658132239
M349T216	0.615362173	0.9709134	0.65714056
M1052T173	0.615296653	0.970899818	0.657009552
M970T24	0.615151344	0.970869648	0.656718591
M557T169	0.614814991	0.970799594	0.656043292
M87T116	0.614771821	0.97079058	0.655956441
M917T173	0.614755185	0.970787094	0.655922825
M595T217	0.614069373	0.97064319	0.654537283
M452T115	0.613950177	0.970618048	0.654295401
M557T114	0.613844232	0.97059564	0.654079819
M260T105	0.613783151	0.970582739	0.653955795
M141T116	0.61363129	0.970548862	0.653626274
M656T348	0.613443617	0.970510522	0.653261136
M114T168	0.613430556	0.970507954	0.65323692
M224T62	0.613398662	0.970501063	0.653170457
M418T246	0.612910584	0.970396721	0.652167957
M153T27	0.612805029	0.970374395	0.651954328
M341T34	0.612587765	0.970325497	0.65148018
M222T60	0.612550671	0.970319761	0.651430132
M451T34	0.612513275	0.970311477	0.651350152
M263T115	0.612485155	0.970305659	0.651294877
M293T119	0.612459027	0.970299502	0.651234659
M645T34	0.612423015	0.97029227	0.651166467
M372T105	0.612139936	0.970231135	0.650580368
M694T213	0.612049435	0.970210579	0.65038127
M563T185	0.611946234	0.970189163	0.650178188
M991T170	0.611943612	0.970188593	0.65017272
M293T168	0.611918963	0.970183243	0.650121471
M283T44	0.611876819	0.970174092	0.650033803
M379T25	0.611850476	0.970168373	0.649979032
M275T581	0.611795495	0.970156424	0.64986458
M756T348	0.611704499	0.970136627	0.649674978

M497T40	0.611661434	0.970127245	0.649585153
M263T34_2	0.61157446	0.970108282	0.649403587
M732T35	0.611482748	0.970088278	0.649212133
M370T231	0.611418202	0.970074172	0.649077119
M711T214	0.611360968	0.970061654	0.64895732
M1046T58	0.611311253	0.970050781	0.648853292
M258T168	0.611162756	0.97001817	0.648541164
M382T53	0.611043937	0.969992154	0.648292509
M581T254	0.611028483	0.969988761	0.648260066
M636T174	0.610928891	0.969966853	0.648050584
M378T24	0.610914664	0.969963685	0.648020221
M313T33	0.610891901	0.969958708	0.647972716
M379T453	0.610746286	0.969926614	0.647665956
M555T107	0.610639845	0.96990311	0.64744135
M535T66	0.61054494	0.969882116	0.64724076
M130T50	0.610490359	0.96986882	0.647111131
M183T279	0.61044389	0.969859726	0.647026871
M296T83	0.610397899	0.969849549	0.646929717
M146T676	0.610378484	0.969845243	0.646888592
M1036T185	0.610326569	0.969833718	0.646778526
M717T151	0.610241632	0.969814856	0.646598436
M259T106	0.610219454	0.969809924	0.64655135
M904T127	0.61012462	0.969788821	0.646349904
M353T115	0.609858566	0.969729482	0.645783693
M206T183	0.609848513	0.969727224	0.645762129
M313T28_1	0.609753785	0.969705318	0.645551626
M298T273	0.609504343	0.969650112	0.645026832
M980T168_1	0.609495024	0.96964802	0.645006861
M563T317	0.609431373	0.969633661	0.644869879
M736T26_2	0.609429792	0.969633356	0.644867086
M781T345	0.608991732	0.969534525	0.643925533
M321T363	0.608973481	0.969530389	0.643886134
M277T353	0.608696212	0.969467491	0.643287381
M449T60	0.608624325	0.969451139	0.643131775
M901T127	0.608571178	0.969438792	0.643013765
M1016T168	0.607922891	0.969290667	0.641605899
M422T242	0.607820724	0.96926714	0.641382353
M279T113	0.607639649	0.969225388	0.640985814
M255T24	0.607493841	0.969191632	0.64066522
M401T33	0.607391425	0.969167946	0.640440473
M1051T157	0.607213798	0.969126705	0.640049112
M282T143	0.607202869	0.969124163	0.640024985
M441T281	0.607179804	0.969118787	0.639973952

M77T230	0.607175866	0.969117882	0.639965396
M320T83	0.607027351	0.969083296	0.639637321
M179T169	0.606738272	0.969015743	0.638996818
M298T24	0.60639357	0.968934795	0.638229815
M429T55	0.606331508	0.968920144	0.638090982
M663T220	0.606238733	0.968897838	0.637878871
M396T581	0.606218119	0.968893176	0.637835159
M119T114	0.606171832	0.968882495	0.637734549
M393T251	0.606142765	0.968875624	0.637669495
M1158T422_1	0.606042788	0.968851254	0.637437331
M197T26_2	0.606002068	0.968842327	0.637354323
M313T314	0.605915945	0.968820282	0.637142342
M365T34	0.605830958	0.968801732	0.636970179
M269T39	0.605762504	0.96878546	0.63681624
M138T25	0.605752742	0.968783137	0.636794267
M318T108	0.605646344	0.968757816	0.636554793
M346T250	0.60556226	0.968737575	0.63636298
M264T388	0.605531035	0.96873031	0.636294675
M486T37	0.605516508	0.968726842	0.636261889
M675T144	0.605292171	0.968673182	0.635754654
M657T32	0.605212701	0.968653873	0.635571678
M298T167	0.605194931	0.96864986	0.635534274
M402T346	0.605115317	0.968630742	0.635353644
M391T247	0.6050085	0.968605047	0.635110939
M276T168	0.604991824	0.968601035	0.635073053
M455T318	0.604892875	0.96857719	0.634847867
M334T169	0.60480715	0.968556465	0.634652114
M269T124	0.604542561	0.968492457	0.634048053
M239T122	0.604517102	0.968486269	0.633989632
M665T220	0.60449682	0.968480558	0.633934146
M1036T172	0.604457643	0.968471846	0.633853582
M181T112	0.604268839	0.968425908	0.633420268
M171T77	0.604175199	0.968403038	0.633204551
M498T127	0.604159669	0.968399284	0.633169233
M158T26	0.604147189	0.968396246	0.633140599
M441T242	0.604134874	0.968393235	0.633112205
M314T243	0.603841749	0.968321477	0.632435885
M737T70	0.603745501	0.96829784	0.632213193
M210T334	0.603719013	0.968291326	0.63215183
M293T82	0.603617043	0.968266231	0.631915472
M357T151	0.603591925	0.968260043	0.631857183
M1021T185	0.603400557	0.968211991	0.631403128
M442T168	0.60335702	0.968202039	0.63131108

M179T114	0.60325616	0.968177064	0.631076022
M394T316	0.602911542	0.968091401	0.630270153
M583T299	0.60289286	0.968086749	0.630226412
M209T316	0.602846602	0.968075206	0.630117869
M538T33	0.602795901	0.968062541	0.629998774
M310T169	0.602783741	0.968059512	0.629970314
M140T90	0.602782958	0.968059308	0.629968381
M152T230	0.6027031	0.968039348	0.629780758
M250T225	0.602616012	0.968017509	0.629575421
M496T214	0.602565624	0.968004234	0.629449383
M494T169	0.602301722	0.967938514	0.628833191
M654T33	0.602260992	0.967928303	0.628737386
M521T115	0.602239553	0.967922908	0.628686752
M345T180	0.601860655	0.967827013	0.627786526
M297T25	0.601663581	0.967776877	0.627316143
M82T26	0.601623738	0.967766722	0.627220892
M605T32	0.601446484	0.96772148	0.626796686
M641T57	0.601399805	0.967709538	0.626684735
M690T111	0.601222458	0.967664071	0.626258582
M310T65	0.601002641	0.967606423	0.625716351
M521T83	0.600835626	0.967564385	0.62532477
M346T222	0.600573853	0.967496529	0.624689552
M283T24	0.600557175	0.967492192	0.624648964
M435T98	0.600471809	0.967469981	0.624441118
M333T35	0.60043087	0.967459324	0.624341414
M303T83	0.600168383	0.967390778	0.623700281
M401T231	0.600115606	0.967376949	0.623570971
M239T257	0.600098159	0.96737238	0.623528261
M645T81	0.60007422	0.967366051	0.62346899
M343T34	0.600065369	0.967362916	0.623438121
M146T267	0.600052088	0.967360298	0.623415305
M486T106	0.59992101	0.967325869	0.623093481
M161T109	0.599837596	0.967303911	0.62288828
M992T170	0.599533706	0.967223609	0.622138092
M194T150	0.599459961	0.967204012	0.621955011
M505T159	0.599318275	0.967166397	0.621603887
M401T47_1	0.599182122	0.967130183	0.621265991
M605T26	0.598999022	0.967081158	0.620808394
M683T203	0.598939633	0.967065254	0.620660042
M600T346	0.598857929	0.967043451	0.620456905
M424T242	0.598633102	0.96698298	0.6198931
M790T52	0.598575035	0.966967219	0.619746009
M407T190	0.598525846	0.96695404	0.619623375

M436T320	0.598483549	0.966942608	0.619516837
M626T166	0.598308956	0.966895334	0.619076407
M148T370	0.598308259	0.966776155	0.6177434
M683T200	0.598249211	0.966879113	0.618925315
M525T51	0.598219115	0.966870443	0.618843638
M303T114	0.598164388	0.966856064	0.61871067
M390T36	0.598066121	0.966829303	0.618461488
M117T242	0.598017754	0.966816032	0.618337784
M393T350	0.59799414	0.966807992	0.618259955
M480T322	0.597909909	0.966786352	0.61806109
M353T213	0.597713792	0.966732929	0.61756453
M320T114	0.597703619	0.96673014	0.617538596
M616T34	0.597693196	0.966727262	0.617511791
M575T26	0.597639081	0.966691969	0.617145497
M203T35_1	0.597569675	0.966693322	0.617196119
M117T114	0.597523109	0.966680497	0.617076835
M795T393	0.597353693	0.966632898	0.616632701
M415T34_2	0.597343914	0.966630283	0.61660857
M447T323	0.597322686	0.966625152	0.616562224
M326T249	0.597315789	0.966623251	0.616544566
M198T151	0.59730617	0.966620591	0.616519845
M223T101	0.597273893	0.966611574	0.616435881
M442T35	0.597172212	0.966583465	0.616174781
M440T169	0.597150495	0.966577429	0.616118667
M205T401	0.597093233	0.96656153	0.615970949
M629T37_1	0.596983079	0.966530877	0.615686166
M423T212	0.596747591	0.966464773	0.61507161
M849T33_1	0.596592503	0.966421622	0.614671632
M801T130	0.596380172	0.966361807	0.614116462
M982T171	0.596321847	0.966345391	0.613964245
M178T286	0.596240536	0.966321338	0.613739186
M677T214	0.596194363	0.966308627	0.613621948
M1020T24	0.596124632	0.96628957	0.613446504
M831T88	0.596064197	0.966271994	0.613282753
M517T105	0.596009519	0.966256886	0.613143465
M514T467	0.595747226	0.966182063	0.612449904
M524T192	0.595671168	0.966160334	0.61224864
M380T26	0.595582188	0.966134751	0.612011513
M547T35	0.595446128	0.966095682	0.611649718
M147T181	0.595433308	0.966091954	0.611615139
M317T301	0.595292107	0.966051293	0.611238755
M960T42	0.595234486	0.966027548	0.611006241
M409T17	0.59515969	0.966010659	0.610858457

M370T319	0.595106751	0.965997694	0.610742728
M330T332	0.595099434	0.965995525	0.610722567
M343T183	0.595044385	0.96597959	0.610575186
M1157T423_2	0.595013555	0.965970143	0.610486908
M215T289	0.594976612	0.965959904	0.610393059
M1160T59	0.594928401	0.965945876	0.61026329
M359T278	0.594545331	0.965833816	0.609226956
M144T317_2	0.594392011	0.965788795	0.608811003
M532T179	0.594178666	0.965724682	0.60821675
M587T414	0.594051992	0.96568824	0.607882162
M380T77	0.594035871	0.965683452	0.607837956
M325T61	0.593827924	0.965621518	0.60726615
M1077T71	0.593766464	0.965603003	0.607094995
M543T60	0.593682195	0.965577924	0.606863804
M114T318	0.593651409	0.965568752	0.606779263
M420T274	0.593619705	0.965559243	0.606691517
M460T85	0.593614043	0.96555755	0.606675906
M193T34_2	0.593532435	0.965533048	0.606449874
M177T33	0.593462655	0.965512022	0.606255888
M279T262	0.593456484	0.965509996	0.60623689
M296T114	0.593451485	0.965508649	0.606224744
M189T59	0.593443363	0.965506254	0.606202745
M207T59	0.593392734	0.96549081	0.606059984
M922T422_2	0.593312805	0.965466195	0.605832158
M421T212	0.593284937	0.965458456	0.605761992
M789T100	0.593088431	0.965398922	0.605213217
M353T92	0.593045447	0.965385838	0.6050926
M728T26_1	0.592908938	0.965344295	0.604709865
M291T68	0.592870016	0.965332417	0.604600444
M330T255	0.592732563	0.965290387	0.604213321
M679T213	0.592580399	0.96524369	0.603783332
M586T203	0.592552015	0.9652338	0.603690292
M222T286	0.592466079	0.965206941	0.603442384
M321T215	0.592445623	0.96520123	0.60339085
M680T213	0.592229066	0.965135171	0.602784525
M1050T352	0.592127958	0.965103824	0.602496244
M572T319	0.592034662	0.965074783	0.60222914
M527T349	0.591935718	0.965043923	0.601945376
M117T413	0.591791075	0.964998646	0.601529116
M127T113	0.591721768	0.964976902	0.601329272
M819T136	0.591717698	0.964975537	0.601316576
M462T255	0.591661497	0.964957956	0.601155149
M772T216	0.591637983	0.964949776	0.601078671

M137T328	0.59157394	0.96492912	0.600888054
M439T408	0.591568415	0.964928644	0.600885813
M293T328	0.591491376	0.96490426	0.600661669
M266T145	0.591470209	0.964897599	0.600600529
M404T246	0.591175892	0.964804246	0.599743287
M463T211	0.591146038	0.964794741	0.599656046
M922T422_4	0.59102059	0.964754634	0.599287833
M513T26_2	0.590998606	0.964747618	0.599223448
M404T114_1	0.59099449	0.964745994	0.599208029
M923T422_2	0.590993034	0.964745867	0.599207431
M224T671	0.590991294	0.964745316	0.599202381
M689T103	0.590962877	0.964736213	0.599118836
M315T119	0.59092035	0.96472259	0.598993835
M1156T425	0.590835181	0.964695248	0.598742971
M432T79	0.590797929	0.964683273	0.59863311
M659T212	0.590738385	0.96466409	0.598457112
M169T35_2	0.59058424	0.964614336	0.598000819
M267T286	0.590502532	0.964587875	0.597758212
M1009T79	0.590332216	0.964532525	0.597250808
M531T230	0.590232476	0.964499991	0.596952651
M477T268	0.590170606	0.964479769	0.596767363
M289T399	0.590043885	0.964438121	0.596385615
M385T215_2	0.589895275	0.96438935	0.595939121
M193T314	0.589860751	0.964377962	0.595834843
M537T32	0.589577789	0.964283734	0.594971442
M137T213	0.589417192	0.964230696	0.594486934
M550T92	0.589358918	0.964211046	0.594306907
M519T110	0.589340036	0.96420489	0.594250842
M458T26_1	0.589198902	0.964157536	0.593817736
M373T432	0.58919458	0.964156085	0.593804469
M675T214_1	0.589160436	0.964144599	0.593699429
M513T92	0.589110972	0.96412791	0.593546777
M208T108	0.589081549	0.964118001	0.593456199
M1153T422_1	0.58904839	0.964106581	0.593351417
M255T210	0.58902791	0.964099891	0.593290618
M557T337	0.588982814	0.964084659	0.593151394
M225T75	0.588975659	0.964082245	0.593129341
M347T48	0.588762661	0.964009951	0.592468628
M293T113	0.588760673	0.96400925	0.592462176
M609T351	0.588756235	0.96400776	0.592448599
M516T346	0.588642321	0.963968908	0.59209365
M519T38	0.588514793	0.963925249	0.591694856
M558T348	0.58841421	0.963890703	0.5913794

M351T358	0.588231687	0.963827737	0.590804521
M407T62	0.588211207	0.96382064	0.590739729
M231T323	0.588181899	0.963810496	0.590647154
M167T59	0.588159564	0.963802738	0.590576312
M638T313	0.588155321	0.963801212	0.590562302
M459T203	0.5879864	0.963741325	0.59001401
M158T179	0.587844938	0.963693162	0.589576573
M358T38	0.587807086	0.96353278	0.587887279
M263T231	0.587804042	0.963678837	0.589445912
M415T104	0.58775828	0.963662787	0.589299531
M85T111	0.587741194	0.963656647	0.589243318
M1049T82	0.587695205	0.963640624	0.589097415
M689T242	0.587690014	0.963638803	0.589080819
M261T135	0.587672922	0.963632791	0.589025997
M923T422_1	0.587655783	0.963626747	0.588970868
M360T259_1	0.587614585	0.963612239	0.588838611
M134T250	0.587430444	0.963546839	0.58824198
M260T246	0.587428105	0.963546303	0.588237551
M131T113	0.587328635	0.963508252	0.587886626
M204T113	0.587293016	0.963498293	0.587800045
M1008T180	0.587188823	0.963461113	0.587461279
M215T328	0.587135264	0.963439199	0.587257458
M243T325	0.587012136	0.963411814	0.587012136
M228T168	0.586658261	0.963392622	0.586658261
M260T82	0.586468535	0.963352122	0.586468535
M848T131	0.586453471	0.96335046	0.586453471
M423T123	0.58642738	0.963347713	0.58642738
M174T225	0.586328398	0.963336717	0.586328398
M680T175	0.586111556	0.963312896	0.586111556
M761T99	0.586052981	0.963306571	0.586052981
M142T23	0.586044255	0.963305505	0.586044255
M362T36	0.584249549	0.963108244	0.584249549
M964T31_2	0.584160628	0.963098785	0.584160628
M460T316	0.58361971	0.96303897	0.58361971
M347T59	0.583363826	0.963010822	0.583363826
M447T210	0.583245179	0.962997766	0.583245179
M917T175	0.582629702	0.962930065	0.582629702
M468T169	0.582301427	0.962893903	0.582301427
M553T81	0.581668442	0.962824211	0.581668442
M181T53_2	0.581005857	0.962751238	0.581005857
M323T423	0.580894848	0.96273925	0.580894848
M523T50	0.580617892	0.962708496	0.580617892
M191T585	0.580508754	0.962696484	0.580508754

M381T257	0.580428329	0.962687612	0.580428329
M772T54	0.580071248	0.962648261	0.580071248
M488T28	0.580009677	0.962641476	0.580009677
M1060T178	0.579781394	0.962616315	0.579781394
M1035T185	0.579719458	0.962609489	0.579719458
M857T70	0.579653831	0.962602253	0.579653831
M264T311	0.579619506	0.962598481	0.579619506
M942T448_1	0.579304985	0.962563987	0.579304985
M555T351	0.579161669	0.962548	0.579161669
M1078T34	0.579149514	0.962546655	0.579149514
M166T319	0.579115944	0.962542955	0.579115944
M758T200_1	0.578922593	0.962521633	0.578922593
M136T286	0.578781034	0.962506023	0.578781034
M209T53	0.578656613	0.962492309	0.578656613
M407T176	0.578337093	0.962457072	0.578337093
M206T285	0.578128228	0.962434027	0.578128228
M357T412	0.578102432	0.962431175	0.578102432
M282T26_2	0.577924719	0.96241157	0.577924719
M641T32	0.577843374	0.962402597	0.577843374
M431T137	0.577485944	0.962363159	0.577485944
M430T345	0.577036817	0.962313593	0.577036817
M1000T35	0.577036805	0.962313592	0.577036805
M327T392	0.576748703	0.962281805	0.576748703
M833T89	0.576735523	0.962280337	0.576735523
M240T233	0.576370114	0.962239998	0.576370114
M577T310	0.57607331	0.962207224	0.57607331
M354T430	0.575837072	0.962181138	0.575837072
M227T34	0.575699658	0.962165988	0.575699658
M698T210	0.575670301	0.962162721	0.575670301
M255T45	0.575375812	0.962130195	0.575375812
M432T204	0.574950836	0.962083653	0.574950836
M111T318	0.57462939	0.962047733	0.57462939
M757T112	0.573695429	0.961944515	0.573695429
M1140T37	0.573656813	0.961940434	0.573656813
M221T235	0.572873763	0.961853673	0.572873763
M468T345	0.572840269	0.961849981	0.572840269
M386T39	0.572817814	0.961847712	0.572817814
M922T422_3	0.572513501	0.961813845	0.572513501
M743T364	0.572427797	0.961804387	0.572427797
M964T167	0.572009945	0.961758138	0.572009945
M769T169	0.571878143	0.961743555	0.571878143
M243T56	0.571653036	0.961718665	0.571653036
M121T231	0.571623659	0.961715403	0.571623659

M404T205	0.571521871	0.961704141	0.571521871
M1009T178	0.571344351	0.961684499	0.571344351
M666T32	0.570935291	0.961639608	0.570935291
M472T103	0.570468053	0.961587521	0.570468053
M390T346	0.570297973	0.961568694	0.570297973
M315T454	0.570146004	0.961551871	0.570146004
M1147T39	0.570119203	0.961548936	0.570119203
M465T210	0.56987441	0.961521803	0.56987441
M449T344	0.569707706	0.961503347	0.569707706
M520T153	0.569594113	0.961490781	0.569594113
M362T222	0.56957978	0.961489371	0.56957978
M315T229	0.569231522	0.961450617	0.569231522
M930T78	0.569129284	0.961439295	0.569129284
M307T53	0.568748742	0.961397148	0.568748742
M384T271	0.5687103	0.961393029	0.5687103
M434T115	0.56865475	0.961386737	0.56865475
M793T89	0.568431059	0.96136212	0.568431059
M565T317	0.568217849	0.961338339	0.568217849
M233T264	0.568102237	0.961325531	0.568102237
M166T376	0.567969445	0.961310827	0.567969445
M696T283	0.567852201	0.96129785	0.567852201
M193T267	0.567582299	0.961267921	0.567582299
M159T35	0.567448882	0.961253137	0.567448882
M327T49	0.566993439	0.961202669	0.566993439
M207T33	0.566681155	0.961168048	0.566681155
M221T231	0.566498393	0.961147806	0.566498393
M578T134	0.56638028	0.9611347	0.56638028
M503T225	0.566332749	0.961129427	0.566332749
M831T540	0.566243468	0.961119528	0.566243468
M521T215	0.566093507	0.961103356	0.566093507
M693T32_3	0.5656431	0.961052961	0.5656431
M703T56_1	0.565539277	0.96104145	0.565539277
M927T423	0.565525789	0.961039953	0.565525789
M520T169_2	0.565291141	0.961013932	0.565291141
M619T214	0.565094985	0.960992177	0.565094985
M206T157	0.564642011	0.960941935	0.564642011
M379T309	0.564306624	0.96090473	0.564306624
M924T34	0.564204206	0.960893368	0.564204206
M268T25	0.56419189	0.960892026	0.56419189
M418T232	0.563827381	0.960851561	0.563827381
M1039T49	0.563362561	0.960799991	0.563362561
M309T393	0.563288949	0.960791814	0.563288949
M201T234	0.563075318	0.960768106	0.563075318

M883T182	0.563006851	0.960760568	0.563006851
M433T34	0.562920886	0.960750971	0.562920886
M446T290	0.562822208	0.960740054	0.562822208
M599T168	0.56194684	0.960642885	0.56194684
M93T231	0.561881425	0.960635583	0.561881425
M693T247	0.561436184	0.960586166	0.561436184
M233T53	0.561322757	0.96057357	0.561322757
M1029T401	0.561284879	0.960569377	0.561284879
M114T310	0.560783678	0.96051369	0.560783678
M429T214	0.560510114	0.960483307	0.560510114
M473T236	0.5602238	0.960451507	0.5602238
M578T36	0.560176568	0.96044626	0.560176568
M783T89	0.559740149	0.960397805	0.559740149
M720T26_1	0.559730579	0.96039672	0.559730579
M340T498	0.559472166	0.960367999	0.559472166
M581T115	0.559369091	0.960356562	0.559369091
M694T190	0.559038498	0.960319905	0.559038498
M257T64	0.558987904	0.96031421	0.558987904
M86T46	0.558889117	0.960303234	0.558889117
M438T109	0.558825399	0.960296152	0.558825399
M343T200	0.558683944	0.960280432	0.558683944
M376T76	0.558382021	0.960246895	0.558382021
M1153T422_4	0.558344265	0.960242715	0.558344265
M744T81	0.558161124	0.960222396	0.558161124
M449T173	0.558073758	0.960212627	0.558073758
M220T32	0.55805992	0.960211087	0.55805992
M204T90	0.557971174	0.960201225	0.557971174
M576T230	0.557790287	0.960181152	0.557790287
M901T54	0.557754526	0.960177148	0.557754526
M506T345	0.557514412	0.960150458	0.557514412
M322T423	0.557232599	0.960119133	0.557232599
M1051T185	0.557168651	0.960112026	0.557168651
M749T112	0.557058464	0.960099866	0.557058464
M143T153	0.556887858	0.960080809	0.556887858
M579T595	0.556868867	0.9600787	0.556868867
M226T76	0.55678205	0.960069048	0.55678205
M1116T54	0.556671695	0.960056799	0.556671695
M589T170	0.556630399	0.960052259	0.556630399
M221T87	0.556566301	0.960045062	0.556566301
M159T110	0.556268743	0.960011985	0.556268743
M203T405	0.55603769	0.95998629	0.55603769
M314T454_1	0.556026869	0.959985086	0.556026869
M156T168	0.555904099	0.959971437	0.555904099

M667T200	0.55518599	0.959891616	0.55518599
M499T35	0.554999587	0.959870875	0.554999587
M439T271	0.554885958	0.959858279	0.554885958
M121T45	0.554827782	0.959851744	0.554827782
M496T174	0.554687693	0.959836165	0.554687693
M511T351	0.553700287	0.959726334	0.553700287
M305T60	0.553693186	0.959725546	0.553693186
M314T454_2	0.553243258	0.959675492	0.553243258
M619T66	0.552885706	0.959635846	0.552885706
M303T180	0.55272544	0.959617886	0.55272544
M252T27_2	0.552707299	0.959616128	0.552707299
M295T345	0.552672843	0.959612032	0.552672843
M324T128	0.552604761	0.959604661	0.552604761
M547T120	0.5524556	0.959587858	0.5524556
M948T39	0.552195448	0.959559072	0.552195448
M944T228	0.552048945	0.95954261	0.552048945
M896T57	0.551903321	0.95952641	0.551903321
M140T55	0.551506772	0.959482481	0.551506772
M607T33	0.551450841	0.959476054	0.551450841
M580T348	0.551350524	0.959464888	0.551350524
M128T59	0.551302843	0.959459584	0.551302843
M436T104	0.550974662	0.959423062	0.550974662
M761T89	0.550951305	0.95942048	0.550951305
M424T33	0.550417132	0.959361011	0.550417132
M341T200_1	0.550254601	0.959342917	0.550254601
M213T104_2	0.549970225	0.959311267	0.549970225
M520T317	0.549821253	0.959294685	0.549821253
M743T81	0.549504916	0.959259473	0.549504916
M328T315	0.54948076	0.959256787	0.54948076
M239T40	0.549229674	0.959228848	0.549229674
M407T183	0.549138918	0.959218733	0.549138918
M673T34_2	0.548979042	0.959200939	0.548979042
M773T155	0.548929338	0.959195401	0.548929338
M723T36	0.548620528	0.959161027	0.548620528
M207T32_1	0.548585353	0.959157108	0.548585353
M526T36	0.54850082	0.959147696	0.54850082
M217T278	0.548183318	0.959112466	0.548183318
M441T28_1	0.547965156	0.95908806	0.547965156
M677T201	0.547762553	0.959065505	0.547762553
M387T32	0.547361259	0.959020826	0.547361259
M237T24	0.547297353	0.959013712	0.547297353
M247T34	0.547165582	0.95899911	0.547165582
M585T169	0.546163529	0.958887477	0.546163529

M1132T55	0.546016753	0.958871354	0.546016753
M699T33_2	0.545551055	0.958819268	0.545551055
M363T390	0.545521677	0.958815991	0.545521677
M410T209	0.545497692	0.958813319	0.545497692
M289T59	0.545206006	0.958780841	0.545206006
M483T26	0.545162236	0.958775967	0.545162236
M451T175	0.544838451	0.95873991	0.544838451
M579T37	0.544227466	0.958671869	0.544227466
M986T167	0.543984878	0.958644859	0.543984878
M1138T59	0.543657569	0.958608402	0.543657569
M284T34_2	0.543538141	0.958595101	0.543538141
M235T64	0.54350844	0.958591798	0.54350844
M465T263	0.543481007	0.958588739	0.543481007
M147T299	0.543360772	0.958576599	0.543360772
M1191T422_1	0.543267908	0.958565005	0.543267908
M885T53	0.543209573	0.958558605	0.543209573
M72T233	0.543194053	0.958577499	0.543194053
M349T292	0.54315028	0.95855191	0.54315028
M585T112	0.543132872	0.958549969	0.543132872
M362T347	0.543089275	0.958545113	0.543089275
M310T311	0.542263804	0.958453183	0.542263804
M109T57	0.541800889	0.958401628	0.541800889
M559T336	0.541481239	0.9583661	0.541481239
M728T36	0.541299452	0.958345782	0.541299452
M854T352	0.541100708	0.958323666	0.541100708
M896T145	0.541025097	0.958315263	0.541025097
M255T288	0.540864482	0.958297406	0.540864482
M369T203	0.540544227	0.958262122	0.540544227
M373T35	0.540347533	0.958239772	0.540347533
M662T200	0.540303082	0.95823482	0.540303082
M751T292	0.540044563	0.958206058	0.540044563
M611T413	0.539882674	0.958188157	0.539882674
M313T158	0.539561838	0.95815231	0.539561838
M489T26	0.539468522	0.958141881	0.539468522
M672T213	0.539422763	0.958136782	0.539422763
M595T463	0.539149575	0.958106365	0.539149575
M289T34	0.539112482	0.958102228	0.539112482
M202T278	0.538890523	0.958077516	0.538890523
M439T122	0.537886788	0.957965742	0.537886788
M336T214	0.537830339	0.957959453	0.537830339
M570T313	0.537719419	0.957947111	0.537719419
M993T169	0.537633893	0.957937584	0.537633893
M326T40	0.536420879	0.957802509	0.536420879

M927T422_5	0.536192937	0.957777129	0.536192937
M833T37	0.535919687	0.957746705	0.535919687
M695T247	0.535571064	0.957707892	0.535571064
M457T69	0.535570126	0.957707788	0.535570126
M473T26	0.53480335	0.957622446	0.53480335
M425T541	0.534766607	0.957618351	0.534766607
M427T300	0.534494947	0.957588091	0.534494947
M119T169	0.534349294	0.957571879	0.534349294
M740T171	0.534159289	0.957550805	0.534159289
M807T138	0.533843378	0.957515562	0.533843378
M177T242	0.533658687	0.957495004	0.533658687
M519T169_1	0.533555342	0.95748351	0.533555342
M149T257	0.533478699	0.957474979	0.533478699
M1084T80	0.533354581	0.957461157	0.533354581
M903T210	0.533131574	0.957436382	0.533131574
M635T414	0.533128185	0.957435961	0.533128185
M528T34	0.532843628	0.957404991	0.532843628
M465T213	0.532782465	0.957397483	0.532782465
M595T292	0.532624734	0.957379929	0.532624734
M754T348	0.532542024	0.957370724	0.532542024
M188T285	0.532391992	0.957354029	0.532391992
M231T81	0.53235674	0.957350164	0.53235674
M552T152	0.531763568	0.957284097	0.531763568
M301T42	0.531750282	0.957282637	0.531750282
M395T35	0.53171655	0.957278875	0.53171655
M164T513	0.5312894	0.957231642	0.5312894
M230T162	0.531267985	0.95722896	0.531267985
M233T314	0.531095882	0.957209806	0.531095882
M368T221	0.530140523	0.957103524	0.530140523
M149T49	0.529578861	0.957041051	0.529578861
M172T53	0.529519634	0.957034464	0.529519634
M237T356	0.529439939	0.9570256	0.529439939
M699T204	0.529431718	0.957024715	0.529431718
M360T116_1	0.529348903	0.957015476	0.529348903
M875T39	0.529283093	0.957008237	0.529283093
M145T169	0.529169979	0.956995579	0.529169979
M460T26	0.528902935	0.956966172	0.528902935
M215T31	0.528831127	0.956957893	0.528831127
M294T269	0.52880561	0.956955068	0.52880561
M665T38	0.528802405	0.956954839	0.528802405
M968T78	0.52818293	0.956885958	0.52818293
M791T200	0.528112152	0.956877945	0.528112152
M668T36	0.528050484	0.956871208	0.528050484

M91T231	0.527874515	0.956851526	0.527874515
M166T177	0.527781133	0.956841144	0.527781133
M149T114	0.527667647	0.956828529	0.527667647
M1111T172	0.52731609	0.956789497	0.52731609
M193T354	0.527115767	0.956767465	0.527115767
M677T31	0.526900277	0.956743576	0.526900277
M985T179	0.526770477	0.956728801	0.526770477
M428T346	0.526685149	0.95671932	0.526685149
M86T497	0.526643078	0.95671464	0.526643078
M455T33	0.526471632	0.956695584	0.526471632
M437T85	0.526392325	0.956687258	0.526392325
M117T304	0.526375119	0.956684859	0.526375119
M322T103	0.526321414	0.956679074	0.526321414
M852T349	0.526293254	0.956675761	0.526293254
M298T499	0.526114656	0.956655914	0.526114656
M264T150	0.526080732	0.956652148	0.526080732
M549T595	0.525949987	0.95663762	0.525949987
M439T393	0.525555729	0.956593809	0.525555729
M680T499	0.525360839	0.956572157	0.525360839
M314T220	0.525284056	0.956563764	0.525284056
M659T169_2	0.525227747	0.956557371	0.525227747
M737T44	0.52490782	0.956521848	0.52490782
M847T85	0.524294009	0.956453655	0.524294009
M932T353	0.524281489	0.956452262	0.524281489
M441T169	0.524084744	0.956430412	0.524084744
M835T168	0.523970338	0.95641771	0.523970338
M534T176	0.523832181	0.956402776	0.523832181
M396T106	0.523825243	0.956401597	0.523825243
M225T109	0.523644604	0.956381554	0.523644604
M237T64	0.52363961	0.956380985	0.52363961
M332T258	0.523474827	0.956362686	0.523474827
M173T299	0.523133206	0.956324757	0.523133206
M229T118	0.522934656	0.956302723	0.522934656
M209T106	0.522851076	0.956293457	0.522851076
M273T55	0.522421085	0.956245705	0.522421085
M425T35	0.522406872	0.956244132	0.522406872
M930T423_4	0.522061241	0.956205778	0.522061241
M784T89	0.522039188	0.956203328	0.522039188
M230T306	0.522017391	0.956200906	0.522017391
M421T111	0.521981869	0.956196968	0.521981869
M451T298	0.521621032	0.956156929	0.521621032
M1096T110	0.521589605	0.956153443	0.521589605
M531T32	0.521451905	0.956138165	0.521451905

M750T169	0.521448803	0.956137817	0.521448803
M350T83	0.521216421	0.956112039	0.521216421
M813T129	0.52121248	0.956111616	0.52121248
M387T258	0.521198081	0.956110003	0.521198081
M615T35	0.521014308	0.956089638	0.521014308
M316T240	0.520996078	0.956087594	0.520996078
M791T70	0.520679477	0.956052502	0.520679477
M734T69	0.520665982	0.956051395	0.520665982
M275T246	0.520606155	0.95604437	0.520606155
M201T255	0.520490984	0.956031573	0.520490984
M379T292	0.520331849	0.956013925	0.520331849
M402T103	0.520129147	0.955991445	0.520129147
M1133T54	0.519887859	0.955964692	0.519887859
M330T39	0.51971316	0.955945358	0.51971316
M569T201	0.519707601	0.955945168	0.519707601
M874T349	0.519597958	0.955932551	0.519597958
M283T292	0.519496694	0.955921325	0.519496694
M536T100	0.519483137	0.9559198	0.519483137
M185T151	0.519423233	0.95591318	0.519423233
M931T422_4	0.519062392	0.95587349	0.519062392
M275T56	0.518923352	0.955857805	0.518923352
M874T39	0.518877363	0.955852681	0.518877363
M221T109	0.518531	0.955814298	0.518531
M160T242	0.518517351	0.955812782	0.518517351
M1002T167	0.518342148	0.955793369	0.518342148
M890T39	0.518046899	0.955760732	0.518046899
M803T169	0.517963736	0.955751449	0.517963736
M255T242	0.517922234	0.955746847	0.517922234
M299T41	0.517648113	0.955716489	0.517648113
M418T110	0.517111018	0.955657003	0.517111018
M873T129	0.516440647	0.955582785	0.516440647
M520T205	0.515934244	0.955526757	0.515934244
M337T380	0.515878259	0.955520539	0.515878259
M710T36	0.515869482	0.955519573	0.515869482
M679T256	0.515823865	0.95551452	0.515823865
M489T160	0.515811219	0.955513123	0.515811219
M649T320	0.515658799	0.955496255	0.515658799
M119T462	0.515455124	0.955473724	0.515455124
M654T260	0.515369027	0.955464197	0.515369027
M379T37	0.515140958	0.955438962	0.515140958
M984T176	0.515124511	0.955437143	0.515124511
M261T92	0.514850871	0.955406878	0.514850871
M305T83	0.514840292	0.955405763	0.514840292

M457T204	0.514827026	0.955404398	0.514827026
M537T33	0.514738344	0.955394429	0.514738344
M306T36	0.5146176	0.95538108	0.5146176
M425T581	0.513949158	0.955307169	0.513949158
M851T78	0.513446476	0.95525161	0.513446476
M797T198	0.512892212	0.955190363	0.512892212
M480T274	0.512880654	0.955189088	0.512880654
M225T26	0.512765876	0.955176415	0.512765876
M866T35	0.512590669	0.955157226	0.512590669
M609T50	0.512467097	0.955143404	0.512467097
M392T345	0.512409105	0.955137014	0.512409105
M685T346	0.512272427	0.955121904	0.512272427
M715T211	0.512200275	0.955113936	0.512200275
M462T315	0.511815787	0.955071484	0.511815787
M1041T82	0.511495899	0.955036179	0.511495899
M360T344	0.511439721	0.955029978	0.511439721
M374T255	0.51128209	0.955012588	0.51128209
M186T26	0.51121857	0.955005561	0.51121857
M467T138_2	0.511171277	0.955000342	0.511171277
M397T56	0.510934749	0.95497522	0.510934749
M373T42	0.510572158	0.954934235	0.510572158
M391T255	0.510420737	0.954917554	0.510420737
M686T34	0.510355155	0.9549103	0.510355155
M675T66	0.510345271	0.954909215	0.510345271
M738T52	0.510092531	0.954881336	0.510092531
M220T117	0.510084873	0.954880522	0.510084873
M766T81_2	0.51004136	0.954875693	0.51004136
M730T56	0.509907796	0.954860961	0.509907796
M229T41	0.50966163	0.954833829	0.50966163
M917T55	0.509470283	0.954812714	0.509470283
M978T31	0.509466767	0.954812343	0.509466767
M605T168	0.509303493	0.954794342	0.509303493
M495T53	0.50923888	0.954787222	0.50923888
M286T248	0.509090815	0.954770909	0.509090815
M218T34	0.50885081	0.954744612	0.50885081
M320T346	0.508806912	0.954739615	0.508806912
M779T53	0.5087992	0.954738839	0.5087992
M458T242	0.508792579	0.954738038	0.508792579
M502T36	0.508596915	0.954716482	0.508596915
M247T263	0.508374407	0.954691972	0.508374407
M267T220	0.508356832	0.954690032	0.508356832
M331T305	0.508262531	0.95467965	0.508262531
M114T181	0.508148032	0.954667211	0.508148032

M283T539	0.508018509	0.954652774	0.508018509
M142T299	0.507867425	0.954636139	0.507867425
M515T151	0.507810965	0.954629921	0.507810965
M517T326	0.507599445	0.954606632	0.507599445
M342T333	0.50748973	0.954594558	0.50748973
M322T256	0.507233432	0.954566346	0.507233432
M593T292	0.507123871	0.954554723	0.507123871
M930T423_1	0.50704525	0.954545649	0.50704525
M346T424	0.506959943	0.954536445	0.506959943
M365T230	0.506724943	0.954510393	0.506724943
M979T168_2	0.506623371	0.95449922	0.506623371
M309T169	0.506538077	0.954489839	0.506538077
M681T33	0.506378505	0.954472613	0.506378505
M292T540_1	0.506084009	0.954439902	0.506084009
M282T92	0.505967608	0.954427124	0.505967608
M105T37	0.505585569	0.954385104	0.505585569
M854T348	0.50526185	0.954349825	0.50526185
M312T34	0.505255236	0.954348805	0.505255236
M273T221	0.504884206	0.954308036	0.504884206
M116T309	0.504750479	0.954293346	0.504750479
M670T199	0.504206995	0.954233661	0.504206995
M336T35_2	0.504090454	0.954220866	0.504090454
M460T348	0.504035025	0.954215298	0.504035025
M771T154	0.503991576	0.954210014	0.503991576
M919T55	0.503759281	0.954184518	0.503759281
M374T310	0.503188322	0.954121895	0.503188322
M588T155	0.502708463	0.954069242	0.502708463
M407T150	0.502512743	0.954047855	0.502512743
M192T678	0.501889569	0.953979484	0.501889569
M339T294	0.501832693	0.953973249	0.501832693
M144T352	0.501791668	0.953968759	0.501791668
M945T465_2	0.50172559	0.953961515	0.50172559
M325T186	0.501691081	0.953957738	0.501691081
M243T297	0.501421438	0.953928202	0.501421438
M938T32	0.500758665	0.953855635	0.500758665
M877T127	0.500578233	0.953835885	0.500578233
M314T467	0.500568106	0.953834779	0.500568106
M263T27	0.500457309	0.953822657	0.500457309
M461T343	0.500148411	0.95378949	0.500148411
M367T370	0.500042636	0.95377729	0.500042636
M372T304	0.499929518	0.953764918	0.499929518
M401T247	0.499573645	0.953726177	0.499573645
M500T212	0.499491931	0.953717068	0.499491931

M754T52	0.499126937	0.953677175	0.499126937
M321T162	0.498755845	0.953637284	0.498755845
M373T335	0.498680968	0.953628477	0.498680968
M1162T423_4	0.498552718	0.953614453	0.498552718
M343T94	0.498243292	0.953580902	0.498243292
M155T352	0.498086605	0.953563544	0.498086605
M205T35	0.497345425	0.953482666	0.497345425
M317T308	0.497282114	0.953476016	0.497282114
M475T117	0.496913038	0.953435509	0.496913038
M180T36	0.496800884	0.953423743	0.496800884
M225T325	0.49675205	0.953417958	0.49675205
M906T38	0.496441236	0.95338408	0.496441236
M294T367	0.49633778	0.953372834	0.49633778
M553T186	0.496239953	0.953362147	0.496239953
M74T366	0.495977561	0.953333894	0.495977561
M470T122	0.495551131	0.953287127	0.495551131
M361T35	0.494957053	0.953222469	0.494957053
M703T210	0.494934898	0.953220057	0.494934898
M321T183	0.494369728	0.953158589	0.494369728
M249T79	0.494285399	0.953149424	0.494285399
M536T35	0.494271008	0.95314786	0.494271008
M279T93	0.494258469	0.953146494	0.494258469
M271T34	0.494117088	0.953131507	0.494117088
M97T181	0.493998463	0.953118236	0.493998463
M70T45	0.493870054	0.953104282	0.493870054
M855T57	0.49349693	0.953063744	0.49349693
M701T212	0.49311673	0.953022842	0.49311673
M315T164	0.492731754	0.952981805	0.492731754
M656T293	0.492670984	0.952974077	0.492670984
M449T123	0.492658176	0.952972688	0.492658176
M1006T177	0.492330006	0.952937086	0.492330006
M332T351	0.492319966	0.952936	0.492319966
M926T422_1	0.492054035	0.952907161	0.492054035
M936T108	0.491810555	0.952881111	0.491810555
M687T353	0.491732477	0.952872303	0.491732477
M295T143	0.491601155	0.952858117	0.491601155
M261T64	0.49154981	0.952852743	0.49154981
M191T370	0.49148801	0.952845809	0.49148801
M150T34	0.491469481	0.952843802	0.491469481
M379T53	0.491361957	0.952832174	0.491361957
M340T35	0.491254037	0.952820462	0.491254037
M284T25	0.49111462	0.952805364	0.49111462
M505T36_2	0.490777047	0.952768831	0.490777047

M360T415	0.490734017	0.952764205	0.490734017
M565T127	0.490372117	0.952724997	0.490372117
M767T58	0.490351625	0.952722773	0.490351625
M290T121	0.489964915	0.952680945	0.489964915
M345T83	0.489928522	0.95267701	0.489928522
M254T334	0.48981443	0.952664674	0.48981443
M374T211	0.489724711	0.952654974	0.489724711
M372T484	0.489672406	0.952649321	0.489672406
M94T94	0.489592174	0.952640654	0.489592174
M438T169	0.489194328	0.952597657	0.489194328
M1062T59	0.48918749	0.952597049	0.48918749
M531T202	0.489126734	0.952590359	0.489126734
M160T397	0.488883238	0.952564059	0.488883238
M521T155	0.48874643	0.952549632	0.48874643
M872T39	0.488746133	0.952549257	0.488746133
M655T212	0.488500552	0.952522745	0.488500552
M192T366	0.488132837	0.952483071	0.488132837
M341T49	0.488128154	0.952482962	0.488128154
M435T34	0.488117305	0.952481402	0.488117305
M757T91	0.487998218	0.952468555	0.487998218
M829T106	0.48778636	0.952445671	0.48778636
M462T39	0.487785317	0.952445598	0.487785317
M204T258	0.487742737	0.952442195	0.487742737
M209T177	0.487601304	0.952425757	0.487601304
M431T33	0.487372252	0.952401069	0.487372252
M735T36	0.487269258	0.952389973	0.487269258
M491T273	0.487230207	0.952385881	0.487230207
M264T346	0.487023515	0.952363498	0.487023515
M159T150	0.486914961	0.952351808	0.486914961
M281T27	0.486610498	0.95231903	0.486610498
M206T185	0.486379836	0.952294206	0.486379836
M403T33	0.485850999	0.95223732	0.485850999
M518T154	0.485604919	0.952210869	0.485604919
M239T191	0.485348873	0.952183352	0.485348873
M96T272	0.485103291	0.952156978	0.485103291
M841T38	0.485062722	0.952152616	0.485062722
M359T234	0.484725144	0.952116391	0.484725144
M879T174	0.484665257	0.952109945	0.484665257
M364T404	0.483871472	0.952024795	0.483871472
M1095T37	0.483793432	0.952016437	0.483793432
M259T620	0.483743824	0.952011116	0.483743824
M725T403	0.483601513	0.951995916	0.483601513
M1034T174	0.483581449	0.951993714	0.483581449

M311T351	0.483410826	0.951975429	0.483410826
M195T354	0.48302909	0.951934509	0.48302909
M687T101	0.482873574	0.951917906	0.482873574
M365T116	0.482630402	0.951891886	0.482630402
M801T115	0.482625487	0.951891358	0.482625487
M1067T187	0.482527668	0.951880896	0.482527668
M481T169	0.4825169	0.951879742	0.4825169
M591T161	0.482332745	0.951860045	0.482332745
M264T167	0.482329718	0.95185975	0.482329718
M869T52	0.482206429	0.951846538	0.482206429
M408T380	0.4820947	0.951834594	0.4820947
M115T73	0.48200653	0.951825187	0.48200653
M295T18	0.481775935	0.951801083	0.481775935
M1020T147	0.481424942	0.951763038	0.481424942
M545T97	0.48120595	0.95178658	0.48120595
M495T168	0.481131432	0.951731705	0.481131432
M183T370	0.480498414	0.951664179	0.480498414
M589T351	0.480419056	0.951655718	0.480419056
M192T19	0.480201317	0.95163251	0.480201317
M933T42	0.480155671	0.951627647	0.480155671
M669T34	0.480143805	0.951626383	0.480143805
M1097T34_1	0.480026791	0.951613915	0.480026791
M912T351	0.479939828	0.951605426	0.479939828
M528T105	0.479922119	0.951602773	0.479922119
M353T130	0.47990708	0.951601166	0.47990708
M705T264	0.479771991	0.951586778	0.479771991
M849T52	0.479339653	0.95154076	0.479339653
M927T422_2	0.479170437	0.951522757	0.479170437
M487T121	0.479115891	0.951517315	0.479115891
M559T32	0.479042985	0.951509202	0.479042985
M787T131	0.478473856	0.951448706	0.478473856
M256T80	0.478138586	0.951413097	0.478138586
M1000T180	0.478122334	0.951411382	0.478122334
M293T177	0.477777813	0.951374805	0.477777813
M743T38	0.477053399	0.951297992	0.477053399
M481T211	0.477048435	0.951297463	0.477048435
M923T52	0.476974888	0.951289676	0.476974888
M617T293	0.476889989	0.951280682	0.476889989
M771T89_2	0.476652622	0.951255542	0.476652622
M507T210	0.476527318	0.951242272	0.476527318
M603T32	0.476249946	0.951213266	0.476249946
M312T146	0.476242468	0.951212126	0.476242468
M268T383	0.475542623	0.951138182	0.475542623

M300T479	0.475523977	0.951136159	0.475523977
M1016T184	0.475369556	0.951119845	0.475369556
M360T200_1	0.475101493	0.951091537	0.475101493
M179T260	0.475088778	0.951090195	0.475088778
M783T162	0.474728625	0.951052185	0.474728625
M467T35_1	0.47468612	0.9510477	0.47468612
M805T353	0.474631531	0.951041944	0.474631531
M621T71	0.474400303	0.951017616	0.474400303
M563T413	0.474395873	0.95101711	0.474395873
M268T580	0.474231824	0.950999804	0.474231824
M262T51	0.47414536	0.950990687	0.47414536
M578T41	0.473818641	0.950956275	0.473818641
M585T203	0.473732491	0.950947197	0.473732491
M376T73	0.473546432	0.950927735	0.473546432
M418T85	0.473254141	0.950896861	0.473254141
M296T254	0.472978265	0.950867848	0.472978265
M519T123	0.472973527	0.950867349	0.472973527
M267T33	0.472715486	0.950840232	0.472715486
M358T404	0.472601637	0.950828273	0.472601637
M185T26	0.472574113	0.950825379	0.472574113
M947T166	0.472497501	0.950817335	0.472497501
M584T35	0.472443034	0.950811859	0.472443034
M420T234	0.471856544	0.950750061	0.471856544
M355T449	0.471833496	0.950747643	0.471833496
M357T354	0.471554465	0.950718389	0.471554465
M605T200	0.471442258	0.950706729	0.471442258
M347T422	0.471319885	0.950693806	0.471319885
M70T309	0.471096602	0.95067042	0.471096602
M194T33	0.471028553	0.950663298	0.471028553
M1157T422	0.470073963	0.950563459	0.470073963
M193T181	0.469945593	0.950550046	0.469945593
M297T36	0.469906155	0.950545929	0.469906155
M398T56	0.469339388	0.950486865	0.469339388
M293T540_2	0.469237551	0.950476147	0.469237551
M484T262	0.468930203	0.950444106	0.468930203
M787T53	0.468906448	0.950441929	0.468906448
M245T394	0.468834046	0.950434129	0.468834046
M529T114	0.467999332	0.950347202	0.467999332
M366T384_1	0.467943188	0.950341366	0.467943188
M139T78	0.467748585	0.950321142	0.467748585
M198T66	0.467745227	0.950320786	0.467745227
M298T319	0.467586953	0.950304342	0.467586953
M309T292	0.467484281	0.950293675	0.467484281

M484T82	0.467385286	0.950283393	0.467385286
M324T424	0.467279329	0.950272461	0.467279329
M203T258_1	0.466961318	0.950239402	0.466961318
M187T280	0.466672293	0.950209415	0.466672293
M517T204	0.46630861	0.950171732	0.46630861
M429T33	0.466120025	0.950152267	0.466120025
M140T181	0.466024615	0.950142321	0.466024615
M118T247	0.465953365	0.95013495	0.465953365
M372T395	0.465481055	0.950086102	0.465481055
M302T190	0.465056637	0.950042248	0.465056637
M272T108	0.46435032	0.949969381	0.46435032
M657T27	0.463770042	0.949909608	0.463770042
M347T403	0.46359936	0.949892046	0.46359936
M399T243	0.463435707	0.949875524	0.463435707
M189T85	0.463381493	0.949870473	0.463381493
M890T53	0.463326436	0.949863981	0.463326436
M612T450	0.462982547	0.949828585	0.462982547
M1182T465_2	0.462971235	0.949827479	0.462971235
M391T345	0.462842003	0.949814206	0.462842003
M77T35	0.462559595	0.949785221	0.462559595
M909T35	0.46215301	0.949743528	0.46215301
M612T36	0.462013847	0.949729267	0.462013847
M381T84	0.461642838	0.949729996	0.461642838
M233T55	0.461558623	0.949682654	0.461558623
M189T307	0.461531126	0.949679838	0.461531126
M814T347	0.461373043	0.949663665	0.461373043
M408T194	0.460921554	0.949617518	0.460921554
M215T120	0.460839493	0.949609136	0.460839493
M276T56	0.460747585	0.949600126	0.460747585
M772T67	0.460114864	0.94953521	0.460114864
M98T675	0.460068179	0.949530455	0.460068179
M323T32	0.460033171	0.949526879	0.460033171
M883T52	0.459998735	0.949523371	0.459998735
M425T221	0.459968326	0.949520274	0.459968326
M614T347	0.459932171	0.949516587	0.459932171
M504T37	0.459603753	0.949483147	0.459603753
M619T353	0.459344051	0.949456723	0.459344051
M875T169_2	0.459280911	0.949450327	0.459280911
M391T103	0.459028692	0.94942487	0.459028692
M375T36	0.458714375	0.949392749	0.458714375
M619T34	0.458633537	0.949384672	0.458633537
M491T26	0.458262838	0.949346936	0.458262838
M180T56	0.45826075	0.949346722	0.45826075

M486T44	0.457969784	0.949317231	0.457969784
M430T79	0.457806307	0.949300678	0.457806307
M589T168_2	0.457781828	0.9492982	0.457781828
M857T124_1	0.457153615	0.949234668	0.457153615
M947T27	0.456547132	0.949173546	0.456547132
M500T346	0.456537304	0.949172428	0.456537304
M173T505	0.455959173	0.949114165	0.455959173
M807T49	0.455678431	0.949085914	0.455678431
M368T87_1	0.455650828	0.949083134	0.455650828
M237T27	0.45553664	0.949071649	0.45553664
M451T146	0.455494126	0.949067373	0.455494126
M314T333	0.455088139	0.949026618	0.455088139
M305T181	0.454998076	0.949017537	0.454998076
M147T59	0.454920268	0.949009726	0.454920268
M708T413	0.454801068	0.948997962	0.454801068
M317T355	0.454690063	0.948986632	0.454690063
M300T432	0.453969133	0.948914412	0.453969133
M103T231	0.453802011	0.94889769	0.453802011
M84T319	0.453781607	0.948895683	0.453781607
M321T58	0.453745214	0.948892019	0.453745214
M478T199	0.453709271	0.948888448	0.453709271
M372T579	0.453402126	0.948857725	0.453402126
M726T52	0.452998208	0.948817412	0.452998208
M387T37	0.452944281	0.948812032	0.452944281
M554T152	0.452170083	0.94873495	0.452170083
M1158T422_4	0.452000313	0.94871817	0.452000313
M786T63	0.451939769	0.948712026	0.451939769
M122T369	0.451905244	0.948708588	0.451905244
M1052T186	0.451829379	0.94870105	0.451829379
M449T105	0.451565179	0.948674816	0.451565179
M803T168	0.451429778	0.948661373	0.451429778
M760T97	0.451393023	0.948657731	0.451393023
M484T482	0.451337157	0.948652908	0.451337157
M363T215	0.451323554	0.948650836	0.451323554
M282T44	0.45132004	0.948650487	0.45132004
M825T52	0.45131974	0.948650458	0.45131974
M301T34	0.451309131	0.948649408	0.451309131
M215T381	0.451258488	0.948644381	0.451258488
M301T104	0.451196789	0.948638336	0.451196789
M891T52	0.451074774	0.948626169	0.451074774
M393T344	0.451037265	0.948622455	0.451037265
M825T103	0.450772769	0.948596255	0.450772769
M238T59	0.450655653	0.948584668	0.450655653

M273T19	0.450424875	0.948561839	0.450424875
M571T34	0.450362693	0.948555687	0.450362693
M827T68	0.450362337	0.948555656	0.450362337
M855T352	0.450288418	0.948548346	0.450288418
M263T581	0.450085417	0.948528293	0.450085417
M824T68	0.449702401	0.948490492	0.449702401
M769T107	0.449458763	0.948466615	0.449458763
M1113T171	0.449105819	0.948431716	0.449105819
M191T306	0.449104333	0.948431567	0.449104333
M233T241	0.449081202	0.948429311	0.449081202
M182T43	0.448932892	0.948414699	0.448932892
M397T292	0.448793617	0.948401096	0.448793617
M485T34	0.448586066	0.948380608	0.448586066
M611T51	0.448556512	0.948377715	0.448556512
M399T113	0.448401785	0.948362511	0.448401785
M277T584	0.448267974	0.948349405	0.448267974
M305T266	0.448194438	0.948342171	0.448194438
M701T200	0.448044213	0.948327433	0.448044213
M625T33	0.447816779	0.948305148	0.447816779
M365T363	0.447693226	0.948293045	0.447693226
M127T320_1	0.447520616	0.948276153	0.447520616
M1026T48	0.447431689	0.948267451	0.447431689
M399T53	0.446944125	0.948219807	0.446944125
M170T55	0.446664068	0.948193061	0.446664068
M939T352	0.44623591	0.948150749	0.44623591
M129T48	0.446230563	0.948150265	0.446230563
M393T162	0.445836289	0.948111875	0.445836289
M645T200	0.445695749	0.948098212	0.445695749
M873T39	0.445486251	0.948077866	0.445486251
M681T210	0.445161061	0.948046311	0.445161061
M409T26_1	0.444996069	0.948030323	0.444996069
M363T218	0.444624814	0.947994368	0.444624814
M887T127	0.444590817	0.947991079	0.444590817
M159T178	0.444408277	0.94797344	0.444408277
M161T59	0.444179211	0.947951293	0.444179211
M198T26_3	0.444048826	0.9479387	0.444048826
M527T49	0.444003603	0.947934342	0.444003603
M1012T33	0.443800682	0.947914761	0.443800682
M132T242_1	0.443603083	0.947895717	0.443603083
M491T33_2	0.443537614	0.947889407	0.443537614
M509T311	0.44343784	0.947879801	0.44343784
M246T128	0.443296386	0.947866176	0.443296386
M249T38	0.44329498	0.947866042	0.44329498

M504T262	0.443208414	0.947857725	0.443208414
M1153T422_2	0.443063143	0.947843773	0.443063143
M196T56	0.442653176	0.947804358	0.442653176
M308T214	0.442498361	0.947789501	0.442498361
M202T234	0.442293829	0.947769895	0.442293829
M437T58	0.442158159	0.947756905	0.442158159
M246T403	0.442146932	0.947755818	0.442146932
M147T180	0.442093226	0.947750675	0.442093226
M261T338	0.441988099	0.947740913	0.441988099
M191T34_1	0.441909936	0.947733132	0.441909936
M681T256	0.441817406	0.947724282	0.441817406
M374T107	0.441368386	0.947681377	0.441368386
M225T47	0.44133014	0.947677727	0.44133014
M1181T465_1	0.441120041	0.94765768	0.441120041
M657T33	0.441073868	0.947653576	0.441073868
M359T200	0.440797672	0.947626961	0.440797672
M826T193	0.440708782	0.947618205	0.440708782
M498T33	0.440349636	0.94758435	0.440349636
M889T39	0.439876913	0.947539463	0.439876913
M782T169_2	0.439665957	0.947519494	0.439665957
M1037T186	0.439378861	0.947492279	0.439378861
M372T272_2	0.439222421	0.947477487	0.439222421
M452T255	0.439138017	0.947469502	0.439138017
M187T401	0.43911568	0.947467386	0.43911568
M993T353	0.43895703	0.947452398	0.43895703
M799T109	0.43892856	0.947449712	0.43892856
M462T348	0.438858315	0.947443089	0.438858315
M369T36_2	0.438672411	0.947425532	0.438672411
M703T103	0.438527166	0.947411842	0.438527166
M196T143	0.438478975	0.947407297	0.438478975
M357T483	0.438373799	0.947397389	0.438373799
M973T34	0.438104102	0.947372073	0.438104102
M357T512	0.438091798	0.947370843	0.438091798
M125T244	0.437970499	0.947359445	0.437970499
M443T123	0.437947379	0.947357359	0.437947379
M396T40	0.437751396	0.947338847	0.437751396
M191T258	0.437704482	0.947334437	0.437704482
M337T292	0.437462466	0.947311781	0.437462466
M774T349	0.43732463	0.947299932	0.43732463
M369T98	0.43701724	0.947270011	0.43701724
M131T305	0.436929939	0.94726184	0.436929939
M249T227	0.436778201	0.947247648	0.436778201
M1091T36	0.436600564	0.947231057	0.436600564

M406T153	0.436374688	0.947209961	0.436374688
M157T181	0.436244021	0.947199012	0.436244021
M849T129	0.436193525	0.947193062	0.436193525
M534T168_2	0.43501689	0.947083729	0.43501689
M473T378	0.434966798	0.947079074	0.434966798
M983T172	0.434856985	0.94706889	0.434856985
M397T433	0.434537303	0.947039283	0.434537303
M975T164	0.434432107	0.947029575	0.434432107
M748T167	0.433721616	0.946964021	0.433721616
M623T32	0.433649225	0.946957351	0.433649225
M833T47	0.433594779	0.946952335	0.433594779
M323T348	0.433565488	0.946949642	0.433565488
M1082T51	0.433163685	0.946912709	0.433163685
M793T242	0.433035668	0.946900958	0.433035668
M262T285	0.432961247	0.94689413	0.432961247
M359T152	0.432796571	0.946879243	0.432796571
M201T446	0.432701895	0.94687092	0.432701895
M680T33	0.432335113	0.946836815	0.432335113
M159T107	0.432180479	0.946822665	0.432180479
M707T202	0.431939168	0.946800671	0.431939168
M797T111	0.431611405	0.946770764	0.431611405
M865T80	0.431304268	0.946742815	0.431304268
M104T437	0.430829928	0.946699743	0.430829928
M183T105	0.430743056	0.946691869	0.430743056
M715T214	0.430570163	0.946676211	0.430570163
M465T36	0.43044129	0.946664567	0.43044129
M328T168	0.430429293	0.946663488	0.430429293
M150T59	0.430349146	0.946656216	0.430349146
M382T114	0.42972668	0.946600016	0.42972668
M239T382	0.429485406	0.94657828	0.429485406
M249T27	0.429350828	0.94656619	0.429350828
M309T336	0.429236262	0.946555874	0.429236262
M923T228	0.428931749	0.946528572	0.428931749
M1192T422_3	0.428829157	0.946519326	0.428829157
M768T87	0.428696083	0.946507688	0.428696083
M375T34	0.428682159	0.946506152	0.428682159
M558T38	0.428680252	0.946505981	0.428680252
M1162T423_2	0.428499284	0.946489839	0.428499284
M97T320	0.428401019	0.946481068	0.428401019
M451T32	0.428361739	0.94647747	0.428361739
M117T205_1	0.428354964	0.946477074	0.428354964
M960T115	0.428173642	0.946460696	0.428173642
M150T167	0.428116254	0.946455535	0.428116254

M453T121	0.428099702	0.94645413	0.428099702
M423T99	0.427679097	0.94641655	0.427679097
M328T26	0.427565332	0.946406424	0.427565332
M175T207	0.427393558	0.946391182	0.427393558
M782T79	0.427318923	0.946384506	0.427318923
M168T385	0.427034088	0.946359222	0.427034088
M1048T201	0.426955788	0.946352271	0.426955788
M459T202	0.426745492	0.94633363	0.426745492
M229T309	0.426469972	0.946309249	0.426469972
M473T34	0.426310927	0.946295192	0.426310927
M701T178	0.426184934	0.946284069	0.426184934
M675T292	0.426154181	0.946281359	0.426154181
M523T18	0.425791141	0.946249407	0.425791141
M401T122	0.425581795	0.946230936	0.425581795
M194T366	0.425463732	0.946220555	0.425463732
M677T200	0.425444452	0.94621886	0.425444452
M339T328	0.425243161	0.946201189	0.425243161
M529T304	0.424913471	0.946172284	0.424913471
M219T19	0.424773801	0.946160067	0.424773801
M373T304_1	0.424679865	0.946151864	0.424679865
M1158T422_2	0.424596858	0.94614459	0.424596858
M329T143	0.424228076	0.946112396	0.424228076
M277T396	0.424130301	0.946103878	0.424130301
M132T168	0.424076915	0.946099225	0.424076915
M681T294	0.424012657	0.94609363	0.424012657
M189T37	0.423785285	0.946073846	0.423785285
M597T33	0.423542225	0.946052764	0.423542225
M980T168_2	0.42331815	0.946033297	0.42331815
M188T272_1	0.422937551	0.946000355	0.422937551
M305T290	0.422464018	0.945959489	0.422464018
M831T156	0.422309494	0.945946192	0.422309494
M827T106	0.422268803	0.945942667	0.422268803
M890T72	0.421942646	0.945914622	0.421942646
M958T24	0.421883368	0.945909537	0.421883368
M774T51	0.421860328	0.945907562	0.421860328
M1192T422_1	0.421303337	0.945859845	0.421303337
M626T129	0.42117434	0.945848839	0.42117434
M1063T37	0.421162352	0.945848308	0.421162352
M121T35	0.420752245	0.945812816	0.420752245
M964T352	0.42035838	0.945779311	0.42035838
M503T102	0.420337485	0.945777548	0.420337485
M587T57	0.419943481	0.945744133	0.419943481
M257T38	0.4197934	0.945731431	0.4197934

M597T148	0.419558206	0.945711547	0.419558206
M115T242	0.419554581	0.945711238	0.419554581
M378T109	0.419386467	0.945697297	0.419386467
M930T423_2	0.419244505	0.945685085	0.419244505
M201T257	0.418951689	0.945660435	0.418951689
M237T220	0.418625955	0.945633081	0.418625955
M257T26	0.418612635	0.945631978	0.418612635
M519T383	0.418561673	0.945627691	0.418561673
M642T169	0.41850257	0.945622863	0.41850257
M364T224	0.417813921	0.945565171	0.417813921
M496T35	0.417774338	0.945561868	0.417774338
M354T169	0.417577632	0.94554549	0.417577632
M307T59	0.417142927	0.945509364	0.417142927
M777T94_1	0.417069372	0.945503266	0.417069372
M728T26_2	0.41704435	0.945501193	0.41704435
M271T26	0.416580174	0.945462795	0.416580174
M334T344	0.416550097	0.945461355	0.416550097
M351T173	0.416528617	0.945458536	0.416528617
M678T31	0.41644859	0.945451942	0.41644859
M461T211	0.416395378	0.945447537	0.416395378
M693T190	0.416393148	0.945447353	0.416393148
M660T169	0.416378828	0.945446169	0.416378828
M613T35	0.416295218	0.945439333	0.416295218
M631T207	0.416147796	0.945427139	0.416147796
M62T109	0.415894948	0.945406397	0.415894948
M279T373	0.415732294	0.945393871	0.415732294
M213T178	0.415557508	0.945433012	0.415557508
M438T102	0.415541894	0.94537744	0.415541894
M869T125	0.415483196	0.945372572	0.415483196
M301T312	0.415231169	0.945352264	0.415231169
M803T51	0.415057723	0.945337795	0.415057723
M269T47	0.415046977	0.945336918	0.415046977
M741T101	0.414901623	0.945325067	0.414901623
M251T71	0.414489438	0.94529153	0.414489438
M325T246	0.414440809	0.945287577	0.414440809
M861T129	0.414350748	0.945280273	0.414350748
M325T174	0.413677586	0.945225811	0.413677586
M383T34	0.413431751	0.945205991	0.413431751
M140T119	0.413381179	0.945201921	0.413381179
M687T352	0.413214201	0.945188498	0.413214201
M653T33_1	0.412277421	0.945113505	0.412277421
M960T158	0.41211656	0.945100689	0.41211656
M751T202	0.412104958	0.945099824	0.412104958

M589T32	0.411742838	0.945070985	0.411742838
M335T33	0.411640568	0.945062946	0.411640568
M785T131	0.411444647	0.945047359	0.411444647
M899T129	0.411358264	0.945041432	0.411358264
M673T34_1	0.4112797	0.945034312	0.4112797
M195T84	0.411171826	0.945027404	0.411171826
M603T83	0.411169027	0.945025569	0.411169027
M376T338	0.411114846	0.945021487	0.411114846
M788T51	0.411034804	0.945014975	0.411034804
M325T378	0.41089529	0.94500398	0.41089529
M274T304	0.410807785	0.944997099	0.410807785
M1009T171	0.410672545	0.944986455	0.410672545
M487T345	0.410599292	0.94498071	0.410599292
M164T26	0.410166916	0.944946808	0.410166916
M137T33	0.410161699	0.944946481	0.410161699
M899T26	0.410118419	0.94494301	0.410118419
M399T301	0.410089473	0.944940748	0.410089473
M327T698	0.409960646	0.944930681	0.409960646
M669T421	0.409855259	0.944922504	0.409855259
M614T36	0.409799053	0.944918058	0.409799053
M136T26_2	0.409674276	0.94490857	0.409674276
M615T235	0.40941407	0.944888122	0.40941407
M236T187	0.409307394	0.944879888	0.409307394
M813T28	0.409200751	0.944871556	0.409200751
M175T571	0.409158028	0.94486824	0.409158028
M773T109	0.408802468	0.944840783	0.408802468
M156T43	0.408416303	0.944810949	0.408416303
M427T221	0.408297923	0.944801844	0.408297923
M199T26_1	0.408198783	0.944794334	0.408198783
M169T34	0.407987092	0.944779081	0.407987092
M190T327	0.407746586	0.944759584	0.407746586
M864T189	0.407585168	0.944747245	0.407585168
M551T91	0.40758281	0.944747403	0.40758281
M292T483_2	0.407548818	0.944744523	0.407548818
M137T320	0.407139173	0.944713265	0.407139173
M411T37	0.406744793	0.944683343	0.406744793
M216T26_1	0.406424886	0.944659162	0.406424886
M967T259	0.40637965	0.944655772	0.40637965
M685T199	0.406363754	0.944654549	0.406363754
M1044T51	0.406134649	0.944637446	0.406134649
M189T33	0.405784514	0.944610997	0.405784514
M815T53	0.405587044	0.944596239	0.405587044
M281T101	0.405399258	0.944582178	0.405399258

M120T365	0.405278541	0.944573254	0.405278541
M346T145	0.404822241	0.944539236	0.404822241
M321T73	0.404583072	0.944521479	0.404583072
M182T112	0.404489168	0.944514952	0.404489168
M335T52	0.404464133	0.944512674	0.404464133
M639T36	0.404338145	0.944503501	0.404338145
M554T168	0.404285311	0.944500008	0.404285311
M195T181_2	0.404276902	0.944498869	0.404276902
M157T257	0.403800648	0.944463788	0.403800648
M276T133	0.403762317	0.944460975	0.403762317
M419T83	0.403748965	0.944459995	0.403748965
M572T278	0.403736159	0.944459055	0.403736159
M677T284	0.40372228	0.944458046	0.40372228
M390T260	0.403513984	0.944442778	0.403513984
M787T122	0.403036933	0.944407953	0.403036933
M308T120	0.402834287	0.944393219	0.402834287
M593T291	0.402627948	0.944378249	0.402627948
M1010T79	0.402063259	0.944337894	0.402063259
M112T424	0.402052419	0.944336677	0.402052419
M534T36_1	0.401630653	0.944306382	0.401630653
M145T258	0.401622295	0.944305783	0.401622295
M828T107	0.401611583	0.944305017	0.401611583
M757T81	0.401536198	0.94430048	0.401536198
M409T352	0.40148338	0.944295835	0.40148338
M895T173	0.401466246	0.944294614	0.401466246
M405T26	0.400979047	0.944259876	0.400979047
M241T96	0.400772745	0.944245221	0.400772745
M519T138	0.400513113	0.944227012	0.400513113
M716T34	0.399929229	0.944185698	0.399929229
M497T107	0.399918726	0.944184999	0.399918726
M783T27	0.399228897	0.944136724	0.399228897
M449T135	0.3989622	0.944118192	0.3989622
M935T32	0.398726059	0.944101826	0.398726059
M265T676	0.398703151	0.944100244	0.398703151
M307T338	0.398573555	0.944091306	0.398573555
M393T258	0.398435645	0.944081775	0.398435645
M142T53	0.398252044	0.944069145	0.398252044
M661T294	0.398054072	0.944055538	0.398054072
M205T300	0.3980399	0.944054568	0.3980399
M289T467	0.398000577	0.944052458	0.398000577
M127T27	0.39761935	0.944025807	0.39761935
M118T370	0.39745451	0.944014613	0.39745451
M981T159	0.397341963	0.944007005	0.397341963

M160T262	0.397039438	0.943986403	0.397039438
M75T105	0.396993516	0.943983327	0.396993516
M527T110	0.396983175	0.943982595	0.396983175
M1066T57	0.396802348	0.943970378	0.396802348
M99T255	0.396425474	0.943945063	0.396425474
M663T306	0.396314	0.943937536	0.396314
M446T414	0.396158862	0.943927146	0.396158862
M261T18	0.396001409	0.943916623	0.396001409
M880T55	0.395961725	0.943913981	0.395961725
M685T34_1	0.395927575	0.943911699	0.395927575
M265T220	0.39588644	0.943908956	0.39588644
M251T409	0.395541367	0.943886013	0.395541367
M757T349	0.395376886	0.943875116	0.395376886
M367T217_2	0.395206603	0.943863865	0.395206603
M1031T127	0.394865596	0.943841407	0.394865596
M700T33	0.394746825	0.943833608	0.394746825
M365T238	0.394604261	0.943824266	0.394604261
M258T64	0.394126554	0.943793101	0.394126554
M408T26	0.394054072	0.943788432	0.394054072
M339T282	0.393873294	0.943776669	0.393873294
M343T59	0.393536011	0.943754874	0.393536011
M979T185	0.393424089	0.943747664	0.393424089
M701T248	0.393357512	0.943743393	0.393357512
M355T203	0.392550158	0.94369207	0.392550158
M363T376	0.392293658	0.943675539	0.392293658
M974T42	0.392151403	0.943666818	0.392151403
M261T101	0.39212663	0.943665068	0.39212663
M449T32	0.391975609	0.943655473	0.391975609
M201T278	0.391733483	0.943640274	0.391733483
M115T72	0.391658641	0.943691625	0.391658641
M855T38	0.391617071	0.943632969	0.391617071
M659T169_1	0.391539697	0.943628124	0.391539697
M704T57	0.390993399	0.943594115	0.390993399
M524T34	0.390893126	0.943587905	0.390893126
M739T208	0.390873147	0.943586669	0.390873147
M528T313	0.390780291	0.943637952	0.390780291
M917T292_2	0.390777772	0.943580773	0.390777772
M700T60	0.390753461	0.943580289	0.390753461
M456T83	0.39069993	0.943576005	0.39069993
M490T115	0.390344879	0.94355416	0.390344879
M101T109	0.390308132	0.943551876	0.390308132
M578T348	0.390258303	0.94354882	0.390258303
M638T272	0.39025667	0.943548882	0.39025667

M331T381	0.390243831	0.943547948	0.390243831
M455T54	0.3900418	0.943535591	0.3900418
M723T352	0.389688775	0.943514244	0.389688775
M595T36	0.389629549	0.943510512	0.389629549
M734T202	0.389392941	0.943497073	0.389392941
M364T383	0.389373595	0.943495032	0.389373595
M365T409	0.389341257	0.943493086	0.389341257
M337T242	0.389135648	0.943480857	0.389135648
M252T375	0.389036646	0.943474753	0.389036646
M607T418_2	0.389010807	0.943473215	0.389010807
M1078T33	0.388830316	0.943462417	0.388830316
M322T346	0.38875177	0.943457702	0.38875177
M715T352	0.388496529	0.943442496	0.388496529
M317T35_2	0.388342904	0.943433375	0.388342904
M127T320_2	0.38828172	0.943430838	0.38828172
M185T67	0.388222051	0.943426216	0.388222051
M120T332	0.388034946	0.943415251	0.388034946
M655T194	0.387881202	0.943406112	0.387881202
M1198T352	0.387763193	0.943399231	0.387763193
M610T463	0.387763034	0.943399169	0.387763034
M289T106	0.387599236	0.943390115	0.387599236
M324T118	0.387355803	0.943375355	0.387355803
M394T115	0.387262652	0.943369941	0.387262652
M946T465_1	0.387237033	0.943369152	0.387237033
M360T102	0.387234666	0.943368312	0.387234666
M989T33	0.387017247	0.943356402	0.387017247
M672T57	0.386677395	0.943336081	0.386677395
M162T46	0.386653988	0.943334733	0.386653988
M663T120	0.386559315	0.943329307	0.386559315
M369T141	0.386415625	0.943321052	0.386415625
M311T194	0.38630822	0.943314929	0.38630822
M933T117	0.386187721	0.94330803	0.386187721
M307T214	0.385988338	0.943296686	0.385988338
M210T35	0.38591769	0.943292796	0.38591769
M1051T168	0.385848755	0.943288839	0.385848755
M107T94	0.385833564	0.94328795	0.385833564
M453T122	0.385766192	0.943284093	0.385766192
M241T66	0.385684797	0.943279493	0.385684797
M239T173	0.385076313	0.943245337	0.385076313
M1100T352	0.385029388	0.943242718	0.385029388
M420T111	0.385021606	0.943242284	0.385021606
M588T168	0.384846163	0.943232583	0.384846163
M774T171	0.384802118	0.943230153	0.384802118

M972T58	0.384199819	0.943196847	0.384199819
M268T35	0.383937516	0.943182596	0.383937516
M415T272	0.383920389	0.94318178	0.383920389
M262T497	0.383865441	0.94317857	0.383865441
M691T126	0.383713414	0.943171471	0.383713414
M362T85	0.383554149	0.943161699	0.383554149
M294T255	0.383474296	0.943157353	0.383474296
M1006T178	0.383473217	0.943157294	0.383473217
M391T55	0.383139199	0.943139312	0.383139199
M305T38	0.382819277	0.943122746	0.382819277
M221T300	0.382768626	0.943119526	0.382768626
M449T35	0.382630908	0.943112192	0.382630908
M200T257	0.382627916	0.943112032	0.382627916
M302T310	0.382474143	0.943103891	0.382474143
M479T342	0.382184195	0.9430886	0.382184195
M247T26_1	0.381609625	0.943058596	0.381609625
M135T34_2	0.38156384	0.943056221	0.38156384
M300T49	0.381402	0.943048895	0.381402
M486T94	0.381123804	0.943033522	0.381123804
M554T479_2	0.380998142	0.943027145	0.380998142
M277T54	0.380951783	0.943024718	0.380951783
M285T155	0.380878705	0.943020976	0.380878705
M746T39	0.380718151	0.943012797	0.380718151
M165T373	0.380689601	0.943011347	0.380689601
M1142T181	0.380527602	0.943004349	0.380527602
M719T54	0.380427521	0.942998069	0.380427521
M680T242	0.380312477	0.942992268	0.380312477
M1007T32	0.380237892	0.942988511	0.380237892
M253T141_2	0.38013203	0.942983215	0.38013203
M244T301	0.380017647	0.942977486	0.380017647
M395T584	0.379834676	0.942968335	0.379834676
M459T122	0.379811621	0.942967205	0.379811621
M339T194	0.379725961	0.94296293	0.379725961
M171T422	0.379657675	0.94295954	0.379657675
M608T418	0.379618398	0.942957684	0.379618398
M499T416	0.379583968	0.942955891	0.379583968
M329T125	0.379538051	0.942953618	0.379538051
M118T205	0.379520468	0.94295275	0.379520468
M418T122	0.379355527	0.942944635	0.379355527
M218T335	0.379314083	0.942942575	0.379314083
M392T105	0.37863472	0.942909446	0.37863472
M109T157	0.378547365	0.942905234	0.378547365
M605T451	0.378221373	0.942889567	0.378221373

M775T156	0.378067934	0.942882242	0.378067934
M588T38	0.378060301	0.942881888	0.378060301
M171T88	0.377552974	0.942857882	0.377552974
M301T23	0.377524342	0.942856523	0.377524342
M1189T291	0.377504998	0.942855612	0.377504998
M573T91	0.377434539	0.942852308	0.377434539
M651T95	0.377197991	0.942841293	0.377197991
M731T135	0.376925833	0.942829769	0.376925833
M294T43	0.376873113	0.942826205	0.376873113

Table S7. Divergent metabolites between Wb-T and Cb-C.

**Metabolism**	**Qst**		
	0.05%	99.50%	Estimated Qst
M319T152	0.925642051	0.996932855	0.9562893
M821T39	0.910700465	0.996157335	0.94524905
M164T32	0.90859961	0.996042522	0.943614047
M427T300	0.904957113	0.99583972	0.940725147
M536T225	0.898485317	0.995467208	0.93541618
M339T31	0.896952612	0.995376562	0.934123632
M268T580	0.896337978	0.995339955	0.933601622
M241T210	0.895289016	0.995277117	0.932705464
M339T27	0.893371974	0.995161054	0.931049774
M397T223	0.893324154	0.995158143	0.931008257
M494T169	0.892625409	0.995115398	0.930398196
M97T320	0.890292855	0.9949713	0.928341807
M534T227	0.888816423	0.994878801	0.927021229
M77T35	0.886209957	0.994713023	0.924653536
M478T175	0.883640692	0.994546402	0.922272506
M398T88	0.883160331	0.994514882	0.921821927
M395T222	0.882768493	0.994489091	0.921453216
M1176T261_2	0.882603549	0.994478211	0.921297678
M130T173	0.88137293	0.994396575	0.920130284
M312T28_2	0.881317601	0.994392886	0.920077523
M422T304	0.879477486	0.994269287	0.918309364
M298T499	0.878919307	0.99423142	0.917767407
M336T301	0.87880411	0.994223603	0.91765562
M311T28	0.878631516	0.994211854	0.917487465
M826T193	0.878433142	0.994198265	0.917292676
M454T204	0.876366599	0.994056137	0.91525806
M310T431	0.876155226	0.994041502	0.915048639
M743T81	0.876041489	0.994033549	0.914934543

M706T38	0.875660232	0.994006968	0.914553735
M672T404	0.875445884	0.993992008	0.914339489
M438T429	0.875128509	0.993969786	0.914021128
M196T272_2	0.873318751	0.993841954	0.912189142
M407T32	0.872132153	0.99375705	0.910971675
M297T28	0.868334981	0.993479477	0.906988153
M185T37	0.867733595	0.99343465	0.906344276
M667T485	0.867133185	0.993389656	0.905697853
M677T31	0.866862151	0.993369261	0.90540476
M451T428	0.866659166	0.993353945	0.905184617
M768T138	0.865092308	0.993234848	0.903472439
M125T99	0.864979125	0.993226154	0.903347303
M395T584	0.864416523	0.993182941	0.902725815
M355T203	0.863221538	0.993090347	0.901393401
M364T404	0.862710868	0.993050444	0.900818924
M391T330	0.862395174	0.993025699	0.90046269
M207T32_1	0.86189177	0.992986065	0.899891902
M511T351	0.860009643	0.992836149	0.897731561
M321T291	0.85974471	0.99281482	0.897424018
M473T236	0.85967883	0.992809511	0.89734747
M255T208	0.858780055	0.992736654	0.896296474
M906T228	0.85839967	0.992705498	0.895846352
M417T180	0.858324939	0.992699435	0.895759049
M831T540	0.857542677	0.99263526	0.8948329
M281T28_1	0.857396149	0.99262327	0.894660185
M1168T354_2	0.856897584	0.992582015	0.894064439
M1002T32	0.856790869	0.992573141	0.893936199
M359T278	0.85677497	0.992571837	0.893917439
M549T82	0.85569148	0.992481324	0.892609646
M417T28	0.855364669	0.992453824	0.892212156
M185T386	0.854923254	0.992416502	0.891672443
M150T239	0.853991524	0.99233722	0.890525753
M274T453	0.853011977	0.992252974	0.889306356
M619T108	0.852993689	0.992251399	0.88928359
M737T70	0.852980417	0.992250251	0.889266969
M420T264	0.852425003	0.992202069	0.888569209
M284T44	0.852386026	0.992198679	0.888520113
M322T131	0.851407286	0.992112992	0.887278447
M431T123	0.851112387	0.992086991	0.886901499
M970T80	0.850866268	0.992065254	0.886586425
M514T221	0.850625163	0.992043878	0.886276429
M487T345	0.85003763	0.991991538	0.885517162

M399T259	0.849851066	0.991974828	0.885274617
M612T36	0.849307911	0.991926039	0.884566529
M85T32	0.849249	0.99192071	0.884489079
M761T81	0.849063358	0.991903966	0.884246048
M118T272_2	0.848996377	0.991897909	0.884158093
M296T79	0.848502813	0.991853087	0.88350691
M313T41	0.848446687	0.991847969	0.883432521
M944T228	0.848077043	0.991814296	0.882943466
M351T358	0.847976489	0.991805095	0.882809748
M588T155	0.847927205	0.99180058	0.882744139
M234T444	0.847805188	0.991789395	0.882581564
M465T263	0.847175427	0.991731402	0.881738394
M1012T353	0.846898232	0.991705736	0.881365084
M237T206	0.846813565	0.991697882	0.881250834
M263T263	0.846744427	0.991691461	0.881157416
M220T32	0.846552256	0.991673584	0.880897309
M801T596	0.846323776	0.991652275	0.880587219
M371T272	0.845638826	0.99158804	0.879652086
M265T79	0.845440292	0.991569318	0.87937942
M495T207	0.845272518	0.991553461	0.87914845
M373T260	0.845251346	0.991551458	0.879119278
M395T260	0.845037123	0.991531156	0.878823476
M254T272	0.844836858	0.99151213	0.878546245
M358T436	0.844781806	0.991506891	0.878469881
M570T298	0.844366178	0.991467225	0.877891699
M439T429	0.844167802	0.991447938	0.877609366
M396T581	0.844011566	0.991433217	0.87739579
M83T33	0.843687242	0.991401805	0.876936918
M435T352	0.843635887	0.991397014	0.87686768
M696T212	0.843059338	0.991341125	0.876052066
M535T165	0.842693021	0.991305395	0.875530395
M115T72	0.842429708	0.991279603	0.875153677
M549T269	0.842418298	0.991278488	0.8751374
M951T228	0.842095036	0.991246687	0.874672758
M848T260	0.841822648	0.991219759	0.874279082
M680T499	0.841790145	0.991216597	0.874233064
M437T428	0.841644143	0.991202136	0.874021676
M214T385	0.841556853	0.991193477	0.873895082
M807T156	0.841311382	0.991169024	0.873537328
M347T422	0.841159067	0.991153893	0.873316231
M376T166	0.84099312	0.991137316	0.873073758
M187T335	0.840772538	0.991115222	0.872750492

M705T181	0.840603321	0.991098231	0.87250186
M295T353	0.839987558	0.991036054	0.871591556
M344T212	0.83981584	0.991018634	0.871336448
M314T454_2	0.839451157	0.990981477	0.870792056
M126T224	0.83930031	0.990966046	0.870565891
M534T366	0.839258801	0.990961429	0.870496826
M286T390	0.839235487	0.990959414	0.870468711
M570T313	0.83911585	0.990947145	0.870288859
M462T429	0.839064269	0.990941758	0.870209535
M375T28	0.838904734	0.990925449	0.869970784
M138T402	0.838853898	0.990920214	0.869894011
M555T321	0.838371635	0.990870367	0.869162853
M285T155	0.838255516	0.990858313	0.86898598
M855T429	0.838059885	0.99083796	0.868687278
M1192T45	0.837920958	0.990823471	0.868474588
M179T260	0.837831227	0.9908141	0.868337029
M310T311	0.837767181	0.990807404	0.868238706
M1057T354	0.837404679	0.990769376	0.86768022
M478T428	0.837377969	0.990766569	0.867638988
M470T122	0.837293573	0.990757629	0.86750745
M197T260	0.836991985	0.990725824	0.867040241
M698T271	0.836978584	0.990724411	0.867019481
M971T34	0.836784255	0.990703793	0.866716332
M315T454	0.836678537	0.990692585	0.866551653
M1050T352	0.836587813	0.990682944	0.866409918
M439T33	0.83658398	0.990682381	0.866401069
M1142T37	0.83653458	0.990677277	0.866326592
M903T353	0.836393763	0.990662257	0.866105699
M298T273	0.836214141	0.990643053	0.865823203
M918T32	0.836210659	0.990642505	0.865814473
M220T151	0.836194197	0.990640918	0.865791785
M989T33	0.836134676	0.990634514	0.865697464
M228T168	0.836076075	0.990628256	0.865605476
M678T31	0.835911963	0.990610607	0.865345679
M257T140	0.835774246	0.990595807	0.865127923
M314T454_1	0.835762879	0.990594576	0.865109776
M168T385	0.835090556	0.990521744	0.864037201
M307T155	0.83477294	0.99048691	0.863523253
M305T115	0.834747818	0.990484307	0.863485446
M395T366	0.834437447	0.99045026	0.862983516
M667T353	0.834354242	0.990441096	0.862848334
M654T260	0.834288981	0.990433908	0.862742344

M283T44	0.833409989	0.990336298	0.86130167
M549T595	0.833392834	0.990333924	0.861264927
M1084T352	0.833310893	0.99032521	0.861137906
M407T190	0.83306447	0.990297554	0.860729327
M173T299	0.83283759	0.990271991	0.860351536
M356T432	0.832723215	0.990259057	0.860160282
M611T353	0.832707455	0.990257306	0.860134517
M298T304	0.832315648	0.990212828	0.859476685
M373T220	0.83230229	0.990211256	0.859453238
M275T304	0.832065166	0.990184251	0.859053856
M802T265	0.832025516	0.990179707	0.858986574
M144T352	0.83197914	0.990173869	0.858898244
M263T581	0.831976087	0.99017406	0.858903043
M687T352	0.831892282	0.990164457	0.858760903
M415T428	0.831678181	0.990139293	0.858386269
M147T216	0.831657173	0.990137447	0.858361006
M571T81	0.83152717	0.990122441	0.85813867
M183T279	0.831409161	0.990108836	0.857937239
M745T82	0.831387927	0.990105974	0.857893356
M245T217	0.831360783	0.990103241	0.857854338
M831T428	0.831288789	0.990094311	0.857719873
M851T189	0.831100682	0.990073082	0.857407446
M1085T37	0.830525276	0.99000588	0.85641094
M158T258	0.83051108	0.990004213	0.856386208
M1198T352	0.830486522	0.990001331	0.856343453
M572T278	0.830026601	0.989947084	0.855538287
M497T428	0.829842939	0.989923256	0.8551771
M463T179	0.829792139	0.989918993	0.855120128
M275T439	0.829468094	0.989880623	0.854550999
M1023T172	0.829278187	0.989857872	0.854212819
M113T276	0.82913678	0.989840882	0.853960196
M629T28	0.82894265	0.989817481	0.853612138
M459T202	0.828870282	0.989808736	0.853482036
M845T352	0.82867261	0.989784794	0.853125761
M454T201	0.828630046	0.98977799	0.8530187
M675T214_2	0.828191809	0.989726181	0.852253004
M735T214	0.828077027	0.989712111	0.85204339
M209T333	0.827823688	0.989680951	0.851579007
M547T428	0.827405067	0.989629119	0.850806019
M269T104	0.827292273	0.989615092	0.850596757
M759T39_1	0.827141025	0.989596146	0.850313724
M200T158	0.827047246	0.989584498	0.850140119

M182T43	0.826851764	0.989559958	0.849773585
M420T211	0.826781508	0.989551111	0.849641395
M190T35	0.826743194	0.989546329	0.849570106
M255T429	0.82664434	0.989533861	0.849383799
M1139T354_2	0.82663498	0.989532674	0.849366052
M271T34	0.826528192	0.989519199	0.849164732
M78T207	0.826455693	0.989510027	0.84902766
M113T158	0.826405697	0.989503697	0.848933051
M980T32	0.826332017	0.989494346	0.848793244
M1071T354	0.826219959	0.989480104	0.848580279
M534T354	0.826080692	0.989461828	0.848305095
M781T39	0.82594534	0.989445061	0.848056074
M1126T36	0.825816178	0.989428524	0.847808627
M475T428	0.825522012	0.989390677	0.847242024
M652T235	0.825457181	0.989382306	0.847116647
M355T260	0.825395606	0.989374344	0.846997383
M507T290	0.825336412	0.989366679	0.846882555
M430T272_1	0.825197529	0.989348664	0.846612609
M103T256	0.82515396	0.989343001	0.846527749
M586T272	0.825105204	0.989336657	0.846432653
M290T440	0.824941166	0.989315269	0.846112003
M143T351	0.82492043	0.989312542	0.846071053
M235T272	0.824862822	0.989305026	0.845958399
M118T302	0.82481011	0.989298126	0.845854906
M196T272_1	0.824566532	0.989266142	0.845375053
M484T262	0.824452597	0.989251119	0.845149557
M291T366	0.824359551	0.98923883	0.844965081
M514T130	0.824304243	0.989231512	0.844855204
M269T39	0.824146198	0.989210553	0.844540423
M946T465_1	0.824116979	0.989206668	0.84448207
M698T210	0.82409571	0.98920384	0.844439575
M676T465_3	0.824063373	0.98919954	0.84437498
M1138T354_2	0.824056984	0.989198687	0.844362169
M131T341	0.8239351	0.989182441	0.844118048
M1059T354_2	0.823850889	0.989171194	0.843949007
M221T278	0.823467012	0.989119644	0.843173755
M1108T339_4	0.823450302	0.98911734	0.84313892
M152T260_1	0.823415884	0.989112746	0.843069975
M474T375	0.82331745	0.989099435	0.84286964
M352T272	0.823294705	0.989096365	0.84282345
M295T176	0.823280546	0.989094447	0.842794578
M255T352	0.822944769	0.989048806	0.842107333

M199T260	0.822843984	0.989034358	0.841887383
M972T34	0.822768467	0.989024704	0.841744175
M283T539	0.822722555	0.989018405	0.841649235
M166T256	0.822410922	0.988975521	0.841002646
M757T39	0.822300961	0.988960312	0.840773192
M469T272	0.822279398	0.988957321	0.840728056
M289T431	0.822240081	0.988951873	0.840645862
M739T352	0.822091425	0.98893122	0.840334132
M571T193	0.822061236	0.988927017	0.840270674
M163T55	0.82189724	0.988904053	0.839923636
M1007T32	0.821648118	0.988870544	0.839422058
M801T266	0.821615813	0.988864655	0.839328629
M210T35	0.821570883	0.988858326	0.839232964
M496T174	0.821549091	0.988855254	0.839186519
M524T192	0.821536121	0.988853425	0.839158874
M1112T36	0.821480778	0.988845614	0.839040787
M183T85	0.821265009	0.988815038	0.838578252
M1016T168	0.821101321	0.988790149	0.838196382
M751T352	0.821057652	0.988785516	0.838131453
M618T204	0.82098187	0.988774727	0.837968237
M728T195	0.820980442	0.988774524	0.837965165
M342T37	0.820908384	0.988764212	0.83780901
M1118T354_2	0.820886668	0.988760903	0.837758251
M401T211	0.820811715	0.988750352	0.837599103
M911T354	0.820704494	0.988734864	0.837364221
M369T221	0.820702718	0.988734677	0.837361615
M854T352	0.820564567	0.988714759	0.837059763
M294T244	0.82055233	0.988712995	0.837033025
M142T242	0.820414441	0.988693034	0.8367304
M251T169	0.820412727	0.988692785	0.836726616
M262T427	0.820281983	0.988673794	0.836438589
M120T256	0.820274374	0.988672683	0.836421707
M1061T352	0.820271703	0.988672286	0.836415669
M974T34	0.820201452	0.988662065	0.836260642
M118T272_1	0.819886234	0.988615906	0.835559889
M661T36	0.819865376	0.988612852	0.83551354
M293T321	0.819864167	0.988612675	0.83551085
M830T479	0.819767466	0.988598413	0.835294139
M174T335	0.819753743	0.988596382	0.835263261
M823T214	0.819726993	0.988592462	0.83520379
M372T503	0.819595629	0.988573037	0.834908584
M669T198	0.819494366	0.988558019	0.834680267

M804T100	0.819434295	0.988549089	0.834544482
M945T465_2	0.819418627	0.988559444	0.834742346
M515T213	0.819405049	0.988544738	0.834478307
M848T352	0.81931052	0.988530644	0.834263911
M224T671	0.819300223	0.988529107	0.834240542
M563T213	0.819288055	0.988527264	0.834212402
M282T129	0.819207991	0.988515323	0.834030811
M293T540_2	0.819183125	0.98851107	0.833964405
M282T260	0.819075342	0.98849512	0.833722304
M1104T37	0.819023862	0.988487696	0.833610266
M434T429	0.818737296	0.988444376	0.832950223
M111T159	0.818694797	0.988437953	0.832852401
M291T335_1	0.818667667	0.988433851	0.83278993
M140T381	0.818586243	0.988421381	0.832599545
M275T399	0.818583695	0.988421084	0.832595301
M1067T36	0.818463347	0.988402729	0.832315388
M1188T172	0.818290148	0.988376201	0.831910618
M452T201	0.818188675	0.988360605	0.831672576
M550T153	0.81813069	0.988351661	0.831535989
M1099T37	0.818065901	0.988341671	0.831383473
M143T153	0.818004638	0.988332197	0.831238755
M248T427	0.81792667	0.988320116	0.831054181
M605T203	0.817910251	0.988317569	0.831015271
M325T209	0.81789941	0.988315299	0.830978765
M949T464	0.817781657	0.988297561	0.830709436
M263T366	0.817769256	0.988295608	0.83067951
M928T209	0.81765425	0.988277687	0.830405586
M170T157	0.817578443	0.988265799	0.830223686
M1090T354_2	0.817373829	0.988233661	0.829731959
M369T203	0.81727819	0.988217943	0.829489399
M888T352	0.817133735	0.988195672	0.829150189
M1176T261_1	0.817123465	0.98819404	0.829125177
M392T185	0.816978458	0.988169682	0.828747977
M630T436	0.816894215	0.98815719	0.828559421
M536T100	0.816829599	0.988147148	0.82840637
M141T334	0.816775315	0.988138438	0.828272765
M176T272	0.816724945	0.988130347	0.828148643
M831T250	0.816706714	0.98812739	0.828103184
M981T159	0.816622681	0.988113876	0.827895871
M973T34	0.816592805	0.988109061	0.827821995
M744T81	0.816551584	0.988102395	0.827719653
M830T196	0.816367055	0.988072477	0.827260177

M309T255	0.816243647	0.988052369	0.826951205
M192T385	0.816214893	0.988047641	0.826878439
M417T429	0.815908607	0.987997376	0.826105451
M471T34_2	0.815886048	0.987993648	0.826048063
M731T354	0.815840836	0.987986164	0.825932835
M396T366	0.815819097	0.987982582	0.825877749
M551T314	0.815798744	0.987979177	0.825825214
M242T238	0.815724096	0.987966829	0.825635186
M554T196	0.81568413	0.987960079	0.825530892
M206T157	0.81562998	0.987951186	0.825394265
M115T242	0.81544637	0.987920608	0.824923326
M589T345	0.815421054	0.987916265	0.824856076
M258T452	0.815416046	0.987915426	0.824843137
M123T299	0.814908725	0.987829399	0.823514867
M945T465_1	0.814802921	0.987811494	0.823238732
M567T260	0.814795047	0.987810137	0.823217738
M193T354	0.814727007	0.987798475	0.823037494
M243T159	0.814564585	0.987770471	0.822604302
M832T333	0.814366217	0.987735815	0.822067143
M742T149	0.814275931	0.987720058	0.821823052
M837T211	0.814101002	0.98768969	0.821353238
M291T303_1	0.814073221	0.987684843	0.821278213
M964T352	0.814058551	0.987701556	0.82159303
M291T256	0.813930686	0.987659709	0.820888423
M1105T37	0.813909222	0.987655837	0.820828126
M100T705	0.813823103	0.987640638	0.820592453
M475T181	0.813632245	0.987606642	0.820064623
M173T423	0.813589864	0.987599058	0.819946791
M1122T354	0.813563749	0.987594378	0.819874074
M386T155	0.813479703	0.987579285	0.819639514
M662T200	0.813372326	0.987559929	0.819338536
M261T123	0.813341949	0.987554438	0.819253131
M786T147	0.813341143	0.987554277	0.819250594
M1138T352_2	0.813277531	0.98754262	0.81906884
M680T200	0.813180569	0.987525158	0.818797504
M831T479_1	0.813057682	0.987502707	0.818447843
M677T201	0.812990687	0.987490465	0.818257216
M843T272	0.812812334	0.987457647	0.8177457
M375T279	0.812753028	0.987446686	0.817574776
M312T134	0.812648247	0.987427007	0.81726708
M284T260	0.812507069	0.987400919	0.816860491
M466T169_2	0.812473113	0.987394562	0.816761192

M150T294	0.812465969	0.987393225	0.816740329
M696T283	0.8124524	0.987390678	0.816700521
M67T32	0.812401753	0.987381177	0.816552106
M305T272	0.812361592	0.987373623	0.816434045
M401T231	0.812354347	0.987372263	0.816412817
M542T152	0.812339103	0.987368194	0.816345896
M1107T339_3	0.812318577	0.987365526	0.816307526
M945T464	0.8121083	0.987325698	0.815684601
M285T381	0.812088661	0.98732196	0.815626116
M110T260	0.812047918	0.987314196	0.815504591
M342T333	0.812014754	0.987307864	0.815405458
M1160T352	0.81190865	0.987287546	0.815087292
M374T211	0.811867713	0.987279687	0.814964184
M863T429	0.811819605	0.987270427	0.814819072
M221T33	0.811786381	0.987264019	0.814718638
M395T263	0.811785617	0.987263873	0.814716346
M703T272	0.811764666	0.987259826	0.814652904
M496T196	0.811695496	0.98724631	0.814440622
M927T199	0.811477209	0.987203945	0.813776239
M256T80	0.811475414	0.987203581	0.8137705
M1159T352_1	0.811449136	0.987198413	0.813689297
M491T272	0.811404643	0.987189713	0.813552722
M220T26	0.811333839	0.987175801	0.813334203
M446T171	0.811237451	0.98715677	0.813035097
M851T78	0.811139409	0.987137327	0.812729359
M840T272_1	0.810922104	0.987093905	0.812045923
M203T381	0.810811541	0.987071635	0.811695085
M333T421	0.810802119	0.987069733	0.811665123
M1044T352	0.81067428	0.987043825	0.811256663
M849T352	0.810620402	0.987032835	0.811083259
M1070T354_1	0.810594516	0.987027378	0.810996688
M393T270	0.810557496	0.987020008	0.810880896
M262T157	0.810420843	0.986991965	0.810438136
M687T353	0.810391951	0.987004905	0.810691868
M427T221	0.810302205	0.98698336	0.810302205
M457T204	0.810065204	0.986968363	0.810065204
M1115T37	0.809806359	0.98695199	0.809806359
M1001T352	0.809798776	0.986951511	0.809798776
M357T175	0.809756795	0.986948857	0.809756795
M205T35	0.809667881	0.986943235	0.809667881
M757T351	0.809651689	0.986942232	0.809651689
M987T352	0.809519407	0.986933848	0.809519407

M816T32	0.808832508	0.986890464	0.808832508
M193T311	0.808794098	0.986888041	0.808794098
M739T78	0.808745977	0.986885065	0.808745977
M291T352	0.808672861	0.986880388	0.808672861
M461T429	0.808547655	0.986872489	0.808547655
M263T27	0.808488393	0.986868751	0.808488393
M473T228_2	0.808044562	0.986840767	0.808044562
M461T211	0.80668563	0.98675523	0.80668563
M372T272_1	0.806606443	0.986750258	0.806606443
M182T299	0.806260936	0.986728545	0.806260936
M496T33	0.806233246	0.986722839	0.806233246
M539T153	0.805821247	0.98670094	0.805821247
M871T429	0.805568411	0.986685082	0.805568411
M620T209	0.805567362	0.986685011	0.805567362
M279T262	0.805412571	0.986675338	0.805412571
M955T32	0.805408762	0.986675078	0.805408762
M299T41	0.805300088	0.986668251	0.805300088
M147T299	0.804969159	0.986647536	0.804969159
M732T35	0.804933823	0.986645299	0.804933823
M357T423	0.804706149	0.98663104	0.804706149
M158T179	0.804437002	0.986614252	0.804437002
M601T170	0.804045217	0.986589682	0.804045217
M487T169	0.803495539	0.986555328	0.803495539
M386T393	0.803403611	0.986549587	0.803403611
M444T170	0.803394717	0.986549031	0.803394717
M455T429	0.803092246	0.986530295	0.803092246
M371T345	0.802938922	0.98652058	0.802938922
M503T171	0.802751112	0.986508864	0.802751112
M759T118	0.80259177	0.986498928	0.80259177
M780T114	0.802371652	0.986485208	0.802371652
M278T256	0.802060141	0.9864658	0.802060141
M769T169	0.801948739	0.986458865	0.801948739
M1192T153	0.801701308	0.986443461	0.801701308
M771T47	0.801655052	0.986440589	0.801655052
M910T354	0.801466405	0.986428845	0.801466405
M489T272_1	0.801314656	0.986419408	0.801314656
M551T267	0.801033551	0.986401933	0.801033551
M772T216	0.80089871	0.986393559	0.80089871
M1055T354	0.800673083	0.986379581	0.800673083
M171T87	0.800578049	0.986373638	0.800578049
M650T32	0.800507281	0.986369293	0.800507281
M445T325	0.800487379	0.98636801	0.800487379

M1192T171	0.80025743	0.986353741	0.80025743
M412T41	0.799929665	0.986333411	0.799929665
M295T334	0.799929074	0.986333418	0.799929074
M663T354	0.799869995	0.986329711	0.799869995
M387T204	0.799749878	0.986322266	0.799749878
M149T257	0.799717575	0.986320264	0.799717575
M365T116	0.79968038	0.986317958	0.79968038
M757T160	0.799361838	0.986298226	0.799361838
M545T18	0.799334014	0.986296503	0.799334014
M390T260	0.799249318	0.986291259	0.799249318
M413T358	0.799078851	0.98628071	0.799078851
M263T428	0.799073267	0.986280426	0.799073267
M694T213	0.798979305	0.986274685	0.798979305
M483T154	0.798937774	0.986271978	0.798937774
M548T272	0.798533705	0.986246989	0.798533705
M109T238	0.798505205	0.986245227	0.798505205
M459T260	0.798479749	0.98624366	0.798479749
M180T56	0.798454488	0.986242099	0.798454488
M183T90	0.798160259	0.986223914	0.798160259
M207T199	0.797772799	0.986199993	0.797772799
M293T328	0.797591161	0.986188787	0.797591161
M239T187	0.797382454	0.986175914	0.797382454
M993T79	0.797367885	0.986175016	0.797367885
M342T58	0.79707213	0.986156786	0.79707213
M803T169	0.796803402	0.986140235	0.796803402
M472T228	0.796535502	0.986123787	0.796535502
M1107T339_1	0.79633221	0.986111234	0.79633221
M423T32_2	0.796294321	0.986108902	0.796294321
M271T212	0.796159903	0.986100636	0.796159903
M933T57	0.796146714	0.986099825	0.796146714
M1095T37	0.795966617	0.986088752	0.795966617
M281T300	0.795935493	0.986086843	0.795935493
M723T207	0.795878855	0.986083358	0.795878855
M243T284	0.79577921	0.986077236	0.79577921
M613T206	0.795549437	0.986063123	0.795549437
M194T366	0.795146676	0.986038402	0.795146676
M199T180	0.794991078	0.986028857	0.794991078
M992T353	0.794959764	0.986026943	0.794959764
M1036T172	0.794833255	0.98601918	0.794833255
M588T202	0.794590378	0.986004314	0.794590378
M303T158	0.794338042	0.985988838	0.794338042
M188T272_1	0.794295623	0.985986249	0.794295623

M279T159	0.794260928	0.985984116	0.794260928
M445T373	0.794106382	0.985974695	0.794106382
M554T479_1	0.794008932	0.985968691	0.794008932
M551T305	0.793486564	0.98593675	0.793486564
M668T271	0.793374647	0.985929909	0.793374647
M217T577	0.793037721	0.985909339	0.793037721
M850T429	0.793033874	0.985909095	0.793033874
M191T585	0.792848793	0.985898275	0.792848793
M374T272	0.792515899	0.98587749	0.792515899
M191T34_1	0.792240654	0.985860713	0.792240654
M520T169_2	0.792167053	0.985856229	0.792167053
M827T68	0.792083645	0.985851154	0.792083645
M565T260	0.791822165	0.985835232	0.791822165
M795T54	0.791621163	0.985822989	0.791621163
M314T33	0.791514445	0.985816561	0.791514445
M801T330	0.791307718	0.985803924	0.791307718
M104T437	0.791158006	0.985794826	0.791158006
M273T197	0.791118809	0.985792443	0.791118809
M277T303_1	0.791095884	0.985791047	0.791095884
M157T352_2	0.790875628	0.985777668	0.790875628
M255T442	0.790791656	0.985772566	0.790791656
M611T171	0.790721655	0.985768515	0.790721655
M410T209	0.790576204	0.985759486	0.790576204
M557T428	0.790230601	0.985738794	0.790230601
M823T102	0.789941073	0.985720966	0.789941073
M313T28_1	0.789902863	0.985718652	0.789902863
M753T175	0.789781561	0.985711436	0.789781561
M169T35_2	0.789482792	0.985693214	0.789482792
M664T272_1	0.789270305	0.98568035	0.789270305
M117T242	0.789229629	0.985677889	0.789229629
M888T112	0.789181626	0.98567498	0.789181626
M485T373	0.789032911	0.985665994	0.789032911
M273T262	0.789005257	0.985664324	0.789005257
M252T27_2	0.788885848	0.985657101	0.788885848
M86T262	0.788729379	0.985647645	0.788729379
M1004T170	0.788664511	0.985643726	0.788664511
M313T378	0.788640854	0.985642297	0.788640854
M739T81	0.78860097	0.98563989	0.78860097
M366T260	0.788551436	0.985636896	0.788551436
M865T175	0.788356441	0.985625123	0.788356441
M409T352	0.788253452	0.985618906	0.788253452
M1094T35	0.788159889	0.985613261	0.788159889

M741T38	0.787981524	0.985602502	0.787981524
M338T354	0.787902868	0.985597759	0.787902868
M471T36	0.787859791	0.985595162	0.787859791
M481T329	0.787776882	0.985590163	0.787776882
M1188T354_2	0.787747585	0.985588397	0.787747585
M554T479_2	0.787019201	0.985544538	0.787019201
M946T352	0.787013813	0.985544212	0.787013813
M849T158	0.786958062	0.98554086	0.786958062
M785T352_1	0.786743842	0.985527982	0.786743842
M112T237	0.786675248	0.985524236	0.786675248
M197T298	0.786649151	0.985522283	0.786649151
M1024T353	0.786387098	0.98550658	0.786387098
M136T299	0.786360716	0.985504954	0.786360716
M1018T33	0.786257651	0.985498769	0.786257651
M1138T354_1	0.785858358	0.985474802	0.785858358
M291T541	0.785844252	0.98547403	0.785844252
M152T260_2	0.785614673	0.985460191	0.785614673
M268T262	0.785566336	0.985457293	0.785566336
M276T263_2	0.785547843	0.985456188	0.785547843
M566T248	0.78541615	0.985448303	0.78541615
M884T352	0.785206003	0.985435712	0.785206003
M195T298	0.784985632	0.985422513	0.784985632
M335T253	0.784829855	0.985413193	0.784829855
M608T171	0.784825341	0.985412924	0.784825341
M508T242	0.784789643	0.985410787	0.784789643
M136T158	0.784763684	0.985409235	0.784763684
M269T47	0.784709417	0.985405994	0.784709417
M213T104_1	0.784670403	0.985403656	0.784670403
M319T333	0.784543871	0.985396091	0.784543871
M267T250	0.784443652	0.985390101	0.784443652
M680T242	0.783905741	0.985357979	0.783905741
M234T272	0.783894633	0.985357315	0.783894633
M605T272	0.783779412	0.98535044	0.783779412
M221T111	0.783713974	0.985346545	0.783713974
M763T197	0.78361536	0.98534066	0.78361536
M638T272	0.783581716	0.985338648	0.783581716
M277T263	0.783565995	0.985337763	0.783565995
M834T353	0.783561439	0.985337439	0.783561439
M114T366	0.783495214	0.985333492	0.783495214
M546T130	0.783363837	0.985325663	0.783363837
M1067T35	0.783355271	0.985325153	0.783355271
M627T354	0.783129191	0.985311683	0.783129191

M957T34	0.78295026	0.98530103	0.78295026
M624T451	0.782803413	0.985292292	0.782803413
M557T304	0.782702792	0.985286305	0.782702792
M1069T36	0.782543418	0.985276827	0.782543418
M862T354	0.782327228	0.985263976	0.782327228
M758T39_1	0.782178994	0.985255169	0.782178994
M488T272	0.782062086	0.985248226	0.782062086
M527T272	0.781773013	0.985231067	0.781773013
M255T210	0.781653457	0.985224032	0.781653457
M1187T352	0.781513558	0.985215912	0.781513558
M737T112	0.781453398	0.985212111	0.781453398
M827T157	0.781133496	0.985193195	0.781133496
M239T257	0.781038481	0.985187528	0.781038481
M577T291	0.780871162	0.985177629	0.780871162
M193T267	0.78063293	0.985163527	0.78063293
M282T294	0.780509696	0.985156243	0.780509696
M481T40	0.780451897	0.985152822	0.780451897
M258T63	0.7803013	0.985143945	0.7803013
M206T183	0.780082187	0.985130983	0.780082187
M647T352	0.78003299	0.985128072	0.78003299
M736T26_1	0.780025422	0.985127658	0.780025422
M625T33	0.779926511	0.985121787	0.779926511
M394T183	0.779924822	0.985121814	0.779924822
M161T370	0.779779095	0.985113085	0.779779095
M256T253	0.779729612	0.985110165	0.779729612
M337T105	0.779595497	0.98510226	0.779595497
M699T202	0.779577417	0.985101188	0.779577417
M292T540_1	0.779318458	0.985085947	0.779318458
M1111T352	0.779148905	0.985075931	0.779148905
M589T201	0.779037061	0.985069434	0.779037061
M529T354	0.778805626	0.985055722	0.778805626
M549T352	0.778546394	0.985040472	0.778546394
M736T151	0.778169233	0.985018307	0.778169233
M409T270	0.778000004	0.98500837	0.778000004
M1034T174	0.777927549	0.985004192	0.777927549
M146T267	0.777676991	0.984989414	0.777676991
M551T364	0.777617928	0.984985951	0.777617928
M883T352_1	0.777513131	0.984979819	0.777513131
M711T214	0.777479454	0.98497783	0.777479454
M757T149	0.777344456	0.984969919	0.777344456
M843T352	0.777317558	0.98496839	0.777317558
M1019T39	0.777194583	0.984961138	0.777194583

M785T272	0.777057689	0.984953124	0.777057689
M303T218	0.776698936	0.984932168	0.776698936
M991T170	0.776613961	0.984927575	0.776613961
M465T154	0.77660607	0.984926698	0.77660607
M653T129	0.77640818	0.98491513	0.77640818
M1177T352	0.776330634	0.984910598	0.776330634
M244T238	0.776117132	0.98489813	0.776117132
M520T193	0.776033243	0.984893232	0.776033243
M750T352	0.775943258	0.984888167	0.775943258
M307T116	0.775733439	0.98487574	0.775733439
M720T26_2	0.77539009	0.984855724	0.77539009
M276T303	0.775285874	0.984849654	0.775285874
M241T285	0.775270605	0.984848766	0.775270605
M546T39	0.775211534	0.984845324	0.775211534
M664T272_2	0.775126528	0.984840377	0.775126528
M619T347	0.775077029	0.984837494	0.775077029
M119T157	0.774954117	0.98483034	0.774954117
M146T385	0.77489685	0.984827009	0.77489685
M507T210	0.774819053	0.984822548	0.774819053
M559T181	0.77447466	0.984802463	0.77447466
M1089T352_1	0.774440898	0.984800504	0.774440898
M722T272	0.774392523	0.984797694	0.774392523
M276T452	0.774313004	0.984802706	0.774313004
M307T203	0.77429777	0.984792189	0.77429777
M314T467	0.774293844	0.984791959	0.774293844
M62T13	0.77410163	0.984780802	0.77410163
M1164T46	0.773417936	0.984741164	0.773417936
M779T40	0.773189152	0.984727919	0.773189152
M946T465_2	0.77260961	0.984694391	0.77260961
M547T272_1	0.772553867	0.984691299	0.772553867
M292T335_1	0.77253039	0.984689832	0.77253039
M625T354	0.771853084	0.984650755	0.771853084
M723T45	0.771576374	0.984634814	0.771576374
M823T354	0.771538991	0.984632661	0.771538991
M305T181	0.771489379	0.984629805	0.771489379
M205T300	0.771480664	0.9846293	0.771480664
M1128T352	0.771361728	0.984622459	0.771361728
M275T581	0.771312033	0.984619599	0.771312033
M265T294	0.77102532	0.984603113	0.77102532
M248T444	0.770962995	0.984599527	0.770962995
M201T190	0.770821658	0.984591406	0.770821658
M333T35	0.770384159	0.984566289	0.770384159

M999T37	0.770321184	0.984562678	0.770321184
M788T465_1	0.770267322	0.984559597	0.770267322
M155T561	0.770253703	0.98455885	0.770253703
M971T354_1	0.77020066	0.984555766	0.77020066
M745T352	0.76993707	0.98454066	0.76993707
M532T41	0.769803383	0.984533006	0.769803383
M335T423	0.769530687	0.9845174	0.769530687
M173T100	0.769487149	0.984514907	0.769487149
M656T171	0.769331162	0.984505985	0.769331162
M581T266	0.769266841	0.984502308	0.769266841
M781T116	0.769231738	0.984500302	0.769231738
M530T32	0.769198592	0.984498409	0.769198592
M577T310	0.769143495	0.984495453	0.769143495
M718T38	0.76907277	0.984491233	0.76907277
M606T272_1	0.768708933	0.984470447	0.768708933
M603T170	0.768488129	0.984457852	0.768488129
M720T33	0.768357123	0.984450385	0.768357123
M700T60	0.768173569	0.984439927	0.768173569
M357T544	0.768016176	0.984431042	0.768016176
M887T355	0.767600229	0.984407304	0.767600229
M499T170	0.767395645	0.984395922	0.767395645
M864T67	0.767220673	0.984385743	0.767220673
M923T228	0.76711324	0.984379642	0.76711324
M487T238	0.767080989	0.984377815	0.767080989
M269T56	0.76698173	0.98437218	0.76698173
M609T171	0.766947003	0.984370225	0.766947003
M333T354	0.766401174	0.984339276	0.766401174
M284T34_2	0.766348241	0.98433628	0.766348241
M730T354	0.766252207	0.984330844	0.766252207
M334T28	0.765984528	0.984315704	0.765984528
M550T171	0.765772501	0.98430372	0.765772501
M368T195	0.765562631	0.984291867	0.765562631
M625T128	0.765532986	0.984290191	0.765532986
M1149T39	0.765357574	0.984280294	0.765357574
M1066T37	0.765342907	0.984279467	0.765342907
M507T352	0.764962399	0.984258013	0.764962399
M1117T354	0.764653337	0.98424061	0.764653337
M244T254	0.764603537	0.984237809	0.764603537
M277T320	0.764579275	0.984236538	0.764579275
M613T235	0.764545591	0.984234547	0.764545591
M864T340	0.764389844	0.984225841	0.764389844
M480T196	0.764346632	0.984223364	0.764346632

M712T190	0.764073999	0.984208044	0.764073999
M556T171	0.764050916	0.984206742	0.764050916
M431T354	0.764037367	0.98420598	0.764037367
M1168T354_1	0.76376591	0.98419075	0.76376591
M947T37	0.763590774	0.984180919	0.763590774
M581T364	0.763572337	0.984179886	0.763572337
M205T327	0.763569258	0.984179713	0.763569258
M573T39	0.763432459	0.984172044	0.763432459
M268T303	0.763201646	0.984159116	0.763201646
M177T242	0.763156252	0.984156573	0.763156252
M395T335	0.763075142	0.984152034	0.763075142
M551T401	0.762991681	0.984147363	0.762991681
M506T298	0.762903824	0.984142447	0.762903824
M358T483_1	0.762821699	0.984137855	0.762821699
M202T272_1	0.762525422	0.984121295	0.762525422
M762T490	0.762216333	0.984104038	0.762216333
M593T463	0.761879501	0.984085348	0.761879501
M507T156	0.76178588	0.984080087	0.76178588
M353T213	0.761736491	0.984077288	0.761736491
M1197T354	0.761594852	0.984069419	0.761594852
M451T45	0.761526217	0.984065578	0.761526217
M567T247	0.761161006	0.984045264	0.761161006
M781T148	0.761055815	0.98403971	0.761055815
M156T343	0.760948883	0.984033478	0.760948883
M593T208	0.760876464	0.984029455	0.760876464
M325T378	0.760835399	0.984027177	0.760835399
M1163T354	0.760819999	0.984026346	0.760819999
M945T465_3	0.760744919	0.984022195	0.760744919
M952T33	0.760525366	0.984009972	0.760525366
M436T328	0.760123229	0.983987755	0.760123229
M485T27	0.759966201	0.983978994	0.759966201
M322T256	0.759933683	0.983977404	0.759933683
M930T176	0.759833925	0.983971675	0.759833925
M478T200	0.75972355	0.983965639	0.75972355
M391T43	0.759664208	0.983962293	0.759664208
M730T56	0.759662434	0.983962194	0.759662434
M349T38	0.759554084	0.983956205	0.759554084
M624T272	0.759407737	0.983948119	0.759407737
M128T386	0.759350567	0.983944963	0.759350567
M1090T352_1	0.75909281	0.983930768	0.75909281
M364T383	0.758983871	0.98392473	0.758983871
M235T354	0.758814927	0.983915424	0.758814927

M204T253	0.758780513	0.983913587	0.758780513
M981T354	0.758718997	0.983910132	0.758718997
M291T335_2	0.75865115	0.983906395	0.75865115
M764T198	0.758649985	0.983906394	0.758649985
M153T238	0.758591436	0.983903106	0.758591436
M1137T354	0.758462591	0.98389639	0.758462591
M725T272	0.758376158	0.983891264	0.758376158
M254T48	0.758346622	0.983889634	0.758346622
M725T354	0.758124146	0.983877402	0.758124146
M567T248	0.757726355	0.983855556	0.757726355
M1169T354	0.757483543	0.983842245	0.757483543
M154T42	0.757290417	0.983832083	0.757290417
M342T236	0.756964878	0.98381383	0.756964878
M960T272	0.756191061	0.983771552	0.756191061
M395T351	0.756029416	0.983762738	0.756029416
M549T91	0.756019182	0.983762179	0.756019182
M277T435	0.755753432	0.983747698	0.755753432
M1198T154	0.755468444	0.983732188	0.755468444
M181T402	0.755330269	0.983724674	0.755330269
M220T56	0.755258157	0.983720854	0.755258157
M1137T352	0.755203829	0.983717806	0.755203829
M208T256	0.754942769	0.983703623	0.754942769
M948T37_2	0.75483789	0.983697931	0.75483789
M393T46	0.754783116	0.983694968	0.754783116
M463T269	0.754738971	0.983692565	0.754738971
M471T124	0.754119361	0.983659006	0.754119361
M364T60	0.753944842	0.98364956	0.753944842
M379T292	0.753881329	0.983646126	0.753881329
M609T352	0.753815042	0.983642603	0.753815042
M145T381_1	0.753634443	0.983632834	0.753634443
M275T453	0.753618467	0.983632534	0.753618467
M465T36	0.753545562	0.983627991	0.753545562
M393T350	0.75348435	0.983624686	0.75348435
M375T253	0.753442093	0.983622411	0.753442093
M455T54	0.75338037	0.983619078	0.75338037
M769T243	0.752974628	0.983597216	0.752974628
M263T335	0.752818044	0.983588778	0.752818044
M195T266	0.752738457	0.983584497	0.752738457
M121T36	0.752732126	0.983584154	0.752732126
M1091T354	0.752123192	0.983551436	0.752123192
M250T408	0.751772113	0.98353261	0.751772113
M805T156	0.751409652	0.983513203	0.751409652

M697T18	0.751398427	0.983512609	0.751398427
M477T54	0.751307482	0.983507737	0.751307482
M185T151	0.750626318	0.983471575	0.750626318
M497T40	0.750457674	0.983462383	0.750457674
M507T272	0.749838113	0.983429461	0.749838113
M757T349	0.749779044	0.983426265	0.749779044
M451T352	0.749497495	0.983431674	0.749497495
M171T36	0.749307593	0.983401246	0.749307593
M329T34	0.74925484	0.98339844	0.74925484
M233T408	0.749109559	0.983390738	0.749109559
M273T242	0.749085756	0.983389481	0.749085756
M328T33	0.749072049	0.983388753	0.749072049
M555T479_1	0.749054553	0.983387824	0.749054553
M482T157	0.74888092	0.983378629	0.74888092
M1129T36	0.748859824	0.983377514	0.748859824
M985T35	0.74882046	0.983375432	0.74882046
M433T136	0.74867186	0.983367732	0.74867186
M660T170	0.748640392	0.983365906	0.748640392
M364T134	0.748630859	0.983365429	0.748630859
M341T49	0.748571456	0.983362462	0.748571456
M275T38	0.748546315	0.983360934	0.748546315
M532T109	0.748525861	0.98335999	0.748525861
M964T228	0.748414623	0.983353976	0.748414623
M539T32	0.747913061	0.983327512	0.747913061
M291T143	0.747789745	0.983321018	0.747789745
M367T50	0.747615247	0.983311827	0.747615247
M791T353	0.74758914	0.983310454	0.74758914
M1159T352_2	0.747488854	0.983305432	0.747488854
M302T193	0.74747484	0.983304441	0.74747484
M615T463	0.747331443	0.983296897	0.747331443
M1187T353	0.747053997	0.98328278	0.747053997
M276T335	0.747033585	0.983281274	0.747033585
M976T155	0.746830435	0.983270595	0.746830435
M653T213	0.746706555	0.983264096	0.746706555
M207T38	0.746491171	0.983252811	0.746491171
M379T204	0.746315816	0.983243636	0.746315816
M451T256	0.74626547	0.983240997	0.74626547
M413T297	0.746051628	0.98322982	0.746051628
M726T197	0.7460119	0.983227739	0.7460119
M300T321	0.745687507	0.983210921	0.745687507
M532T92	0.745558775	0.983204403	0.745558775
M282T92	0.745193719	0.983185158	0.745193719

M981T353	0.744952906	0.983172538	0.744952906
M275T334	0.744598807	0.983154134	0.744598807
M633T354	0.74438431	0.983143006	0.74438431
M658T171	0.744274514	0.983137553	0.744274514
M794T352	0.74418914	0.983132886	0.74418914
M278T142	0.744162501	0.983131515	0.744162501
M512T36	0.744036003	0.983125237	0.744036003
M488T28	0.743908653	0.983118366	0.743908653
M148T386	0.74389896	0.983117862	0.74389896
M967T354	0.743542926	0.983099452	0.743542926
M828T198	0.743248074	0.983084232	0.743248074
M210T334	0.743238838	0.983083763	0.743238838
M244T213	0.742967448	0.983069766	0.742967448
M315T35_1	0.742919587	0.983067296	0.742919587
M746T39	0.742630726	0.983052633	0.742630726
M608T272	0.742583785	0.983050015	0.742583785
M339T337	0.742529516	0.983047223	0.742529516
M646T79	0.742473069	0.983044291	0.742473069
M366T383	0.742218072	0.983031224	0.742218072
M625T32	0.742019449	0.983021031	0.742019449
M335T268	0.741840806	0.983011877	0.741840806
M277T335_1	0.741799102	0.983009737	0.741799102
M269T213	0.7417807	0.983008808	0.7417807
M787T465	0.741534487	0.982996201	0.741534487
M485T264	0.741450191	0.982991877	0.741450191
M360T259_1	0.741438809	0.982991475	0.741438809
M415T36	0.741118475	0.982974925	0.741118475
M641T57	0.741032989	0.982970564	0.741032989
M1188T354_1	0.740918627	0.982964726	0.740918627
M265T299	0.740848175	0.982961132	0.740848175
M319T353	0.740791153	0.982958482	0.740791153
M359T34	0.740708084	0.982954054	0.740708084
M247T262	0.740622726	0.982949643	0.740622726
M697T212	0.740561045	0.9829465	0.740561045
M941T352	0.74033468	0.982934981	0.74033468
M437T299	0.740244035	0.982930371	0.740244035
M402T211	0.739930515	0.982914443	0.739930515
M709T354	0.73990104	0.982912949	0.73990104
M263T34_1	0.739835823	0.982909638	0.739835823
M853T38	0.739781099	0.98290686	0.739781099
M113T27_2	0.739738484	0.982904702	0.739738484
M766T196	0.739691142	0.9829023	0.739691142

M609T405	0.738899502	0.98286224	0.738899502
M565T353	0.738850756	0.98285978	0.738850756
M141T34	0.738820193	0.982858287	0.738820193
M576T34	0.738816178	0.982858036	0.738816178
M711T170	0.738799426	0.982857188	0.738799426
M423T212	0.738524878	0.98284334	0.738524878
M572T193	0.738338708	0.982833961	0.738338708
M835T71	0.738338342	0.982833942	0.738338342
M337T244	0.738144601	0.982824192	0.738144601
M1175T261_2	0.738055795	0.982819725	0.738055795
M583T299	0.737944268	0.982814335	0.737944268
M967T228	0.737851129	0.98280944	0.737851129
M757T118	0.737828275	0.982808292	0.737828275
M981T81	0.737678465	0.982800769	0.737678465
M239T254	0.73747258	0.982790443	0.73747258
M547T168	0.737412964	0.982787453	0.737412964
M209T225	0.737262872	0.982779937	0.737262872
M395T303	0.737123811	0.982772986	0.737123811
M417T32	0.736866064	0.982760085	0.736866064
M715T112	0.736674502	0.982750517	0.736674502
M456T352	0.73652498	0.982743104	0.73652498
M428T513	0.736343245	0.982734449	0.736343245
M421T311	0.735990492	0.982716434	0.735990492
M361T280	0.735329673	0.982683665	0.735329673
M585T352	0.735191633	0.982676774	0.735191633
M1034T34	0.735152585	0.982674842	0.735152585
M939T352	0.735037592	0.982669171	0.735037592
M258T376	0.734990692	0.982666829	0.734990692
M160T242	0.734850706	0.982659903	0.734850706
M531T320	0.734812851	0.982658032	0.734812851
M227T212	0.73464213	0.982649598	0.73464213
M353T352	0.734587944	0.982646931	0.734587944
M425T581	0.734319981	0.982633702	0.734319981
M307T33	0.734269167	0.982631269	0.734269167
M680T175	0.734183098	0.982626956	0.734183098
M421T263	0.73410861	0.982623288	0.73410861
M337T35_1	0.734049317	0.9826332	0.734049317
M118T257	0.733818707	0.982609024	0.733818707
M305T50	0.733757739	0.982606026	0.733757739
M183T357	0.733733236	0.982604824	0.733733236
M1083T36	0.733587402	0.982597666	0.733587402
M1189T291	0.733545467	0.982595602	0.733545467

M430T272_2	0.733324023	0.98258477	0.733324023
M1019T354	0.733248739	0.982581046	0.733248739
M569T122	0.732991896	0.982568467	0.732991896
M317T373	0.732979765	0.982568133	0.732979765
M551T342	0.732921121	0.982565004	0.732921121
M271T33	0.73283406	0.982560745	0.73283406
M223T59	0.732619162	0.982550244	0.732619162
M1000T35	0.732544474	0.982546597	0.732544474
M613T47	0.732036187	0.98252182	0.732036187
M432T204	0.731926405	0.982516552	0.731926405
M213T35	0.731596518	0.982500443	0.731596518
M713T213	0.731491963	0.982495368	0.731491963
M251T585	0.73147001	0.982494301	0.73147001
M147T256	0.731386922	0.982490481	0.731386922
M558T38	0.731237973	0.982483135	0.731237973
M227T297	0.731198459	0.982481138	0.731198459
M498T127	0.731176207	0.982480058	0.731176207
M554T168	0.731016405	0.982472324	0.731016405
M571T34	0.730863043	0.982464902	0.730863043
M365T238	0.730830554	0.982463332	0.730830554
M573T429	0.730662969	0.982455233	0.730662969
M461T45	0.730379775	0.982440649	0.730379775
M1025T354	0.730266243	0.98243631	0.730266243
M206T67	0.73014389	0.982430201	0.73014389
M261T92	0.729677334	0.982407767	0.729677334
M981T352	0.729613714	0.982404713	0.729613714
M389T220	0.729336213	0.982391399	0.729336213
M595T292	0.729335064	0.982391345	0.729335064
M232T452	0.729260301	0.982387762	0.729260301
M205T401	0.729023471	0.982376425	0.729023471
M166T319	0.728812115	0.982366324	0.728812115
M283T41	0.728747482	0.982363232	0.728747482
M358T68	0.728681168	0.982360156	0.728681168
M950T465	0.728503177	0.982351678	0.728503177
M597T148	0.728472077	0.982350091	0.728472077
M152T158	0.728040556	0.982329542	0.728040556
M704T57	0.72773796	0.982315163	0.72773796
M140T352	0.727654995	0.982348472	0.727654995
M539T320	0.727544123	0.982305977	0.727544123
M153T158	0.72736603	0.982297529	0.72736603
M425T221	0.727320169	0.982295357	0.727320169
M837T70	0.727246271	0.982291859	0.727246271

M138T286	0.727154764	0.982287529	0.727154764
M315T347	0.727118435	0.98228581	0.727118435
M411T344	0.727054315	0.982282778	0.727054315
M242T284	0.726935661	0.98227724	0.726935661
M97T348	0.726814528	0.982271452	0.726814528
M271T155	0.726708457	0.982266447	0.726708457
M547T259	0.726612691	0.982261931	0.726612691
M363T203	0.726480772	0.982255726	0.726480772
M245T278	0.726284901	0.982246492	0.726284901
M105T37	0.726024846	0.982234267	0.726024846
M478T199	0.725958084	0.982231171	0.725958084
M853T141	0.725897988	0.982228327	0.725897988
M433T246	0.725825376	0.982224902	0.725825376
M662T170	0.725735665	0.982220695	0.725735665
M242T59	0.72555958	0.982212443	0.72555958
M134T157_2	0.725544559	0.982211799	0.725544559
M343T94	0.725499444	0.982209632	0.725499444
M303T374	0.725497522	0.982209538	0.725497522
M265T26	0.725250661	0.982198009	0.725250661
M451T175	0.725173004	0.982194362	0.725173004
M742T256	0.724966364	0.98218471	0.724966364
M1198T171	0.724897655	0.982181504	0.724897655
M255T253	0.724775362	0.982175809	0.724775362
M1110T352_2	0.724624023	0.982168756	0.724624023
M166T193	0.724561895	0.982165867	0.724561895
M153T380	0.724404575	0.982158544	0.724404575
M1061T354_2	0.723992467	0.982139954	0.723992467
M163T429	0.723479698	0.982115669	0.723479698
M770T354	0.723327722	0.982108648	0.723327722
M1110T353	0.723017151	0.982094329	0.723017151
M743T38	0.722976556	0.982092451	0.722976556
M277T396	0.722780141	0.982083408	0.722780141
M638T344	0.722518541	0.982071383	0.722518541
M243T345_1	0.722405355	0.982066194	0.722405355
M415T272	0.72239415	0.982065672	0.72239415
M1167T354_1	0.722253266	0.98205921	0.722253266
M247T231	0.721942715	0.982044988	0.721942715
M1079T354	0.7218992	0.982042996	0.7218992
M977T352	0.721862107	0.982041299	0.721862107
M921T354	0.72178378	0.982037953	0.72178378
M701T178	0.721402539	0.982020315	0.721402539
M185T192	0.720925212	0.981998662	0.720925212

M337T351	0.720801986	0.981992991	0.720801986
M904T210	0.720779723	0.981992222	0.720779723
M186T253	0.720662685	0.981986662	0.720662685
M381T127	0.720624539	0.981984939	0.720624539
M207T326	0.720605167	0.981984206	0.720605167
M446T290	0.720549356	0.981981531	0.720549356
M159T35	0.720043506	0.981958635	0.720043506
M275T322	0.719781877	0.981946838	0.719781877
M980T352	0.719612828	0.981939218	0.719612828
M655T212	0.719483668	0.981933403	0.719483668
M287T135	0.719457814	0.981932242	0.719457814
M1181T465_2	0.719450418	0.981931908	0.719450418
M889T173	0.719422364	0.981930648	0.719422364
M935T32	0.719411092	0.981930151	0.719411092
M133T195	0.719261011	0.981923394	0.719261011
M578T41	0.71875919	0.98190089	0.71875919
M391T53	0.718723362	0.981899285	0.718723362
M131T257	0.718455813	0.981887325	0.718455813
M297T333	0.718400129	0.981884849	0.718400129
M217T462	0.718191669	0.981875534	0.718191669
M874T39	0.718031024	0.981868376	0.718031024
M522T189	0.717992231	0.981867121	0.717992231
M254T46	0.717915728	0.981863243	0.717915728
M933T97	0.717797649	0.98185799	0.717797649
M319T41	0.717673265	0.981852463	0.717673265
M271T165	0.717498757	0.981844719	0.717498757
M293T198	0.717486746	0.981844183	0.717486746
M229T118	0.717389326	0.981839862	0.717389326
M295T333	0.717319144	0.981836751	0.717319144
M315T157	0.717282518	0.981835131	0.717282518
M497T170	0.717265993	0.981834656	0.717265993
M356T163	0.71709989	0.981827045	0.71709989
M432T99	0.717098701	0.981826992	0.717098701
M723T272_1	0.716905269	0.981818442	0.716905269
M575T356	0.716882422	0.981817692	0.716882422
M806T198	0.71679195	0.981813433	0.71679195
M844T67	0.716489692	0.981800109	0.716489692
M1012T33	0.71637848	0.981795216	0.71637848
M664T171	0.716156627	0.981785459	0.716156627
M633T37	0.716104072	0.981783148	0.716104072
M755T148	0.716051058	0.981780835	0.716051058
M401T341	0.715411218	0.981753147	0.715411218

M223T39	0.71531178	0.981748492	0.71531178
M669T421	0.715272673	0.981746766	0.715272673
M135T229	0.715180361	0.981742731	0.715180361
M284T367	0.715111143	0.981739702	0.715111143
M339T40_2	0.714872753	0.981729318	0.714872753
M379T356	0.714469742	0.981711806	0.714469742
M982T32	0.714442315	0.981710618	0.714442315
M831T33	0.714153516	0.981698111	0.714153516
M509T237	0.713993935	0.9816912	0.713993935
M84T199	0.71390391	0.981687311	0.71390391
M135T260	0.713862998	0.981685544	0.713862998
M551T91	0.713599125	0.981674161	0.713599125
M353T253	0.713511642	0.981670398	0.713511642
M195T59	0.71334633	0.981663335	0.71334633
M315T408	0.713312093	0.981661806	0.713312093
M697T113	0.713290577	0.981660879	0.713290577
M603T32	0.71327025	0.981660007	0.71327025
M837T352	0.713127781	0.981653888	0.713127781
M302T238	0.713114464	0.981653317	0.713114464
M107T157	0.713099775	0.981652687	0.713099775
M959T354	0.712872651	0.981642941	0.712872651
M70T95	0.712843772	0.981641702	0.712843772
M217T334	0.712749595	0.98163767	0.712749595
M1157T354	0.712737309	0.981637145	0.712737309
M271T55	0.712417678	0.981623483	0.712417678
M72T233	0.712116514	0.981610631	0.712116514
M234T182	0.712072639	0.981608766	0.712072639
M205T59	0.711972024	0.981604481	0.711972024
M651T406	0.711904602	0.981602088	0.711904602
M1110T354	0.71186619	0.981599978	0.71186619
M170T258	0.711746267	0.981594882	0.711746267
M132T242_1	0.711719374	0.98159374	0.711719374
M933T42	0.711574083	0.981587575	0.711574083
M654T169	0.711545324	0.981586358	0.711545324
M627T162	0.711243461	0.981573569	0.711243461
M208T157	0.711115834	0.981568171	0.711115834
M597T190	0.710940062	0.98156075	0.710940062
M1158T352	0.710930439	0.981560344	0.710930439
M325T390	0.710785297	0.981554222	0.710785297
M655T379	0.710508677	0.981542577	0.710508677
M298T319	0.71044363	0.981539843	0.71044363
M206T231	0.710425587	0.981539084	0.710425587

M581T342	0.710202807	0.981529733	0.710202807
M971T354_2	0.710197501	0.981529508	0.710197501
M960T42	0.709822798	0.981513814	0.709822798
M125T35	0.709360189	0.981494507	0.709360189
M685T32	0.709310043	0.981492428	0.709310043
M549T428	0.709226126	0.981488924	0.709226126
M157T93	0.709208994	0.981488212	0.709208994
M1119T352	0.709189384	0.981487509	0.709189384
M147T180	0.709185826	0.981487338	0.709185826
M193T34_2	0.708995321	0.98147933	0.708995321
M521T272	0.708958224	0.981477788	0.708958224
M291T273	0.708749364	0.981469146	0.708749364
M315T373	0.708079686	0.981441453	0.708079686
M243T345_2	0.707977604	0.981437399	0.707977604
M666T23	0.707920107	0.981434879	0.707920107
M399T50	0.707905779	0.981434369	0.707905779
M421T335	0.707859403	0.98143235	0.707859403
M417T248	0.707843179	0.981431992	0.707843179
M725T403	0.707385126	0.981412913	0.707385126
M185T303	0.707251535	0.981407442	0.707251535
M688T193	0.707166919	0.981403981	0.707166919
M481T153	0.706773363	0.981387918	0.706773363
M807T138	0.706746648	0.981386829	0.706746648
M331T305	0.70651758	0.981377508	0.70651758
M1186T353	0.706272612	0.981367559	0.706272612
M639T36	0.706268424	0.98136743	0.706268424
M281T356	0.705748841	0.981346735	0.705748841
M815T33	0.705681097	0.981343862	0.705681097
M791T465	0.705563568	0.981338883	0.705563568
M771T89_1	0.705432366	0.981333604	0.705432366
M505T79	0.705346478	0.981330142	0.705346478
M387T32	0.705267403	0.981326971	0.705267403
M429T45	0.70522054	0.9813253	0.70522054
M497T212	0.704754061	0.981306372	0.704754061
M783T114	0.704413307	0.981292756	0.704413307
M136T156	0.70433601	0.98128967	0.70433601
M582T298	0.704293959	0.981288225	0.704293959
M251T186	0.704277781	0.981287349	0.704277781
M164T256	0.704164791	0.98128285	0.704164791
M705T264	0.70414928	0.981282704	0.70414928
M632T354	0.704000992	0.981276334	0.704000992
M257T277	0.703887375	0.98127182	0.703887375

M518T154	0.703881758	0.981271669	0.703881758
M707T413	0.703837483	0.981270009	0.703837483
M286T299_1	0.703806757	0.981268622	0.703806757
M1196T32	0.703720384	0.981265195	0.703720384
M437T58	0.703689637	0.981263977	0.703689637
M259T647	0.703687921	0.981264254	0.703687921
M513T428	0.703230456	0.981245835	0.703230456
M150T34	0.703117755	0.981241469	0.703117755
M639T380	0.702647764	0.981222889	0.702647764
M855T85	0.702562662	0.981219549	0.702562662
M343T91	0.702507299	0.981217378	0.702507299
M461T380	0.70250053	0.981217114	0.70250053
M285T130	0.702397964	0.981213097	0.702397964
M374T306	0.702241514	0.981206976	0.702241514
M527T428	0.70217973	0.981204566	0.70217973
M619T114	0.702086726	0.981200929	0.702086726
M1188T352	0.701819617	0.981190524	0.701819617
M1039T352_1	0.701705404	0.981186069	0.701705404
M339T328	0.701672134	0.981184776	0.701672134
M754T52	0.701478884	0.98117727	0.701478884
M855T352	0.701269995	0.981169242	0.701269995
M547T272_2	0.701101665	0.981162659	0.701101665
M546T206	0.70106118	0.981161101	0.70106118
M1114T171	0.701001186	0.981158774	0.701001186
M1143T36	0.700868916	0.981153667	0.700868916
M492T144	0.700805728	0.981151232	0.700805728
M485T380	0.700701925	0.981147232	0.700701925
M283T292	0.700622284	0.981144165	0.700622284
M158T26	0.700446361	0.981137402	0.700446361
M260T246	0.70026897	0.981130983	0.70026897
M1088T353	0.700249087	0.98112984	0.700249087
M344T197	0.699976946	0.98111941	0.699976946
M511T157_1	0.699926218	0.98111747	0.699926218
M231T260	0.699849082	0.981114522	0.699849082
M412T491	0.699779166	0.981111865	0.699779166
M605T451	0.699771354	0.981111556	0.699771354
M223T222	0.699770951	0.981111664	0.699770951
M336T352	0.699761062	0.981111166	0.699761062
M119T299	0.699610984	0.981105918	0.699610984
M162T398	0.699504193	0.981101376	0.699504193
M195T465	0.699483574	0.98110077	0.699483574
M425T204	0.699420539	0.981098193	0.699420539

M601T40	0.699369021	0.981096571	0.699369021
M227T381	0.699223082	0.981090698	0.699223082
M189T33	0.69900703	0.981082501	0.69900703
M488T169	0.698875952	0.981077557	0.698875952
M281T243	0.698787105	0.981074182	0.698787105
M559T32	0.698550968	0.981065272	0.698550968
M660T169	0.698227306	0.981053093	0.698227306
M762T148	0.698142398	0.981049906	0.698142398
M207T261	0.698060777	0.981046848	0.698060777
M398T203	0.698048193	0.98104637	0.698048193
M992T352_2	0.697995627	0.98104441	0.697995627
M633T247	0.697418451	0.981022846	0.697418451
M1032T352_2	0.697381277	0.98102146	0.697381277
M447T154	0.697365256	0.981020856	0.697365256
M811T130	0.697305851	0.981018645	0.697305851
M421T303	0.697092204	0.98101086	0.697092204
M140T90	0.696994037	0.981007064	0.696994037
M739T150	0.696990227	0.981006921	0.696990227
M933T161	0.696912269	0.981004032	0.696912269
M956T25	0.696460153	0.980987322	0.696460153
M1107T36	0.696403413	0.980985231	0.696403413
M672T213	0.696316643	0.980982035	0.696316643
M151T228	0.696215072	0.980978298	0.696215072
M896T145	0.696117933	0.980974737	0.696117933
M1085T352	0.696070815	0.980972996	0.696070815
M847T131	0.696020434	0.980971147	0.696020434
M336T262	0.695881947	0.98096607	0.695881947
M521T97	0.695649253	0.980957601	0.695649253
M558T170	0.69505793	0.980936022	0.69505793
M213T415	0.695023911	0.980934787	0.695023911
M213T55	0.694914228	0.980930811	0.694914228
M321T215	0.694801095	0.980926699	0.694801095
M617T293	0.694660383	0.980921646	0.694660383
M70T374	0.694612205	0.980919869	0.694612205
M951T113	0.69442044	0.980913366	0.69442044
M927T354	0.694383992	0.980911633	0.694383992
M1039T349	0.694361184	0.98091081	0.694361184
M1130T352	0.693917655	0.980894873	0.693917655
M292T395	0.693859858	0.980892799	0.693859858
M845T67	0.693828847	0.980891695	0.693828847
M593T415	0.69375318	0.980888987	0.69375318
M513T92	0.693433288	0.980877551	0.693433288

M541T35	0.693246994	0.980870921	0.693246994
M517T39	0.692982028	0.98086151	0.692982028
M325T292	0.692908128	0.980858901	0.692908128
M80T206	0.692887258	0.980858149	0.692887258
M788T147	0.692825147	0.980855945	0.692825147
M311T154	0.692703099	0.980851636	0.692703099
M1187T354_2	0.692679876	0.980850933	0.692679876
M474T135	0.692462815	0.980843144	0.692462815
M393T251	0.692425142	0.980842124	0.692425142
M917T173	0.69238355	0.980840352	0.69238355
M1079T352	0.692089772	0.980830035	0.692089772
M1090T352_2	0.691913343	0.980823836	0.691913343
M506T211	0.691848972	0.980821582	0.691848972
M118T27	0.691843309	0.980821384	0.691843309
M379T53	0.69163214	0.980814002	0.69163214
M834T189	0.691324926	0.980803669	0.691324926
M132T168	0.691269757	0.980801379	0.691269757
M164T273	0.69124812	0.980800633	0.69124812
M147T181	0.691054331	0.980793901	0.691054331
M751T292	0.691005587	0.980792212	0.691005587
M569T201	0.691003809	0.980792466	0.691003809
M812T354	0.690998192	0.980791955	0.690998192
M1097T34_1	0.690967063	0.980790877	0.690967063
M82T26	0.690937594	0.980789855	0.690937594
M401T180_1	0.69079701	0.980784993	0.69079701
M435T98	0.69068158	0.980781006	0.69068158
M431T272	0.690439566	0.980772666	0.690439566
M767T353	0.690414214	0.980771794	0.690414214
M1006T177	0.690394262	0.98077139	0.690394262
M483T26	0.690058817	0.980759594	0.690058817
M787T131	0.689517371	0.980741567	0.689517371
M171T268	0.689465882	0.980739737	0.689465882
M174T264	0.689357727	0.980735695	0.689357727
M205T286	0.689313971	0.980734416	0.689313971
M105T381	0.689292702	0.98073348	0.689292702
M337T292	0.689286215	0.980733259	0.689286215
M508T443	0.689215842	0.980730916	0.689215842
M673T192	0.689149561	0.98072881	0.689149561
M501T170	0.689073013	0.980726308	0.689073013
M431T250	0.689003476	0.980723685	0.689003476
M391T124	0.688784631	0.980716298	0.688784631
M137T354	0.688760895	0.980715499	0.688760895

M736T26_2	0.688757765	0.980715392	0.688757765
M336T68	0.688221585	0.98069739	0.688221585
M257T34_1	0.688208715	0.980696959	0.688208715
M301T374	0.688114842	0.980693816	0.688114842
M321T183	0.688097657	0.980693257	0.688097657
M794T71	0.687962218	0.980715295	0.687962218
M201T278	0.687918936	0.980687276	0.687918936
M1059T354_1	0.687286612	0.980666411	0.687286612
M950T464_1	0.686805642	0.980650434	0.686805642
M486T106	0.686699766	0.980646958	0.686699766
M348T230	0.686698542	0.98064692	0.686698542
M433T374	0.686698293	0.980646915	0.686698293
M116T374	0.686373519	0.980636279	0.686373519
M289T116	0.686364533	0.980636364	0.686364533
M204T433	0.686323552	0.980634648	0.686323552
M471T374	0.686318455	0.980634478	0.686318455
M295T357	0.686307407	0.980634121	0.686307407
M459T203	0.686252833	0.980632357	0.686252833
M793T96	0.686232012	0.980631665	0.686232012
M219T262	0.686174483	0.980629787	0.686174483
M387T37	0.685982936	0.980623568	0.685982936
M411T35	0.685968394	0.980623093	0.685968394
M476T119	0.685954167	0.980623078	0.685954167
M715T243	0.685592403	0.980610933	0.685592403
M692T199	0.685379811	0.98060404	0.685379811
M535T354	0.685293305	0.980601255	0.685293305
M339T352	0.685278624	0.980600781	0.685278624
M160T35	0.68520756	0.980598496	0.68520756
M717T171	0.685043982	0.980593243	0.685043982
M677T35	0.684838208	0.980586654	0.684838208
M761T353	0.684492217	0.980575616	0.684492217
M709T171	0.684405596	0.980573149	0.684405596
M369T565	0.684340467	0.980570791	0.684340467
M589T260	0.684333266	0.980570633	0.684333266
M194T249	0.68417536	0.980565551	0.68417536
M1190T171	0.683922972	0.980557568	0.683922972
M689T463	0.683553199	0.980545945	0.683553199
M891T292_1	0.683415193	0.980541596	0.683415193
M1069T354_1	0.683389603	0.980540865	0.683389603
M209T293	0.683354858	0.980539704	0.683354858
M1167T354_2	0.683317145	0.980538525	0.683317145
M1013T354	0.683316693	0.98053851	0.683316693

M743T351	0.683186681	0.980534444	0.683186681
M293T272	0.683165579	0.980533786	0.683165579
M195T493	0.682931099	0.98052696	0.682931099
M129T402	0.682906159	0.980525699	0.682906159
M231T323	0.682896389	0.980525396	0.682896389
M996T139	0.682716657	0.980519812	0.682716657
M270T143	0.682526164	0.980513912	0.682526164
M385T201	0.682449242	0.980511532	0.682449242
M346T250	0.68173979	0.980489722	0.68173979
M665T354	0.68170705	0.980488888	0.68170705
M315T288	0.681690651	0.980488241	0.681690651
M233T264	0.681609608	0.980485748	0.681609608
M370T231	0.681458942	0.980481153	0.681458942
M576T92	0.681416767	0.980479869	0.681416767
M509T385	0.681335577	0.980477399	0.681335577
M435T374	0.681055088	0.980468894	0.681055088
M160T397	0.680705898	0.980458355	0.680705898
M291T247	0.680703479	0.980458283	0.680703479
M425T541	0.680247183	0.980444598	0.680247183
M637T340	0.680238852	0.980444355	0.680238852
M858T135	0.680207056	0.980443398	0.680207056
M429T214	0.680112783	0.980440575	0.680112783
M205T39	0.680056779	0.980438914	0.680056779
M996T161	0.680050245	0.980438721	0.680050245
M999T42	0.679954831	0.980435879	0.679954831
M629T451	0.679937469	0.980435363	0.679937469
M577T81	0.679501975	0.980422599	0.679501975
M994T348	0.679198879	0.980413526	0.679198879
M1139T354_1	0.67904323	0.980408969	0.67904323
M305T290	0.678547717	0.980394488	0.678547717
M275T219	0.678495921	0.980392995	0.678495921
M606T272_2	0.678485564	0.980392681	0.678485564
M418T246	0.678387641	0.980389838	0.678387641
M180T83	0.678089859	0.980381264	0.678089859
M371T211	0.678033836	0.980379605	0.678033836
M684T32	0.677648327	0.980368832	0.677648327
M389T32	0.677646106	0.98036846	0.677646106
M247T278	0.677344383	0.980359837	0.677344383
M227T431	0.677124049	0.980353567	0.677124049
M302T236	0.676975287	0.980349347	0.676975287
M640T350	0.6769263	0.98034796	0.6769263
M433T199	0.676862993	0.980346169	0.676862993

M604T168	0.676782811	0.980343904	0.676782811
M207T304	0.676654098	0.980340274	0.676654098
M515T321	0.676318209	0.980330838	0.676318209
M135T34_2	0.676161216	0.980326547	0.676161216
M89T56	0.676131249	0.980325611	0.676131249
M379T453	0.675917849	0.980320071	0.675917849
M816T433	0.675819093	0.980316918	0.675819093
M256T184	0.675577519	0.980310226	0.675577519
M534T264	0.675543767	0.980309364	0.675543767
M248T272	0.67551834	0.980308589	0.67551834
M376T34	0.675404524	0.98030545	0.675404524
M347T379	0.674974073	0.980293631	0.674974073
M343T200	0.67486257	0.980290974	0.67486257
M943T202	0.674382273	0.980277532	0.674382273
M812T50_1	0.674380585	0.980277507	0.674380585
M479T113	0.67434678	0.980276572	0.67434678
M279T109	0.674310481	0.980275589	0.674310481
M517T204	0.674171651	0.980272317	0.674171651
M144T398	0.674033155	0.980268115	0.674033155
M261T35	0.67327133	0.980247779	0.67327133
M919T352	0.672924693	0.980238681	0.672924693
M244T256	0.672787785	0.980235027	0.672787785
M307T380	0.672786685	0.980234994	0.672786685
M224T62	0.672773285	0.980234642	0.672773285
M330T72	0.672528343	0.980228748	0.672528343
M1022T39_2	0.672342217	0.980223373	0.672342217
M363T390	0.672265229	0.980221728	0.672265229
M631T352	0.672035843	0.980215421	0.672035843
M179T59	0.671977622	0.98021392	0.671977622
M297T346	0.671863453	0.980210967	0.671863453
M113T27_1	0.671804449	0.980209462	0.671804449
M349T216	0.671767379	0.980208493	0.671767379
M308T214	0.671754352	0.980208157	0.671754352
M1189T354	0.671689487	0.980206561	0.671689487
M269T381	0.671675301	0.980206186	0.671675301
M325T117	0.67082142	0.980184372	0.67082142
M261T64	0.670803217	0.980183911	0.670803217
M277T170	0.670709804	0.980181556	0.670709804
M491T33_3	0.670655679	0.980180192	0.670655679
M1099T352	0.670445283	0.980174909	0.670445283
M177T105	0.670410575	0.980174039	0.670410575
M466T124	0.670208787	0.980168995	0.670208787

M138T380	0.670193518	0.980168615	0.670193518
M172T184	0.670187755	0.980168472	0.670187755
M668T22	0.670129158	0.980167014	0.670129158
M74T366	0.670088435	0.980165993	0.670088435
M301T311	0.670077442	0.980165725	0.670077442
M352T93	0.670029474	0.980164533	0.670029474
M699T214	0.669973054	0.980163133	0.669973054
M370T239	0.669943647	0.980162498	0.669943647
M1017T32	0.669607214	0.980154089	0.669607214
M234T65	0.669597273	0.980153842	0.669597273
M285T378	0.669449842	0.98015022	0.669449842
M226T78	0.669447007	0.980150149	0.669447007
M603T83	0.669295861	0.980146446	0.669295861
M299T367	0.669156209	0.980143035	0.669156209
M441T174	0.669003906	0.980139326	0.669003906
M739T208	0.66899685	0.980139155	0.66899685
M1109T354_1	0.668965318	0.980138388	0.668965318
M805T56	0.668934025	0.980137628	0.668934025
M353T130	0.668713944	0.980132319	0.668713944
M281T34	0.668529003	0.980127856	0.668529003
M116T309	0.668236345	0.980120821	0.668236345
M193T173	0.667984309	0.980114813	0.667984309
M213T85	0.667788791	0.980110176	0.667788791
M610T352	0.667777373	0.980109909	0.667777373
M88T392	0.667662255	0.980107187	0.667662255
M749T112	0.66728443	0.980098306	0.66728443
M141T303	0.667283258	0.98009828	0.667283258
M449T60	0.667145949	0.980095135	0.667145949
M293T255	0.667071721	0.980094231	0.667071721
M940T228	0.66705961	0.980093209	0.66705961
M587T414	0.666911366	0.980089616	0.666911366
M130T363	0.666873441	0.980088752	0.666873441
M837T39	0.666688227	0.980084456	0.666688227
M92T157	0.666679082	0.980084241	0.666679082
M352T130	0.666515289	0.980080469	0.666515289
M217T28	0.666483802	0.980079745	0.666483802
M296T194	0.666267482	0.980074788	0.666267482
M302T252	0.666240571	0.980074217	0.666240571
M678T33_2	0.666231117	0.980073959	0.666231117
M876T171	0.666231089	0.980073958	0.666231089
M495T38	0.665916691	0.980066806	0.665916691
M489T169	0.665869058	0.980065727	0.665869058

M886T102	0.665768739	0.98006346	0.665768739
M349T28_2	0.665717276	0.980062297	0.665717276
M863T354	0.665696933	0.980061847	0.665696933
M174T48	0.665679798	0.980061454	0.665679798
M1181T39	0.66557616	0.980094065	0.66557616
M750T78	0.665543707	0.980058393	0.665543707
M915T126	0.665514027	0.980057733	0.665514027
M1023T354	0.6652443	0.980051692	0.6652443
M223T257	0.665097352	0.980048437	0.665097352
M201T91	0.665090635	0.980048276	0.665090635
M167T47	0.665066517	0.98004774	0.665066517
M261T330	0.664970283	0.980045603	0.664970283
M153T27	0.664832947	0.980042566	0.664832947
M360T76	0.664828447	0.980042466	0.664828447
M785T131	0.664758024	0.980040914	0.664758024
M501T379	0.664582752	0.980037062	0.664582752
M1161T354	0.664389958	0.980032842	0.664389958
M605T152	0.663648053	0.980016805	0.663648053
M420T179	0.663556791	0.98001485	0.663556791
M625T351	0.66349883	0.980013612	0.66349883
M146T85	0.663330545	0.980010314	0.663330545
M293T464	0.663241679	0.980008143	0.663241679
M388T333	0.662872104	0.98000035	0.662872104
M483T45	0.662823029	0.979999811	0.662823029
M639T373	0.662777659	0.979998369	0.662777659
M404T115	0.66262809	0.979995245	0.66262809
M409T251	0.662151628	0.979985378	0.662151628
M846T66	0.662141939	0.979985182	0.662141939
M295T18	0.662029835	0.979982883	0.662029835
M1111T172	0.661867156	0.979979546	0.661867156
M241T96	0.66177088	0.97997772	0.66177088
M319T239	0.661687878	0.979975894	0.661687878
M1176T260	0.66168478	0.979975831	0.66168478
M281T213	0.661682507	0.979975785	0.661682507
M653T352	0.661568434	0.979973471	0.661568434
M386T334	0.660907536	0.979960214	0.660907536
M208T381	0.660752763	0.979957143	0.660752763
M817T354	0.660680822	0.979955721	0.660680822
M255T262	0.660596184	0.979954052	0.660596184
M788T137	0.66054362	0.979953017	0.66054362
M437T231	0.660354432	0.979949308	0.660354432
M419T261	0.660297587	0.979948198	0.660297587

M510T253	0.660135984	0.979945078	0.660135984
M764T354	0.660134833	0.979945037	0.660134833
M970T354	0.660100884	0.979944367	0.660100884
M806T157	0.660079212	0.979943948	0.660079212
M591T381	0.659786495	0.979938295	0.659786495
M587T260	0.659674092	0.979936139	0.659674092
M87T374	0.659619243	0.979935591	0.659619243
M353T204	0.659204156	0.979927194	0.659204156
M429T55	0.658982903	0.97992303	0.658982903
M133T374	0.658689097	0.979917544	0.658689097
M74T374	0.658679473	0.979917363	0.658679473
M294T269	0.658635694	0.979916547	0.658635694
M1069T354_2	0.658445882	0.979913058	0.658445882
M641T484	0.658429174	0.97991273	0.658429174
M357T243_2	0.658313432	0.979910598	0.658313432
M440T260	0.658150472	0.979907611	0.658150472
M451T32	0.657933914	0.979903899	0.657933914
M249T38	0.657425689	0.979894521	0.657425689
M291T442	0.657240149	0.979891225	0.657240149
M931T185	0.657032416	0.979887547	0.657032416
M992T176	0.657024367	0.979887406	0.657024367
M126T48	0.656945767	0.979886052	0.656945767
M575T26	0.656727322	0.979882201	0.656727322
M102T386	0.65666182	0.979881061	0.65666182
M304T127	0.656608857	0.979880157	0.656608857
M237T125	0.656593412	0.979879889	0.656593412
M184T377	0.656501374	0.979878279	0.656501374
M813T74	0.656493375	0.97987814	0.656493375
M222T281	0.656399925	0.979876526	0.656399925
M1147T39	0.656396235	0.979876462	0.656396235
M437T352	0.655871005	0.979867494	0.655871005
M154T44	0.655731333	0.979865139	0.655731333
M353T92	0.655576041	0.979862529	0.655576041
M249T79	0.655431679	0.97986015	0.655431679
M237T356	0.655339189	0.979859142	0.655339189
M316T179	0.655271833	0.979857502	0.655271833
M1062T352	0.655214327	0.979856513	0.655214327
M195T181_2	0.655202112	0.979856314	0.655202112
M351T397	0.654830106	0.979850211	0.654830106
M823T70	0.654775982	0.979849343	0.654775982
M728T26_1	0.65477235	0.979849271	0.65477235
M255T343	0.654747693	0.979848869	0.654747693

M187T381	0.654708347	0.979848389	0.654708347
M496T130	0.654660722	0.979847459	0.654660722
M177T464	0.65456324	0.97984589	0.65456324
M482T115	0.654554001	0.979845847	0.654554001
M812T202	0.654480663	0.979844575	0.654480663
M289T467	0.654384301	0.979843008	0.654384301
M413T184	0.653650466	0.97983142	0.653650466
M559T34	0.653297574	0.979825965	0.653297574
M565T213	0.652979073	0.979821134	0.652979073
M505T290	0.652654691	0.979816231	0.652654691
M790T52	0.652487503	0.979813736	0.652487503
M659T212	0.652389501	0.979812285	0.652389501
M304T183	0.652333986	0.979811465	0.652333986
M652T352	0.652291078	0.979811015	0.652291078
M118T205	0.652049278	0.97980729	0.652049278
M1129T352_1	0.652011639	0.979806742	0.652011639
M439T393	0.651709675	0.979802379	0.651709675
M155T380	0.651606534	0.979800901	0.651606534
M438T169	0.651584807	0.97980059	0.651584807
M455T305	0.651577733	0.97980049	0.651577733
M361T388	0.651345319	0.979797188	0.651345319
M70T309	0.651279442	0.97979626	0.651279442
M265T111	0.651267926	0.979796098	0.651267926
M370T252	0.651157586	0.97979455	0.651157586
M686T193	0.651103105	0.979793785	0.651103105
M551T234	0.651077027	0.979793422	0.651077027
M562T40	0.650879132	0.97979087	0.650879132
M149T315	0.650620981	0.979787121	0.650620981
M827T106	0.650618581	0.979787246	0.650618581
M339T82	0.650403338	0.97978429	0.650403338
M450T26_1	0.650307232	0.979782864	0.650307232
M924T34	0.650190951	0.979781302	0.650190951
M817T148_2	0.650149813	0.979780751	0.650149813
M357T113	0.649999991	0.97977876	0.649999991
M785T352_2	0.649979883	0.979778723	0.649979883
M491T273	0.64981277	0.979776282	0.64981277
M291T264	0.649794793	0.979776046	0.649794793
M367T150	0.649581622	0.979773262	0.649581622
M242T256	0.649549368	0.979772839	0.649549368
M406T297	0.649385269	0.979770718	0.649385269
M497T51	0.649362967	0.979770457	0.649362967
M310T34	0.649358096	0.979770739	0.649358096

M190T36	0.649323671	0.979769926	0.649323671
M845T56	0.649143696	0.979767627	0.649143696
M163T320	0.648989627	0.979765674	0.648989627
M308T377	0.648985883	0.979765627	0.648985883
M891T52	0.648860337	0.979764056	0.648860337
M110T159	0.648726052	0.979762369	0.648726052
M691T57	0.648651181	0.979761459	0.648651181
M119T462	0.64854019	0.979760066	0.64854019
M553T33	0.648446535	0.979758912	0.648446535
M689T414_2	0.648409659	0.979758462	0.648409659
M162T337	0.648409598	0.979758465	0.648409598
M914T354_2	0.648341741	0.979757633	0.648341741
M897T93	0.648299193	0.979757113	0.648299193
M366T217_2	0.648250537	0.979756522	0.648250537
M766T81_1	0.648247628	0.979756498	0.648247628
M263T32	0.64820826	0.979756039	0.64820826
M124T206	0.648166422	0.979755504	0.648166422
M486T37	0.648038824	0.979753966	0.648038824
M843T89	0.647939057	0.979752773	0.647939057
M195T408	0.647853841	0.979751757	0.647853841
M573T191	0.647683854	0.979749747	0.647683854
M390T272	0.647439611	0.979746938	0.647439611
M315T360	0.64735968	0.979745978	0.64735968
M647T356	0.647274715	0.979745002	0.647274715
M331T183	0.647136069	0.979743397	0.647136069
M267T33	0.647024647	0.979742127	0.647024647
M1151T39	0.646833219	0.979739972	0.646833219
M567T354	0.64675897	0.979739141	0.64675897
M260T281	0.646722427	0.979738734	0.646722427
M195T354	0.64654547	0.979736773	0.64654547
M141T98	0.646458705	0.979735832	0.646458705
M453T378	0.646458417	0.979735847	0.646458417
M791T70	0.646355259	0.979734689	0.646355259
M255T26_2	0.645936215	0.979730434	0.645936215
M447T34	0.645882943	0.97972962	0.645882943
M253T129	0.645728395	0.979727993	0.645728395
M468T345	0.645348634	0.979724064	0.645348634
M815T129	0.645023115	0.979720773	0.645023115
M181T38	0.644724161	0.979717813	0.644724161
M772T54	0.643895936	0.979709932	0.643895936
M765T81_2	0.643749035	0.979708586	0.643749035
M841T38	0.643633229	0.97970753	0.643633229

M321T103	0.643595752	0.979707191	0.643595752
M957T353	0.64347975	0.97970615	0.64347975
M293T117	0.643332291	0.979704836	0.643332291
M373T422	0.643074201	0.979702602	0.643074201
M356T35_1	0.642982196	0.979701779	0.642982196
M722T203	0.64282842	0.979700465	0.64282842
M171T51	0.642759719	0.979700073	0.642759719
M828T353	0.642604254	0.979698576	0.642604254
M1120T38	0.642601949	0.979698558	0.642601949
M507T160	0.642526888	0.979698213	0.642526888
M1063T354	0.642286124	0.979696061	0.642286124
M927T102	0.64222882	0.979695492	0.64222882
M513T348	0.64213954	0.979694873	0.64213954
M807T352	0.642050525	0.979694023	0.642050525
M564T212	0.641894776	0.97969283	0.641894776
M178T204	0.641838702	0.979692416	0.641838702
M1162T354	0.641673901	0.979691113	0.641673901
M375T155	0.641670981	0.979691091	0.641670981
M678T500	0.641569713	0.979690315	0.641569713
M515T92	0.641534091	0.979690044	0.641534091
M1181T465_1	0.641496216	0.979689757	0.641496216
M567T272	0.64128121	0.979688144	0.64128121
M127T205	0.640717856	0.979684074	0.640717856
M145T320	0.640680628	0.979683813	0.640680628
M151T37	0.64064314	0.979683551	0.64064314
M880T55	0.640632693	0.979683492	0.640632693
M399T243	0.640551306	0.979682954	0.640551306
M379T129	0.640458127	0.979682457	0.640458127
M192T19	0.640285122	0.979681103	0.640285122
M173T17	0.640227985	0.9796807	0.640227985
M207T59	0.640196188	0.979680507	0.640196188
M445T170	0.640051067	0.979679546	0.640051067
M309T136	0.640047365	0.979679522	0.640047365
M452T115	0.639498389	0.979676053	0.639498389
M666T36	0.639254063	0.979674756	0.639254063
M293T381	0.639171323	0.97967406	0.639171323
M331T380	0.638870413	0.979672318	0.638870413
M277T353	0.638740416	0.979671572	0.638740416
M217T34	0.638712284	0.979671415	0.638712284
M117T413	0.638350487	0.979669439	0.638350487
M294T254	0.63822685	0.979668784	0.63822685
M581T306	0.638131809	0.979668289	0.638131809

M307T293	0.637894798	0.979667085	0.637894798
M439T122	0.637797894	0.979666605	0.637797894
M206T185	0.637781757	0.979666561	0.637781757
M187T328	0.637581993	0.979665572	0.637581993
M1050T100	0.636870175	0.979662345	0.636870175
M329T100	0.636767016	0.97966193	0.636767016
M595T36	0.636735088	0.979661799	0.636735088
M584T33	0.636237429	0.979659807	0.636237429
M215T289	0.636175767	0.9796596	0.636175767
M375T234	0.635948784	0.979658741	0.635948784
M1032T171	0.635789006	0.979658181	0.635789006
M171T375	0.635783108	0.97965816	0.635783108
M379T422	0.635595229	0.979657527	0.635595229
M818T147_2	0.635562019	0.979657417	0.635562019
M229T192	0.63553423	0.979657326	0.63553423
M452T130	0.635304719	0.979656598	0.635304719
M1113T36	0.635192897	0.979656257	0.635192897
M684T29	0.634971552	0.979655611	0.634971552
M480T143	0.634929577	0.979655492	0.634929577
M369T239	0.63491609	0.979655458	0.63491609
M497T91	0.634891658	0.979655386	0.634891658
M368T44	0.634853699	0.979655287	0.634853699
M365T436	0.634736979	0.979654965	0.634736979
M337T33	0.634713527	0.979654941	0.634713527
M317T308	0.634269713	0.979653836	0.634269713
M298T109	0.633839123	0.97965321	0.633839123
M331T408	0.633798825	0.979652802	0.633798825
M996T115	0.633761674	0.979652785	0.633761674
M404T205	0.633732791	0.979652682	0.633732791
M460T26	0.633630464	0.979652484	0.633630464
M71T108	0.633594603	0.979652419	0.633594603
M308T120	0.633204378	0.979651777	0.633204378
M211T351	0.633167234	0.979651723	0.633167234
M151T143	0.633107617	0.979651635	0.633107617
M306T517	0.632967868	0.979651445	0.632967868
M469T202	0.632922944	0.979651386	0.632922944
M467T169	0.632821557	0.97965126	0.632821557
M363T96	0.632814185	0.979651251	0.632814185
M351T345	0.632776839	0.9796529	0.632776839
M739T239	0.632756061	0.979651677	0.632756061
M223T263	0.632738755	0.979651163	0.632738755
M421T171	0.632551919	0.979650972	0.632551919

M545T116	0.632506773	0.979650919	0.632506773
M114T374	0.632296851	0.979650748	0.632296851
M689T414_1	0.631997675	0.979650545	0.631997675
M507T321	0.631601923	0.979650366	0.631601923
M217T278	0.631263738	0.979650323	0.631263738
M961T354_1	0.630699005	0.979650494	0.630699005
M134T204	0.630082546	0.979651223	0.630082546
M264T311	0.630054098	0.979651201	0.630054098
M540T98	0.629989504	0.979650967	0.629989504
M529T304	0.629744721	0.979651281	0.629744721
M463T379	0.629672304	0.979651342	0.629672304
M343T252	0.629419595	0.979651673	0.629419595
M180T36	0.629415907	0.979651693	0.629415907
M250T35	0.62938067	0.979652177	0.62938067
M229T557	0.629224203	0.979651991	0.629224203
M775T156	0.629072847	0.979652226	0.629072847
M856T352	0.628919335	0.979652502	0.628919335
M816T38	0.62854096	0.979653264	0.62854096
M620T354	0.628282849	0.97965385	0.628282849
M150T380	0.628195956	0.979654076	0.628195956
M393T258	0.62819587	0.979654061	0.62819587
M1077T318_2	0.628156152	0.979654158	0.628156152
M247T269	0.628138783	0.979654201	0.628138783
M116T392	0.628131453	0.979654226	0.628131453
M114T310	0.628116715	0.979654256	0.628116715
M813T354	0.628100753	0.979654328	0.628100753
M315T229	0.627967443	0.979654639	0.627967443
M1101T340_3	0.627921076	0.979654762	0.627921076
M191T200	0.627886104	0.979654876	0.627886104
M364T110	0.62760839	0.979655644	0.62760839
M390T382	0.627585783	0.97965577	0.627585783
M677T177	0.627517838	0.979655905	0.627517838
M686T37	0.627359689	0.979656389	0.627359689
M366T240	0.627311536	0.979656547	0.627311536
M502T108	0.627303302	0.979656564	0.627303302
M369T492	0.627276464	0.979656651	0.627276464
M1097T34_2	0.627251065	0.979656732	0.627251065
M479T309	0.62691321	0.979657864	0.62691321
M747T256	0.626891068	0.979658046	0.626891068
M1155T34	0.626692796	0.979658654	0.626692796
M365T230	0.626609548	0.979658962	0.626609548
M589T358	0.626596216	0.979659043	0.626596216

M339T308	0.626452287	0.979659559	0.626452287
M293T408	0.626375121	0.979659861	0.626375121
M231T409	0.626372018	0.979659875	0.626372018
M207T280	0.626091148	0.979661045	0.626091148
M457T285	0.625951168	0.979661631	0.625951168
M1160T59	0.625917088	0.979661761	0.625917088
M697T200	0.625406892	0.979664071	0.625406892
M245T297	0.625185993	0.979665121	0.625185993
M321T363	0.625015535	0.979665971	0.625015535
M801T452	0.624823815	0.979666968	0.624823815
M351T256	0.62469517	0.979667637	0.62469517
M674T34_1	0.624343964	0.979669562	0.624343964
M87T116	0.624326706	0.979669656	0.624326706
M707T241	0.624113517	0.979670887	0.624113517
M240T233	0.624002064	0.97967157	0.624002064
M167T59	0.62383053	0.979672559	0.62383053
M890T292	0.623818727	0.979672631	0.623818727
M1111T354	0.623772813	0.97967291	0.623772813
M742T200	0.623645581	0.979673708	0.623645581
M643T35	0.623412076	0.979675185	0.623412076
M337T587	0.623275937	0.97967605	0.623275937
M149T31	0.623173696	0.979676723	0.623173696
M1064T354	0.623029705	0.979677688	0.623029705
M893T352	0.62263675	0.979680398	0.62263675
M193T381	0.622434765	0.979681847	0.622434765
M766T81_2	0.622424311	0.97968194	0.622424311
M501T129	0.622163851	0.979683843	0.622163851
M725T146	0.622102325	0.979684305	0.622102325
M332T354	0.622080767	0.979684468	0.622080767
M227T106	0.621883107	0.97968599	0.621883107
M75T171	0.621876342	0.979686175	0.621876342
M331T124	0.621791121	0.979686694	0.621791121
M882T352	0.621699569	0.979687674	0.621699569
M423T99	0.621539912	0.979688682	0.621539912
M263T303	0.621221273	0.979691284	0.621221273
M901T54	0.621112866	0.979692189	0.621112866
M439T36	0.621032059	0.979692872	0.621032059
M261T77	0.620832753	0.979694577	0.620832753
M208T108	0.620751011	0.979695285	0.620751011
M344T86	0.620750833	0.979695289	0.620750833
M485T303	0.620446841	0.979698254	0.620446841
M460T85	0.620426864	0.97969818	0.620426864

M765T171	0.620412803	0.979698306	0.620412803
M933T25	0.620318833	0.979699135	0.620318833
M136T380	0.619764933	0.979705411	0.619764933
M927T168	0.619764893	0.979704316	0.619764893
M779T111	0.619435853	0.97970751	0.619435853
M159T258	0.619345102	0.979708441	0.619345102
M267T259	0.61932269	0.97970864	0.61932269
M1113T171	0.619288935	0.979708973	0.619288935
M356T243	0.619159385	0.979710289	0.619159385
M776T112	0.618641027	0.979715656	0.618641027
M673T354	0.618623131	0.979715837	0.618623131
M96T59	0.618560928	0.979716493	0.618560928
M236T27	0.618544849	0.979716685	0.618544849
M680T293	0.618144763	0.979711606	0.618144763
M594T292_1	0.618081439	0.97972171	0.618081439
M237T182	0.617153457	0.979732391	0.617153457
M461T129	0.616965499	0.979734657	0.616965499
M1113T339_2	0.616835147	0.979736265	0.616835147
M111T33	0.616768431	0.979737062	0.616768431
M1129T352_2	0.616517426	0.979740167	0.616517426
M330T39	0.615854369	0.979738825	0.615854369
M142T272	0.615745657	0.979750103	0.615745657
M1179T39	0.615590763	0.979752162	0.615590763
M388T183	0.615581416	0.979752293	0.615581416
M355T236	0.61556676	0.979752488	0.61556676
M301T114	0.615360928	0.979755261	0.615360928
M150T167	0.615163865	0.979757956	0.615163865
M247T259	0.615090077	0.979758976	0.615090077
M374T310	0.614978866	0.97976052	0.614978866
M273T135	0.614922642	0.979761305	0.614922642
M93T320	0.614777553	0.979763481	0.614777553
M739T77	0.614687051	0.979764628	0.614687051
M490T34_2	0.614577474	0.979766192	0.614577474
M402T230	0.614401148	0.979768734	0.614401148
M255T272	0.614199032	0.979771679	0.614199032
M902T352	0.614148787	0.979772417	0.614148787
M1182T465_1	0.613986988	0.979774812	0.613986988
M122T207	0.613822903	0.97977728	0.613822903
M247T389	0.613821518	0.979777286	0.613821518
M248T116	0.613759071	0.979778249	0.613759071
M245T157	0.613715789	0.97977888	0.613715789
M1051T168	0.61328729	0.979785455	0.61328729

M345T251	0.61325691	0.979785924	0.61325691
M303T51	0.613239643	0.979786192	0.613239643
M360T116_1	0.613118006	0.9798004	0.613118006
M666T416	0.612970946	0.979790424	0.612970946
M476T199	0.612877394	0.979791902	0.612877394
M521T155	0.612609887	0.979796315	0.612609887
M287T378	0.612305372	0.97980121	0.612305372
M676T156	0.612248838	0.979802107	0.612248838
M306T91	0.61217264	0.979803369	0.61217264
M679T32_1	0.612152542	0.979803702	0.612152542
M338T34	0.611950847	0.979807103	0.611950847
M274T169	0.611933697	0.979807364	0.611933697
M746T32	0.611926425	0.979807487	0.611926425
M413T408	0.611465168	0.979815359	0.611465168
M614T236	0.611351845	0.979817331	0.611351845
M489T32	0.610964719	0.979824143	0.610964719
M805T353	0.610901088	0.979825347	0.610901088
M151T242	0.610814665	0.979827019	0.610814665
M404T333	0.610679809	0.979829243	0.610679809
M337T319	0.610385452	0.979825044	0.610385452
M329T104	0.610376539	0.979834766	0.610376539
M373T42	0.61021513	0.97983775	0.61021513
M821T47	0.610177873	0.979838435	0.610177873
M157T53_1	0.609963201	0.979842438	0.609963201
M486T154	0.609646057	0.97984844	0.609646057
M812T50_2	0.609382271	0.979853508	0.609382271
M159T343	0.609019683	0.979860592	0.609019683
M234T357	0.608810539	0.979864731	0.608810539
M246T274	0.608800922	0.979864922	0.608800922
M222T60	0.608785889	0.979865222	0.608785889
M163T258	0.608774556	0.979865448	0.608774556
M955T97	0.608721203	0.979866543	0.608721203
M190T327	0.608683087	0.979867305	0.608683087
M112T299	0.608669602	0.979867577	0.608669602
M765T354	0.608662267	0.97986791	0.608662267
M389T380	0.608614848	0.979868646	0.608614848
M342T163	0.608558497	0.979869788	0.608558497
M543T37	0.608408487	0.979872818	0.608408487
M313T292	0.608178813	0.979877516	0.608178813
M603T188	0.607856818	0.979884189	0.607856818
M547T320	0.607790006	0.979885577	0.607790006
M351T120	0.607724859	0.979886944	0.607724859

M507T381	0.607553099	0.979890568	0.607553099
M847T66	0.607469678	0.979892338	0.607469678
M688T414	0.607209172	0.979897913	0.607209172
M321T274	0.607132985	0.979899562	0.607132985
M766T138	0.606613889	0.979910912	0.606613889
M155T352	0.606561794	0.979912068	0.606561794
M222T204	0.606544452	0.979912459	0.606544452
M475T388	0.606543193	0.979912495	0.606543193
M675T144	0.606512275	0.979913169	0.606512275
M384T215	0.606498729	0.979913471	0.606498729
M520T205	0.606443909	0.979914692	0.606443909
M839T52	0.606423274	0.979915151	0.606423274
M473T378	0.606421715	0.979915186	0.606421715
M513T335	0.606272333	0.979918534	0.606272333
M322T260	0.606222396	0.979919669	0.606222396
M905T112	0.606183716	0.979920532	0.606183716
M505T351	0.606071899	0.979923397	0.606071899
M337T39	0.605952216	0.979925865	0.605952216
M439T168_2	0.605883461	0.979927365	0.605883461
M341T435	0.60577152	0.979929966	0.60577152
M282T237	0.605522103	0.97993571	0.605522103
M191T370	0.605496311	0.979936302	0.605496311
M400T245	0.605443675	0.97993753	0.605443675
M593T292	0.605003974	0.979947899	0.605003974
M1181T465_4	0.604963007	0.97994888	0.604963007
M288T274	0.604819673	0.979952323	0.604819673
M209T78	0.604789645	0.979953026	0.604789645
M387T381	0.604619315	0.979957136	0.604619315
M153T123	0.604546115	0.979958913	0.604546115
M114T341	0.604319182	0.979964451	0.604319182
M325T61	0.604259138	0.979965944	0.604259138
M1109T354_2	0.603628245	0.979981653	0.603628245
M669T353	0.603601498	0.979982329	0.603601498
M489T55	0.603395354	0.979987567	0.603395354
M347T48	0.603286335	0.979990352	0.603286335
M534T198	0.603157415	0.979993869	0.603157415
M843T67	0.60307346	0.979995835	0.60307346
M610T463	0.602511289	0.980010494	0.602511289
M986T167	0.602490592	0.980011085	0.602490592
M343T437	0.602391698	0.980013709	0.602391698
M135T333	0.602262622	0.980017569	0.602262622
M459T194	0.602213881	0.980018452	0.602213881

M217T246	0.602187282	0.980019164	0.602187282
M138T192	0.602106448	0.980022544	0.602106448
M216T48	0.601936562	0.980025905	0.601936562
M292T255	0.601788779	0.980029934	0.601788779
M667T354	0.601444203	0.980039381	0.601444203
M407T351	0.601293272	0.980043634	0.601293272
M401T57	0.601227276	0.980045398	0.601227276
M677T164	0.600989072	0.980052096	0.600989072
M349T87	0.60090739	0.980054731	0.60090739
M371T261	0.600880863	0.98005511	0.600880863
M806T352_1	0.600804477	0.980057241	0.600804477
M455T443	0.600627569	0.980062295	0.600627569
M868T186	0.600310593	0.980071959	0.600310593
M504T262	0.600265159	0.9800727	0.600265159
M778T33_1	0.599976293	0.980081092	0.599976293
M357T440	0.599920041	0.980082937	0.599920041
M829T155	0.599914851	0.980082892	0.599914851
M623T32	0.599463146	0.980096246	0.599463146
M420T370	0.599347892	0.980099679	0.599347892
M531T230	0.599332214	0.980100151	0.599332214
M721T35	0.599276156	0.98010267	0.599276156
M961T159	0.599212674	0.980103736	0.599212674
M983T172	0.599057509	0.980108416	0.599057509
M346T408	0.599048714	0.980109112	0.599048714
M482T259	0.598997912	0.980110503	0.598997912
M413T257	0.598769401	0.98011728	0.598769401
M193T181	0.597668858	0.980151558	0.597668858
M701T265	0.597668137	0.980151528	0.597668137
M256T111	0.597544934	0.980155458	0.597544934
M390T36	0.597519316	0.980156313	0.597519316
M348T116	0.597277758	0.98016405	0.597277758
M931T161	0.597000523	0.980173026	0.597000523
M213T362	0.596960802	0.980174322	0.596960802
M261T225	0.596772268	0.980180493	0.596772268
M202T278	0.596656214	0.980184313	0.596656214
M329T280	0.596532897	0.980188387	0.596532897
M534T112	0.596451775	0.980191115	0.596451775
M362T104	0.596416957	0.980192306	0.596416957
M456T35	0.596376304	0.980193586	0.596376304
M965T259	0.596328479	0.980195181	0.596328479
M225T47	0.596259936	0.980197469	0.596259936
M130T135	0.596087833	0.980203242	0.596087833

M716T170	0.595894406	0.980209777	0.595894406
M349T292	0.595729857	0.980215355	0.595729857
M375T98	0.595523286	0.980222415	0.595523286
M109T34	0.595491499	0.980223546	0.595491499
M97T408_2	0.594904869	0.980243842	0.594904869
M367T251	0.594901442	0.980243966	0.594901442
M131T374	0.594669371	0.98025212	0.594669371
M649T320	0.594353245	0.980263931	0.594353245
M397T56	0.594342391	0.980263722	0.594342391
M315T181_2	0.594266196	0.980266442	0.594266196
M407T246	0.594225479	0.980267901	0.594225479
M333T314	0.593928982	0.980278573	0.593928982
M113T374	0.593543083	0.980292619	0.593543083
M696T352	0.593403204	0.980297758	0.593403204
M579T170	0.593364821	0.980299167	0.593364821
M979T160	0.593299307	0.980301623	0.593299307
M225T230	0.593165185	0.980306544	0.593165185
M425T239_2	0.592932281	0.980315254	0.592932281
M480T409	0.592925417	0.980315501	0.592925417
M600T239	0.592865556	0.980320776	0.592865556
M179T169	0.592626706	0.980326686	0.592626706
M241T277	0.592535875	0.980330117	0.592535875
M986T352	0.592317666	0.980338449	0.592317666
M221T55	0.591974161	0.980351562	0.591974161
M379T255	0.591922441	0.980353555	0.591922441
M357T243_1	0.591811284	0.980357852	0.591811284
M231T32	0.591713799	0.98036163	0.591713799
M116T299	0.591684677	0.980362765	0.591684677
M393T162	0.591655274	0.980363908	0.591655274
M292T396	0.591292833	0.980378088	0.591292833
M441T205	0.591191442	0.980382082	0.591191442
M382T53	0.591176976	0.980382777	0.591176976
M398T58	0.590905197	0.980393434	0.590905197
M496T107	0.5907846	0.980398246	0.5907846
M860T49	0.590610077	0.980405242	0.590610077
M80T408	0.590433003	0.980412423	0.590433003
M317T431	0.589589857	0.980446959	0.589589857
M742T171	0.589575324	0.980447493	0.589575324
M412T379	0.589289065	0.980459419	0.589289065
M263T34_2	0.589249805	0.980461064	0.589249805
M666T300	0.58902971	0.980470315	0.58902971
M373T395	0.588654488	0.98048682	0.588654488

M503T153	0.588637652	0.980486979	0.588637652
M534T36_1	0.588560394	0.980490238	0.588560394
M404T114_1	0.588531731	0.980491471	0.588531731
M445T204	0.588530353	0.980491523	0.588530353
M773T155	0.588483893	0.98049351	0.588483893
M611T200	0.588329685	0.980500173	0.588329685
M381T96	0.588227973	0.98050452	0.588227973
M202T234	0.588217068	0.980504991	0.588217068
M362T85	0.5882093	0.980506373	0.5882093
M129T381	0.588086934	0.980510623	0.588086934
M227T98	0.587975271	0.980515478	0.587975271
M935T352	0.587853331	0.980520787	0.587853331
M286T338	0.587749227	0.980525341	0.587749227
M586T309	0.58760623	0.980531612	0.58760623
M163T35_1	0.587516717	0.980535574	0.587516717
M79T463	0.587465303	0.98053782	0.587465303
M169T374	0.587358017	0.980542566	0.587358017
M555T107	0.587313822	0.980544527	0.587313822
M367T380	0.587085898	0.980554666	0.587085898
M422T271	0.587056908	0.98055596	0.587056908
M779T149	0.586987791	0.980559052	0.586987791
M219T19	0.586489247	0.980581529	0.586489247
M675T74	0.5864808	0.980581915	0.5864808
M72T55	0.586386836	0.980586194	0.586386836
M761T490_3	0.585878589	0.980609503	0.585878589
M900T54_2	0.585824477	0.980612007	0.585824477
M218T45	0.585694206	0.980603111	0.585694206
M159T178	0.585336657	0.980634748	0.585336657
M334T169	0.585295609	0.980636674	0.585295609
M589T168_1	0.585177751	0.98064221	0.585177751
M390T44	0.585161004	0.98064301	0.585161004
M201T258	0.585152466	0.980643437	0.585152466
M407T62	0.585063865	0.980647712	0.585063865
M279T27	0.584720867	0.980666225	0.584720867
M332T336	0.584511738	0.980673893	0.584511738
M693T32_3	0.584476564	0.980675582	0.584476564
M565T212	0.584474836	0.980675666	0.584474836
M491T335	0.584412142	0.98067868	0.584412142
M588T309	0.584368635	0.980680778	0.584368635
M724T207	0.584293209	0.980684415	0.584293209
M118T247	0.584247413	0.980687153	0.584247413
M762T33	0.584217161	0.9806881	0.584217161

M792T161	0.58411699	0.980692954	0.58411699
M487T230	0.584102109	0.980693672	0.584102109
M707T196	0.5840518	0.980696115	0.5840518
M966T33	0.58402861	0.980697242	0.58402861
M671T196	0.58390519	0.980703257	0.58390519
M277T272	0.583884359	0.980704289	0.583884359
M202T300	0.583852852	0.980705815	0.583852852
M740T181	0.58371253	0.980714694	0.58371253
M655T387	0.583638174	0.980716341	0.583638174
M79T261	0.583525764	0.98072188	0.583525764
M316T73	0.58334787	0.980730676	0.58334787
M1129T354	0.583289436	0.980733576	0.583289436
M464T308	0.583251568	0.980735555	0.583251568
M400T36	0.583202901	0.980737876	0.583202901
M564T32	0.583161996	0.980739926	0.583161996
M861T41	0.582834198	0.980756318	0.582834198
M166T34	0.582812116	0.980757425	0.582812116
M261T446	0.582648352	0.98076568	0.582648352
M600T265	0.582647877	0.980765694	0.582647877
M309T292	0.58261416	0.980767417	0.58261416
M445T168_1	0.582330353	0.980781848	0.582330353
M133T426	0.582234929	0.980786689	0.582234929
M336T46	0.582102717	0.980793453	0.582102717
M439T200	0.581969968	0.980800263	0.581969968
M255T320	0.581762116	0.980810989	0.581762116
M149T42	0.581658511	0.980816353	0.581658511
M359T365	0.581649391	0.980816826	0.581649391
M327T174	0.581513681	0.980823882	0.581513681
M1145T36	0.581333547	0.98083328	0.581333547
M981T182	0.581041713	0.98084865	0.581041713
M527T110	0.580877477	0.980857285	0.580877477
M246T384	0.580733934	0.980864899	0.580733934
M357T483	0.580538644	0.980875307	0.580538644
M1159T353	0.580488837	0.980877968	0.580488837
M874T171	0.580206793	0.980893112	0.580206793
M861T354	0.579953438	0.980906813	0.579953438
M667T56	0.579945414	0.980907251	0.579945414
M77T17	0.57973988	0.98091843	0.57973988
M247T144	0.579657161	0.980922944	0.579657161
M119T169	0.579584569	0.980926956	0.579584569
M154T393	0.579435924	0.980935069	0.579435924
M423T123	0.579403551	0.980937703	0.579403551

M253T359	0.579401558	0.980937002	0.579401558
M259T147	0.579305259	0.980942273	0.579305259
M156T391	0.579289286	0.980943148	0.579289286
M469T172	0.579125395	0.980952211	0.579125395
M272T415	0.579107548	0.980953198	0.579107548
M611T51	0.579042597	0.9809568	0.579042597
M180T110	0.578837985	0.980968222	0.578837985
M503T236	0.578800077	0.980970341	0.578800077
M185T35	0.578524345	0.980985768	0.578524345
M297T36	0.578319441	0.980997365	0.578319441
M301T207	0.578270886	0.981000112	0.578270886
M263T381	0.577946257	0.981018546	0.577946257
M903T210	0.577773834	0.981028405	0.577773834
M218T446	0.57765449	0.981035675	0.57765449
M235T53	0.577502213	0.98104404	0.577502213
M253T381	0.577442512	0.981047531	0.577442512
M367T299	0.576936737	0.981076918	0.576936737
M373T499	0.576816718	0.981083956	0.576816718
M167T237	0.576778066	0.981086234	0.576778066
M433T250	0.576718804	0.981089712	0.576718804
M247T334	0.576356955	0.98111115	0.576356955
M1183T352	0.576221773	0.981119216	0.576221773
M285T137	0.576145346	0.981123736	0.576145346
M337T346	0.576036978	0.981130201	0.576036978
M415T292	0.575743405	0.981147845	0.575743405
M268T259	0.575704835	0.981150173	0.575704835
M511T352	0.575645107	0.981153835	0.575645107
M283T201	0.57491017	0.981198617	0.57491017
M583T351	0.574902199	0.981199109	0.574902199
M361T32_2	0.574845575	0.981202749	0.574845575
M313T299	0.574789553	0.981206104	0.574789553
M448T130	0.574550782	0.981220836	0.574550782
M642T35	0.574439168	0.981227854	0.574439168
M251T351	0.573948043	0.981258536	0.573948043
M129T48	0.57388717	0.981262375	0.57388717
M635T414	0.573516856	0.981285845	0.573516856
M142T34	0.573354579	0.981296195	0.573354579
M1003T179	0.573327912	0.981297903	0.573327912
M1188T292_1	0.573175143	0.981307687	0.573175143
M282T34_2	0.573076073	0.981314422	0.573076073
M788T209	0.573015643	0.98131794	0.573015643
M329T143	0.57295625	0.98132182	0.57295625

M129T403	0.5728832	0.981326538	0.5728832
M605T352	0.572637788	0.981342394	0.572637788
M473T228_1	0.57257321	0.981346593	0.57257321
M776T118	0.572514879	0.981350469	0.572514879
M723T352	0.572395452	0.981358191	0.572395452
M272T378	0.572295344	0.981364744	0.572295344
M309T44	0.572248371	0.981367825	0.572248371
M1182T352	0.572090746	0.981378194	0.572090746
M130T50	0.571950255	0.981387458	0.571950255
M282T143	0.571579429	0.981412057	0.571579429
M643T484	0.571463022	0.981419823	0.571463022
M433T168	0.57140255	0.981423872	0.57140255
M618T354	0.571125663	0.981442464	0.571125663
M550T92	0.570837778	0.981461912	0.570837778
M1182T465_3	0.570753476	0.981467683	0.570753476
M187T279	0.570545848	0.981481794	0.570545848
M1182T465_2	0.570399074	0.98149181	0.570399074
M770T76	0.570293664	0.98149904	0.570293664
M761T490_1	0.570219808	0.981504116	0.570219808
M461T409	0.57021315	0.981504577	0.57021315
M168T43	0.569995255	0.981519631	0.569995255
M130T272	0.569991606	0.981519856	0.569991606
M332T125	0.569887734	0.981527054	0.569887734
M1177T37	0.569810058	0.981532439	0.569810058
M359T259_1	0.569768011	0.981535399	0.569768011
M189T307	0.569765128	0.981535553	0.569765128
M331T381	0.569263299	0.98157071	0.569263299
M765T123	0.569201028	0.981575017	0.569201028
M300T99	0.568576152	0.981619332	0.568576152
M419T83	0.568538144	0.98162208	0.568538144
M589T234	0.568471022	0.981626848	0.568471022
M378T184	0.568408083	0.981631388	0.568408083
M636T174	0.568275301	0.981641009	0.568275301
M98T374	0.568245667	0.981643068	0.568245667
M591T161	0.568079078	0.981655079	0.568079078
M389T321	0.567859582	0.981670922	0.567859582
M424T33	0.567625844	0.98168801	0.567625844
M1003T80	0.567394375	0.981704867	0.567394375
M1124T36	0.567314296	0.981710928	0.567314296
M1041T354_2	0.5672014	0.981719078	0.5672014
M245T335	0.567100674	0.981726481	0.567100674
M761T490_2	0.56702539	0.981732712	0.56702539

M718T188	0.566952681	0.981737471	0.566952681
M143T334	0.566455521	0.981774474	0.566455521
M385T300	0.566134392	0.981798598	0.566134392
M373T484_1	0.566086634	0.98180225	0.566086634
M91T299	0.566049307	0.981805023	0.566049307
M333T272	0.565925988	0.981814377	0.565925988
M247T299	0.56554582	0.98183794	0.56554582
M366T217_1	0.565319285	0.981860644	0.565319285
M222T431	0.565309926	0.981861816	0.565309926
M381T205	0.565169118	0.98187222	0.565169118
M737T44	0.565161792	0.981872766	0.565161792
M137T33	0.565079925	0.98187908	0.565079925
M133T409	0.565045196	0.981881761	0.565045196
M1114T36	0.564977465	0.981887108	0.564977465
M236T393	0.564965612	0.981887918	0.564965612
M398T34	0.564932318	0.981890493	0.564932318
M596T169	0.564895757	0.981893337	0.564895757
M218T323	0.56484289	0.981897444	0.56484289
M228T40	0.564696292	0.981908827	0.564696292
M427T219	0.5645464	0.981920559	0.5645464
M99T408	0.56437587	0.981933934	0.56437587
M311T18	0.564325359	0.981937827	0.564325359
M293T177	0.564239268	0.981944591	0.564239268
M647T257	0.563977909	0.981965205	0.563977909
M409T33	0.563917509	0.981969989	0.563917509
M237T467	0.56379501	0.981979697	0.56379501
M159T466	0.563757338	0.981982691	0.563757338
M255T242	0.56365165	0.981991106	0.56365165
M1042T183	0.563604097	0.981994893	0.563604097
M293T392	0.563482996	0.982004561	0.563482996
M1103T339_2	0.563405073	0.982010803	0.563405073
M447T99	0.563275049	0.982021247	0.563275049
M99T24	0.56276209	0.982062669	0.56276209
M291T272	0.56270444	0.982067349	0.56270444
M1138T59	0.562644342	0.982072237	0.562644342
M231T192	0.562641524	0.982072452	0.562641524
M1037T352	0.562460972	0.982087494	0.562460972
M947T27	0.562328643	0.982098005	0.562328643
M861T90	0.562167817	0.982111216	0.562167817
M315T452	0.561893451	0.982133847	0.561893451
M1002T48	0.561609782	0.982157386	0.561609782
M433T292	0.561604576	0.982157837	0.561604576

M785T354	0.561558151	0.982161688	0.561558151
M95T237	0.561392139	0.982175601	0.561392139
M682T287	0.561331281	0.982180637	0.561331281
M221T300	0.56119644	0.982191971	0.56119644
M713T243	0.560944762	0.982213133	0.560944762
M328T168	0.560553491	0.982246394	0.560553491
M369T141	0.560383019	0.982261597	0.560383019
M864T52	0.560299093	0.982268026	0.560299093
M160T335	0.560293909	0.982268456	0.560293909
M204T298	0.560139735	0.982281672	0.560139735
M287T28	0.559768038	0.982313728	0.559768038
M582T34	0.55971593	0.982318225	0.55971593
M479T342	0.559684032	0.982321123	0.559684032
M316T321	0.559650281	0.982323928	0.559650281
M229T301	0.559435296	0.982342613	0.559435296
M677T284	0.559389752	0.982346582	0.559389752
M459T36_1	0.5590815	0.982373557	0.5590815
M1187T354_1	0.558913407	0.982390376	0.558913407
M235T221	0.558837576	0.982395026	0.558837576
M457T54	0.558514075	0.982423734	0.558514075
M290T294	0.558350705	0.982438244	0.558350705
M265T621	0.557923056	0.982476501	0.557923056
M99T463	0.557788613	0.982488609	0.557788613
M137T35_1	0.557739073	0.982493079	0.557739073
M355T123	0.557726556	0.98249421	0.557726556
M287T150	0.557606727	0.982505044	0.557606727
M254T357	0.557563389	0.982509012	0.557563389
M166T120	0.557422257	0.982521774	0.557422257
M657T27	0.556611022	0.982596198	0.556611022
M277T35	0.556447686	0.982611231	0.556447686
M341T113	0.556442972	0.982611669	0.556442972
M392T124	0.55642952	0.982612916	0.55642952
M168T149	0.556348978	0.982620391	0.556348978
M158T299	0.556332645	0.98262191	0.556332645
M609T344	0.556158129	0.982638666	0.556158129
M192T678	0.555843412	0.982667603	0.555843412
M855T125	0.555764189	0.982675049	0.555764189
M1033T352	0.555690042	0.982682024	0.555690042
M95T374	0.55544687	0.982704979	0.55544687
M391T247	0.555301693	0.982718739	0.555301693
M557T114	0.555158984	0.982732312	0.555158984
M892T33	0.555153674	0.982732806	0.555153674

M217T206	0.555111127	0.982736859	0.555111127
M553T186	0.554914982	0.982755583	0.554914982
M114T381	0.554714549	0.982774827	0.554714549
M339T354	0.554702916	0.982775914	0.554702916
M365T285	0.554362835	0.982808705	0.554362835
M875T171	0.554334693	0.982811421	0.554334693
M519T383	0.553760663	0.982867307	0.553760663
M228T116	0.553702878	0.982873052	0.553702878
M327T285	0.553473233	0.982895532	0.553473233
M507T203	0.553233966	0.982919152	0.553233966
M332T351	0.553063726	0.982936028	0.553063726
M410T274	0.552913266	0.982950992	0.552913266
M277T584	0.552793216	0.982962964	0.552793216
M225T26	0.552779623	0.982964338	0.552779623
M295T396	0.552740661	0.982968213	0.552740661
M434T84	0.55267199	0.982975083	0.55267199
M838T433	0.55238857	0.983003528	0.55238857
M483T409	0.552301237	0.983012324	0.552301237
M889T352	0.552001389	0.983042654	0.552001389
M359T208	0.551732796	0.983069971	0.551732796
M930T423_4	0.551723817	0.983070879	0.551723817
M540T114	0.551506304	0.983093112	0.551506304
M205T258	0.551464876	0.983097358	0.551464876
M231T393	0.551100391	0.983134854	0.551100391
M733T33	0.550886668	0.983157012	0.550886668
M723T82	0.550740165	0.983172193	0.550740165
M263T231	0.550572537	0.983189651	0.550572537
M251T671	0.550561765	0.98319082	0.550561765
M346T130	0.550486516	0.983198735	0.550486516
M346T104	0.550099684	0.983239225	0.550099684
M548T36	0.549954087	0.983254547	0.549954087
M434T34	0.549849066	0.983265681	0.549849066
M674T191	0.549721125	0.983279643	0.549721125
M367T38	0.549518026	0.983300836	0.549518026
M825T351	0.549336462	0.983320216	0.549336462
M531T32	0.549321941	0.983321772	0.549321941
M435T40	0.54900233	0.983361724	0.54900233
M215T328	0.548950671	0.983361617	0.548950671
M611T413	0.548928016	0.983364056	0.548928016
M869T52	0.548829759	0.983374664	0.548829759
M433T34	0.548719308	0.983386771	0.548719308
M132T264	0.548710509	0.98338756	0.548710509

M150T260	0.548676525	0.983391234	0.548676525
M717T106	0.548568484	0.983402942	0.548568484
M123T34	0.548310547	0.983431013	0.548310547
M657T33	0.547941716	0.983471404	0.547941716
M411T201	0.547920484	0.983473726	0.547920484
M180T299	0.547901616	0.9834758	0.547901616
M783T168	0.547836753	0.983482966	0.547836753
M293T301	0.547676651	0.983500588	0.547676651
M206T293	0.547528612	0.983516984	0.547528612
M357T412	0.547385986	0.98353278	0.547385986
M1131T38	0.547303139	0.983542009	0.547303139
M409T17	0.547015988	0.983574068	0.547015988
M686T56	0.546936432	0.983582931	0.546936432
M418T122	0.546924739	0.983584244	0.546924739
M303T83	0.54689459	0.983587634	0.54689459
M237T299	0.546775834	0.983600947	0.546775834
M672T235	0.546321088	0.983652282	0.546321088
M343T222	0.546056768	0.983682325	0.546056768
M881T352	0.54602516	0.983685931	0.54602516
M323T84_1	0.545595059	0.983735193	0.545595059
M145T258	0.545493151	0.983746905	0.545493151
M185T26	0.545361397	0.983762105	0.545361397
M163T33	0.545206896	0.983779998	0.545206896
M339T319	0.544862655	0.983819988	0.544862655
M762T158	0.544738747	0.983834447	0.544738747
M390T91	0.544666448	0.983842967	0.544666448
M246T128	0.544592917	0.98385152	0.544592917
M619T34	0.544384654	0.983875976	0.544384654
M787T352_2	0.544323792	0.983883148	0.544323792
M983T42	0.544219338	0.983895474	0.544219338
M754T35	0.544106468	0.983908802	0.544106468
M248T409	0.544083176	0.98391156	0.544083176
M754T32	0.543886835	0.983935178	0.543886835
M581T171	0.543809248	0.983944068	0.543809248
M368T384	0.543721304	0.983954539	0.543721304
M987T100	0.543533503	0.983976981	0.543533503
M298T284	0.543441246	0.983988	0.543441246
M549T388	0.543429997	0.983989353	0.543429997
M192T485	0.543373335	0.983996152	0.543373335
M844T188	0.543179254	0.984019502	0.543179254
M876T169	0.542980736	0.98404343	0.542980736
M255T125	0.542513161	0.984100198	0.542513161

M145T45	0.542491019	0.984102897	0.542491019
M409T307	0.542490317	0.984102988	0.542490317
M482T441	0.542487399	0.984103335	0.542487399
M603T351	0.542481119	0.984104105	0.542481119
M435T243	0.542450238	0.984107874	0.542450238
M680T33	0.542226611	0.984135292	0.542226611
M589T168_2	0.542212201	0.984137043	0.542212201
M472T103	0.54179257	0.984188674	0.54179257
M431T32_1	0.541788338	0.984189208	0.541788338
M762T329	0.541533515	0.984220782	0.541533515
M404T273	0.541515174	0.984223063	0.541515174
M497T223	0.54151516	0.984223103	0.54151516
M449T346	0.541456627	0.984230343	0.541456627
M616T35	0.54096594	0.984291706	0.54096594
M1040T352_2	0.540894384	0.984300664	0.540894384
M317T164	0.540774994	0.984315694	0.540774994
M81T334	0.540249845	0.984382294	0.540249845
M367T261	0.540224966	0.984385375	0.540224966
M427T297	0.539897108	0.984427254	0.539897108
M767T81_1	0.539786917	0.984441383	0.539786917
M678T161	0.539552411	0.984471562	0.539552411
M155T584	0.539414801	0.984489328	0.539414801
M309T352	0.539410874	0.984489836	0.539410874
M686T352	0.53924674	0.984511275	0.53924674
M603T207	0.539187098	0.984519839	0.539187098
M623T289	0.539092446	0.984531132	0.539092446
M1100T352	0.539070634	0.98453397	0.539070634
M1163T39_2	0.539043843	0.984537459	0.539043843
M284T300	0.53864232	0.984589928	0.53864232
M279T293	0.538616991	0.984593593	0.538616991
M314T113	0.538442579	0.984616183	0.538442579
M1002T352_1	0.538420149	0.984619136	0.538420149
M715T214	0.538347814	0.984628714	0.538347814
M708T413	0.538328761	0.984631556	0.538328761
M177T408	0.538067098	0.9846658	0.538067098
M757T208	0.538053269	0.984667625	0.538053269
M1046T58	0.537983702	0.984676859	0.537983702
M814T199	0.537943411	0.984682215	0.537943411
M978T160	0.537734209	0.984710068	0.537734209
M272T120	0.537227435	0.984786189	0.537227435
M593T291	0.537069232	0.984799339	0.537069232
M767T106	0.536813423	0.98483444	0.536813423

M581T381	0.536758875	0.984841421	0.536758875
M924T375	0.536024103	0.984941894	0.536024103
M550T36_2	0.535872389	0.984962826	0.535872389
M590T351	0.535823185	0.984970529	0.535823185
M728T26_2	0.535801266	0.984972787	0.535801266
M864T352	0.535744352	0.984980526	0.535744352
M201T312	0.535725056	0.984983195	0.535725056
M385T167	0.535682875	0.984989048	0.535682875
M72T298	0.535568427	0.985004967	0.535568427
M309T380	0.535533203	0.985009814	0.535533203
M423T24	0.535457254	0.985020377	0.535457254
M381T263	0.535317359	0.98504014	0.535317359
M647T416	0.535146386	0.985063773	0.535146386
M537T423	0.535098738	0.985144348	0.535098738
M287T137	0.535073876	0.98507393	0.535073876
M275T246	0.535072415	0.98507413	0.535072415
M272T108	0.535011887	0.98508262	0.535011887
M184T496	0.534906793	0.985098205	0.534906793
M117T205_1	0.534878005	0.98510143	0.534878005
M860T103	0.534145015	0.985205248	0.534145015
M335T467	0.534042043	0.98521996	0.534042043
M240T63	0.533738159	0.985263482	0.533738159
M302T114	0.533659817	0.98527478	0.533659817
M112T408	0.533640914	0.985278059	0.533640914
M357T404	0.533378661	0.985312447	0.533378661
M618T34	0.5333544	0.98531987	0.5333544
M208T56	0.53330011	0.985326667	0.53330011
M293T185	0.533275745	0.985330198	0.533275745
M406T333	0.533209688	0.985340251	0.533209688
M306T36	0.533004858	0.98537048	0.533004858
M684T272	0.532643307	0.985422397	0.532643307
M903T239	0.532619578	0.985425873	0.532619578
M447T137	0.532495913	0.985444016	0.532495913
M504T173	0.532235076	0.985482865	0.532235076
M379T210	0.532140912	0.985496357	0.532140912
M1014T53	0.532037202	0.985512764	0.532037202
M613T354	0.532016106	0.985514846	0.532016106
M317T301	0.532006776	0.985516257	0.532006776
M972T58	0.531869063	0.985536693	0.531869063
M161T44	0.531728322	0.985557644	0.531728322
M627T202	0.531584513	0.985579098	0.531584513
M316T185	0.531252748	0.985629188	0.531252748

M387T351	0.530862985	0.985687785	0.530862985
M1157T352	0.53086137	0.985688166	0.53086137
M314T243	0.530773087	0.985701283	0.530773087
M134T250	0.530525133	0.985739028	0.530525133
M406T168	0.530500803	0.985742698	0.530500803
M681T32	0.530492511	0.985743953	0.530492511
M295T453	0.530451652	0.985750187	0.530451652
M1158T353	0.530289699	0.98577513	0.530289699
M211T51	0.530189516	0.985790289	0.530189516
M348T142	0.53009333	0.985805045	0.53009333
M145T402	0.52968107	0.985868606	0.52968107
M95T34_2	0.529613857	0.985879015	0.529613857
M241T34_1	0.529544993	0.985889687	0.529544993
M731T26	0.529537499	0.985890856	0.529537499
M233T372	0.52900647	0.985973656	0.52900647
M662T174	0.528985383	0.985977731	0.528985383
M598T169	0.528856956	0.985997108	0.528856956
M243T47	0.5287105	0.986020148	0.5287105
M1049T82	0.528666172	0.986027131	0.528666172
M409T101	0.528368052	0.986074253	0.528368052
M467T326	0.528242336	0.986095268	0.528242336
M355T385	0.528055823	0.986124193	0.528055823
M875T180	0.527650528	0.986188733	0.527650528
M615T205	0.527582064	0.986199739	0.527582064
M522T460	0.527503732	0.986212344	0.527503732
M687T194	0.527360098	0.9862355	0.527360098
M417T104	0.527242332	0.986254532	0.527242332
M144T259	0.527213383	0.986259216	0.527213383
M245T34_2	0.527076564	0.986281392	0.527076564
M779T53	0.52703884	0.986287609	0.52703884
M354T169	0.527032731	0.986288508	0.527032731
M830T51	0.527025945	0.986289606	0.527025945
M1012T352_2	0.526826302	0.986322092	0.526826302
M709T198	0.52671746	0.986339925	0.52671746
M431T292	0.526611016	0.986357292	0.526611016
M402T103	0.526404901	0.986391056	0.526404901
M237T95	0.526216991	0.986472761	0.526216991
M148T370	0.526141537	0.986434417	0.526141537
M591T207	0.525809598	0.986489371	0.525809598
M593T408	0.525379431	0.98656108	0.525379431
M290T60	0.525299125	0.986574534	0.525299125
M340T35_2	0.524952299	0.986632835	0.524952299

M200T257	0.524829305	0.986653599	0.524829305
M213T380	0.524726538	0.98667099	0.524726538
M532T179	0.524678838	0.986679069	0.524678838
M459T32	0.524469573	0.986714604	0.524469573
M289T351	0.524420279	0.986723008	0.524420279
M714T58	0.524233459	0.986756876	0.524233459
M226T50	0.523840077	0.986822311	0.523840077
M359T200	0.523788138	0.986831256	0.523788138
M409T345	0.523715636	0.986843768	0.523715636
M246T426	0.523684885	0.986849067	0.523684885
M204T53	0.523463899	0.986887275	0.523463899
M960T115	0.523308966	0.986914154	0.523308966
M1020T24	0.523265419	0.986921721	0.523265419
M183T370	0.523231433	0.986927633	0.523231433
M201T389	0.523151143	0.986941615	0.523151143
M322T231	0.522998833	0.986968188	0.522998833
M147T105	0.522982222	0.986971092	0.522982222
M1043T36	0.522853868	0.98699175	0.522853868
M763T354	0.522675209	0.987024891	0.522675209
M234T354	0.522667342	0.987026266	0.522667342
M506T345	0.522509632	0.987054046	0.522509632
M830T53	0.52240797	0.987071976	0.52240797
M982T171	0.522312989	0.98708877	0.522312989
M816T69	0.522185721	0.987111311	0.522185721
M162T46	0.522148065	0.987117987	0.522148065
M272T320	0.522038679	0.98713742	0.522038679
M992T170	0.521989271	0.987146199	0.521989271
M93T231	0.521950425	0.987153119	0.521950425
M435T272	0.521867579	0.987168013	0.521867579
M933T336	0.521722257	0.987193836	0.521722257
M381T257	0.521432629	0.987245759	0.521432629
M521T385	0.52123611	0.987281147	0.52123611
M241T304	0.520942694	0.987334212	0.520942694
M397T292	0.52093248	0.987336067	0.52093248
M127T320_1	0.52085069	0.98735091	0.52085069
M966T25	0.52084551	0.987351853	0.52084551
M1006T178	0.520820122	0.987356917	0.520820122
M406T305	0.520780503	0.987363715	0.520780503
M763T255	0.520705218	0.987377369	0.520705218
M478T408	0.520523887	0.987410451	0.520523887
M968T26	0.520415358	0.987430306	0.520415358
M797T198	0.520379694	0.987436834	0.520379694

M284T304	0.520356481	0.987441143	0.520356481
M613T171	0.520324923	0.987446887	0.520324923
M355T311	0.520053457	0.987496777	0.520053457
M129T442	0.520038555	0.987499571	0.520038555
M337T401	0.520034475	0.987500279	0.520034475
M675T292	0.520026387	0.987501767	0.520026387
M353T381_1	0.519976188	0.987511029	0.519976188
M741T239	0.519891778	0.987527001	0.519891778
M315T344	0.519850355	0.987534251	0.519850355
M1050T168	0.519722146	0.987558015	0.519722146
M267T63_1	0.519641976	0.987572883	0.519641976
M1030T354	0.518753595	0.987851401	0.518753595
M315T509	0.518499286	0.987787253	0.518499286
M269T334	0.51847266	0.987792385	0.51847266
M870T200	0.518224105	0.987839538	0.518224105
M598T408	0.51745329	0.987987399	0.51745329
M506T407	0.517176375	0.988041059	0.517176375
M156T256	0.517147894	0.988046554	0.517147894
M292T483_1	0.516727909	0.988128478	0.516727909
M239T233	0.51651791	0.988169624	0.51651791
M174T369	0.516463456	0.988180366	0.516463456
M784T352	0.51640678	0.988191475	0.51640678
M395T55	0.516244621	0.988223448	0.516244621
M711T26_2	0.516169196	0.988238435	0.516169196
M810T32	0.516138535	0.988244408	0.516138535
M666T169	0.516131686	0.988250785	0.516131686
M869T39	0.516092527	0.988253511	0.516092527
M441T303	0.515844362	0.988302759	0.515844362
M872T39	0.515357896	0.988399942	0.515357896
M273T19	0.515185193	0.988435135	0.515185193
M841T56	0.515160166	0.988439675	0.515160166
M230T89	0.51507208	0.988457669	0.51507208
M129T181_2	0.514873779	0.988497498	0.514873779
M924T35	0.514819428	0.988508496	0.514819428
M323T32	0.51454832	0.988563556	0.51454832
M366T310	0.514284859	0.988617332	0.514284859
M298T289	0.51423428	0.988627682	0.51423428
M776T94	0.513848537	0.988706955	0.513848537
M1191T172	0.513744604	0.988728405	0.513744604
M294T105	0.513615989	0.988755022	0.513615989
M215T31	0.513605962	0.988757041	0.513605962
M144T258	0.51343657	0.988792225	0.51343657

M247T381	0.51327846	0.988825124	0.51327846
M135T93	0.513103634	0.988861623	0.513103634
M896T57	0.512730197	0.988939935	0.512730197
M702T40	0.512616595	0.988963869	0.512616595
M309T316	0.512482009	0.988992279	0.512482009
M801T250_2	0.512373687	0.989015203	0.512373687
M900T38	0.512326709	0.989025102	0.512326709
M1070T354_2	0.512300829	0.989030647	0.512300829
M615T235	0.512061057	0.98908161	0.512061057
M298T167	0.511843376	0.989128088	0.511843376
M457T200	0.511692095	0.989160488	0.511692095
M527T146	0.511521403	0.989197141	0.511521403
M168T495	0.511514443	0.98919864	0.511514443
M266T256	0.511392602	0.989224888	0.511392602
M365T409	0.51121565	0.989263111	0.51121565
M743T148	0.510940316	0.989323056	0.510940316
M95T348	0.510768242	0.989360329	0.510768242
M220T117	0.510762478	0.989361559	0.510762478
M219T263	0.510710596	0.989373819	0.510710596
M396T176	0.510662603	0.989374449	0.510662603
M167T42	0.51063813	0.989388712	0.51063813
M501T159	0.510414826	0.989437817	0.510414826
M375T36	0.510404075	0.989440001	0.510404075
M581T234	0.510393505	0.98944232	0.510393505
M731T78	0.510257113	0.98947232	0.510257113
M405T73	0.510112724	0.98950415	0.510112724
M588T36	0.510053611	0.989517211	0.510053611
M229T371	0.509981245	0.989533468	0.509981245
M1007T177	0.509588579	0.989620414	0.509588579
M460T317	0.508829393	0.989790827	0.508829393
M557T24	0.508828038	0.989791116	0.508828038
M1011T79	0.508752926	0.989808423	0.508752926
M390T56	0.508668061	0.989827335	0.508668061
M155T251	0.508647434	0.989832016	0.508647434
M934T352_2	0.508550571	0.989853967	0.508550571
M186T135	0.508447726	0.989877396	0.508447726
M825T52	0.508377759	0.98989334	0.508377759
M266T63	0.508305898	0.989909711	0.508305898
M491T254	0.508213436	0.989930809	0.508213436
M849T129	0.50817699	0.989939132	0.50817699
M740T79	0.507937569	0.989994266	0.507937569
M352T138	0.507742979	0.990039081	0.507742979

M626T33	0.507723636	0.990043307	0.507723636
M1187T292	0.507581181	0.990076236	0.507581181
M262T112	0.507507146	0.990093355	0.507507146
M506T169	0.507435942	0.990109854	0.507435942
M440T169	0.507235169	0.99015649	0.507235169
M317T407	0.507217403	0.990160631	0.507217403
M546T35	0.507139674	0.990178743	0.507139674
M588T114	0.507095702	0.990189025	0.507095702
M1021T52	0.507056361	0.990198183	0.507056361
M127T35_1	0.507051009	0.990199435	0.507051009
M452T200	0.506758146	0.990268015	0.506758146
M980T168_2	0.506669537	0.990288847	0.506669537
M1076T318_3	0.506573984	0.99031134	0.506573984
M100T78	0.506416162	0.990348584	0.506416162
M263T115	0.505887739	0.990474079	0.505887739
M853T50	0.505815547	0.990491321	0.505815547
M1028T34	0.505754284	0.990506146	0.505754284
M476T317	0.505686219	0.990522248	0.505686219
M253T37	0.505681764	0.990523364	0.505681764
M276T41	0.505220182	0.990634393	0.505220182
M235T406	0.504761075	0.990746065	0.504761075
M147T704	0.504552921	0.990797389	0.504552921
M257T337	0.504539581	0.990799901	0.504539581
M859T49	0.504439934	0.990824313	0.504439934
M333T246	0.504296829	0.990859445	0.504296829
M155T334	0.504281957	0.990863127	0.504281957
M725T131	0.504267058	0.990866772	0.504267058
M729T81	0.504202169	0.990882737	0.504202169
M100T59	0.504183732	0.99088727	0.504183732
M1002T167	0.504150555	0.990895471	0.504150555
M1060T353_2	0.504114477	0.990904319	0.504114477
M717T153	0.50382281	0.990976782	0.50382281
M371T233	0.503664238	0.991015891	0.503664238
M830T33	0.503650785	0.991019694	0.503650785
M812T49_2	0.503537829	0.99104738	0.503537829
M405T50	0.503372406	0.991088702	0.503372406
M347T123	0.503368699	0.991089632	0.503368699
M279T373	0.503343736	0.991095869	0.503343736
M495T201	0.503326312	0.991100238	0.503326312
M719T54	0.503271241	0.991115289	0.503271241
M130T242	0.503181709	0.991136978	0.503181709
M549T344	0.503132738	0.991148783	0.503132738

M561T210	0.503033783	0.991173683	0.503033783
M799T53	0.502866063	0.991215966	0.502866063
M359T234	0.502825755	0.991226149	0.502825755
M183T299	0.50267844	0.991263464	0.50267844
M430T345	0.502478126	0.991314338	0.502478126
M267T286	0.502345356	0.991348125	0.502345356
M702T211	0.502257074	0.991370685	0.502257074
M335T161	0.501818949	0.991483053	0.501818949
M291T321	0.501551899	0.991552009	0.501551899
M658T35	0.501486472	0.991568979	0.501486472
M187T301	0.501436334	0.991581977	0.501436334
M726T52	0.501402917	0.991590657	0.501402917
M840T209	0.501348042	0.991604911	0.501348042
M189T59	0.500999847	0.991695736	0.500999847
M399T41	0.500997297	0.99169639	0.500997297
M449T28	0.500858921	0.991732642	0.500858921
M505T159	0.500382317	0.991858279	0.500382317
M85T260	0.500283138	0.991884548	0.500283138
M233T33	0.500258386	0.99189111	0.500258386
M417T332	0.500018065	0.991955046	0.500018065
M596T309	0.500013921	0.991956151	0.500013921
M690T499_1	0.499953289	0.991972373	0.499953289
M822T68	0.499802469	0.992012648	0.499802469
M397T320	0.499753455	0.992025822	0.499753455
M239T210	0.499735628	0.992030561	0.499735628
M387T136	0.499592878	0.992068879	0.499592878
M217T234	0.499470041	0.9921019	0.499470041
M459T123	0.499300595	0.992147671	0.499300595
M1089T352_2	0.499115023	0.992197934	0.499115023
M876T97	0.499075973	0.992209537	0.499075973
M739T171	0.498743367	0.992299136	0.498743367
M178T262	0.498718308	0.992305972	0.498718308
M526T145	0.498690715	0.992313559	0.498690715
M545T97	0.498529253	0.99235776	0.498529253
M409T204	0.498506891	0.99236391	0.498506891
M414T224	0.498186051	0.992452258	0.498186051
M246T336	0.497523315	0.992636575	0.497523315
M949T236	0.497434046	0.992661538	0.497434046
M301T42	0.497313902	0.992695295	0.497313902
M846T187	0.497216084	0.992722785	0.497216084
M262T497	0.497054636	0.992768309	0.497054636
M837T68_2	0.49685248	0.992825518	0.49685248

M119T34	0.496709094	0.992866198	0.496709094
M595T337	0.496507356	0.992923727	0.496507356
M1053T35	0.496409961	0.992951563	0.496409961
M421T212	0.496242883	0.992999741	0.496242883
M365T52	0.496097466	0.993041233	0.496097466
M1181T465_3	0.495968123	0.993078513	0.495968123
M685T199	0.495906297	0.993096363	0.495906297
M374T115	0.495480492	0.99321992	0.495480492
M423T232	0.494808824	0.993417336	0.494808824
M1091T353	0.494807511	0.993417379	0.494807511
M415T54	0.494606022	0.993477025	0.494606022
M653T33	0.494586291	0.993482871	0.494586291
M370T319	0.494499556	0.993508742	0.494499556
M259T34	0.494416169	0.993533435	0.494416169
M487T121	0.494092612	0.993630089	0.494092612
M885T39	0.493948671	0.993673285	0.493948671
M936T161	0.493948472	0.9936733	0.493948472
M239T42	0.49389058	0.993690707	0.49389058
M342T389	0.493868734	0.993697295	0.493868734
M251T238	0.493842204	0.993705272	0.493842204
M203T36	0.493772513	0.993726322	0.493772513
M163T111	0.493728239	0.993739776	0.493728239
M595T409	0.493606797	0.993776399	0.493606797
M231T378	0.493599818	0.993778519	0.493599818
M138T4	0.493522918	0.99380188	0.493522918
M361T361	0.49337719	0.993846083	0.49337719
M173T334	0.493265583	0.993880045	0.493265583
M1076T318_1	0.493224104	0.993892712	0.493224104
M904T40	0.492847672	0.994007959	0.492847672
M293T119	0.49278953	0.99402585	0.49278953
M117T706	0.492328905	0.994168314	0.492328905
M293T25	0.492200514	0.99420827	0.492200514
M245T41	0.492195304	0.994209885	0.492195304
M689T124	0.491757015	0.994347104	0.491757015
M515T36	0.491693936	0.994366996	0.491693936
M290T121	0.491652861	0.994379894	0.491652861
M791T200	0.491521745	0.994421275	0.491521745
M580T74	0.49143998	0.99444713	0.49143998
M708T32	0.491352839	0.994474758	0.491352839
M401T247	0.491117625	0.994549547	0.491117625
M609T275	0.490829244	0.994641753	0.490829244
M250T300	0.490599976	0.994715458	0.490599976

M155T35_2	0.490403178	0.994779023	0.490403178
M255T466	0.490234059	0.994833871	0.490234059
M409T363	0.490187944	0.994848848	0.490187944
M787T72	0.490123485	0.994869915	0.490123485
M312T128	0.489742515	0.994994413	0.489742515
M455T201	0.489669416	0.995018411	0.489669416
M307T247	0.489494752	0.995075984	0.489494752
M576T408	0.489361138	0.995120121	0.489361138
M256T376	0.489240009	0.995160865	0.489240009
M529T114	0.489102013	0.995206161	0.489102013
M681T33	0.488998049	0.995240787	0.488998049
M236T374	0.488959695	0.9952536	0.488959695
M603T152	0.488661242	0.99535355	0.488661242
M1023T188	0.488580934	0.995380626	0.488580934
M873T171	0.488359787	0.995455292	0.488359787
M545T62	0.488309251	0.995464003	0.488309251
M237T408	0.488205034	0.995507702	0.488205034
M916T30	0.488081732	0.995549691	0.488081732
M1008T180	0.488054869	0.995623886	0.488054869
M586T312	0.487559646	0.995728551	0.487559646
M676T465_2	0.487536682	0.995736455	0.487536682
M491T136	0.487404687	0.99578321	0.487404687
M597T336	0.48723225	0.995841799	0.48723225
M649T409	0.486616877	0.996056952	0.486616877
M399T53	0.48628851	0.996173028	0.48628851
M504T37	0.486221362	0.996196878	0.486221362
M181T231	0.48621072	0.996200747	0.48621072
M645T200	0.486095474	0.996241688	0.486095474
M203T430	0.485954486	0.99629218	0.485954486
M505T28	0.485445682	0.996476419	0.485445682
M465T37	0.485329687	0.996517172	0.485329687
M296T83	0.485174606	0.996573605	0.485174606
M215T207	0.485048541	0.996619597	0.485048541
M274T401	0.484812914	0.996706304	0.484812914
M134T65	0.484773896	0.996720328	0.484773896
M688T352_1	0.484037047	0.996993911	0.484037047
M201T261	0.484031623	0.996996009	0.484031623
M246T554	0.483320267	0.997266495	0.483320267
M449T105	0.483214919	0.997305357	0.483214919
M483T58	0.483195777	0.997312705	0.483195777
M142T113	0.482960379	0.997403132	0.482960379
M298T34_2	0.482921772	0.997418012	0.482921772

M148T286	0.482809627	0.99746133	0.482809627
M1108T354	0.482781641	0.997472107	0.482781641
M471T354	0.482671786	0.997514695	0.482671786
M260T105	0.482614506	0.997537025	0.482614506
M590T352	0.482610484	0.997538522	0.482610484
M267T209	0.482607344	0.997539816	0.482607344
M543T60	0.482388003	0.99761627	0.482388003
M104T235	0.482246455	0.997681339	0.482246455
M360T200_1	0.482088901	0.997742818	0.482088901
M330T253	0.48200209	0.997777176	0.48200209
M278T126	0.481775319	0.997867125	0.481775319
M275T145	0.481657516	0.997914076	0.481657516
M118T506	0.481410889	0.998012875	0.481410889
M163T415	0.481385837	0.998022953	0.481385837
M198T259	0.481379163	0.998025641	0.481379163
M346T319	0.481215506	0.998091648	0.481215506
M813T39	0.481006783	0.998176368	0.481006783
M226T35	0.480933926	0.99820587	0.480933926
M747T78	0.480908197	0.998216432	0.480908197
M470T34	0.480866705	0.99823336	0.480866705
M411T37	0.480772339	0.998271793	0.480772339
M293T32	0.480447242	0.998405715	0.480447242
M292T436	0.480404718	0.998423326	0.480404718
M461T411	0.479877459	0.998643345	0.479877459
M417T170	0.4798701	0.998646437	0.4798701
M711T193	0.479424424	0.998835207	0.479424424
M263T408	0.479036029	0.999001939	0.479036029
M528T34	0.478941287	0.999043004	0.478941287
M801T403	0.478728225	0.99913564	0.478728225
M296T53	0.478586907	0.999197594	0.478586907
M163T274	0.478483218	0.999242857	0.478483218
M281T254	0.478434262	0.999264301	0.478434262
M277T45	0.478390254	0.999283844	0.478390254
M447T39	0.478219124	0.999359322	0.478219124
M420T166	0.478139185	0.999394746	0.478139185
M490T169	0.478018561	0.999448257	0.478018561
M188T345	0.47792468	0.999490142	0.47792468
M670T199	0.47762021	0.999626129	0.47762021
M402T34	0.477606216	0.999632423	0.477606216
M150T59	0.477601345	0.99963457	0.477601345
M387T258	0.477093307	0.999859462	0.477093307
M137T272	0.477039487	0.999882294	0.477039487

M323T116	0.476833347	0.999964745	0.476833347
M268T167	0.476754061	0.999990519	0.476754061
M269T101	0.476750592	0.99999152	0.476750592
M74T181	0.472658862	2.227567952	0.472658862
M497T107	0.452916796	2.611166025	0.452916796
M471T182	0.420909908	1.373896321	0.420909908
M181T53_2	0.412173751	1.389382071	0.412173751
M327T392	0.336463001	82.44592876	0.336463001

Table S8. Selective annotated metabolites.

Metabolite ID	Classification	Description	KEGG.ID	Wb-NE vs Wb-T	Wb-T vs Cb-C	Common
M166T177	Alkaloid	Hordenine	C06199	√		
M152T230	Alkaloid	N-Methyltyramine		√		
M159T178	Amines	Allantoin	C01551			√
M157T181	Amines	Allantoin	C01551	√		
M177T242	Amines	Serotonin	C00780			√
M150T167	Amines	Triethanolamine	C06771			√
M100T59	Amines	Cyclohexylamine	C00571		√	
M184T496	Amines	Phosphorylcholine	C00588		√	
M118T2722	Amines	Betaine	C00719		√	
M136T299	Amines	Dopamine	C03758		√	
M116T309	Amino acid and derivative	D-Proline	C00763			√
M132T168	Amino acid and derivative	L-Leucine	C00123			√
M114T310	Amino acid and derivative	L-Proline	C00148			√
M120T365	Amino acid and derivative	L-Threonine	C00188	√		
M557T304	Amino acid and derivative	Lys-Leu				√
M246T403	Amino acid and derivative	Lys-Val		√		
M217T370	Amino acid and derivative	N-.alpha.-Acetyl-L-arginine		√		
M191T306	Amino acid and derivative	Thr-Ala		√		
M233T241	Amino acid and derivative	Thr-Leu		√		
M267T220	Amino acid and derivative	Thr-Phe		√		
M330T255	Amino acid and derivative	Tyr-Met		√		
M298T499	Amino acid and derivative	Val-Tyr				√

M219T19	Amino acid and derivative	BHT				√
M215T371	Amino acid and derivative	N-.alpha.-Acetyl-L-arginine		√		
M205T258	Amino acid and derivative	L-Tryptophan	C00078		√	
M148T386	Amino acid and derivative	L-Glutamate	C00025		√	
M232T452	Amino acid and derivative	N-(omega)-Hydroxyarginine	C05933		√	
M275T439	Amino acid and derivative	gamma-L-Glutamyl-L-glutamic acid	C05282		√	
M146T385	Amino acid and derivative	L-Glutamate	C00025		√	
M130T272	Amino acid and derivative	L-Isoleucine	C00407		√	
M116T299	Amino acid and derivative	L-Valine	C00183		√	
M130T363	Amino acid and derivative	N-Acetyl-L-alanine			√	
M133T374	Amino acid and derivative	L-Asparagine	C00152		√	
M182T299	Amino acid and derivative	L-Tyrosine	C00082		√	
M131T374	Amino acid and derivative	L-Asparagine	C00152		√	
M218T446	Amino acid and derivative	Ala-Lys			√	
M246T426	Amino acid and derivative	Arg-Ala			√	
M219T262	Amino acid and derivative	Ser-Ile			√	
M260T281	Amino acid and derivative	Ile-Ser			√	
M234T444	Amino acid and derivative	Lys-Ser			√	
M248T444	Amino acid and derivative	Lys-Thr			√	
M294T254	Amino acid and derivative	Phe-Gln			√	
M287T137	Amino acid and derivative	Prilocaine			√	
M187T335	Amino acid and derivative	Pro-Ala			√	
M272T415	Amino acid and derivative	Pro-Arg			√	
M207T326	Amino acid and derivative	Ser-Thr			√	
M205T286	Amino acid and derivative	Ser-Val			√	

M248T427	Amino acid and derivative	Thr-Lys			√	
M386T155	Amino acid and derivative	Tyr-Gln			√	
M246T274	Amino acid and derivative	Val-Gln			√	
M231T192	Amino acid and derivative	Val-Ile			√	
M217T206	Amino acid and derivative	Val-Val			√	
M187T328	Carboxylic Acids	Azelaic acid	C08261			√
M136T286	Carboxylic Acids	Anthranilic acid (Vitamin L1)	C00108	√		
M160T397	Carboxylic Acids	DL-2-Aminoadipic acid	C00956			√
M129T442	Carboxylic Acids	Mesaconic acid	C01732		√	
M162T398	Carboxylic Acids	DL-2-Aminoadipic acid	C00956		√	
M149T31	Carboxylic Acids	1,2-Benzenedicarboxylic acid			√	
M171T268	Carboxylic Acids	3-Dehydroshikimic acid			√	
M114T374	Carboxylic Acids	Maleamic acid			√	
M298T319	Glycosides	1-methylguanosine				√
M282T294	Glycosides	1-Methyladenosine	C02494		√	
M284T260	Glycosides	Guanosine	C00387		√	
M282T129	Glycosides	N6-methyladenosine			√	
M245T157	Glycosides	Uridine	C00299		√	
M282T260	Glycosides	Guanosine	C00387		√	
M257T140	Glycosides	Ribothymidine			√	
M303T158	Glycosides	Uridine	C00299		√	
M549T388	Glycosides	Gemcitabine			√	
M336T68	Glycosides	Isopentenyladenosine			√	
M298T284	Glycosides	8-hydroxy Guanosine			√	
M268T167	Glycosides	Adenosine	C00212		√	

M263T342	Lipids	Linoleic acid	C01595			√
M255T45	Lipids	Palmitic acid	C00249	√		
M283T44	Lipids	Stearic acid	C01530			√
M281T101	Lipids	Oleic acid	C00712	√		
M454T201	Lipids	1-Palmitoyl-2-hydroxy-sn-glycero-3-phosphoethanolamine				√
M496T196	Lipids	1-Palmitoyl-sn-glycero-3-phosphocholine				√
M452T201	Lipids	1-Palmitoyl-2-hydroxy-sn-glycero-3-phosphoethanolamine				√
M313T158	Lipids	1-Palmitoylglycerol		√		
M343T34	Lipids	20-Hydroxyarachidonic acid		√		
M330T39	Lipids	Eicosapentaenoic Acid ethyl ester				√
M337T244	Lipids	MG(18:2(9Z,12Z)/0:0/0:0)[rac]				√
M819T136	Lipids	2-Oleoyl-1-palmitoyl-sn-glycero-3-phosphocholine(PC(16:0/18:1(9Z)))		√		
M339T31	Lipids	Norethindrone Acetate				√
M275T56	Lipids	Stearidonic Acid	C00262	√		
M339T402	Lipids	Behenic acid	C08281		√	
M253T37	Lipids	cis-9-Palmitoleic acid	C08362		√	
M277T45	Lipids	all cis-(6,9,12)-Linolenic acid	C06426		√	
M137T213	Nucleic acids and derivative	Hypoxanthine	C04779	√		
M324T424	Nucleic acids and derivative	3'-O-methylcytidine		√		

M323T423	Nucleic acids and derivative	5'-CMP		√		
M282T92	Nucleic acids and derivative	2'-O-methyladenosine	C04779			√
M152T2602	Nucleic acids and derivative	2-Hydroxyadenine			√	
M112T237	Nucleic acids and derivative	Cytosine	C00380		√	
M113T158	Nucleic acids and derivative	Uracil	C00106		√	
M113T374	Nucleic acids and derivative	Dihydrouracil	C00429		√	
M111T159	Nucleic acids and derivative	Uracil	C00106		√	
M330T253	Nucleic acids and derivative	Adenosine 2',3'-cyclic monophosphate	C02353		√	
M244T238	Nucleic acids and derivative	Cytidine	C00475		√	
M146T267	Other	Oxyquinoline				
M391T247	Other	RU-0211				
M301T23	Other	Diosmetin	C00811			
M329T100	Other	Acetylvalerenolic acid				
M128T386	Other	Ammelide				
M233T33	Other	Confertifoline				
M166T256	Phenylpropanoids	L-Phenylalanine	C00079		√	

M164T256	Phenylpropanoids	L-Phenylalanine	C00079		√	
M147T299	Phenylpropanoids	4-Hydroxycinnamic acid	C00811			√
M565T212	Phenylpropanoids	Apiin	C04858		√	
M563T213	Phenylpropanoids	APIIN	C04858		√	
M173T181	Plant hormone	3-Methylindole	C05235	√		
M220T117	Plant hormone	trans-Zeatin	C00371			√
M170T258	Plant hormone	Indoleacrylic acid			√	
M87T374	Plant hormone	4-Hydroxybutanoic acid lactone			√	
M300T432	Sugar and derivative	N-Acetylglucosamine 1-phosphate		√		
M522T189	Sugar and derivative	1-Oleoyl-sn-glycero-3-phosphocholine				√
M524T192	Sugar and derivative	1-Stearoyl-2-hydroxy-sn-glycero-3-phosphocholine				√
M435T243	Sugar and derivative	1-Oleoyl-L-.alpha.-lysophosphatidic acid				√
M177T105	Sugar and derivative	L-Gulonic gamma-lactone	C01040		√	
M198T259	Sugar and derivative	D-Mannose	C00159		√	
M667T485	Sugar and derivative	Stachyose	C01613		√	
M193T311	Sugar and derivative	2'-Deoxy-D-ribose	C01801		√	
M147T180	Sugar and derivative	Hydroxyacetone	C05235			√
M151T228	Sugar and derivative	Xylitol	C00379		√	
M757T39	Sugar and derivative	PC(16:0/16:0)	C00157		√	
M173T334	Sugar and derivative	Glycerol 3-phosphate	C00093		√	
M759T391	Sugar and derivative	Thioetheramide-PC			√	
M409T251	Sugar and derivative	1-Palmitoyl Lysophosphatidic Acid				√
M237T356	Sugar and derivative	2-Dehydro-3-deoxy-D-gluconate	C00204			√

M124T206	Vitamins	Nicotinate	C00253		√	
M122T207	Vitamins	Nicotinate	C00253		√	

Table S9. Selective annotated metabolites between Wb-NE and Wb-T.

Metabolite ID	Classification	Description	KEGG.ID
M166T177	Alkaloid	Hordenine	C06199
M152T230	Alkaloid	N-Methyltyramine	
M159T178	Amines	Allantoin	C01551
M157T181	Amines	Allantoin	C01551
M177T242	Amines	Serotonin	C00780
M150T167	Amines	Triethanolamine	C06771
M116T309	Amino acid and derivative	D-Proline	C00763
M132T168	Amino acid and derivative	L-Leucine	C00123
M114T310	Amino acid and derivative	L-Proline	C00148
M120T365	Amino acid and derivative	L-Threonine	C00188
M557T304	Amino acid and derivative	Lys-Leu	
M246T403	Amino acid and derivative	Lys-Val	
M217T370	Amino acid and derivative	N-.alpha.-Acetyl-L-arginine	
M191T306	Amino acid and derivative	Thr-Ala	
M233T241	Amino acid and derivative	Thr-Leu	
M267T220	Amino acid and derivative	Thr-Phe	
M330T255	Amino acid and derivative	Tyr-Met	
M298T499	Amino acid and derivative	Val-Tyr	
M219T19	Amino acid and derivative	BHT	
M215T371	Amino acid and derivative	N-.alpha.-Acetyl-L-arginine	
M187T328	Carboxylic Acids	Azelaic acid	C08261
M136T286	Carboxylic Acids	Anthranilic acid (Vitamin L1)	C00108
M160T397	Carboxylic Acids	DL-2-Aminoadipic acid	C00956
M298T319	Glycosides	1-methylguanosine	
M263T342	Lipids	Linoleic acid	C01595
M255T45	Lipids	Palmitic acid	C00249
M283T44	Lipids	Stearic acid	C01530
M281T101	Lipids	Oleic acid	C00712
M454T201	Lipids	1-Palmitoyl-2-hydroxy-sn-glycero-3-phosphoethanolamine	
M496T196	Lipids	1-Palmitoyl-sn-glycero-3-phosphocholine	
M452T201	Lipids	1-Palmitoyl-2-hydroxy-sn-glycero-3-phosphoethanolamine	
M313T158	Lipids	1-Palmitoylglycerol	
M343T34	Lipids	20-Hydroxyarachidonic acid	
M330T39	Lipids	Eicosapentaenoic Acid ethyl ester	
M337T244	Lipids	MG(18:2(9Z,12Z)/0:0/0:0)[rac]	
M819T136	Lipids	2-Oleoyl-1-palmitoyl-sn-glycero-3-phosphocholine(PC(16:0/18:1(9Z)))	

M339T31	Lipids	Norethindrone Acetate	
M275T56	Lipids	Stearidonic Acid	C00262
M137T213	Nucleic acids and derivative	Hypoxanthine	C04779
M282T92	Nucleic acids and derivative	2'-O-methyladenosine	
M324T424	Nucleic acids and derivative	3'-O-methylcytidine	
M323T423	Nucleic acids and derivative	5'-CMP	
M146T267	Other	Oxyquinoline	
M391T247	Other	RU-0211	
M301T23	Other	Diosmetin	C00811
M147T299	Phenylpropanoids	4-Hydroxycinnamic acid	C00371
M220T117	Plant hormone	trans-Zeatin	C08313
M173T181	Plant hormone	3-Methylindole	C05235
M147T180	Sugar and derivative	Hydroxyacetone	C04256
M300T432	Sugar and derivative	N-Acetylglucosamine 1-phosphate	
M522T189	Sugar and derivative	1-Oleoyl-sn-glycero-3-phosphocholine	
M524T192	Sugar and derivative	1-Stearoyl-2-hydroxy-sn-glycero-3-phosphocholine	
M435T243	Sugar and derivative	1-Oleoyl-L-.alpha.-lysophosphatidic acid	
M409T251	Sugar and derivative	1-Palmitoyl Lysophosphatidic Acid	C00204
M237T356	Sugar and derivative	2-Dehydro-3-deoxy-D-gluconate	

Table S10. Selective annotated metabolites between Wb-T and Cb-C.

Metabolite ID	Classification	Description	KEGG.ID
M159T178	Amines	Allantoin	C01551
M177T242	Amines	Serotonin	C00780
M150T167	Amines	Triethanolamine	C06771
M100T59	Amines	Cyclohexylamine	C00571
M184T496	Amines	Phosphorylcholine	C00588
M118T2722	Amines	Betaine	C00719
M136T299	Amines	Dopamine	C03758
M205T258	Amino acid and derivative	L-Tryptophan	C00078
M116T309	Amino acid and derivative	D-Proline	C00763
M148T386	Amino acid and derivative	L-Glutamate	C00025
M132T168	Amino acid and derivative	L-Leucine	C00123
M232T452	Amino acid and derivative	N-(omega)-Hydroxyarginine	C05933
M275T439	Amino acid and derivative	gamma-L-Glutamyl-L-glutamic acid	C05282
M146T385	Amino acid and derivative	L-Glutamate	C00025
M130T272	Amino acid and derivative	L-Isoleucine	C00407
M114T310	Amino acid and derivative	L-Proline	C00148
M116T299	Amino acid and derivative	L-Valine	C00183

M130T363	Amino acid and derivative	N-Acetyl-L-alanine	
M133T374	Amino acid and derivative	L-Asparagine	C00152
M182T299	Amino acid and derivative	L-Tyrosine	C00082
M131T374	Amino acid and derivative	L-Asparagine	C00152
M218T446	Amino acid and derivative	Ala-Lys	
M246T426	Amino acid and derivative	Arg-Ala	
M219T262	Amino acid and derivative	Ser-Ile	
M260T281	Amino acid and derivative	Ile-Ser	
M557T304	Amino acid and derivative	Lys-Leu	
M234T444	Amino acid and derivative	Lys-Ser	
M248T444	Amino acid and derivative	Lys-Thr	
M294T254	Amino acid and derivative	Phe-Gln	
M287T137	Amino acid and derivative	Prilocaine	
M187T335	Amino acid and derivative	Pro-Ala	
M272T415	Amino acid and derivative	Pro-Arg	
M207T326	Amino acid and derivative	Ser-Thr	
M205T286	Amino acid and derivative	Ser-Val	
M248T427	Amino acid and derivative	Thr-Lys	
M386T155	Amino acid and derivative	Tyr-Gln	
M246T274	Amino acid and derivative	Val-Gln	
M231T192	Amino acid and derivative	Val-Ile	
M298T499	Amino acid and derivative	Val-Tyr	
M217T206	Amino acid and derivative	Val-Val	
M219T19	Amino acid and derivative	BHT	
M187T328	Carboxylic Acids	Azelaic acid	C08261
M129T442	Carboxylic Acids	Mesaconic acid	C01732
M162T398	Carboxylic Acids	DL-2-Aminoadipic acid	C00956
M160T397	Carboxylic Acids	DL-2-Aminoadipic acid	C00956
M149T31	Carboxylic Acids	1,2-Benzenedicarboxylic acid	
M171T268	Carboxylic Acids	3-Dehydroshikimic acid	
M114T374	Carboxylic Acids	Maleamic acid	
M282T294	Glycosides	1-Methyladenosine	C02494
M298T319	Glycosides	1-methylguanosine	
M284T260	Glycosides	Guanosine	C00387
M282T129	Glycosides	N6-methyladenosine	
M245T157	Glycosides	Uridine	C00299
M282T260	Glycosides	Guanosine	C00387
M257T140	Glycosides	Ribothymidine	
M303T158	Glycosides	Uridine	C00299
M549T388	Glycosides	Gemcitabine	
M336T68	Glycosides	Isopentenyladenosine	
M298T284	Glycosides	8-hydroxy Guanosine	
M268T167	Glycosides	Adenosine	C00212

M263T342	Lipids	Linoleic acid	C01595
M283T44	Lipids	Stearic acid	C01530
M339T402	Lipids	Behenic acid	C08281
M452T201	Lipids	1-Palmitoyl-2-hydroxy-sn-glycero-3-phosphoethanolamine	
M253T37	Lipids	cis-9-Palmitoleic acid	C08362
M277T45	Lipids	all cis-(6,9,12)-Linolenic acid	C06426
M454T201	Lipids	1-Palmitoyl-2-hydroxy-sn-glycero-3-phosphoethanolamine	
M496T196	Lipids	1-Palmitoyl-sn-glycero-3-phosphocholine	
M330T39	Lipids	Eicosapentaenoic Acid ethyl ester	
M337T244	Lipids	MG(18:2(9Z,12Z)/0:0/0:0)[rac]	
M339T31	Lipids	Norethindrone Acetate	
M152T2602	Nucleic acids and derivative	2-Hydroxyadenine	
M112T237	Nucleic acids and derivative	Cytosine	C00380
M113T158	Nucleic acids and derivative	Uracil	C00106
M113T374	Nucleic acids and derivative	Dihydrouracil	C00429
M111T159	Nucleic acids and derivative	Uracil	C00106
M282T92	Nucleic acids and derivative	2'-O-methyladenosine	C04779
M330T253	Nucleic acids and derivative	Adenosine 2',3'-cyclic monophosphate	C02353
M244T238	Nucleic acids and derivative	Cytidine	C00475
M146T267	Other	Oxyquinoline	
M329T100	Other	Acetylvalerenolic acid	
M391T247	Other	RU-0211	
M128T386	Other	Ammelide	
M233T33	Other	Confertifoline	
M166T256	Phenylpropanoids	L-Phenylalanine	C00079
M164T256	Phenylpropanoids	L-Phenylalanine	C00079
M147T299	Phenylpropanoids	4-Hydroxycinnamic acid	C00811
M565T212	Phenylpropanoids	Apiin	C04858
M563T213	Phenylpropanoids	APIIN	C04858
M220T117	Plant hormone	trans-Zeatin	C00371
M170T258	Plant hormone	Indoleacrylic acid	
M87T374	Plant hormone	4-Hydroxybutanoic acid lactone	
M177T105	Sugar and derivative	L-Gulonic gamma-lactone	C01040
M198T259	Sugar and derivative	D-Mannose	C00159
M667T485	Sugar and derivative	Stachyose	C01613
M193T311	Sugar and derivative	2'-Deoxy-D-ribose	C01801
M147T180	Sugar and derivative	Hydroxyacetone	C05235
M151T228	Sugar and derivative	Xylitol	C00379
M757T39	Sugar and derivative	PC(16:0/16:0)	C00157
M173T334	Sugar and derivative	Glycerol 3-phosphate	C00093

M759T391	Sugar and derivative	Thioetheramide-PC	
M522T189	Sugar and derivative	1-Oleoyl-sn-glycero-3-phosphocholine	
M524T192	Sugar and derivative	1-Stearoyl-2-hydroxy-sn-glycero-3-phosphocholine	
M435T243	Sugar and derivative	1-Oleoyl-L-.alpha.-lysophosphatidic acid	
M409T251	Sugar and derivative	1-Palmitoyl Lysophosphatidic Acid	
M237T356	Sugar and derivative	2-Dehydro-3-deoxy-D-gluconate	C00204
M124T206	Vitamins	Nicotinate	C00253
M122T207	Vitamins	Nicotinate	C00253

Table S11. Selective genes between Wb-NE and Wb-T.

Chromosome	Start position	End position	Strand	Gene ID	KEGG ID
chr2H	14035555	14070479	-	HORVU2Hr1G006720	
chr2H	27503508	27512959	+	HORVU2Hr1G012860	
chr2H	40313666	40315533	-	HORVU2Hr1G017300	
chr2H	41955172	41958298	+	HORVU2Hr1G017400	
chr2H	62692442	62695537	-	HORVU2Hr1G021980	K24824
chr2H	62703527	62704596	-	HORVU2Hr1G022000	K02895
chr2H	64023931	64026621	+	HORVU2Hr1G022160	
chr2H	64030980	64033323	+	HORVU2Hr1G022180	
chr2H	64039579	64039934	+	HORVU2Hr1G022190	
chr2H	65411125	65417655	+	HORVU2Hr1G022490	
chr2H	65416034	65416931	-	HORVU2Hr1G022500	
chr2H	79714638	79718093	+	HORVU2Hr1G025530	K01726
chr2H	79718897	79719047	-	HORVU2Hr1G025550	K00799
chr2H	94192061	94192853	-	HORVU2Hr1G028080	
chr2H	94195785	94195904	-	ENSRNA049482530	
chr2H	94196295	94355239	-	HORVU2Hr1G028120	
chr2H	97111015	97114741	-	HORVU2Hr1G028510	K02997
chr2H	97111868	97119451	+	HORVU2Hr1G028520	
chr2H	108488405	108489309	+	HORVU2Hr1G029880	
chr2H	109878074	109878913	+	HORVU2Hr1G030050	
chr2H	114195967	114196191	-	HORVU2Hr1G030770	
chr2H	115538449	115548113	+	HORVU2Hr1G030930	K11517
chr2H	121673709	121686884	+	HORVU2Hr1G031870	
chr2H	124882875	124884558	+	HORVU2Hr1G032320	K08065
chr2H	126731735	126731808	-	ENSRNA049438045	
chr2H	130780008	130783905	+	HORVU2Hr1G033040	K17795
chr2H	137894271	137903719	+	HORVU2Hr1G034130	K24869
chr2H	140881095	140887745	-	HORVU2Hr1G034440	

chr2H	155161630	155169232	+	HORVU2Hr1G035860	K03937
chr2H	155169270	155170560	-	HORVU2Hr1G035870	
chr2H	162431269	162449010	+	HORVU2Hr1G036510	
chr2H	162999670	163003669	+	HORVU2Hr1G036570	
chr2H	432600008	432600090	-	ENSRNA049483063	
chr2H	487257573	487271837	+	HORVU2Hr1G069110	
chr2H	487261444	487261927	-	HORVU2Hr1G069120	
chr2H	487617705	487633705	+	HORVU2Hr1G069270	
chr2H	488184779	488190175	-	HORVU2Hr1G069390	K20179
chr2H	539497427	539500460	-	HORVU2Hr1G074850	K14525
chr2H	576950309	576959632	+	HORVU2Hr1G079790	
chr2H	579376827	579384098	-	HORVU2Hr1G080050	
chr2H	587891009	587891705	-	HORVU2Hr1G081060	
chr2H	587894299	587898426	-	HORVU2Hr1G081080	
chr2H	610379562	610383259	-	HORVU2Hr1G084170	
chr2H	610385517	610385756	+	HORVU2Hr1G084180	
chr2H	617536376	617538731	+	HORVU2Hr1G085350	K18172
chr2H	617540426	617547291	+	HORVU2Hr1G085360	
chr2H	617555501	617560124	-	HORVU2Hr1G085370	
chr2H	621780030	621783089	+	HORVU2Hr1G086220	
chr2H	621783477	621790943	-	HORVU2Hr1G086230	
chr2H	626231946	626233423	+	HORVU2Hr1G087070	
chr2H	626232169	626232678	-	HORVU2Hr1G087080	
chr2H	626233591	626237975	-	HORVU2Hr1G087090	K00759
chr2H	627474697	627479072	-	HORVU2Hr1G087380	
chr2H	629557364	629558265	+	HORVU2Hr1G087700	
chr2H	629559066	629559712	-	HORVU2Hr1G087710	
chr2H	647300676	647304039	+	HORVU2Hr1G091200	
chr2H	647312810	647317007	+	HORVU2Hr1G091220	K10666
chr2H	647313038	647322216	-	HORVU2Hr1G091230	K10666
chr2H	647828573	647831604	+	HORVU2Hr1G091360	
chr2H	647831678	647838081	-	HORVU2Hr1G091370	K05543
chr2H	648515999	648516959	-	HORVU2Hr1G091580	
chr2H	649203485	649232890	-	HORVU2Hr1G091680	
chr2H	663643530	663645270	+	HORVU2Hr1G094190	
chr2H	671774443	671776153	-	HORVU2Hr1G095840	
chr2H	671998367	672009876	+	HORVU2Hr1G095880	
chr2H	672009432	672011461	-	HORVU2Hr1G095890	
chr2H	677092416	677092610	+	HORVU2Hr1G096900	
chr2H	699752648	699757948	-	HORVU2Hr1G103310	K17609
chr2H	706342872	706346612	-	HORVU2Hr1G105380	
chr2H	706347266	706352048	-	HORVU2Hr1G105390	
chr2H	707265947	707269205	+	HORVU2Hr1G105650	

chr2H	707274729	707277732	+	HORVU2Hr1G105670	
chr2H	708297044	708299453	-	HORVU2Hr1G106020	
chr2H	710833148	710844185	-	HORVU2Hr1G106880	
chr2H	714371933	714375104	+	HORVU2Hr1G108480	
chr2H	714379383	714385493	-	HORVU2Hr1G108490	
chr2H	714380655	714380969	-	HORVU2Hr1G108500	
chr2H	721943807	721946820	-	HORVU2Hr1G111070	
chr2H	723000994	723005790	+	HORVU2Hr1G111530	
chr2H	725464044	725470901	+	HORVU2Hr1G112260	K00327
chr2H	725822715	725826075	-	HORVU2Hr1G112480	
chr2H	731175559	731178336	-	HORVU2Hr1G114190	
chr2H	732688460	732690886	-	HORVU2Hr1G114610	K19730
chr2H	732691119	732698865	+	HORVU2Hr1G114640	K13998
chr2H	735306656	735309864	+	HORVU2Hr1G115710	
chr2H	739142706	739147950	-	HORVU2Hr1G116910	
chr2H	741642130	741643459	+	HORVU2Hr1G118100	
chr2H	742162324	742163148	+	HORVU2Hr1G118290	K16616
chr2H	742197934	742204374	+	HORVU2Hr1G118320	
chr2H	742206222	742207877	-	HORVU2Hr1G118340	
chr2H	750522486	750525536	+	HORVU2Hr1G121020	
chr2H	750530898	750537187	+	HORVU2Hr1G121030	
chr2H	765910275	765917856	+	HORVU2Hr1G127090	
chr2H	765910275	765919163	-	HORVU2Hr1G127100	
chr3H	3242648	3407139	+	HORVU3Hr1G001430	
chr3H	3252999	3253070	+	ENSRNA049437470	
chr3H	24938037	24942947	+	HORVU3Hr1G011540	K23871
chr3H	26934253	26955710	+	HORVU3Hr1G012410	
chr3H	26959609	26961034	+	HORVU3Hr1G012420	K15322
chr3H	26967175	26968718	-	HORVU3Hr1G012430	
chr3H	46958934	46961758	+	HORVU3Hr1G018110	K17508
chr3H	57786136	57791992	+	HORVU3Hr1G019790	K00029
chr3H	57860355	57863598	-	HORVU3Hr1G019840	
chr3H	66838733	66851154	+	HORVU3Hr1G021080	
chr3H	66851391	66851461	-	ENSRNA049434451	
chr3H	68834975	68838931	+	HORVU3Hr1G021310	
chr3H	68842852	68843934	+	HORVU3Hr1G021320	
chr3H	82253055	82262374	-	HORVU3Hr1G022770	K00837
chr3H	86458896	86459282	+	HORVU3Hr1G023380	
chr3H	86464715	86466871	+	HORVU3Hr1G023390	
chr3H	86467302	86469633	+	HORVU3Hr1G023400	K13496
chr3H	86470238	86470507	+	HORVU3Hr1G023410	
chr3H	95271594	95276163	-	HORVU3Hr1G024530	K01115
chr3H	98779822	98788847	-	HORVU3Hr1G024990	

chr3H	103084771	103088991	+	HORVU3Hr1G025920	
chr3H	120578160	120581861	+	HORVU3Hr1G027760	
chr3H	134490014	134493168	-	HORVU3Hr1G029260	
chr3H	134969489	134976262	-	HORVU3Hr1G029340	
chr3H	135042403	135047265	-	HORVU3Hr1G029350	K07198
chr3H	135042439	135043641	+	HORVU3Hr1G029360	
chr3H	135048197	135052830	-	HORVU3Hr1G029370	
chr3H	140890689	140900652	+	HORVU3Hr1G030010	
chr3H	140897671	140901526	-	HORVU3Hr1G030020	
chr3H	175380429	175384468	+	HORVU3Hr1G033430	K18417
chr3H	175384784	175388226	-	HORVU3Hr1G033440	
chr3H	175389167	175397886	-	HORVU3Hr1G033450	K10406
chr3H	177845781	177854317	+	HORVU3Hr1G033700	
chr3H	182123991	182130284	+	HORVU3Hr1G033920	K00031
chr3H	182129348	182135814	-	HORVU3Hr1G033930	
chr3H	182278276	182279474	+	HORVU3Hr1G033950	
chr3H	183306618	183309396	+	HORVU3Hr1G034040	
chr3H	183313528	183314125	-	HORVU3Hr1G034050	
chr3H	186947109	186951309	-	HORVU3Hr1G034440	K00924
chr3H	188607093	188608685	-	HORVU3Hr1G034640	
chr3H	197437297	197446030	+	HORVU3Hr1G035420	
chr3H	198160791	198175649	-	HORVU3Hr1G035590	
chr3H	205145726	205150621	+	HORVU3Hr1G036310	
chr3H	231376289	231384751	+	HORVU3Hr1G039220	K00873
chr3H	231377594	231384772	-	HORVU3Hr1G039230	K00873
chr3H	231388264	231388873	-	HORVU3Hr1G039240	
chr3H	235267245	235286946	-	HORVU3Hr1G039600	K18460
chr3H	238598524	238601525	+	HORVU3Hr1G039930	K04035
chr3H	244048158	244055241	+	HORVU3Hr1G040350	K17301
chr3H	259759283	259761294	+	HORVU3Hr1G041520	
chr3H	261009541	261028057	+	HORVU3Hr1G041810	
chr3H	261022546	261028057	-	HORVU3Hr1G041820	K13436
chr3H	268906906	268955150	-	HORVU3Hr1G042330	
chr3H	270264122	270269601	+	HORVU3Hr1G042540	K11000
chr3H	280495354	280501135	+	HORVU3Hr1G043510	K08592
chr3H	438233271	438235842	+	HORVU3Hr1G058150	
chr3H	470477288	470480401	+	HORVU3Hr1G061790	K10580
chr3H	470539593	470583534	+	HORVU3Hr1G061800	
chr3H	474423476	474428164	-	HORVU3Hr1G062130	K20869
chr3H	476380758	476388549	-	HORVU3Hr1G062370	
chr3H	476389377	476389965	-	HORVU3Hr1G062390	
chr3H	479976250	479983057	-	HORVU3Hr1G062740	
chr3H	479983413	480017371	-	HORVU3Hr1G062760	

chr3H	511310492	511321405	+	HORVU3Hr1G067300	
chr3H	518532518	518534806	-	HORVU3Hr1G068400	
chr3H	520311285	520315332	-	HORVU3Hr1G068700	
chr3H	530321347	530325450	-	HORVU3Hr1G070040	
chr3H	530324742	530329449	+	HORVU3Hr1G070050	K05954
chr3H	538802945	538807675	-	HORVU3Hr1G071240	
chr3H	541043012	541050693	+	HORVU3Hr1G071550	K09419
chr3H	541485968	541492933	+	HORVU3Hr1G071570	K16055
chr3H	552738456	552750250	+	HORVU3Hr1G073220	
chr3H	555470279	555480914	-	HORVU3Hr1G073790	
chr3H	555555698	555559733	-	HORVU3Hr1G073800	
chr3H	559879827	559884486	+	HORVU3Hr1G074800	K03644
chr3H	574886154	574890042	+	HORVU3Hr1G077780	
chr3H	576162439	576169103	-	HORVU3Hr1G078110	K10695
chr3H	582534472	582542774	+	HORVU3Hr1G079270	K00109
chr3H	597391061	597393409	-	HORVU3Hr1G082030	
chr3H	598334946	598337882	+	HORVU3Hr1G082230	K22733
chr3H	598337922	598346861	-	HORVU3Hr1G082240	
chr3H	611776668	611781499	+	HORVU3Hr1G085280	K00967
chr3H	616206893	616210388	+	HORVU3Hr1G086200	K05909
chr3H	616215859	616219980	-	HORVU3Hr1G086210	
chr3H	619313438	619316091	-	HORVU3Hr1G086930	K02957
chr3H	620456651	620458731	+	HORVU3Hr1G087180	
chr3H	654228674	654232763	+	HORVU3Hr1G096710	
chr3H	654496084	654499747	+	HORVU3Hr1G096780	
chr3H	654881439	654883221	-	HORVU3Hr1G097000	
chr3H	654885248	654889252	-	HORVU3Hr1G097010	K01188
chr3H	654891181	654892689	+	HORVU3Hr1G097030	
chr3H	660840084	660849791	+	HORVU3Hr1G099120	K13430
chr3H	660840235	660849784	-	HORVU3Hr1G099130	K13430
chr3H	670194785	670197235	-	HORVU3Hr1G105870	
chr3H	673750478	673751897	+	HORVU3Hr1G107910	
chr3H	673755306	673761742	+	HORVU3Hr1G107920	
chr3H	688839243	688844859	+	HORVU3Hr1G113640	
chr3H	688850191	688852300	+	HORVU3Hr1G113650	
chr3H	688853248	688854537	-	HORVU3Hr1G113660	
chr3H	688952163	688952246	-	ENSRNA049436778	
chr3H	688954217	688954300	-	ENSRNA049436790	
chr3H	688959593	688959676	-	ENSRNA049436800	
chr3H	688959786	688967856	-	HORVU3Hr1G113720	
chr3H	688963885	688964552	+	HORVU3Hr1G113730	
chr3H	690416248	690421349	-	HORVU3Hr1G114240	
chr3H	690418017	690418964	+	HORVU3Hr1G114250	

chr3H	690680636	690689148	+	HORVU3Hr1G114310	K04498
chr5H	20455220	20455874	+	HORVU5Hr1G009020	
chr5H	38555667	38562124	+	HORVU5Hr1G012770	K00547
chr5H	38557723	38562136	-	HORVU5Hr1G012780	K00547
chr5H	71837213	71844074	-	HORVU5Hr1G018040	
chr5H	160187107	160247758	-	HORVU5Hr1G027830	
chr5H	193052889	193074651	+	HORVU5Hr1G030970	
chr5H	193054074	193054505	-	HORVU5Hr1G030980	
chr5H	193062941	193063135	-	HORVU5Hr1G031010	
chr5H	220744391	220744960	-	HORVU5Hr1G033240	
chr5H	231082080	231113023	-	HORVU5Hr1G034400	
chr5H	231086676	231087259	+	HORVU5Hr1G034420	
chr5H	231087953	231088333	+	HORVU5Hr1G034430	
chr5H	231088786	231089365	+	HORVU5Hr1G034440	
chr5H	255509706	255512098	-	HORVU5Hr1G036250	K02155
chr5H	256600980	256606500	+	HORVU5Hr1G036360	
chr5H	257988740	257995792	-	HORVU5Hr1G036460	K01897
chr5H	270203770	270207437	-	HORVU5Hr1G037600	K03686
chr5H	280775389	280777976	+	HORVU5Hr1G038920	K02997
chr5H	286690669	286711619	+	HORVU5Hr1G039430	
chr5H	288433498	288439832	+	HORVU5Hr1G039620	
chr5H	290716942	290720528	+	HORVU5Hr1G039850	K10355
chr5H	293329487	293336347	+	HORVU5Hr1G040040	
chr5H	293476239	293476827	+	HORVU5Hr1G040080	
chr5H	308435568	308435744	-	HORVU5Hr1G041040	
chr5H	359517904	359532721	-	HORVU5Hr1G046250	
chr5H	359527202	359529307	+	HORVU5Hr1G046260	
chr5H	359535548	359536767	-	HORVU5Hr1G046280	
chr5H	363428454	363429949	-	HORVU5Hr1G046850	
chr5H	363433374	363438316	+	HORVU5Hr1G046860	
chr5H	373446467	373455729	+	HORVU5Hr1G048070	K01953
chr5H	373450820	373456880	-	HORVU5Hr1G048100	
chr5H	376919025	377036656	+	HORVU5Hr1G048660	
chr5H	440157127	440167130	-	HORVU5Hr1G056280	
chr5H	441631409	441633105	+	HORVU5Hr1G056380	
chr5H	441635331	441639155	+	HORVU5Hr1G056390	K01792
chr5H	441639539	441643067	-	HORVU5Hr1G056400	
chr5H	480973094	480984107	+	HORVU5Hr1G061530	
chr5H	519242296	519242487	-	HORVU5Hr1G068670	
chr5H	519246409	519246624	+	HORVU5Hr1G068680	
chr5H	519247291	519247497	+	HORVU5Hr1G068700	
chr5H	520429673	520434263	+	HORVU5Hr1G069050	
chr5H	533642537	533643952	+	HORVU5Hr1G071720	

chr5H	540583851	540586087	+	HORVU5Hr1G073680	K12836
chr5H	540586729	540590466	+	HORVU5Hr1G073690	
chr5H	540599752	540600513	+	HORVU5Hr1G073700	
chr5H	541383545	541385735	-	HORVU5Hr1G073950	
chr5H	542981416	542984571	+	HORVU5Hr1G074340	
chr5H	542981815	542991490	-	HORVU5Hr1G074350	
chr5H	546258332	546260686	-	HORVU5Hr1G075200	
chr5H	547114171	547116264	+	HORVU5Hr1G075420	K02912
chr5H	549517615	549520630	-	HORVU5Hr1G076120	K13947
chr5H	549527020	549530983	+	HORVU5Hr1G076130	
chr5H	552951563	552952213	-	HORVU5Hr1G077170	
chr5H	552963033	552970060	-	HORVU5Hr1G077190	
chr5H	552970957	552978011	-	HORVU5Hr1G077200	K00162
chr5H	566073813	566077051	-	HORVU5Hr1G082010	
chr5H	566073828	566074100	+	HORVU5Hr1G082020	
chr5H	568196281	568199026	-	HORVU5Hr1G082680	
chr5H	568255238	568258790	-	HORVU5Hr1G082700	
chr5H	569819567	569822936	-	HORVU5Hr1G083310	
chr5H	574614503	574619304	+	HORVU5Hr1G085320	K10875
chr5H	574625123	574631196	-	HORVU5Hr1G085330	
chr5H	587901515	587907220	-	HORVU5Hr1G092250	
chr5H	589575141	589583643	+	HORVU5Hr1G092840	K03010
chr5H	590853571	590854869	+	HORVU5Hr1G093190	
chr5H	590856159	590860165	-	HORVU5Hr1G093210	K20818
chr5H	592408069	592409479	+	HORVU5Hr1G093640	K14484
chr5H	592411406	592420140	-	HORVU5Hr1G093650	K03116
chr5H	592424716	592426560	-	HORVU5Hr1G093660	
chr5H	592515276	592521098	-	HORVU5Hr1G093720	K16277
chr5H	592524226	592525113	+	HORVU5Hr1G093730	
chr5H	595570698	595574227	-	HORVU5Hr1G094430	K01534
chr5H	612296412	612298786	-	HORVU5Hr1G101010	
chr5H	612299923	612302632	-	HORVU5Hr1G101020	K01807
chr5H	620734634	620739948	+	HORVU5Hr1G105040	K15407
chr5H	623058746	623061089	-	HORVU5Hr1G105970	K14553
chr5H	623061300	623067793	+	HORVU5Hr1G105980	
chr5H	626650157	626657447	+	HORVU5Hr1G107360	
chr5H	629282149	629287350	+	HORVU5Hr1G109160	
chr5H	629289966	629290184	-	ENSRNA049478581	
chr5H	629291325	629291544	-	ENSRNA049478578	
chr5H	629295132	629296380	+	HORVU5Hr1G109190	
chr5H	630038180	630039815	+	HORVU5Hr1G109450	K11251
chr5H	630039071	630039937	-	HORVU5Hr1G109460	K11251
chr5H	630049593	630057341	+	HORVU5Hr1G109470	

chr5H	630712092	630713548	-	HORVU5Hr1G109710	K02692
chr5H	630778643	630784838	-	HORVU5Hr1G109720	K09613
chr5H	630787393	630789598	+	HORVU5Hr1G109730	
chr5H	631251083	631252934	-	HORVU5Hr1G109910	
chr5H	631585522	631592253	-	HORVU5Hr1G110000	K10999
chr5H	632089832	632091949	-	HORVU5Hr1G110170	
chr5H	632292884	632293044	+	ENSRNA049480406	
chr5H	632292896	632293152	+	HORVU5Hr1G110270	
chr5H	632293956	632302971	-	HORVU5Hr1G110280	K17508
chr5H	632383586	632386920	+	HORVU5Hr1G110330	K02737
chr5H	632390004	632399276	-	HORVU5Hr1G110340	K03357
chr5H	632392604	632393119	-	HORVU5Hr1G110350	K03357
chr5H	636383933	636384036	+	ENSRNA049480398	
chr5H	636675899	636680290	+	HORVU5Hr1G111540	K14945
chr5H	636814022	636816516	+	HORVU5Hr1G111640	K02879
chr5H	636816684	636816767	+	ENSRNA049443512	
chr5H	638459130	638478382	-	HORVU5Hr1G112210	
chr5H	644817652	644820205	+	HORVU5Hr1G114840	
chr5H	645045171	645052413	+	HORVU5Hr1G114910	
chr5H	645429217	645433078	+	HORVU5Hr1G115010	
chr5H	645433637	645438458	+	HORVU5Hr1G115030	
chr5H	646848440	646853969	+	HORVU5Hr1G115530	
chr5H	651473577	651473823	+	HORVU5Hr1G117890	
chr5H	651481517	651485571	+	HORVU5Hr1G117900	K10355
chr5H	652964039	652967879	+	HORVU5Hr1G118460	
chr5H	652968576	652968647	+	ENSRNA049443432	
chr5H	652969084	652974847	-	HORVU5Hr1G118480	
chr5H	654684138	654689663	-	HORVU5Hr1G119060	
chr5H	655883016	655884783	+	HORVU5Hr1G119500	
chr5H	655885659	655888626	-	HORVU5Hr1G119510	
chr5H	655899900	656067721	-	HORVU5Hr1G119520	
chr5H	656651812	656654121	-	HORVU5Hr1G119810	
chr5H	657712172	657727609	-	HORVU5Hr1G120480	
chr5H	659415973	659422455	+	HORVU5Hr1G121310	
chr5H	663388588	663390379	-	HORVU5Hr1G123120	
chr5H	663395230	663397277	-	HORVU5Hr1G123150	
chr5H	663397690	663402446	-	HORVU5Hr1G123160	K13989
chr5H	668242695	668244938	+	HORVU5Hr1G125190	
chr5H	668250315	668252666	+	HORVU5Hr1G125200	K06269
chr5H	668262529	668266358	-	HORVU5Hr1G125220	
chr7H	3419740	3423227	+	HORVU7Hr1G001560	K22139
chr7H	3430922	3433264	-	HORVU7Hr1G001570	
chr7H	4055831	4058258	+	HORVU7Hr1G002060	K02940

chr7H	4278136	4283136	+	HORVU7Hr1G002200	K02641
chr7H	4279678	4283236	-	HORVU7Hr1G002210	K02641
chr7H	4657357	4658776	-	HORVU7Hr1G002440	
chr7H	4659281	4663257	-	HORVU7Hr1G002450	
chr7H	24723050	24724705	+	HORVU7Hr1G018530	K00430
chr7H	26195337	26195407	+	ENSRNA049442277	
chr7H	26196461	26208756	-	HORVU7Hr1G019550	
chr7H	27958533	28145362	+	HORVU7Hr1G020660	
chr7H	30203510	30205049	-	HORVU7Hr1G021320	
chr7H	30207753	30213665	+	HORVU7Hr1G021340	
chr7H	31744977	31749786	+	HORVU7Hr1G021980	
chr7H	32641735	32644162	-	HORVU7Hr1G022310	
chr7H	32659079	32660072	-	HORVU7Hr1G022330	
chr7H	34396250	34402461	+	HORVU7Hr1G022910	
chr7H	34424780	34430943	-	HORVU7Hr1G022940	
chr7H	34432709	34435123	-	HORVU7Hr1G022970	K02737
chr7H	34436857	34440982	-	HORVU7Hr1G022980	
chr7H	34771324	34773069	-	HORVU7Hr1G023060	
chr7H	34773076	34777739	+	HORVU7Hr1G023070	
chr7H	34796990	34945086	-	HORVU7Hr1G023100	
chr7H	34820156	34822028	-	HORVU7Hr1G023140	
chr7H	34826121	34839822	+	HORVU7Hr1G023150	
chr7H	38049222	38051414	+	HORVU7Hr1G024100	
chr7H	40734383	40737494	-	HORVU7Hr1G024890	K14566
chr7H	41832522	41834863	-	HORVU7Hr1G025380	
chr7H	41834939	41841912	-	HORVU7Hr1G025390	
chr7H	41843877	41845648	-	HORVU7Hr1G025400	
chr7H	42673891	42673962	+	ENSRNA049442250	
chr7H	42674384	42674455	+	ENSRNA049442241	
chr7H	42684121	42690645	-	HORVU7Hr1G025620	
chr7H	42957041	42958811	-	HORVU7Hr1G025720	K09422
chr7H	42960311	42964316	+	HORVU7Hr1G025730	K24418
chr7H	44222056	44227665	+	HORVU7Hr1G025990	
chr7H	44237192	44240267	+	HORVU7Hr1G026000	
chr7H	44239049	44242739	-	HORVU7Hr1G026020	
chr7H	45701766	45710711	-	HORVU7Hr1G026430	
chr7H	47595794	47598767	-	HORVU7Hr1G027010	
chr7H	50176082	50176275	+	ENSRNA049488590	
chr7H	50177527	50178703	+	HORVU7Hr1G028070	
chr7H	50182341	50182536	+	ENSRNA049488586	
chr7H	50183680	50184306	-	HORVU7Hr1G028080	
chr7H	50185054	50185249	+	ENSRNA049488589	
chr7H	51814370	51817043	-	HORVU7Hr1G028370	

chr7H	58171206	58178764	+	HORVU7Hr1G030040	
chr7H	58178807	58183475	-	HORVU7Hr1G030050	
chr7H	62540594	62540770	+	HORVU7Hr1G030990	
chr7H	62540774	62541217	+	HORVU7Hr1G031000	
chr7H	62541536	62541691	+	HORVU7Hr1G031010	
chr7H	62553494	62559649	+	HORVU7Hr1G031020	
chr7H	62744251	62752110	+	HORVU7Hr1G031100	K14311
chr7H	69927208	69928231	+	HORVU7Hr1G033780	K03841
chr7H	69932081	69940038	-	HORVU7Hr1G033820	
chr7H	85850542	85854380	+	HORVU7Hr1G036660	
chr7H	89778600	89780563	+	HORVU7Hr1G037410	
chr7H	90402643	90406306	-	HORVU7Hr1G037470	
chr7H	90403030	90405049	+	HORVU7Hr1G037480	
chr7H	90406533	90414581	-	HORVU7Hr1G037510	
chr7H	90574611	90578805	-	HORVU7Hr1G037550	K15032
chr7H	109373213	109374520	-	HORVU7Hr1G040370	K08912
chr7H	109380119	109381636	-	HORVU7Hr1G040380	
chr7H	109382547	109389554	-	HORVU7Hr1G040390	
chr7H	144284135	144291911	+	HORVU7Hr1G045550	
chr7H	144289836	144293353	-	HORVU7Hr1G045580	
chr7H	146582057	146582296	-	HORVU7Hr1G045750	
chr7H	146585894	146590335	+	HORVU7Hr1G045770	K16732
chr7H	149875814	149879925	+	HORVU7Hr1G046250	K01188
chr7H	149880190	149883832	-	HORVU7Hr1G046270	
chr7H	149883969	149885304	+	HORVU7Hr1G046280	
chr7H	150238886	150248647	+	HORVU7Hr1G046290	
chr7H	154200185	154200457	-	HORVU7Hr1G046790	
chr7H	154201749	154203538	-	HORVU7Hr1G046800	
chr7H	154203905	154205840	-	HORVU7Hr1G046820	K21483
chr7H	154206334	154207469	-	HORVU7Hr1G046830	
chr7H	156144132	156151763	+	HORVU7Hr1G047020	K18749
chr7H	159155959	159169545	+	HORVU7Hr1G047420	
chr7H	159165280	159165582	-	HORVU7Hr1G047430	
chr7H	159465068	159468935	-	HORVU7Hr1G047500	
chr7H	161416110	161417025	-	HORVU7Hr1G047780	
chr7H	161419405	161421513	-	HORVU7Hr1G047790	
chr7H	162957742	162969327	-	HORVU7Hr1G047980	
chr7H	162972005	162982693	-	HORVU7Hr1G047990	
chr7H	162983078	162983380	+	HORVU7Hr1G048000	K03878
chr7H	163191173	163195598	+	HORVU7Hr1G048040	
chr7H	164006021	164011620	-	HORVU7Hr1G048120	
chr7H	164864300	164884445	+	HORVU7Hr1G048330	
chr7H	171722329	171736691	-	HORVU7Hr1G049220	

chr7H	171726034	171726321	-	HORVU7Hr1G049230	
chr7H	172195118	172199921	-	HORVU7Hr1G049240	K03327
chr7H	173367734	173374639	+	HORVU7Hr1G049400	K14724
chr7H	173376233	173376772	-	HORVU7Hr1G049420	
chr7H	180596856	180615545	+	HORVU7Hr1G050300	
chr7H	180865508	180866625	+	HORVU7Hr1G050340	
chr7H	182986551	182987664	-	HORVU7Hr1G050540	
chr7H	185963320	185966560	-	HORVU7Hr1G050740	
chr7H	187439604	187448519	-	HORVU7Hr1G050880	
chr7H	188001870	188003539	+	HORVU7Hr1G050950	K22378
chr7H	189799612	189803487	+	HORVU7Hr1G051070	K14677
chr7H	190708379	190711713	+	HORVU7Hr1G051140	K05956
chr7H	190712096	190716193	-	HORVU7Hr1G051150	
chr7H	192879289	192885430	-	HORVU7Hr1G051400	
chr7H	193865646	193872026	+	HORVU7Hr1G051550	
chr7H	193865675	193872300	-	HORVU7Hr1G051560	
chr7H	194166695	194170342	-	HORVU7Hr1G051570	K00434
chr7H	194170650	194172694	-	HORVU7Hr1G051580	
chr7H	195559671	195560519	-	HORVU7Hr1G051740	K09422
chr7H	195560821	195569574	-	HORVU7Hr1G051750	
chr7H	195561676	195564596	+	HORVU7Hr1G051760	K01214
chr7H	195566397	195567259	-	HORVU7Hr1G051770	
chr7H	196771438	196779677	-	HORVU7Hr1G051930	
chr7H	197645209	197649451	+	HORVU7Hr1G052060	
chr7H	197645407	197650059	-	HORVU7Hr1G052070	
chr7H	197650488	197655955	-	HORVU7Hr1G052090	
chr7H	198245435	198252919	-	HORVU7Hr1G052190	
chr7H	200511571	200514820	-	HORVU7Hr1G052520	
chr7H	200516181	200519231	-	HORVU7Hr1G052530	K22857
chr7H	201214148	201216151	+	HORVU7Hr1G052580	
chr7H	202232707	202236187	+	HORVU7Hr1G052720	K12272
chr7H	202238220	202239207	-	HORVU7Hr1G052730	
chr7H	203224790	203227407	-	HORVU7Hr1G052800	
chr7H	206624036	206628806	-	HORVU7Hr1G053170	K09571
chr7H	206628334	206628591	+	HORVU7Hr1G053180	
chr7H	207066666	207071314	+	HORVU7Hr1G053230	
chr7H	208489726	208501010	-	HORVU7Hr1G053310	
chr7H	208495957	208496570	-	HORVU7Hr1G053320	
chr7H	208499001	208499183	-	HORVU7Hr1G053350	
chr7H	208499718	208499948	-	HORVU7Hr1G053360	
chr7H	209107148	209116437	-	HORVU7Hr1G053440	
chr7H	213013811	213019782	+	HORVU7Hr1G053940	
chr7H	217284728	217287615	+	HORVU7Hr1G054440	

chr7H	217288945	217289820	-	HORVU7Hr1G054460	
chr7H	221347389	221350163	-	HORVU7Hr1G054670	K02936
chr7H	221602866	221606903	-	HORVU7Hr1G054690	
chr7H	234654877	234669226	-	HORVU7Hr1G056410	
chr7H	234665154	234712484	+	HORVU7Hr1G056420	
chr7H	235074749	235077901	+	HORVU7Hr1G056450	
chr7H	235075668	235080845	-	HORVU7Hr1G056460	K15191
chr7H	235388068	235395343	-	HORVU7Hr1G056470	
chr7H	236127458	236134439	+	HORVU7Hr1G056490	
chr7H	237161371	237174117	+	HORVU7Hr1G056570	
chr7H	237647324	237651565	+	HORVU7Hr1G056590	K01641
chr7H	237946753	237949777	-	HORVU7Hr1G056600	
chr7H	238440698	238446807	+	HORVU7Hr1G056630	
chr7H	238655386	238656052	+	HORVU7Hr1G056650	
chr7H	238656122	238662065	-	HORVU7Hr1G056660	
chr7H	240513113	240531374	+	HORVU7Hr1G056770	
chr7H	240520656	240521269	+	HORVU7Hr1G056780	
chr7H	240628874	240631292	+	HORVU7Hr1G056790	
chr7H	243535369	243549081	+	HORVU7Hr1G057010	
chr7H	248143264	248146274	+	HORVU7Hr1G057310	K19045
chr7H	248580108	248581914	+	HORVU7Hr1G057330	
chr7H	248978165	248980455	-	HORVU7Hr1G057350	
chr7H	249987945	249993911	+	HORVU7Hr1G057390	
chr7H	251131641	251134363	-	HORVU7Hr1G057470	
chr7H	254995599	254996632	+	HORVU7Hr1G058040	
chr7H	259173617	259189496	-	HORVU7Hr1G058410	
chr7H	261448634	261452472	-	HORVU7Hr1G058850	
chr7H	285759629	285782957	+	HORVU7Hr1G061220	
chr7H	310310675	310312040	-	HORVU7Hr1G063280	K02901
chr7H	310315042	310316509	-	HORVU7Hr1G063290	K10251
chr7H	312354778	312373967	-	HORVU7Hr1G063520	
chr7H	314409375	314409915	+	HORVU7Hr1G063770	
chr7H	324264552	324268115	+	HORVU7Hr1G065280	K14207
chr7H	343592862	343593277	+	HORVU7Hr1G066740	
chr7H	343596981	343600920	-	HORVU7Hr1G066750	
chr7H	350974016	350976357	+	HORVU7Hr1G067280	K02985
chr7H	399070399	399080044	-	HORVU7Hr1G071800	K03141
chr7H	400513342	400517923	+	HORVU7Hr1G071940	
chr7H	400518832	400523214	-	HORVU7Hr1G071960	
chr7H	400829251	400835819	-	HORVU7Hr1G072050	
chr7H	401049202	401096899	-	HORVU7Hr1G072120	
chr7H	404317811	404321138	+	HORVU7Hr1G072420	
chr7H	405458507	405463986	-	HORVU7Hr1G072480	

chr7H	408933656	408966137	+	HORVU7Hr1G072790	
chr7H	408945029	408945184	-	HORVU7Hr1G072800	
chr7H	408945305	408945904	-	HORVU7Hr1G072810	
chr7H	408945917	408946516	-	HORVU7Hr1G072820	
chr7H	413570039	413582405	+	HORVU7Hr1G073220	
chr7H	427045026	427046170	-	HORVU7Hr1G074600	
chr7H	427238722	427242968	-	HORVU7Hr1G074640	
chr7H	445815006	445817001	+	HORVU7Hr1G076300	
chr7H	445817491	445819772	-	HORVU7Hr1G076310	
chr7H	451171861	451177182	+	HORVU7Hr1G077110	
chr7H	460866409	460868613	-	HORVU7Hr1G078500	K08515
chr7H	462745728	462752895	+	HORVU7Hr1G078750	K15377
chr7H	463238773	463242433	-	HORVU7Hr1G078770	
chr7H	463492869	463507474	+	HORVU7Hr1G078800	
chr7H	463508051	463514690	+	HORVU7Hr1G078850	
chr7H	464640783	464646432	+	HORVU7Hr1G079000	K13095
chr7H	464653655	464653727	+	ENSRNA049441543	
chr7H	465025226	465036375	-	HORVU7Hr1G079030	
chr7H	465887867	465889464	+	HORVU7Hr1G079190	K19033
chr7H	469365393	469367735	+	HORVU7Hr1G079500	
chr7H	469375538	469380413	-	HORVU7Hr1G079510	
chr7H	473215437	473216410	-	HORVU7Hr1G080050	
chr7H	473559055	473559912	-	HORVU7Hr1G080080	K19038
chr7H	500367633	500378590	-	HORVU7Hr1G083070	K14799
chr7H	502090949	502092564	+	HORVU7Hr1G083360	
chr7H	502233708	502695154	-	HORVU7Hr1G083370	
chr7H	502928126	502931563	-	HORVU7Hr1G083410	
chr7H	503360357	503366602	-	HORVU7Hr1G083480	K18418
chr7H	503369928	503381186	-	HORVU7Hr1G083490	
chr7H	517019396	517024657	+	HORVU7Hr1G085500	K14829
chr7H	522972754	522975238	-	HORVU7Hr1G086720	
chr7H	534305062	534315817	+	HORVU7Hr1G088250	
chr7H	537244411	537251423	+	HORVU7Hr1G088450	
chr7H	540257078	540264097	+	HORVU7Hr1G088960	K20523
chr7H	540271901	540278990	-	HORVU7Hr1G088980	
chr7H	540677331	540679335	+	HORVU7Hr1G089110	
chr7H	540689692	540693022	+	HORVU7Hr1G089120	
chr7H	542577531	542579345	-	HORVU7Hr1G089370	K00430
chr7H	542583419	542586014	-	HORVU7Hr1G089380	
chr7H	543246447	543248174	+	HORVU7Hr1G089480	
chr7H	543727930	543729952	-	HORVU7Hr1G089510	
chr7H	545412647	545417673	-	HORVU7Hr1G089660	
chr7H	546156753	546158544	+	HORVU7Hr1G089870	

chr7H	546426392	546430875	+	HORVU7Hr1G089910	
chr7H	546913078	546917047	-	HORVU7Hr1G089960	
chr7H	547205417	547213806	-	HORVU7Hr1G090020	
chr7H	547315437	547316288	+	HORVU7Hr1G090040	
chr7H	547317678	547318796	-	HORVU7Hr1G090050	
chr7H	548078348	548082099	-	HORVU7Hr1G090140	
chr7H	548933250	548935805	-	HORVU7Hr1G090240	
chr7H	550404963	550413291	-	HORVU7Hr1G090330	
chr7H	550404963	550413302	+	HORVU7Hr1G090340	
chr7H	550734743	550737520	-	HORVU7Hr1G090350	
chr7H	550740587	550742318	-	HORVU7Hr1G090360	
chr7H	550853424	550858384	+	HORVU7Hr1G090380	K03064
chr7H	550858891	550866295	+	HORVU7Hr1G090390	
chr7H	552670602	552694892	+	HORVU7Hr1G090650	
chr7H	552684535	552685357	+	HORVU7Hr1G090670	K15627
chr7H	553103732	553106127	-	HORVU7Hr1G090790	K12834
chr7H	554017461	554023977	-	HORVU7Hr1G090900	
chr7H	554198073	554200387	-	HORVU7Hr1G090940	K15151
chr7H	555785737	555788694	-	HORVU7Hr1G091100	
chr7H	557913758	557915024	+	HORVU7Hr1G091350	K14488
chr7H	557948460	557957088	-	HORVU7Hr1G091360	
chr7H	559400701	559403941	-	HORVU7Hr1G091440	
chr7H	559896533	559897789	+	HORVU7Hr1G091560	
chr7H	559899709	559906098	+	HORVU7Hr1G091570	
chr7H	559905250	559906090	-	HORVU7Hr1G091580	
chr7H	561659828	561668258	+	HORVU7Hr1G091810	
chr7H	561672364	561673570	+	HORVU7Hr1G091830	
chr7H	563336962	563339376	+	HORVU7Hr1G092030	
chr7H	564731459	564735265	+	HORVU7Hr1G092230	K13356
chr7H	564737238	564740225	-	HORVU7Hr1G092240	K13456
chr7H	566875125	566878840	+	HORVU7Hr1G092550	
chr7H	566885978	566889696	-	HORVU7Hr1G092560	
chr7H	567022987	567025116	+	HORVU7Hr1G092570	
chr7H	568286293	568289672	+	HORVU7Hr1G092810	
chr7H	570046163	570059714	+	HORVU7Hr1G093200	
chr7H	577781998	577789646	+	HORVU7Hr1G094850	
chr7H	577786087	577793427	-	HORVU7Hr1G094860	
chr7H	582766299	582768290	+	HORVU7Hr1G095550	K15227
chr7H	586453584	586456625	-	HORVU7Hr1G096170	K14864
chr7H	587050932	587064975	+	HORVU7Hr1G096240	
chr7H	587065464	587069800	+	HORVU7Hr1G096250	
chr7H	587334940	587339776	+	HORVU7Hr1G096300	K01698
chr7H	587340841	587343131	-	HORVU7Hr1G096310	

chr7H	587768812	587773329	+	HORVU7Hr1G096360	
chr7H	587770547	587773339	-	HORVU7Hr1G096370	
chr7H	588873152	588876551	+	HORVU7Hr1G096670	
chr7H	591550703	591556171	-	HORVU7Hr1G097250	
chr7H	592415795	592422825	+	HORVU7Hr1G097470	
chr7H	593797574	593805946	+	HORVU7Hr1G097860	
chr7H	593806745	593807955	+	HORVU7Hr1G097870	K22312
chr7H	593809872	593809944	+	ENSRNA049441092	
chr7H	606342950	606344505	+	HORVU7Hr1G100790	
chr7H	606347563	606350332	-	HORVU7Hr1G100800	
chr7H	609402126	609407087	+	HORVU7Hr1G101500	K17871
chr7H	617090172	617090751	-	HORVU7Hr1G105470	K01047
chr7H	617091976	617101370	-	HORVU7Hr1G105490	
chr7H	617102780	617103913	-	HORVU7Hr1G105500	K08065
chr7H	617110917	617114740	-	HORVU7Hr1G105510	
chr7H	617115496	617117707	+	HORVU7Hr1G105520	K09422
chr7H	620784469	620789669	+	HORVU7Hr1G106780	
chr7H	620792136	620794036	-	HORVU7Hr1G106790	
chr7H	621825575	621833118	+	HORVU7Hr1G107050	K17680
chr7H	621832543	621832731	-	HORVU7Hr1G107060	
chr7H	621834268	621836472	+	HORVU7Hr1G107070	
chr7H	621836594	621839030	+	HORVU7Hr1G107080	K12668
chr7H	621839414	621842700	-	HORVU7Hr1G107100	K07375
chr7H	623413985	623419666	+	HORVU7Hr1G107550	
chr7H	623697682	623701888	+	HORVU7Hr1G107740	
chr7H	627313778	627314831	-	HORVU7Hr1G109200	
chr7H	627315504	627317238	-	HORVU7Hr1G109210	
chr7H	628247557	628248157	-	HORVU7Hr1G109540	
chr7H	628254382	628257576	+	HORVU7Hr1G109550	
chr7H	628360508	628360580	+	ENSRNA049440972	
chr7H	628363979	628364051	+	ENSRNA049440961	
chr7H	628364391	628364463	-	ENSRNA049441783	
chr7H	628364665	628369128	-	HORVU7Hr1G109580	
chr7H	628369567	628374492	-	HORVU7Hr1G109590	
chr7H	628952998	628958777	+	HORVU7Hr1G109780	K01669
chr7H	628954869	628958773	-	HORVU7Hr1G109800	K01669
chr7H	629272958	629278828	+	HORVU7Hr1G109990	
chr7H	629279184	629279406	+	HORVU7Hr1G110010	
chr7H	629279194	629279413	-	ENSRNA049488657	
chr7H	629282113	629282332	-	ENSRNA049488655	
chr7H	629950371	629954881	-	HORVU7Hr1G110440	
chr7H	637258554	637259907	-	HORVU7Hr1G113530	
chr7H	638730005	638730454	-	HORVU7Hr1G114090	

chr7H	638911322	638913789	+	HORVU7Hr1G114170	
chr7H	640595999	640598651	-	HORVU7Hr1G114950	K01673
chr7H	640603497	640606918	-	HORVU7Hr1G114960	
chr7H	640618568	640618780	-	HORVU7Hr1G114980	
chr7H	640840159	640840713	-	HORVU7Hr1G115130	
chr7H	640845847	640846103	+	HORVU7Hr1G115150	
chr7H	646985126	646986042	-	HORVU7Hr1G118220	
chr7H	646996504	647004378	-	HORVU7Hr1G118230	
chr7H	647183877	647190478	+	HORVU7Hr1G118300	
chr7H	648306135	648307667	+	HORVU7Hr1G118760	
chr7H	648927631	648929836	+	HORVU7Hr1G119040	
chr7H	648932670	648934784	+	HORVU7Hr1G119050	
chr7H	649461248	649462826	+	HORVU7Hr1G119360	
chr7H	649466990	649472294	+	HORVU7Hr1G119370	
chr7H	649994417	649998004	+	HORVU7Hr1G119590	
chr7H	649999627	650002431	+	HORVU7Hr1G119610	
chr7H	650390677	650393515	-	HORVU7Hr1G119810	K12871
chr7H	650398234	650404481	+	HORVU7Hr1G119820	K01354
chr7H	650553586	650555302	+	HORVU7Hr1G119870	
chr7H	650875980	650880895	+	HORVU7Hr1G120070	K00791
chr7H	650882126	650883358	-	HORVU7Hr1G120100	
chr7H	650884266	650887906	+	HORVU7Hr1G120120	
chr7H	650888232	650891426	-	HORVU7Hr1G120130	
chr7H	651001497	651006560	-	HORVU7Hr1G120170	
chr7H	651006689	651006874	-	HORVU7Hr1G120180	
chr7H	651008226	651010988	-	HORVU7Hr1G120190	
chr7H	651066589	651066788	+	HORVU7Hr1G120220	
chr7H	651190889	651192744	+	HORVU7Hr1G120340	K18801
chr7H	651202413	651203090	-	HORVU7Hr1G120350	K00224
chr7H	651204386	651204457	-	ENSRNA049441948	
chr7H	651727091	651731987	-	HORVU7Hr1G120520	K00695
chr7H	651732286	651737413	-	HORVU7Hr1G120540	
chr4H	1139723	1146439	+	HORVU4Hr1G000660	
chr4H	1139723	1146539	-	HORVU4Hr1G000670	
chr4H	4190962	4197198	+	HORVU4Hr1G002270	K00549
chr4H	4194802	4197705	-	HORVU4Hr1G002280	K00549
chr4H	23461411	23468904	+	HORVU4Hr1G008530	
chr4H	24979535	24985488	-	HORVU4Hr1G008820	K11649
chr4H	41102174	41113394	+	HORVU4Hr1G012400	
chr4H	41113699	41120291	+	HORVU4Hr1G012420	
chr4H	46530586	46535255	-	HORVU4Hr1G013370	K01557
chr4H	169641352	169659789	+	HORVU4Hr1G027660	
chr4H	361478965	361479169	+	HORVU4Hr1G045320	

chr4H	361484961	361485272	+	HORVU4Hr1G045330	K02954
chr4H	361485922	361489404	-	HORVU4Hr1G045340	
chr4H	361490376	361490975	-	HORVU4Hr1G045350	
chr4H	361491123	361491833	-	HORVU4Hr1G045360	K02967
chr4H	361492123	361495642	-	HORVU4Hr1G045370	
chr4H	400956204	400956770	-	HORVU4Hr1G049520	
chr4H	417883099	417890420	+	HORVU4Hr1G051080	
chr4H	510061239	510070825	+	HORVU4Hr1G060770	
chr4H	510639049	510643775	+	HORVU4Hr1G060840	K01689
chr4H	510644289	510652701	-	HORVU4Hr1G060850	K09377
chr4H	510806317	510808521	+	HORVU4Hr1G060870	
chr4H	510807431	510836346	-	HORVU4Hr1G060880	
chr4H	514972820	514973061	+	HORVU4Hr1G061280	
chr4H	548294448	548301852	+	HORVU4Hr1G065840	
chr4H	551855901	551858769	+	HORVU4Hr1G066270	K01723
chr4H	552163701	552170700	+	HORVU4Hr1G066290	
chr4H	552171438	552177564	+	HORVU4Hr1G066300	K20293
chr4H	552178325	552187672	+	HORVU4Hr1G066310	
chr4H	553587802	553591880	+	HORVU4Hr1G066430	K17497
chr4H	568144386	568145562	-	HORVU4Hr1G069020	K08818
chr4H	570191753	570193879	+	HORVU4Hr1G069340	
chr4H	570198175	570203753	-	HORVU4Hr1G069350	K14961
chr4H	570685107	570688166	-	HORVU4Hr1G069400	
chr4H	580160224	580171376	-	HORVU4Hr1G071270	K14521
chr4H	581783894	581788589	+	HORVU4Hr1G071730	K01858
chr4H	581790031	581793337	+	HORVU4Hr1G071740	
chr4H	586053285	586054726	+	HORVU4Hr1G072580	
chr4H	586057297	586062059	-	HORVU4Hr1G072590	K21919
chr4H	587734249	587744270	-	HORVU4Hr1G073060	
chr4H	592930774	592934000	+	HORVU4Hr1G074090	
chr4H	592935468	592938873	-	HORVU4Hr1G074110	
chr4H	593061716	593070429	+	HORVU4Hr1G074120	
chr4H	595320934	595321222	-	ENSRNA049476731	
chr4H	595326889	595332144	+	HORVU4Hr1G074740	
chr4H	595328382	595332072	-	HORVU4Hr1G074750	
chr4H	595765001	595767436	+	HORVU4Hr1G074800	K02911
chr4H	595767928	595772404	+	HORVU4Hr1G074810	K01783
chr4H	596118056	596119843	+	HORVU4Hr1G074980	
chr4H	596122913	596125612	-	HORVU4Hr1G074990	
chr4H	597422094	597430076	+	HORVU4Hr1G075250	
chr4H	602036645	602040680	-	HORVU4Hr1G076940	K15397
chr4H	608004520	608006040	+	HORVU4Hr1G078580	K14823
chr4H	608008537	608012340	-	HORVU4Hr1G078590	

chr4H	609242210	609247714	+	HORVU4Hr1G078820	K04077
chr4H	609248237	609251008	-	HORVU4Hr1G078830	K09486
chr4H	610930296	610934429	-	HORVU4Hr1G079090	
chr4H	611679942	611682960	-	HORVU4Hr1G079260	
chr4H	611690785	611692599	-	HORVU4Hr1G079270	
chr4H	613789679	613791820	+	HORVU4Hr1G079990	
chr4H	613793336	613797119	-	HORVU4Hr1G080000	K01945
chr4H	613798524	613801057	-	HORVU4Hr1G080020	
chr4H	613805852	613807701	-	HORVU4Hr1G080030	
chr4H	613808586	613814590	-	HORVU4Hr1G080050	
chr4H	615347929	615351231	+	HORVU4Hr1G080410	
chr4H	615697912	615705266	-	HORVU4Hr1G080450	
chr4H	617388840	617391479	+	HORVU4Hr1G080780	K08242
chr4H	617393608	617393889	+	HORVU4Hr1G080790	
chr4H	619752840	619755607	+	HORVU4Hr1G081590	
chr4H	619756654	619761706	-	HORVU4Hr1G081600	
chr4H	622059599	622062306	+	HORVU4Hr1G082290	K00939
chr4H	626074071	626080945	+	HORVU4Hr1G083530	
chr4H	626728557	626730255	-	HORVU4Hr1G083800	
chr4H	626735127	626735197	-	ENSRNA049445750	
chr4H	626835280	626849146	+	HORVU4Hr1G083860	K24169
chr4H	626844134	626849158	-	HORVU4Hr1G083870	
chr4H	626997423	627002978	+	HORVU4Hr1G083950	K16296
chr4H	628371674	628373285	-	HORVU4Hr1G084360	K09422
chr4H	628801846	628803652	+	HORVU4Hr1G084420	K02889
chr4H	629355424	629360910	+	HORVU4Hr1G084590	
chr4H	629483972	629487192	+	HORVU4Hr1G084600	
chr4H	629644007	629644737	+	HORVU4Hr1G084750	
chr4H	629644892	629647909	-	HORVU4Hr1G084760	K10577
chr4H	630166309	630173377	+	HORVU4Hr1G084840	
chr4H	630183883	630188317	+	HORVU4Hr1G084850	
chr4H	630855665	630860598	+	HORVU4Hr1G085050	K22390
chr4H	630862306	630862811	+	HORVU4Hr1G085060	
chr4H	630915285	630916047	+	HORVU4Hr1G085120	
chr4H	631469114	631473133	-	HORVU4Hr1G085280	
chr4H	632308238	632310222	+	HORVU4Hr1G085730	
chr4H	632310354	632313768	-	HORVU4Hr1G085740	K19323
chr4H	632316301	632325141	-	HORVU4Hr1G085750	
chr4H	633040947	633043407	+	HORVU4Hr1G085960	
chr4H	633160208	633161206	+	HORVU4Hr1G085990	
chr4H	633169005	633174352	+	HORVU4Hr1G086000	
chr4H	633178339	633181842	+	HORVU4Hr1G086010	K03036
chr4H	633181302	633184491	-	HORVU4Hr1G086020	

chr4H	636582909	636595402	-	HORVU4Hr1G087360	
chr4H	637460167	637467469	-	HORVU4Hr1G087580	
chr4H	641086962	641088357	+	HORVU4Hr1G088780	
chr4H	641185538	641189869	+	HORVU4Hr1G088860	K24175
chr4H	641191823	641192977	-	HORVU4Hr1G088870	K17794
chr4H	641293324	641295356	-	HORVU4Hr1G088930	
chr4H	642064582	642066848	+	HORVU4Hr1G089270	
chr4H	642064583	642071249	-	HORVU4Hr1G089280	
chr4H	643004384	643006737	-	HORVU4Hr1G089560	
chr4H	643012334	643013799	+	HORVU4Hr1G089580	
chr4H	643053070	643099588	-	HORVU4Hr1G089590	
chr4H	643065106	643069109	-	HORVU4Hr1G089610	K13508
chr4H	645151567	645157915	+	HORVU4Hr1G090440	K03695
chr6H	1003557	1008128	-	HORVU6Hr1G000410	
chr6H	7115759	7811829	-	HORVU6Hr1G002980	
chr6H	11516051	11518503	-	HORVU6Hr1G005250	K10528
chr6H	12304004	12305697	+	HORVU6Hr1G005510	
chr6H	30624331	30625123	-	HORVU6Hr1G014380	K10807
chr6H	31190108	31192857	+	HORVU6Hr1G014630	
chr6H	31209949	31217307	+	HORVU6Hr1G014650	K22048
chr6H	47359727	47366647	+	HORVU6Hr1G018520	K01322
chr6H	59555539	59559020	+	HORVU6Hr1G020720	K22390
chr6H	59559052	59560252	-	HORVU6Hr1G020730	
chr6H	61095579	61098767	+	HORVU6Hr1G020920	K17435
chr6H	61106759	61108080	+	HORVU6Hr1G020950	K00430
chr6H	74343823	74348510	-	HORVU6Hr1G023040	
chr6H	74352626	74367496	-	HORVU6Hr1G023050	
chr6H	74382826	74385170	+	HORVU6Hr1G023070	
chr6H	92181799	92188149	+	HORVU6Hr1G025410	
chr6H	92188590	92188713	+	ENSRNA049486160	
chr6H	109666059	109673065	+	HORVU6Hr1G027650	
chr6H	109668342	109673985	-	HORVU6Hr1G027660	
chr6H	130614643	130616732	-	HORVU6Hr1G030940	
chr6H	146581719	146584641	-	HORVU6Hr1G032940	K12486
chr6H	146603312	146607205	-	HORVU6Hr1G032960	K13448
chr6H	169991412	169996625	+	HORVU6Hr1G035370	
chr6H	396723351	396725966	+	HORVU6Hr1G059910	
chr6H	460161434	460166982	-	HORVU6Hr1G066470	
chr6H	509623775	509629249	+	HORVU6Hr1G073590	
chr6H	522762416	522768170	+	HORVU6Hr1G075950	K00703
chr6H	543053435	543055116	+	HORVU6Hr1G080830	
chr6H	567649130	567650087	+	HORVU6Hr1G088870	
chr6H	567650424	567653825	-	HORVU6Hr1G088880	

chr6H	581140213	581142855	-	HORVU6Hr1G094400	
chr6H	581144737	581147339	-	HORVU6Hr1G094410	
chr6H	581146367	581146438	+	ENSRNA049446549	
chr6H	581148325	581149332	-	HORVU6Hr1G094420	
chr6H	581260966	581272419	-	HORVU6Hr1G094470	
chr6H	582628571	582636599	+	HORVU6Hr1G095000	
chr1H	4165175	4167785	-	HORVU1Hr1G001920	K07904
chr1H	8912375	8919563	+	HORVU1Hr1G004200	K17943
chr1H	8925545	8925626	-	ENSRNA049447299	
chr1H	8928791	8928872	-	ENSRNA049447315	
chr1H	8929487	8937936	+	HORVU1Hr1G004230	K21989
chr1H	10739577	10746629	+	HORVU1Hr1G004960	K14298
chr1H	10747147	10750379	-	HORVU1Hr1G004970	
chr1H	22161111	22162182	-	HORVU1Hr1G009880	
chr1H	22166621	22176854	+	HORVU1Hr1G009900	K14301
chr1H	22179163	22179855	+	HORVU1Hr1G009910	
chr1H	23059462	23064407	+	HORVU1Hr1G010130	
chr1H	37164281	37167912	-	HORVU1Hr1G013780	
chr1H	41005783	41007234	+	HORVU1Hr1G014700	
chr1H	41010020	41012215	-	HORVU1Hr1G014710	
chr1H	41229756	41235195	+	HORVU1Hr1G014800	
chr1H	49797840	49799683	-	HORVU1Hr1G016140	
chr1H	73917857	73919159	-	HORVU1Hr1G019560	
chr1H	73920478	73928662	+	HORVU1Hr1G019570	
chr1H	73929575	73934623	-	HORVU1Hr1G019580	
chr1H	76984039	76992770	+	HORVU1Hr1G020000	
chr1H	76998224	77000693	+	HORVU1Hr1G020010	
chr1H	77001065	77004795	-	HORVU1Hr1G020020	
chr1H	80475435	80478390	+	HORVU1Hr1G020420	
chr1H	80487032	80488367	-	HORVU1Hr1G020430	
chr1H	80825648	80825788	-	HORVU1Hr1G020460	
chr1H	80827355	80832088	+	HORVU1Hr1G020470	K12449
chr1H	84326638	84338957	+	HORVU1Hr1G021120	
chr1H	84348112	84349441	+	HORVU1Hr1G021140	K00799
chr1H	84399076	84400190	+	HORVU1Hr1G021150	K00799
chr1H	85081952	85093686	-	HORVU1Hr1G021190	
chr1H	101577960	101579688	-	HORVU1Hr1G023220	
chr1H	104746260	104750117	-	HORVU1Hr1G023630	
chr1H	108166411	108190963	+	HORVU1Hr1G024170	
chr1H	109039019	109045407	-	HORVU1Hr1G024220	
chr1H	109045587	109055596	-	HORVU1Hr1G024240	
chr1H	114810823	114815858	+	HORVU1Hr1G024640	
chr1H	114851140	114855324	+	HORVU1Hr1G024660	

chr1H	114851474	114862830	-	HORVU1Hr1G024670	
chr1H	116968884	116969646	+	HORVU1Hr1G024860	
chr1H	122695030	122698867	-	HORVU1Hr1G025360	K00565
chr1H	123301589	123308893	-	HORVU1Hr1G025390	
chr1H	161035667	161047820	+	HORVU1Hr1G029110	
chr1H	163825897	163828523	+	HORVU1Hr1G029460	
chr1H	167188888	167192229	-	HORVU1Hr1G029850	K13448
chr1H	176445767	176446329	+	HORVU1Hr1G030580	
chr1H	184270591	184270927	-	HORVU1Hr1G031150	
chr1H	196891816	196896688	-	HORVU1Hr1G031970	
chr1H	206698784	206708456	+	HORVU1Hr1G033060	
chr1H	264142661	264144956	+	HORVU1Hr1G038260	
chr1H	264143785	264148150	-	HORVU1Hr1G038270	K01519
chr1H	265256443	265260727	-	HORVU1Hr1G038330	
chr1H	271292873	271294712	+	HORVU1Hr1G038890	K02889
chr1H	299740681	299751249	+	HORVU1Hr1G041790	K09338
chr1H	321614760	321618227	+	HORVU1Hr1G044040	
chr1H	321616679	321617299	+	HORVU1Hr1G044050	
chr1H	327493923	327498458	-	HORVU1Hr1G045080	
chr1H	327493945	327498471	+	HORVU1Hr1G045090	
chr1H	341763204	341772442	-	HORVU1Hr1G046630	K01873
chr1H	352060712	352077732	+	HORVU1Hr1G047640	
chr1H	353957421	353968844	+	HORVU1Hr1G047820	
chr1H	355275799	355286904	+	HORVU1Hr1G047980	
chr1H	356712296	356717513	-	HORVU1Hr1G048340	
chr1H	361583031	361592163	+	HORVU1Hr1G048720	
chr1H	361646491	361651154	+	HORVU1Hr1G048760	
chr1H	366447146	366454682	+	HORVU1Hr1G049460	
chr1H	366453344	366460489	-	HORVU1Hr1G049470	
chr1H	366507315	366510797	-	HORVU1Hr1G049480	
chr1H	377081195	377090680	+	HORVU1Hr1G050840	
chr1H	377082048	377090723	-	HORVU1Hr1G050850	
chr1H	379463530	379470722	-	HORVU1Hr1G051150	K00968
chr1H	379485428	379487713	+	HORVU1Hr1G051160	
chr1H	379493162	379494164	+	HORVU1Hr1G051180	
chr1H	380089048	380094403	-	HORVU1Hr1G051220	
chr1H	380919406	380920906	-	HORVU1Hr1G051400	
chr1H	380925803	380928956	-	HORVU1Hr1G051410	
chr1H	381313035	381328695	+	HORVU1Hr1G051460	
chr1H	381322517	381328711	-	HORVU1Hr1G051470	
chr1H	382299770	382300802	-	HORVU1Hr1G051560	
chr1H	382301293	382317173	-	HORVU1Hr1G051570	K17973
chr1H	383460565	383462205	-	HORVU1Hr1G051700	

chr1H	385625811	385629388	+	HORVU1Hr1G052010	
chr1H	387545376	387551621	-	HORVU1Hr1G052260	K04523
chr1H	387550377	387553526	+	HORVU1Hr1G052270	K03013
chr1H	387556524	387557972	+	HORVU1Hr1G052280	
chr1H	393441626	393460600	-	HORVU1Hr1G053190	
chr1H	393963164	393967064	-	HORVU1Hr1G053270	
chr1H	393971002	393974978	-	HORVU1Hr1G053280	
chr1H	394194166	394199269	-	HORVU1Hr1G053320	K00826
chr1H	394411517	394417153	+	HORVU1Hr1G053340	
chr1H	394417623	394420439	-	HORVU1Hr1G053350	
chr1H	395873135	395875994	+	HORVU1Hr1G053510	
chr1H	397672141	397673986	-	HORVU1Hr1G053890	
chr1H	397861757	397861827	-	ENSRNA049447675	
chr1H	398276256	398284346	-	HORVU1Hr1G053930	
chr1H	398281263	398281754	-	HORVU1Hr1G053940	
chr1H	398652421	398808254	-	HORVU1Hr1G053990	
chr1H	399234596	399237714	-	HORVU1Hr1G054060	
chr1H	400546354	400547881	+	HORVU1Hr1G054200	
chr1H	401040803	401045363	-	HORVU1Hr1G054240	
chr1H	406525155	406531073	+	HORVU1Hr1G055510	
chr1H	406533021	406535450	+	HORVU1Hr1G055520	
chr1H	406997955	407009508	+	HORVU1Hr1G055580	
chr1H	410771953	410775529	-	HORVU1Hr1G056070	K00652
chr1H	414864751	414869005	+	HORVU1Hr1G056510	
chr1H	415278902	415281508	+	HORVU1Hr1G056630	K06118
chr1H	420229299	420233386	-	HORVU1Hr1G057570	K03955
chr1H	422557131	422560475	+	HORVU1Hr1G057950	
chr1H	435593439	435596729	-	HORVU1Hr1G059960	
chr1H	435667107	435670374	+	HORVU1Hr1G059990	K09537
chr1H	437148534	437151974	+	HORVU1Hr1G060100	K11363
chr1H	437159294	437160728	-	HORVU1Hr1G060110	
chr1H	437163783	437164350	+	HORVU1Hr1G060130	
chr1H	437275262	437279877	+	HORVU1Hr1G060140	
chr1H	437281398	437287737	-	HORVU1Hr1G060150	
chr1H	437443399	437450543	-	HORVU1Hr1G060180	K14293
chr1H	437443473	437446092	+	HORVU1Hr1G060190	
chr1H	443090309	443090737	-	HORVU1Hr1G061070	
chr1H	443090314	443091482	+	HORVU1Hr1G061080	
chr1H	443091773	443131375	-	HORVU1Hr1G061090	
chr1H	444526428	444530074	-	HORVU1Hr1G061430	K14009
chr1H	448818340	448821848	+	HORVU1Hr1G062290	
chr1H	451995060	451999971	+	HORVU1Hr1G062940	
chr1H	451995105	452001043	-	HORVU1Hr1G062950	

chr1H	454317352	454321668	+	HORVU1Hr1G063320	
chr1H	470634738	470637183	-	HORVU1Hr1G066120	
chr1H	471472632	471476335	-	HORVU1Hr1G066240	K03809
chr1H	472946083	472947141	+	HORVU1Hr1G066500	K02116
chr1H	472948820	472951168	-	HORVU1Hr1G066510	
chr1H	473008356	473010850	+	HORVU1Hr1G066530	
chr1H	477270747	477276317	-	HORVU1Hr1G067200	
chr1H	487882055	487884794	-	HORVU1Hr1G069990	
chr1H	518159277	518160365	-	HORVU1Hr1G077680	K14516
chr1H	518554761	518558462	-	HORVU1Hr1G077790	
chr1H	522495889	522497697	+	HORVU1Hr1G079210	
chr1H	524725774	524730118	-	HORVU1Hr1G080290	K14497
chr1H	525014151	525021376	-	HORVU1Hr1G080320	
chr1H	525562530	525565898	+	HORVU1Hr1G080510	
chr1H	525567058	525571275	+	HORVU1Hr1G080520	K14156
chr1H	527443694	527446128	+	HORVU1Hr1G081300	K09419
chr1H	527446416	527451361	-	HORVU1Hr1G081310	
chr1H	527813325	527814684	+	HORVU1Hr1G081500	
chr1H	532367085	532371148	+	HORVU1Hr1G083720	K10758
chr1H	532368566	532373925	-	HORVU1Hr1G083730	K10758
chr1H	540571001	540572989	-	HORVU1Hr1G087870	
chr1H	540581949	540584326	-	HORVU1Hr1G087880	
chr1H	540584485	540593837	-	HORVU1Hr1G087900	
chr1H	541346878	541351320	+	HORVU1Hr1G088270	
chr1H	544690027	544692914	-	HORVU1Hr1G089710	K15397
chr1H	544734788	544736482	+	HORVU1Hr1G089730	K22068
chr1H	544741794	544742915	+	HORVU1Hr1G089760	K02935
chr1H	544744250	544746044	-	HORVU1Hr1G089770	K09422
chr1H	544749530	544755172	+	HORVU1Hr1G089780	
chr1H	544903067	544906482	+	HORVU1Hr1G089820	
chr1H	544907085	544909570	-	HORVU1Hr1G089830	K15095
chr1H	544918998	544920368	-	HORVU1Hr1G089840	
chr1H	545123927	545127861	-	HORVU1Hr1G090020	K01792
chr1H	545321477	545323501	+	HORVU1Hr1G090100	
chr1H	546270397	546273711	+	HORVU1Hr1G090450	
chr1H	546276302	546276823	+	HORVU1Hr1G090460	
chr1H	546836401	546839215	-	HORVU1Hr1G090730	K15397
chr1H	546839963	546843526	-	HORVU1Hr1G090740	
chr1H	552686367	552688044	-	HORVU1Hr1G093350	
chr1H	554147341	554150184	+	HORVU1Hr1G093860	
chrUn	2088100	2089839	+	HORVU0Hr1G000320	
chrUn	2092196	2094795	+	HORVU0Hr1G000340	
chrUn	7499051	7499564	-	HORVU0Hr1G001420	

chrUn	7503092	7504913	+	HORVU0Hr1G001430	
chrUn	7507037	7511309	-	HORVU0Hr1G001440	
chrUn	7514888	7516723	+	HORVU0Hr1G001450	K14397
chrUn	7518568	7521884	+	HORVU0Hr1G001460	
chrUn	11134211	11134795	-	HORVU0Hr1G002480	
chrUn	30719336	30723028	+	HORVU0Hr1G005570	
chrUn	84272792	84274315	-	HORVU0Hr1G015440	
chrUn	86842236	86843359	+	HORVU0Hr1G016180	K06995
chrUn	86844210	86852751	+	HORVU0Hr1G016190	
chrUn	86854468	86857218	+	HORVU0Hr1G016220	K01537
chrUn	109690093	109695375	+	HORVU0Hr1G020800	
chrUn	109695370	109699020	-	HORVU0Hr1G020810	
chrUn	110178333	110179149	+	HORVU0Hr1G021040	
chrUn	110179208	110183094	-	HORVU0Hr1G021050	
chrUn	110183556	110187206	-	HORVU0Hr1G021060	
chrUn	113429049	113430432	-	HORVU0Hr1G021850	
chrUn	113431335	113434146	+	HORVU0Hr1G021860	
chrUn	113597769	113600997	+	HORVU0Hr1G021960	K05542
chrUn	113597770	113603041	-	HORVU0Hr1G021970	
chrUn	113603037	113607130	+	HORVU0Hr1G021980	
chrUn	113607305	113609587	-	HORVU0Hr1G022000	
chrUn	113609647	113610944	-	HORVU0Hr1G022020	
chrUn	114762265	114764964	-	HORVU0Hr1G022480	
chrUn	114767419	114770863	-	HORVU0Hr1G022500	
chrUn	114833807	114837176	+	HORVU0Hr1G022570	
chrUn	114837829	114842665	+	HORVU0Hr1G022580	K14831
chrUn	114845124	114896567	+	HORVU0Hr1G022590	
chrUn	116818257	116823079	+	HORVU0Hr1G023200	
chrUn	249627284	249629122	-	HORVU0Hr1G040230	
chrUn	249629274	249629895	+	HORVU0Hr1G040240	
chrUn	249631240	249632422	+	HORVU0Hr1G040270	
chrUn	249632640	249633128	-	HORVU0Hr1G040280	
chrUn	249633502	249634434	-	HORVU0Hr1G040290	
chrUn	249635218	249636470	+	HORVU0Hr1G040300	
chrUn	249636707	249641694	+	HORVU0Hr1G040310	K03012
chrUn	249636783	249641729	-	HORVU0Hr1G040320	K03012

Table S12. Selective genes between Wb-T and Cb-C.

**Chromosome**	**Start position**	**End position**	**Strand**	**Gene ID**	**KEGG ID**
chr2H	39666071	39673442	+	HORVU2Hr1G017170	
chr2H	40313666	40315533	-	HORVU2Hr1G017300	

chr2H	50888142	50888963	+	HORVU2Hr1G019580	
chr2H	51955647	51957502	+	HORVU2Hr1G019790	
chr2H	125569689	125571566	-	HORVU2Hr1G032360	
chr2H	196889691	196892849	-	HORVU2Hr1G040570	K00873
chr2H	234820504	234831306	+	HORVU2Hr1G044910	
chr2H	582422103	582422183	-	ENSRNA049439149	
chr2H	582422344	582428411	+	HORVU2Hr1G080370	K01551
chr2H	599026212	599031507	-	HORVU2Hr1G082590	
chr2H	620401652	620404709	-	HORVU2Hr1G085870	
chr2H	620408029	620412655	-	HORVU2Hr1G085880	K11090
chr2H	620613412	620632316	+	HORVU2Hr1G085910	
chr2H	638240842	638242321	-	HORVU2Hr1G089310	
chr2H	640052177	640066173	+	HORVU2Hr1G089630	
chr2H	640052329	640066310	-	HORVU2Hr1G089640	
chr2H	640727115	640737542	+	HORVU2Hr1G089830	
chr2H	648515999	648516959	-	HORVU2Hr1G091580	
chr2H	649203485	649232890	-	HORVU2Hr1G091680	
chr2H	649733130	649736343	+	HORVU2Hr1G091850	
chr2H	650773966	650775199	-	HORVU2Hr1G092070	K14488
chr2H	651528086	651534019	-	HORVU2Hr1G092250	K11498
chr2H	651534346	651540462	-	HORVU2Hr1G092260	K10728
chr2H	652416364	652419744	+	HORVU2Hr1G092340	
chr2H	653323469	653360286	-	HORVU2Hr1G092420	
chr2H	654157450	654168952	-	HORVU2Hr1G092600	
chr2H	668997933	669004655	+	HORVU2Hr1G095210	K09338
chr2H	669005028	669009842	-	HORVU2Hr1G095220	K10755
chr2H	669560144	669562123	+	HORVU2Hr1G095390	
chr2H	680332013	680337386	-	HORVU2Hr1G097760	
chr2H	685376486	685385984	+	HORVU2Hr1G098860	
chr2H	704386639	704387155	-	HORVU2Hr1G104670	K15032
chr2H	704387240	704387847	-	HORVU2Hr1G104680	K14156
chr2H	704389810	704392568	+	HORVU2Hr1G104690	
chr2H	704875529	704885840	+	HORVU2Hr1G104900	
chr2H	704886345	704891615	+	HORVU2Hr1G104920	K05387
chr2H	706342872	706346612	-	HORVU2Hr1G105380	
chr2H	706347266	706352048	-	HORVU2Hr1G105390	
chr2H	708177034	708179766	-	HORVU2Hr1G105930	
chr2H	708253613	708259832	-	HORVU2Hr1G105980	
chr2H	708262945	708266739	-	HORVU2Hr1G106010	K19985
chr2H	708558226	708561788	+	HORVU2Hr1G106080	
chr2H	708562341	708565774	-	HORVU2Hr1G106090	
chr2H	708637015	708689912	-	HORVU2Hr1G106110	
chr2H	710599715	710603129	+	HORVU2Hr1G106810	

chr2H	710604987	710610388	+	HORVU2Hr1G106820	
chr2H	710833148	710844185	-	HORVU2Hr1G106880	
chr2H	714112939	714131564	+	HORVU2Hr1G108380	
chr2H	716480145	716484046	-	HORVU2Hr1G109380	K16298
chr2H	718430317	718437579	-	HORVU2Hr1G109960	
chr2H	720338804	720342575	-	HORVU2Hr1G110580	K15148
chr2H	721943807	721946820	-	HORVU2Hr1G111070	
chr2H	722484027	722488008	-	HORVU2Hr1G111340	
chr2H	723000994	723005790	+	HORVU2Hr1G111530	
chr2H	723652502	723658875	-	HORVU2Hr1G111640	
chr2H	724700867	724701548	-	HORVU2Hr1G111990	K15255
chr2H	724706982	724710722	-	HORVU2Hr1G112000	
chr2H	725822715	725826075	-	HORVU2Hr1G112480	
chr2H	726001105	726004354	+	HORVU2Hr1G112580	K05986
chr2H	726007333	726011807	+	HORVU2Hr1G112590	
chr2H	726010794	726015308	-	HORVU2Hr1G112600	K05986
chr2H	726018842	726034727	-	HORVU2Hr1G112620	
chr2H	727206601	727211591	+	HORVU2Hr1G113050	
chr2H	727208236	727216963	-	HORVU2Hr1G113060	
chr2H	728031852	728032855	+	HORVU2Hr1G113300	
chr2H	728037581	728038321	+	HORVU2Hr1G113310	
chr2H	728042593	728046120	-	HORVU2Hr1G113320	K14570
chr2H	728158118	728160470	-	HORVU2Hr1G113330	
chr2H	731302774	731306944	+	HORVU2Hr1G114260	K00864
chr2H	732543347	732556439	-	HORVU2Hr1G114490	
chr2H	732688460	732690886	-	HORVU2Hr1G114610	K19730
chr2H	732691119	732698865	+	HORVU2Hr1G114640	K13998
chr2H	732701181	732707697	+	HORVU2Hr1G114660	
chr2H	732704699	732706055	-	HORVU2Hr1G114670	
chr2H	735292922	735304274	+	HORVU2Hr1G115690	K00891
chr2H	735297582	735304406	-	HORVU2Hr1G115700	K00891
chr2H	735306656	735309864	+	HORVU2Hr1G115710	
chr2H	735380131	735382200	-	HORVU2Hr1G115730	
chr2H	738354470	738355057	+	HORVU2Hr1G116570	K20791
chr2H	739038891	739324196	-	HORVU2Hr1G116880	
chr2H	739334630	739335172	-	HORVU2Hr1G117060	
chr2H	739342287	739343776	+	HORVU2Hr1G117070	K07999
chr2H	742197934	742204374	+	HORVU2Hr1G118320	
chr2H	742206222	742207877	-	HORVU2Hr1G118340	
chr2H	742278295	742284032	+	HORVU2Hr1G118410	K23953
chr2H	742284835	742287234	-	HORVU2Hr1G118420	
chr2H	743137237	743140775	+	HORVU2Hr1G118670	K00869
chr2H	743141590	743144509	-	HORVU2Hr1G118680	

chr2H	746557796	746557914	+	ENSRNA049481293	
chr2H	746560785	746560900	+	ENSRNA049481304	
chr2H	746562978	746567627	-	HORVU2Hr1G119900	
chr2H	746563699	746563814	+	ENSRNA049481301	
chr2H	746569148	746574176	-	HORVU2Hr1G119920	
chr2H	753391612	753392265	-	HORVU2Hr1G122560	
chr2H	753393466	753393972	-	HORVU2Hr1G122570	
chr2H	753395800	753397331	+	HORVU2Hr1G122580	
chr2H	753496840	753497543	+	HORVU2Hr1G122630	
chr2H	753497875	753501250	+	HORVU2Hr1G122640	
chr2H	753498170	753501261	-	HORVU2Hr1G122650	
chr2H	753502776	753505395	+	HORVU2Hr1G122660	
chr2H	753923725	753928860	-	HORVU2Hr1G122850	
chr2H	758674366	758675622	-	HORVU2Hr1G124540	
chr2H	758798962	758800868	+	HORVU2Hr1G124610	
chr2H	759279572	759283062	+	HORVU2Hr1G124850	K05906
chr2H	759290209	759292377	-	HORVU2Hr1G124860	
chr2H	765910275	765917856	+	HORVU2Hr1G127090	
chr2H	765910275	765919163	-	HORVU2Hr1G127100	
chr2H	765936571	765937404	-	HORVU2Hr1G127120	
chr2H	765943371	765945953	-	HORVU2Hr1G127130	
chr2H	767240974	767272689	+	HORVU2Hr1G127440	
chr3H	1501667	1503269	+	HORVU3Hr1G000560	
chr3H	1505375	1508124	+	HORVU3Hr1G000570	K00052
chr3H	1510612	1510696	-	ENSRNA049434181	
chr3H	1511897	1518900	-	HORVU3Hr1G000580	K22544
chr3H	11698452	11703468	+	HORVU3Hr1G004510	K24736
chr3H	13428510	13434368	+	HORVU3Hr1G005400	
chr3H	13434376	13438303	-	HORVU3Hr1G005410	
chr3H	13440000	13443931	+	HORVU3Hr1G005420	
chr3H	13445141	13448185	-	HORVU3Hr1G005430	K04506
chr3H	14008525	14009025	+	HORVU3Hr1G005700	
chr3H	14107206	14111742	-	HORVU3Hr1G005770	
chr3H	14114434	14117915	+	HORVU3Hr1G005780	
chr3H	14192104	14193862	+	HORVU3Hr1G005820	
chr3H	14202606	14205398	-	HORVU3Hr1G005830	
chr3H	14864392	14865434	+	HORVU3Hr1G005980	K22856
chr3H	15206021	15208412	+	HORVU3Hr1G006180	K01213
chr3H	15212799	15215795	+	HORVU3Hr1G006200	
chr3H	15250416	15253110	+	HORVU3Hr1G006230	K23754
chr3H	15253191	15254140	+	HORVU3Hr1G006240	K15803
chr3H	15255304	15258498	-	HORVU3Hr1G006250	
chr3H	15300748	15302081	-	HORVU3Hr1G006310	

chr3H	15309409	15320174	+	HORVU3Hr1G006320	
chr3H	17175802	17177553	+	HORVU3Hr1G007060	
chr3H	17261086	17261893	+	HORVU3Hr1G007070	
chr3H	17267754	17270761	+	HORVU3Hr1G007080	K15139
chr3H	17421800	17421872	-	ENSRNA049434223	
chr3H	17955161	17955355	-	HORVU3Hr1G007530	
chr3H	17959927	17980985	-	HORVU3Hr1G007540	
chr3H	19016502	19019476	+	HORVU3Hr1G008400	
chr3H	19022221	19025999	-	HORVU3Hr1G008420	
chr3H	20026353	20201668	-	HORVU3Hr1G009360	
chr3H	20026465	20201833	+	HORVU3Hr1G009370	
chr3H	20503965	20505366	+	HORVU3Hr1G009560	
chr3H	23089798	23098821	+	HORVU3Hr1G010460	
chr3H	23092314	23099614	-	HORVU3Hr1G010470	
chr3H	23309016	23315926	-	HORVU3Hr1G010570	K14638
chr3H	24938037	24942947	+	HORVU3Hr1G011540	K23871
chr3H	24949143	24952430	+	HORVU3Hr1G011560	K15414
chr3H	30655192	30662805	+	HORVU3Hr1G013820	K13176
chr3H	30922678	30926507	+	HORVU3Hr1G013860	
chr3H	30927341	30935804	-	HORVU3Hr1G013880	K10527
chr3H	32215742	32220605	-	HORVU3Hr1G014120	
chr3H	32229816	32232000	-	HORVU3Hr1G014140	
chr3H	33511574	33514482	+	HORVU3Hr1G014580	K12198
chr3H	34669953	34674574	+	HORVU3Hr1G014890	K13457
chr3H	36651148	36659153	-	HORVU3Hr1G015570	K15027
chr3H	38304662	38308839	+	HORVU3Hr1G016000	K03845
chr3H	38309028	38316910	+	HORVU3Hr1G016010	K10684
chr3H	47009339	47013112	-	HORVU3Hr1G018130	K14809
chr3H	52590346	52593305	+	HORVU3Hr1G019240	
chr3H	52596223	52605278	-	HORVU3Hr1G019260	
chr3H	57508046	57511540	-	HORVU3Hr1G019770	
chr3H	61065898	61070706	+	HORVU3Hr1G020100	
chr3H	63256964	63266250	+	HORVU3Hr1G020340	K09272
chr3H	63436467	63441228	-	HORVU3Hr1G020400	
chr3H	64380575	64394865	+	HORVU3Hr1G020580	
chr3H	65663711	65671005	+	HORVU3Hr1G020810	
chr3H	65673316	65675317	+	HORVU3Hr1G020830	K22684
chr3H	65673316	65687992	-	HORVU3Hr1G020840	
chr3H	66851391	66851461	-	ENSRNA049434451	
chr3H	78237356	78239333	-	HORVU3Hr1G022260	
chr3H	78241796	78243136	-	HORVU3Hr1G022270	
chr3H	111849654	111854296	-	HORVU3Hr1G026870	
chr3H	113622335	113629361	+	HORVU3Hr1G027070	

chr3H	118267916	118270871	+	HORVU3Hr1G027470	
chr3H	119233220	119249031	+	HORVU3Hr1G027580	
chr3H	134887089	134891126	+	HORVU3Hr1G029320	K03627
chr3H	142551261	142552617	+	HORVU3Hr1G030150	
chr3H	160759519	160760457	+	HORVU3Hr1G031900	
chr3H	160765584	160769048	-	HORVU3Hr1G031930	
chr3H	162893194	162896873	-	HORVU3Hr1G032110	
chr3H	174823674	174829266	+	HORVU3Hr1G033350	K23166
chr3H	174829790	174830461	+	HORVU3Hr1G033360	
chr3H	209705434	209707313	-	HORVU3Hr1G037030	
chr3H	209708379	209712895	-	HORVU3Hr1G037040	
chr3H	209724469	209728558	+	HORVU3Hr1G037050	
chr3H	212000808	212019643	+	HORVU3Hr1G037310	
chr3H	213937394	213943684	+	HORVU3Hr1G037600	K13447
chr3H	215058374	215065252	-	HORVU3Hr1G037780	K23678
chr3H	216207971	216208192	-	HORVU3Hr1G037870	
chr3H	216208340	216208702	-	HORVU3Hr1G037880	
chr3H	216208892	216209068	-	HORVU3Hr1G037890	
chr3H	219053861	219059045	-	HORVU3Hr1G038060	
chr3H	219970698	219977303	+	HORVU3Hr1G038200	
chr3H	424270621	424275307	+	HORVU3Hr1G056650	
chr3H	431180711	431184807	-	HORVU3Hr1G057440	
chr3H	432097070	432118808	-	HORVU3Hr1G057530	
chr3H	438233271	438235842	+	HORVU3Hr1G058150	
chr3H	446058318	446062780	-	HORVU3Hr1G059060	K00031
chr3H	447236706	447245971	+	HORVU3Hr1G059140	K01897
chr3H	479983413	480017371	-	HORVU3Hr1G062760	
chr3H	482165393	482176766	+	HORVU3Hr1G063050	K00264
chr3H	482884414	482889270	+	HORVU3Hr1G063250	
chr3H	493841629	493847850	+	HORVU3Hr1G064740	K01056
chr3H	494195872	494209570	-	HORVU3Hr1G064750	
chr3H	494198593	494199879	+	HORVU3Hr1G064760	
chr3H	497609992	497617860	+	HORVU3Hr1G065260	K05658
chr3H	498131674	498136884	+	HORVU3Hr1G065350	
chr3H	498131881	498136937	-	HORVU3Hr1G065360	K05389
chr3H	507036443	507063465	+	HORVU3Hr1G066700	
chr3H	543667426	543667944	-	HORVU3Hr1G072000	
chr3H	552738456	552750250	+	HORVU3Hr1G073220	
chr3H	558953626	558953697	-	ENSRNA049436027	
chr3H	558953771	558953844	-	ENSRNA049436036	
chr3H	558954084	558957766	+	HORVU3Hr1G074620	K01285
chr3H	559136249	559136491	-	HORVU3Hr1G074670	
chr3H	559145074	559148685	-	HORVU3Hr1G074680	

chr3H	559794503	559801078	+	HORVU3Hr1G074770	K12617
chr3H	561431835	561438847	+	HORVU3Hr1G075050	
chr3H	562266303	562282316	+	HORVU3Hr1G075220	
chr3H	563053326	563061504	+	HORVU3Hr1G075340	
chr3H	564770923	564779425	-	HORVU3Hr1G075540	
chr3H	567578333	567582966	+	HORVU3Hr1G076060	K22390
chr3H	574246394	574247535	-	HORVU3Hr1G077660	
chr3H	575421996	575424645	+	HORVU3Hr1G077960	K03113
chr3H	576142004	576149293	-	HORVU3Hr1G078090	K00074
chr3H	576142353	576146580	+	HORVU3Hr1G078100	K00074
chr3H	578142357	578143352	+	HORVU3Hr1G078420	K06981
chr3H	578461509	578465414	+	HORVU3Hr1G078490	
chr3H	578730032	578860260	-	HORVU3Hr1G078530	
chr3H	580440661	580444139	+	HORVU3Hr1G078820	
chr3H	580440772	580443838	-	HORVU3Hr1G078830	
chr3H	581094773	581098244	+	HORVU3Hr1G078940	K07375
chr3H	582534472	582542774	+	HORVU3Hr1G079270	K00109
chr3H	583337677	583341550	+	HORVU3Hr1G079440	K24405
chr3H	583341763	583346358	-	HORVU3Hr1G079450	
chr3H	586480063	586481307	-	HORVU3Hr1G080010	K00326
chr3H	589362224	589366802	+	HORVU3Hr1G080530	K15633
chr3H	590802757	590806060	+	HORVU3Hr1G080810	
chr3H	590806885	590818660	-	HORVU3Hr1G080820	
chr3H	590910969	590914623	-	HORVU3Hr1G080830	K00487
chr3H	593197380	593201865	+	HORVU3Hr1G081100	
chr3H	595660559	595667334	-	HORVU3Hr1G081660	
chr3H	598334946	598337882	+	HORVU3Hr1G082230	K22733
chr3H	598337922	598346861	-	HORVU3Hr1G082240	
chr3H	598788471	598796755	+	HORVU3Hr1G082310	K12619
chr3H	598809309	598810131	+	HORVU3Hr1G082320	
chr3H	599122576	599125180	+	HORVU3Hr1G082460	
chr3H	601470215	601474659	+	HORVU3Hr1G083090	K14948
chr3H	601475494	601480058	-	HORVU3Hr1G083110	
chr3H	602089588	602094803	+	HORVU3Hr1G083180	K22943
chr3H	602659829	602660254	-	HORVU3Hr1G083280	
chr3H	602773272	602776454	+	HORVU3Hr1G083300	K12622
chr3H	604261977	604267781	+	HORVU3Hr1G083540	
chr3H	604263642	604267797	-	HORVU3Hr1G083550	K22745
chr3H	605209510	605218490	-	HORVU3Hr1G083780	
chr3H	605212593	605212672	-	ENSRNA049436258	
chr3H	605212795	605218151	+	HORVU3Hr1G083790	K17917
chr3H	605220151	605221405	+	HORVU3Hr1G083800	
chr3H	606305501	606306847	+	HORVU3Hr1G084110	

chr3H	606309479	606311167	+	HORVU3Hr1G084120	
chr3H	606318504	606326254	-	HORVU3Hr1G084170	
chr3H	606519570	606522571	-	HORVU3Hr1G084260	
chr3H	606944820	606948009	-	HORVU3Hr1G084310	K02865
chr3H	607296280	607298550	+	HORVU3Hr1G084380	
chr3H	607571715	607573675	-	HORVU3Hr1G084410	
chr3H	607582405	607586340	-	HORVU3Hr1G084420	
chr3H	608249205	608253045	+	HORVU3Hr1G084520	K12873
chr3H	608253179	608257632	+	HORVU3Hr1G084530	
chr3H	608257844	608263105	-	HORVU3Hr1G084540	
chr3H	611543439	611547591	-	HORVU3Hr1G085210	K13463
chr3H	612001045	612007393	-	HORVU3Hr1G085320	
chr3H	612482009	612482425	-	HORVU3Hr1G085340	
chr3H	612487276	612491283	+	HORVU3Hr1G085350	
chr3H	612492738	612496699	+	HORVU3Hr1G085360	
chr3H	612819203	612827928	+	HORVU3Hr1G085460	
chr3H	613897934	613903093	-	HORVU3Hr1G085720	
chr3H	614506659	614516560	+	HORVU3Hr1G085820	K00966
chr3H	614506741	614516670	-	HORVU3Hr1G085830	K00966
chr3H	618052174	618055308	+	HORVU3Hr1G086630	K02879
chr3H	618056041	618060093	+	HORVU3Hr1G086650	
chr3H	621227948	621233457	-	HORVU3Hr1G087470	
chr3H	656380934	656386027	+	HORVU3Hr1G097680	
chr3H	677734589	677740710	+	HORVU3Hr1G109370	
chr3H	679253174	679260455	+	HORVU3Hr1G109740	
chr5H	23245645	23247052	-	HORVU5Hr1G009600	K02920
chr5H	23346295	23348364	+	HORVU5Hr1G009610	
chr5H	38555667	38562124	+	HORVU5Hr1G012770	K00547
chr5H	38557723	38562136	-	HORVU5Hr1G012780	K00547
chr5H	81572972	81576544	-	HORVU5Hr1G019100	
chr5H	96618827	96627750	-	HORVU5Hr1G020800	
chr5H	130588927	130594377	-	HORVU5Hr1G024340	K00850
chr5H	167331874	167339562	+	HORVU5Hr1G028360	K03263
chr5H	169904862	169914285	+	HORVU5Hr1G028770	K09506
chr5H	169909813	169911039	+	HORVU5Hr1G028780	
chr5H	184424429	184440566	-	HORVU5Hr1G030040	
chr5H	242763832	242768228	+	HORVU5Hr1G035220	
chr5H	246407973	246427633	-	HORVU5Hr1G035440	
chr5H	246411822	246411974	+	HORVU5Hr1G035470	
chr5H	266750649	266750726	+	ENSRNA049475627	
chr5H	266751365	266752357	-	HORVU5Hr1G037150	
chr5H	266753711	266763867	+	HORVU5Hr1G037160	K17545
chr5H	266762421	266773231	-	HORVU5Hr1G037200	

chr5H	270203770	270207437	-	HORVU5Hr1G037600	K03686
chr5H	273896608	273897854	+	HORVU5Hr1G037890	
chr5H	273898538	273915692	-	HORVU5Hr1G037910	
chr5H	273913641	273942237	+	HORVU5Hr1G037920	
chr5H	280775389	280777976	+	HORVU5Hr1G038920	K02997
chr5H	284176627	284183906	+	HORVU5Hr1G039210	
chr5H	286183119	286184251	-	HORVU5Hr1G039400	
chr5H	286690669	286711619	+	HORVU5Hr1G039430	
chr5H	288433498	288439832	+	HORVU5Hr1G039620	
chr5H	290716942	290720528	+	HORVU5Hr1G039850	K10355
chr5H	293329487	293336347	+	HORVU5Hr1G040040	
chr5H	293336044	293339459	-	HORVU5Hr1G040050	K00416
chr5H	295088520	295090806	-	HORVU5Hr1G040160	
chr5H	296068400	296073717	+	HORVU5Hr1G040190	
chr5H	296724701	296734969	+	HORVU5Hr1G040210	
chr5H	301880519	301885577	+	HORVU5Hr1G040540	
chr5H	315391439	315396861	-	HORVU5Hr1G041530	
chr5H	348281526	348288722	-	HORVU5Hr1G045020	
chr5H	349058357	349066233	+	HORVU5Hr1G045150	K01209
chr5H	349701419	349705399	-	HORVU5Hr1G045180	K14432
chr5H	357322486	357326766	+	HORVU5Hr1G045930	
chr5H	359517904	359532721	-	HORVU5Hr1G046250	
chr5H	359527202	359529307	+	HORVU5Hr1G046260	
chr5H	359535548	359536767	-	HORVU5Hr1G046280	
chr5H	361462479	361467032	-	HORVU5Hr1G046490	
chr5H	416429927	416435918	-	HORVU5Hr1G053150	
chr5H	464320300	464336491	+	HORVU5Hr1G059430	
chr5H	480973094	480984107	+	HORVU5Hr1G061530	
chr5H	480978299	480996766	-	HORVU5Hr1G061540	
chr5H	483236263	483236977	-	HORVU5Hr1G062080	K01520
chr5H	483237696	483244300	+	HORVU5Hr1G062090	K14085
chr5H	484372038	484375692	+	HORVU5Hr1G062220	
chr5H	565764418	565774332	+	HORVU5Hr1G081790	
chr5H	581801752	581806256	-	HORVU5Hr1G088430	
chr5H	608567838	608568942	+	HORVU5Hr1G098710	
chr5H	608578257	608580493	-	HORVU5Hr1G098720	
chr5H	608584118	608584590	+	HORVU5Hr1G098730	
chr5H	618537775	618542148	-	HORVU5Hr1G104200	
chr5H	626123504	626124759	+	HORVU5Hr1G107180	
chr5H	626125556	626133605	+	HORVU5Hr1G107190	
chr5H	626140764	626142586	+	HORVU5Hr1G107210	K03671
chr5H	634566325	634568382	-	HORVU5Hr1G110940	
chr5H	639807894	639809373	+	HORVU5Hr1G112580	

chr5H	640025889	640030224	-	HORVU5Hr1G112710	
chr5H	640375896	640380995	+	HORVU5Hr1G112850	
chr5H	642237666	642240639	+	HORVU5Hr1G113620	
chr5H	642240822	642246866	-	HORVU5Hr1G113630	
chr5H	642937025	642939601	-	HORVU5Hr1G113950	
chr5H	643367175	643367993	-	HORVU5Hr1G114190	
chr5H	643377919	643378912	+	HORVU5Hr1G114200	
chr5H	643433504	643436058	-	HORVU5Hr1G114250	K07904
chr5H	643444530	643447569	+	HORVU5Hr1G114260	
chr5H	644028373	644028732	-	HORVU5Hr1G114520	
chr5H	644035801	644039389	-	HORVU5Hr1G114530	K19882
chr5H	646681145	646686179	+	HORVU5Hr1G115490	
chr5H	646682608	646686179	-	HORVU5Hr1G115500	
chr5H	646848440	646853969	+	HORVU5Hr1G115530	
chr5H	647629286	647630979	+	HORVU5Hr1G116310	
chr5H	653908194	653918421	+	HORVU5Hr1G118860	
chr5H	657762006	657770015	-	HORVU5Hr1G120520	
chr5H	657767507	657767578	+	ENSRNA049443290	
chr5H	658376226	658378900	-	HORVU5Hr1G120800	K06911
chr5H	661995105	661999262	+	HORVU5Hr1G122350	
chr5H	662606046	662611272	+	HORVU5Hr1G122640	K11092
chr5H	662742090	662744634	+	HORVU5Hr1G122750	
chr5H	662745649	662958544	+	HORVU5Hr1G122760	
chr5H	662806066	662812892	+	HORVU5Hr1G122810	
chr5H	662814297	662817523	+	HORVU5Hr1G122820	
chr5H	663355222	663363898	+	HORVU5Hr1G123080	
chr5H	663395230	663397277	-	HORVU5Hr1G123150	
chr5H	663397690	663402446	-	HORVU5Hr1G123160	K13989
chr5H	663496644	663500015	-	HORVU5Hr1G123240	
chr5H	663885157	663889732	+	HORVU5Hr1G123370	K14403
chr5H	665176518	665181669	-	HORVU5Hr1G124090	
chr7H	2209634	2212132	+	HORVU7Hr1G001010	
chr7H	2209871	2353258	-	HORVU7Hr1G001020	
chr7H	3848027	3856319	+	HORVU7Hr1G001830	
chr7H	4094645	4097230	+	HORVU7Hr1G002090	
chr7H	4101379	4104133	+	HORVU7Hr1G002110	
chr7H	4108971	4109165	-	ENSRNA049489220	
chr7H	4109856	4110159	-	ENSRNA049489215	
chr7H	4278136	4283136	+	HORVU7Hr1G002200	K02641
chr7H	4279678	4283236	-	HORVU7Hr1G002210	K02641
chr7H	8358004	8367149	+	HORVU7Hr1G006370	
chr7H	41832522	41834863	-	HORVU7Hr1G025380	
chr7H	41834939	41841912	-	HORVU7Hr1G025390	

chr7H	41843877	41845648	-	HORVU7Hr1G025400	
chr7H	42673891	42673962	+	ENSRNA049442250	
chr7H	42674384	42674455	+	ENSRNA049442241	
chr7H	42684121	42690645	-	HORVU7Hr1G025620	
chr7H	42695636	42700024	+	HORVU7Hr1G025650	
chr7H	42700406	42700477	-	ENSRNA049440354	
chr7H	42957041	42958811	-	HORVU7Hr1G025720	K09422
chr7H	42960311	42964316	+	HORVU7Hr1G025730	K24418
chr7H	53818355	53824943	+	HORVU7Hr1G028910	
chr7H	53823972	53827605	-	HORVU7Hr1G028920	
chr7H	54080027	54083868	+	HORVU7Hr1G029060	
chr7H	62540594	62540770	+	HORVU7Hr1G030990	
chr7H	62540774	62541217	+	HORVU7Hr1G031000	
chr7H	62541536	62541691	+	HORVU7Hr1G031010	
chr7H	62744251	62752110	+	HORVU7Hr1G031100	K14311
chr7H	65632288	65636503	-	HORVU7Hr1G032070	
chr7H	65639396	65647425	-	HORVU7Hr1G032080	K12818
chr7H	91967526	91970897	+	HORVU7Hr1G037910	
chr7H	99963282	99965890	-	HORVU7Hr1G038820	
chr7H	102935178	102937837	+	HORVU7Hr1G039320	K03439
chr7H	102935489	102939889	-	HORVU7Hr1G039330	K03439
chr7H	103566716	103569555	-	HORVU7Hr1G039440	K03134
chr7H	103630064	103630999	-	HORVU7Hr1G039460	
chr7H	106400469	106420985	+	HORVU7Hr1G039850	
chr7H	109373213	109374520	-	HORVU7Hr1G040370	K08912
chr7H	109380119	109381636	-	HORVU7Hr1G040380	
chr7H	109382547	109389554	-	HORVU7Hr1G040390	
chr7H	120221128	120224149	+	HORVU7Hr1G042110	
chr7H	120221217	120225757	-	HORVU7Hr1G042120	K14638
chr7H	120352822	120355429	-	HORVU7Hr1G042130	K01512
chr7H	142388902	142396963	+	HORVU7Hr1G045240	
chr7H	143690820	143696411	+	HORVU7Hr1G045350	
chr7H	144284135	144291911	+	HORVU7Hr1G045550	
chr7H	144289836	144293353	-	HORVU7Hr1G045580	
chr7H	146582057	146582296	-	HORVU7Hr1G045750	
chr7H	146585894	146590335	+	HORVU7Hr1G045770	K16732
chr7H	149875814	149879925	+	HORVU7Hr1G046250	K01188
chr7H	149880190	149883832	-	HORVU7Hr1G046270	
chr7H	149883969	149885304	+	HORVU7Hr1G046280	
chr7H	150238886	150248647	+	HORVU7Hr1G046290	
chr7H	215393894	215397163	+	HORVU7Hr1G054190	
chr7H	233957547	233961150	+	HORVU7Hr1G056230	
chr7H	233961100	233963281	-	HORVU7Hr1G056240	K01802

chr7H	234654877	234669226	-	HORVU7Hr1G056410	
chr7H	234665154	234712484	+	HORVU7Hr1G056420	
chr7H	235074749	235077901	+	HORVU7Hr1G056450	
chr7H	235075668	235080845	-	HORVU7Hr1G056460	K15191
chr7H	235388068	235395343	-	HORVU7Hr1G056470	
chr7H	236127458	236134439	+	HORVU7Hr1G056490	
chr7H	237161371	237174117	+	HORVU7Hr1G056570	
chr7H	237647324	237651565	+	HORVU7Hr1G056590	K01641
chr7H	237946753	237949777	-	HORVU7Hr1G056600	
chr7H	238440698	238446807	+	HORVU7Hr1G056630	
chr7H	238655386	238656052	+	HORVU7Hr1G056650	
chr7H	238656122	238662065	-	HORVU7Hr1G056660	
chr7H	240513113	240531374	+	HORVU7Hr1G056770	
chr7H	240520656	240521269	+	HORVU7Hr1G056780	
chr7H	240628874	240631292	+	HORVU7Hr1G056790	
chr7H	242561602	242565943	+	HORVU7Hr1G056910	
chr7H	243535369	243549081	+	HORVU7Hr1G057010	
chr7H	247684936	247687293	-	HORVU7Hr1G057280	
chr7H	247694774	247695889	-	HORVU7Hr1G057290	K03301
chr7H	248143264	248146274	+	HORVU7Hr1G057310	K19045
chr7H	248580108	248581914	+	HORVU7Hr1G057330	
chr7H	248978165	248980455	-	HORVU7Hr1G057350	
chr7H	249987945	249993911	+	HORVU7Hr1G057390	
chr7H	250591388	250596270	+	HORVU7Hr1G057430	K00434
chr7H	251131641	251134363	-	HORVU7Hr1G057470	
chr7H	255291641	255295975	+	HORVU7Hr1G058060	
chr7H	255333368	255334106	+	HORVU7Hr1G058070	
chr7H	255335961	255342936	+	HORVU7Hr1G058080	
chr7H	255345089	255349794	+	HORVU7Hr1G058090	K18195
chr7H	258860422	258866854	-	HORVU7Hr1G058360	
chr7H	260597062	260597722	-	HORVU7Hr1G058720	
chr7H	260598567	260607607	-	HORVU7Hr1G058730	K08232
chr7H	260912236	260917644	-	HORVU7Hr1G058750	
chr7H	260916805	260921420	+	HORVU7Hr1G058760	K25307
chr7H	260920818	260922470	-	HORVU7Hr1G058770	
chr7H	261793143	261795118	+	HORVU7Hr1G058860	K01517
chr7H	261801075	261802127	+	HORVU7Hr1G058900	
chr7H	263804676	263807137	+	HORVU7Hr1G059090	K02865
chr7H	264045880	264047843	-	HORVU7Hr1G059190	
chr7H	264053847	264061164	-	HORVU7Hr1G059220	K10398
chr7H	264054465	264057015	+	HORVU7Hr1G059230	
chr7H	265435033	265435950	+	HORVU7Hr1G059290	K22255
chr7H	265667640	265670302	+	HORVU7Hr1G059320	K12733

chr7H	270642667	270647056	-	HORVU7Hr1G059850	K09646
chr7H	273940013	273953143	-	HORVU7Hr1G060130	
chr7H	275457995	275458504	+	HORVU7Hr1G060220	
chr7H	282128361	282138576	+	HORVU7Hr1G060790	
chr7H	284995960	285000564	-	HORVU7Hr1G061180	
chr7H	289708638	289720688	-	HORVU7Hr1G061640	
chr7H	300518692	300523227	-	HORVU7Hr1G062400	
chr7H	308189001	308194876	-	HORVU7Hr1G063150	K12822
chr7H	312097918	312104877	+	HORVU7Hr1G063510	
chr7H	314391517	314425666	+	HORVU7Hr1G063720	
chr7H	314399516	314400061	-	HORVU7Hr1G063750	
chr7H	314409375	314409915	+	HORVU7Hr1G063770	
chr7H	315905183	315918537	-	HORVU7Hr1G063970	K13126
chr7H	315920567	315922328	-	HORVU7Hr1G063980	K00392
chr7H	321635166	321636524	+	HORVU7Hr1G064860	
chr7H	333730513	333781184	-	HORVU7Hr1G066170	
chr7H	336285348	336309444	-	HORVU7Hr1G066300	K06636
chr7H	342078562	342095008	-	HORVU7Hr1G066590	
chr7H	343592862	343593277	+	HORVU7Hr1G066740	
chr7H	343596981	343600920	-	HORVU7Hr1G066750	
chr7H	346181113	346195444	+	HORVU7Hr1G066930	
chr7H	347129641	347132081	+	HORVU7Hr1G067060	K02873
chr7H	348270156	348270398	+	HORVU7Hr1G067100	K03358
chr7H	348272673	348276098	-	HORVU7Hr1G067110	
chr7H	350306493	350310404	+	HORVU7Hr1G067200	
chr7H	352048347	352050036	+	HORVU7Hr1G067520	K12581
chr7H	354110299	354144212	-	HORVU7Hr1G067620	
chr7H	354914497	354921990	-	HORVU7Hr1G067710	
chr7H	354925302	354925758	+	HORVU7Hr1G067730	
chr7H	357542424	357546443	-	HORVU7Hr1G067950	
chr7H	360255170	360279085	-	HORVU7Hr1G068320	
chr7H	361405465	361419175	-	HORVU7Hr1G068410	
chr7H	366099321	366227518	-	HORVU7Hr1G068990	
chr7H	367194699	367199111	+	HORVU7Hr1G069070	
chr7H	386642801	386646158	+	HORVU7Hr1G070970	
chr7H	400829251	400835819	-	HORVU7Hr1G072050	
chr7H	401049202	401096899	-	HORVU7Hr1G072120	
chr7H	402151029	402163440	-	HORVU7Hr1G072240	
chr7H	402159225	402159449	+	HORVU7Hr1G072300	
chr7H	444626348	444636100	-	HORVU7Hr1G076160	K22611
chr7H	450200080	450209793	+	HORVU7Hr1G077000	
chr7H	451171861	451177182	+	HORVU7Hr1G077110	
chr7H	621836594	621839030	+	HORVU7Hr1G107080	K12668

chr7H	621839414	621842700	-	HORVU7Hr1G107100	K07375
chr7H	623284314	623284435	-	ENSRNA049488666	
chr7H	623284859	623291176	-	HORVU7Hr1G107520	K12735
chr7H	623413985	623419666	+	HORVU7Hr1G107550	
chr7H	623647717	623653945	-	HORVU7Hr1G107700	
chr7H	623658288	623662004	-	HORVU7Hr1G107720	
chr7H	623669799	623670056	+	HORVU7Hr1G107730	
chr7H	623887563	623895540	+	HORVU7Hr1G107760	
chr7H	623890892	623896987	-	HORVU7Hr1G107770	K11593
chr7H	625794100	625809365	-	HORVU7Hr1G108600	
chr7H	626156440	626200880	+	HORVU7Hr1G108730	
chr7H	626291758	626294967	+	HORVU7Hr1G108780	K14563
chr7H	626293156	626293230	+	ENSRNA049488662	
chr7H	626293486	626293560	+	ENSRNA049488663	
chr7H	626418473	626424722	-	HORVU7Hr1G108890	
chr7H	627296198	627297570	+	HORVU7Hr1G109170	K22683
chr7H	627298807	627305366	+	HORVU7Hr1G109180	
chr7H	627303101	627307263	-	HORVU7Hr1G109190	
chr7H	627313778	627314831	-	HORVU7Hr1G109200	
chr7H	627315504	627317238	-	HORVU7Hr1G109210	
chr7H	627501280	627503618	+	HORVU7Hr1G109280	
chr7H	627501855	627509102	-	HORVU7Hr1G109290	
chr7H	628034411	628038083	+	HORVU7Hr1G109420	K05756
chr7H	628247557	628248157	-	HORVU7Hr1G109540	
chr7H	628254382	628257576	+	HORVU7Hr1G109550	
chr7H	628259528	628263040	+	HORVU7Hr1G109560	K21813
chr7H	628360508	628360580	+	ENSRNA049440972	
chr7H	628363979	628364051	+	ENSRNA049440961	
chr7H	628364391	628364463	-	ENSRNA049441783	
chr7H	628364665	628369128	-	HORVU7Hr1G109580	
chr7H	628369567	628374492	-	HORVU7Hr1G109590	
chr7H	628801065	628813596	-	HORVU7Hr1G109770	
chr7H	629619602	629624174	-	HORVU7Hr1G110260	
chr7H	629640247	629641500	+	HORVU7Hr1G110280	K11252
chr7H	629640922	629641332	-	HORVU7Hr1G110290	K11252
chr7H	629652507	629653312	-	HORVU7Hr1G110310	K11252
chr7H	629662818	629664093	+	HORVU7Hr1G110320	K11252
chr7H	629663547	629664043	-	HORVU7Hr1G110330	K11252
chr7H	629950371	629954881	-	HORVU7Hr1G110440	
chr7H	632201936	632202007	+	ENSRNA049440927	
chr7H	632202536	632208108	-	HORVU7Hr1G110990	
chr7H	636161492	636165359	-	HORVU7Hr1G112920	
chr7H	636161495	636163096	+	HORVU7Hr1G112930	K02377

chr7H	636164621	636165448	+	HORVU7Hr1G112940	
chr4H	17438157	17446158	+	HORVU4Hr1G007020	
chr4H	23461411	23468904	+	HORVU4Hr1G008530	
chr4H	26338990	26341159	-	HORVU4Hr1G009270	
chr4H	26664812	26669252	-	HORVU4Hr1G009360	K18328
chr4H	28425448	28427524	+	HORVU4Hr1G009760	
chr4H	28932392	28936565	+	HORVU4Hr1G009870	
chr4H	28937626	28939374	-	HORVU4Hr1G009880	
chr4H	29194024	29198432	-	HORVU4Hr1G009970	
chr4H	29424896	29431208	-	HORVU4Hr1G009990	
chr4H	30747069	30748133	+	HORVU4Hr1G010290	
chr4H	30750565	30755045	+	HORVU4Hr1G010300	
chr4H	35007639	35013513	+	HORVU4Hr1G011370	
chr4H	35087022	35091459	+	HORVU4Hr1G011420	
chr4H	41102174	41113394	+	HORVU4Hr1G012400	
chr4H	43582368	43584005	+	HORVU4Hr1G012850	K04505
chr4H	43592359	43592818	-	HORVU4Hr1G012870	K02144
chr4H	44238559	44247903	+	HORVU4Hr1G013030	
chr4H	46767208	46774429	+	HORVU4Hr1G013420	
chr4H	65649263	65650382	+	HORVU4Hr1G016210	
chr4H	66861420	66864859	-	HORVU4Hr1G016350	
chr4H	68845951	68847192	-	HORVU4Hr1G016600	
chr4H	69379505	69383521	-	HORVU4Hr1G016640	K14007
chr4H	72684241	72689839	+	HORVU4Hr1G017030	K14190
chr4H	81552157	81566437	+	HORVU4Hr1G018150	
chr4H	85417562	85418639	-	HORVU4Hr1G018650	
chr4H	92750153	92757367	+	HORVU4Hr1G019360	K10644
chr4H	92763298	92766044	-	HORVU4Hr1G019380	
chr4H	93624533	93627841	+	HORVU4Hr1G019460	
chr4H	93635488	93644440	+	HORVU4Hr1G019470	
chr4H	94469497	94474320	-	HORVU4Hr1G019530	
chr4H	94599322	94602229	+	HORVU4Hr1G019560	K13140
chr4H	94602390	94605682	+	HORVU4Hr1G019570	K01623
chr4H	95579515	95581578	-	HORVU4Hr1G019850	K21844
chr4H	96358327	96366275	+	HORVU4Hr1G019910	
chr4H	96369114	96371272	-	HORVU4Hr1G019930	K13946
chr4H	96770575	96773221	+	HORVU4Hr1G019980	K02925
chr4H	96774405	96780109	+	HORVU4Hr1G019990	
chr4H	96780385	96784552	+	HORVU4Hr1G020000	
chr4H	97456114	97457293	-	HORVU4Hr1G020030	
chr4H	98873364	98878574	+	HORVU4Hr1G020160	
chr4H	107198462	107206973	+	HORVU4Hr1G021140	
chr4H	123782482	123797229	+	HORVU4Hr1G023100	K11717

chr4H	145111427	145111564	+	HORVU4Hr1G025060	
chr4H	145113474	145117222	+	HORVU4Hr1G025070	K13430
chr4H	145113474	145123037	-	HORVU4Hr1G025080	K13430
chr4H	165585311	165588617	-	HORVU4Hr1G027180	
chr4H	348274482	348277389	+	HORVU4Hr1G043660	K02884
chr4H	348342112	348344688	-	HORVU4Hr1G043680	K24917
chr4H	350262817	350266616	+	HORVU4Hr1G043910	K09580
chr4H	351970606	351985432	+	HORVU4Hr1G043970	K00876
chr4H	387764460	387765655	-	HORVU4Hr1G048160	
chr4H	402185706	402208358	+	HORVU4Hr1G049590	
chr4H	413874464	413887775	-	HORVU4Hr1G050710	
chr4H	437127502	437137117	-	HORVU4Hr1G052570	
chr4H	437167861	437169180	-	HORVU4Hr1G052610	K22074
chr4H	438608884	438609405	+	HORVU4Hr1G052770	
chr4H	438612259	438619827	+	HORVU4Hr1G052790	
chr4H	438669562	438673914	-	HORVU4Hr1G052810	K01756
chr4H	438675397	438675606	+	HORVU4Hr1G052820	
chr4H	476598333	476598935	+	HORVU4Hr1G056750	
chr4H	476599485	476600818	+	HORVU4Hr1G056760	
chr4H	480892997	480895942	-	HORVU4Hr1G057200	K20536
chr4H	482105453	482113450	-	HORVU4Hr1G057360	
chr4H	504382270	504387591	-	HORVU4Hr1G060190	
chr4H	574701706	574708389	-	HORVU4Hr1G070220	
chr4H	580092085	580097073	+	HORVU4Hr1G071250	K17469
chr4H	580160224	580171376	-	HORVU4Hr1G071270	K14521
chr4H	583649413	583655355	+	HORVU4Hr1G072150	K01904
chr4H	586897060	586903117	+	HORVU4Hr1G072920	
chr4H	586899433	586902977	-	HORVU4Hr1G072930	
chr4H	587500662	587501437	+	HORVU4Hr1G072960	K15397
chr4H	587511230	587512083	-	HORVU4Hr1G072970	
chr4H	595099439	595100488	+	HORVU4Hr1G074690	
chr4H	595103615	595109265	-	HORVU4Hr1G074700	K04382
chr4H	595523761	595528850	+	HORVU4Hr1G074790	
chr4H	596060773	596066666	-	HORVU4Hr1G074960	
chr4H	596060795	596064280	+	HORVU4Hr1G074970	
chr4H	596299561	596307590	+	HORVU4Hr1G075040	
chr4H	597090974	597096520	+	HORVU4Hr1G075200	K15378
chr4H	599032193	599035023	-	HORVU4Hr1G075710	K02930
chr4H	599159830	599166651	-	HORVU4Hr1G075720	K20843
chr4H	601100279	601108113	+	HORVU4Hr1G076570	K00454
chr4H	605486888	605488575	+	HORVU4Hr1G077790	K13065
chr4H	605492266	605497222	+	HORVU4Hr1G077800	
chr4H	606225436	606234907	+	HORVU4Hr1G078050	

chr4H	609867536	609872982	-	HORVU4Hr1G078920	
chr4H	611031626	611040666	+	HORVU4Hr1G079100	
chr4H	611470870	611473982	+	HORVU4Hr1G079170	
chr4H	611470881	611475710	-	HORVU4Hr1G079180	
chr4H	611627598	611633156	-	HORVU4Hr1G079250	
chr4H	611679942	611682960	-	HORVU4Hr1G079260	
chr4H	611690785	611692599	-	HORVU4Hr1G079270	
chr4H	611794086	611804953	-	HORVU4Hr1G079300	
chr4H	611811711	611814381	+	HORVU4Hr1G079310	
chr4H	611815171	611818868	+	HORVU4Hr1G079330	K13071
chr4H	612079781	612082649	+	HORVU4Hr1G079400	
chr4H	612083626	612108450	+	HORVU4Hr1G079420	
chr4H	613198175	613209115	+	HORVU4Hr1G079710	
chr4H	613205845	613208339	-	HORVU4Hr1G079740	
chr4H	613751731	613756976	+	HORVU4Hr1G079940	K02068
chr4H	613759966	613781275	-	HORVU4Hr1G079950	
chr4H	613789679	613791820	+	HORVU4Hr1G079990	
chr4H	613793336	613797119	-	HORVU4Hr1G080000	K01945
chr4H	613805852	613807701	-	HORVU4Hr1G080030	
chr4H	613808586	613814590	-	HORVU4Hr1G080050	
chr4H	615383587	615388493	+	HORVU4Hr1G080420	
chr4H	615697912	615705266	-	HORVU4Hr1G080450	
chr4H	616240827	616250618	+	HORVU4Hr1G080560	
chr4H	616241933	616249757	-	HORVU4Hr1G080570	K01102
chr4H	617388840	617391479	+	HORVU4Hr1G080780	K08242
chr4H	617393608	617393889	+	HORVU4Hr1G080790	
chr4H	617794026	617809180	+	HORVU4Hr1G080940	
chr4H	618414859	618416109	+	HORVU4Hr1G081100	K00799
chr4H	618692052	618693972	-	HORVU4Hr1G081230	K11279
chr4H	618869082	618875298	-	HORVU4Hr1G081310	
chr4H	622330494	622332849	-	HORVU4Hr1G082470	K23618
chr4H	624578081	624580802	+	HORVU4Hr1G083200	
chr4H	624583900	624589934	-	HORVU4Hr1G083210	
chr4H	625193355	625194764	+	HORVU4Hr1G083400	
chr4H	626997423	627002978	+	HORVU4Hr1G083950	K16296
chr4H	627011903	627020858	-	HORVU4Hr1G083970	
chr4H	629483972	629487192	+	HORVU4Hr1G084600	
chr4H	629524229	629528665	+	HORVU4Hr1G084670	
chr4H	629524254	629534446	-	HORVU4Hr1G084680	
chr4H	629589221	629593649	-	HORVU4Hr1G084710	
chr4H	629644007	629644737	+	HORVU4Hr1G084750	
chr4H	629644892	629647909	-	HORVU4Hr1G084760	K10577
chr4H	630855665	630860598	+	HORVU4Hr1G085050	K22390

chr4H	630862306	630862811	+	HORVU4Hr1G085060	
chr4H	630899295	630906064	+	HORVU4Hr1G085100	
chr4H	631020732	631022177	-	HORVU4Hr1G085150	
chr4H	631305594	631307831	-	HORVU4Hr1G085250	K09873
chr4H	631469114	631473133	-	HORVU4Hr1G085280	
chr4H	631473499	631479506	-	HORVU4Hr1G085290	
chr4H	631485665	631488175	+	HORVU4Hr1G085300	
chr4H	631485732	631490073	-	HORVU4Hr1G085310	
chr4H	632308238	632310222	+	HORVU4Hr1G085730	
chr4H	632310354	632313768	-	HORVU4Hr1G085740	K19323
chr4H	632316301	632325141	-	HORVU4Hr1G085750	
chr4H	633160208	633161206	+	HORVU4Hr1G085990	
chr4H	633169005	633174352	+	HORVU4Hr1G086000	
chr4H	633178339	633181842	+	HORVU4Hr1G086010	K03036
chr4H	633181302	633184491	-	HORVU4Hr1G086020	
chr4H	633598304	633602296	+	HORVU4Hr1G086300	K01193
chr4H	633831715	633835334	+	HORVU4Hr1G086320	K01074
chr4H	633837715	633843075	+	HORVU4Hr1G086330	K03217
chr4H	633848205	633851299	+	HORVU4Hr1G086340	
chr4H	634679688	634693548	+	HORVU4Hr1G086600	
chr4H	634681305	634684145	-	HORVU4Hr1G086610	K10523
chr4H	634691102	634691644	-	HORVU4Hr1G086620	
chr4H	634768494	634773415	+	HORVU4Hr1G086640	
chr4H	634776721	634782324	+	HORVU4Hr1G086650	
chr4H	634782750	634783854	-	HORVU4Hr1G086660	
chr4H	634903821	634904932	+	HORVU4Hr1G086700	
chr4H	634958591	634967450	-	HORVU4Hr1G086770	
chr4H	635064480	635066507	+	HORVU4Hr1G086790	K11096
chr4H	636203381	636206530	-	HORVU4Hr1G087110	K13356
chr4H	636210530	636214016	-	HORVU4Hr1G087120	K04506
chr4H	636346638	636348300	+	HORVU4Hr1G087220	
chr4H	636352038	636357622	-	HORVU4Hr1G087230	K14641
chr4H	636577557	636581046	-	HORVU4Hr1G087350	K08994
chr4H	636582909	636595402	-	HORVU4Hr1G087360	
chr4H	637460167	637467469	-	HORVU4Hr1G087580	
chr4H	637477795	637503164	+	HORVU4Hr1G087590	
chr4H	641252933	641254865	-	HORVU4Hr1G088890	
chr4H	641255864	641265633	-	HORVU4Hr1G088900	K01872
chr4H	641271439	641285258	+	HORVU4Hr1G088910	
chr4H	641293324	641295356	-	HORVU4Hr1G088930	
chr4H	641590588	641595822	+	HORVU4Hr1G089060	K00103
chr4H	641600081	641606961	-	HORVU4Hr1G089080	
chr4H	641608344	641613015	+	HORVU4Hr1G089090	K04043

chr4H	642064582	642066848	+	HORVU4Hr1G089270	
chr4H	642064583	642071249	-	HORVU4Hr1G089280	
chr4H	642261253	642262994	+	HORVU4Hr1G089410	
chr4H	643004384	643006737	-	HORVU4Hr1G089560	
chr4H	643012334	643013799	+	HORVU4Hr1G089580	K22312
chr4H	643053070	643099588	-	HORVU4Hr1G089590	
chr4H	643065106	643069109	-	HORVU4Hr1G089610	K13508
chr4H	644829984	644832691	-	HORVU4Hr1G090260	
chr4H	644831527	644837545	+	HORVU4Hr1G090270	
chr4H	644838102	644840787	+	HORVU4Hr1G090280	K03115
chr4H	644976930	644980105	-	HORVU4Hr1G090330	
chr4H	644985085	644988633	+	HORVU4Hr1G090350	K18065
chr4H	644989198	644992163	-	HORVU4Hr1G090360	K20889
chr6H	11919134	11925471	+	HORVU6Hr1G005350	
chr6H	11922767	12061749	-	HORVU6Hr1G005360	
chr6H	11928429	11944450	-	HORVU6Hr1G005370	K12821
chr6H	43926918	43928295	+	HORVU6Hr1G017960	
chr6H	43928354	43928550	-	ENSRNA049485813	
chr6H	43928474	43928672	+	HORVU6Hr1G017970	
chr6H	143256473	143261264	+	HORVU6Hr1G032570	K03064
chr6H	355516468	355521532	+	HORVU6Hr1G055820	
chr6H	364354426	364362218	+	HORVU6Hr1G056490	
chr6H	405010351	405014188	-	HORVU6Hr1G060760	
chr6H	413265108	413265651	-	HORVU6Hr1G061740	
chr6H	413274272	413281744	-	HORVU6Hr1G061750	
chr6H	424348311	424353175	+	HORVU6Hr1G063100	K10268
chr6H	424353691	424356849	+	HORVU6Hr1G063120	
chr6H	442928653	442931507	-	HORVU6Hr1G064820	
chr6H	489027667	489029492	+	HORVU6Hr1G070350	
chr6H	500725043	500735879	+	HORVU6Hr1G072070	
chr6H	500730262	500730788	-	HORVU6Hr1G072080	
chr6H	500732208	500735891	-	HORVU6Hr1G072100	K15445
chr6H	502525838	502530698	+	HORVU6Hr1G072300	
chr6H	502529735	502530403	-	HORVU6Hr1G072310	
chr6H	503268669	503269507	-	HORVU6Hr1G072390	
chr6H	503270970	503276564	-	HORVU6Hr1G072420	
chr6H	509623775	509629249	+	HORVU6Hr1G073590	
chr6H	513108626	513115648	+	HORVU6Hr1G074210	
chr6H	513108807	513120207	-	HORVU6Hr1G074220	
chr6H	530729201	530732139	+	HORVU6Hr1G077500	K18810
chr6H	538081936	538088851	+	HORVU6Hr1G079560	
chr6H	538093475	538094848	+	HORVU6Hr1G079590	
chr6H	559749653	559758664	-	HORVU6Hr1G085710	

chr6H	560155444	560160792	+	HORVU6Hr1G085870	K17086
chr6H	560158863	560183696	-	HORVU6Hr1G085880	
chr6H	575766021	575769296	+	HORVU6Hr1G091540	
chr6H	575769878	575774590	-	HORVU6Hr1G091550	K06269
chr1H	23198930	23202488	-	HORVU1Hr1G010220	
chr1H	67288427	67289119	-	HORVU1Hr1G018370	
chr1H	67297058	67303566	-	HORVU1Hr1G018380	K07466
chr1H	80825648	80825788	-	HORVU1Hr1G020460	
chr1H	80827355	80832088	+	HORVU1Hr1G020470	K12449
chr1H	81825284	81832874	-	HORVU1Hr1G020620	K11374
chr1H	82141298	82143805	-	HORVU1Hr1G020690	K00430
chr1H	84326638	84338957	+	HORVU1Hr1G021120	
chr1H	84343681	84347935	+	HORVU1Hr1G021130	K01893
chr1H	84399076	84400190	+	HORVU1Hr1G021150	K00799
chr1H	84405715	84407984	+	HORVU1Hr1G021160	K00799
chr1H	84414501	84415817	+	HORVU1Hr1G021170	K00799
chr1H	284571341	284583896	+	HORVU1Hr1G040250	
chr1H	284574939	284575781	-	HORVU1Hr1G040260	
chr1H	284579816	284580490	-	HORVU1Hr1G040300	
chr1H	284580560	284580919	-	HORVU1Hr1G040310	
chr1H	321614760	321618227	+	HORVU1Hr1G044040	
chr1H	321616679	321617299	+	HORVU1Hr1G044050	
chr1H	322787504	322790215	+	HORVU1Hr1G044380	
chr1H	322796701	322797001	+	HORVU1Hr1G044400	
chr1H	323431234	323433765	-	HORVU1Hr1G044530	
chr1H	328624334	328628558	-	HORVU1Hr1G045220	K20858
chr1H	341763204	341772442	-	HORVU1Hr1G046630	K01873
chr1H	342779014	342781162	-	HORVU1Hr1G046710	
chr1H	344583893	344588775	-	HORVU1Hr1G046870	K11093
chr1H	348548115	348552175	+	HORVU1Hr1G047220	K09755
chr1H	361583031	361592163	+	HORVU1Hr1G048720	
chr1H	362142404	362144861	-	HORVU1Hr1G048830	K03327
chr1H	362147439	362152283	-	HORVU1Hr1G048840	
chr1H	365007068	365008396	-	HORVU1Hr1G049260	
chr1H	365007166	365010002	+	HORVU1Hr1G049270	K00799
chr1H	366507315	366510797	-	HORVU1Hr1G049480	
chr1H	367819284	367820502	-	HORVU1Hr1G049650	
chr1H	367825851	367827802	-	HORVU1Hr1G049660	
chr1H	368818215	368824801	+	HORVU1Hr1G049810	
chr1H	368818336	368824678	-	HORVU1Hr1G049820	
chr1H	375899671	375903804	+	HORVU1Hr1G050640	
chr1H	376369608	376374160	+	HORVU1Hr1G050730	
chr1H	376375852	376380580	+	HORVU1Hr1G050740	

chr1H	376375968	376376787	-	HORVU1Hr1G050750	
chr1H	376378591	376383176	-	HORVU1Hr1G050760	K00913
chr1H	381313035	381328695	+	HORVU1Hr1G051460	
chr1H	381322517	381328711	-	HORVU1Hr1G051470	
chr1H	382299770	382300802	-	HORVU1Hr1G051560	
chr1H	382301293	382317173	-	HORVU1Hr1G051570	K17973
chr1H	383460565	383462205	-	HORVU1Hr1G051700	
chr1H	384608005	384616626	+	HORVU1Hr1G051840	
chr1H	388627045	388643679	+	HORVU1Hr1G052430	
chr1H	391997066	392011255	+	HORVU1Hr1G053020	K23288
chr1H	392012662	392013871	-	HORVU1Hr1G053050	
chr1H	393441626	393460600	-	HORVU1Hr1G053190	
chr1H	393963164	393967064	-	HORVU1Hr1G053270	
chr1H	393971002	393974978	-	HORVU1Hr1G053280	
chr1H	395873135	395875994	+	HORVU1Hr1G053510	
chr1H	397164492	397171992	+	HORVU1Hr1G053840	
chr1H	397858671	397861712	+	HORVU1Hr1G053900	
chr1H	397861757	397861827	-	ENSRNA049447675	
chr1H	398652421	398808254	-	HORVU1Hr1G053990	
chr1H	398749497	398753454	+	HORVU1Hr1G054020	
chr1H	399234596	399237714	-	HORVU1Hr1G054060	
chr1H	401040803	401045363	-	HORVU1Hr1G054240	
chr1H	401397085	401413879	-	HORVU1Hr1G054260	
chr1H	404207619	404209620	-	HORVU1Hr1G054930	K19042
chr1H	404914155	404917858	-	HORVU1Hr1G055210	K22684
chr1H	405296725	405301886	+	HORVU1Hr1G055300	K06268
chr1H	405305563	405312023	-	HORVU1Hr1G055320	
chr1H	405305563	405312023	+	HORVU1Hr1G055330	
chr1H	406525155	406531073	+	HORVU1Hr1G055510	
chr1H	406533021	406535450	+	HORVU1Hr1G055520	
chr1H	406621879	406625635	+	HORVU1Hr1G055530	
chr1H	406997955	407009508	+	HORVU1Hr1G055580	
chr1H	407342662	407538866	-	HORVU1Hr1G055600	
chr1H	407348346	407349410	+	HORVU1Hr1G055630	
chr1H	409504534	409511833	+	HORVU1Hr1G055890	
chr1H	419378870	419385796	-	HORVU1Hr1G057460	
chr1H	419390597	419391353	+	HORVU1Hr1G057480	
chr1H	419781402	419786824	-	HORVU1Hr1G057520	
chr1H	420229299	420233386	-	HORVU1Hr1G057570	K03955
chr1H	420569969	420574287	+	HORVU1Hr1G057640	
chr1H	420569993	420574422	-	HORVU1Hr1G057650	
chr1H	420574729	420581630	-	HORVU1Hr1G057660	K14411
chr1H	423112771	423120255	+	HORVU1Hr1G058000	

chr1H	423122710	423123436	-	HORVU1Hr1G058010	
chr1H	423126092	423131749	+	HORVU1Hr1G058020	K13207
chr1H	424467284	424470411	+	HORVU1Hr1G058170	
chr1H	424823399	424830206	-	HORVU1Hr1G058190	
chr1H	425801911	425807445	-	HORVU1Hr1G058330	
chr1H	425921313	425925269	-	HORVU1Hr1G058340	
chr1H	428065978	428068607	+	HORVU1Hr1G058750	
chr1H	428079112	428085155	-	HORVU1Hr1G058770	K18482
chr1H	458912977	458916662	-	HORVU1Hr1G064160	
chr1H	458915520	458915711	-	HORVU1Hr1G064180	
chr1H	461398914	461403858	+	HORVU1Hr1G064440	K08472
chr1H	464420461	464423230	-	HORVU1Hr1G064950	
chr1H	465323892	465328316	-	HORVU1Hr1G065000	K22733
chr1H	469073016	469075988	+	HORVU1Hr1G065820	
chr1H	471472632	471476335	-	HORVU1Hr1G066240	K03809
chr1H	472946083	472947141	+	HORVU1Hr1G066500	K02116
chr1H	472948820	472951168	-	HORVU1Hr1G066510	
chr1H	507686919	507690316	-	HORVU1Hr1G074350	K10355
chr1H	508567384	508569768	-	HORVU1Hr1G074600	
chr1H	508779949	508787438	-	HORVU1Hr1G074660	
chr1H	508789844	508794190	-	HORVU1Hr1G074670	
chr1H	509508851	509513968	+	HORVU1Hr1G074940	
chr1H	509515883	509527854	-	HORVU1Hr1G074960	
chr1H	509763075	509768157	-	HORVU1Hr1G075110	
chr1H	509776082	509776153	+	ENSRNA049448274	
chr1H	514008170	514016820	+	HORVU1Hr1G076380	
chr1H	514922336	514929397	+	HORVU1Hr1G076610	
chr1H	514925678	514933568	-	HORVU1Hr1G076620	
chr1H	518159277	518160365	-	HORVU1Hr1G077680	K14516
chr1H	520109884	520111240	+	HORVU1Hr1G078380	K08912
chr1H	520118880	520122133	+	HORVU1Hr1G078390	K09775
chr1H	521271209	521271499	-	HORVU1Hr1G078870	
chr1H	521283934	521292837	+	HORVU1Hr1G078880	
chr1H	521293226	521300129	-	HORVU1Hr1G078890	
chr1H	522260222	522266530	-	HORVU1Hr1G079050	K12393
chr1H	522260258	522261172	+	HORVU1Hr1G079060	
chr1H	522473999	522486419	-	HORVU1Hr1G079170	
chr1H	522486714	522492991	-	HORVU1Hr1G079200	
chr1H	522495889	522497697	+	HORVU1Hr1G079210	
chr1H	523730064	523732132	-	HORVU1Hr1G079730	
chr1H	524503284	524509889	+	HORVU1Hr1G080070	K01952
chr1H	530744050	530752626	+	HORVU1Hr1G082900	K18693
chr1H	531647372	531648679	+	HORVU1Hr1G083340	

chr1H	540571001	540572989	-	HORVU1Hr1G087870	
chr1H	540581949	540584326	-	HORVU1Hr1G087880	
chr1H	540584485	540593837	-	HORVU1Hr1G087900	
chr1H	541346878	541351320	+	HORVU1Hr1G088270	
chr1H	541902822	541907742	-	HORVU1Hr1G088510	
chrUn	7484963	7494019	-	HORVU0Hr1G001400	K24763
chrUn	27394947	27395845	-	HORVU0Hr1G004750	
chrUn	30807952	30809673	+	HORVU0Hr1G005640	
chrUn	54922165	54922650	+	HORVU0Hr1G009370	
chrUn	54927981	54933076	-	HORVU0Hr1G009380	
chrUn	64097381	64101477	-	HORVU0Hr1G011310	
chrUn	64109105	64109510	+	HORVU0Hr1G011320	
chrUn	64109877	64110408	+	HORVU0Hr1G011330	
chrUn	104228480	104230005	+	HORVU0Hr1G019800	
chrUn	110178333	110179149	+	HORVU0Hr1G021040	
chrUn	110179208	110183094	-	HORVU0Hr1G021050	
chrUn	110183556	110187206	-	HORVU0Hr1G021060	
chrUn	247086982	247091166	-	HORVU0Hr1G038600	
chrUn	247095871	247103683	-	HORVU0Hr1G038620	K10885
chrUn	247095871	247099519	+	HORVU0Hr1G038610	
chrUn	247105278	247106340	-	HORVU0Hr1G038630	
chrUn	248023923	248025701	+	HORVU0Hr1G039050	
chrUn	248032532	248035876	+	HORVU0Hr1G039070	
chrUn	248570417	248597651	-	HORVU0Hr1G039330	
chrUn	248576468	248576737	-	HORVU0Hr1G039350	

Table S13. All accessions used in this study.

Populations	NCBI ID	Accession ID	Sequencing type
Wb-NE	Hsp	WGS-Hsp1	resequencing
Wb-NE	SAMN08005653	WGS-Hsp2	resequencing
Wb-NE	SAMN08005654	WGS-Hsp3	resequencing
Wb-NE	SAMN08005655	WGS-Htr	resequencing
Wb-NE	SAMN20065695	HS7	RNA-resequencing
Wb-NE	SAMN20065696	HS26	RNA-resequencing
Wb-NE	SAMN20065697	HS38	RNA-resequencing
Wb-NE	SAMN20065698	HS56	RNA-resequencing
Wb-NE	SAMN20065699	HS18	RNA-resequencing
Wb-NE	SAMN20065700	HS23	RNA-resequencing
Wb-NE	SAMN20065701	HS30	RNA-resequencing

Wb-NE	SAMN20065702	HS31	RNA-resequencing
Wb-NE	SAMN20065703	HS39	RNA-resequencing
Cb-NE	SAMN08005706	WGS-Ld1	resequencing
Cb-NE	SAMN08005707	WGS-Ld2	resequencing
Cb-NE	SAMN08005659	WGS-Cu8	resequencing
Cb-NE	SAMN08005660	WGS-Cu9	resequencing
Cb-NE	SAMN08005662	WGS-Cu11	resequencing
Cb-NE	SAMN08005670	WGS-Cu19	resequencing
Wb-T	SAMN08005724	WGS-Tw10	resequencing
Wb-T	SAMN08005722	WGS-Tw8	resequencing
Wb-T	SAMN08005723	WGS-Tw9	resequencing
Wb-T	SAMN08005715	WGS-Tw1	resequencing
Wb-T	SAMN08005716	WGS-Tw2	resequencing
Wb-T	SAMN08005717	WGS-Tw3	resequencing
Wb-T	SAMN08005719	WGS-Tw5	resequencing
Wb-T	SAMN08005720	WGS-Tw6	resequencing
Wb-T	SAMN08005718	WGS-Tw4	resequencing
Wb-T	SAMN08005721	WGS-Tw7	resequencing
Wb-T	SAMN20065685	HS100	RNA-resequencing
Wb-T	SAMN20065686	HS109	RNA-resequencing
Wb-T	SAMN20065687	HS105	RNA-resequencing
Wb-T	SAMN20065688	HS106	RNA-resequencing
Wb-T	SAMN20065689	HS107	RNA-resequencing
Wb-T	SAMN20065690	HS108	RNA-resequencing
Wb-T	SAMN20065691	HS101	RNA-resequencing
Wb-T	SAMN20065692	HS102	RNA-resequencing
Wb-T	SAMN20065693	HS103	RNA-resequencing
Wb-T	SAMN20065694	HS104	RNA-resequencing
Cb-C	SAMN08005668	WGS-Cu17	resequencing
Cb-C	SAMN08005676	WGS-Cu25	resequencing
Cb-C	SAMN08005687	WGS-Cu36	resequencing
Cb-C	SAMN08005688	WGS-Cu37	resequencing
Cb-C	SAMN08005690	WGS-Cu39	resequencing
Cb-C	SAMN08005692	WGS-Cu41	resequencing
Cb-C	SAMN08005696	WGS-Cu45	resequencing
Cb-C	SAMN08005697	WGS-Cu46	resequencing

Cb-C	SAMN08005698	WGS-Cu47	resequencing
Cb-C	SAMN08005699	WGS-Cu48	resequencing
Cb-C	SAMN08005761	WGS-Qk37	resequencing
Cb-C	SAMN08005708	WGS-Ld3	resequencing
Cb-C	SAMN08005709	WGS-Ld4	resequencing
Cb-C	SAMN08005710	WGS-Ld5	resequencing
Cb-C	SAMN08005711	WGS-Ld6	resequencing
Cb-C	SAMN08005712	WGS-Ld7	resequencing
Cb-C	SAMN08005713	WGS-Ld8	resequencing
Cb-C	SAMN08005714	WGS-Ld9	resequencing
Cb-C	SAMN08005691	WGS-Cu40	resequencing
Cb-C	SAMN08005693	WGS-Cu42	resequencing
Cb-C	SAMN08005694	WGS-Cu43	resequencing
Cb-C	SAMN08005695	WGS-Cu44	resequencing
Cb-C	SAMN08005701	WGS-Cu50	resequencing
Cb-C	SAMN08005702	WGS-Cu51	resequencing
Cb-C	SAMN08005703	WGS-Cu52	resequencing
Cb-C	SAMN08005704	WGS-Cu53	resequencing
Cb-C	SAMN08005705	WGS-Cu54	resequencing
Cb-C	SAMN08005733	WGS-Qk9	resequencing
Cb-C	SAMN08005823	WGS-Qk99	resequencing
Cb-C	SAMN08005734	WGS-Qk10	resequencing
Cb-C	SAMN08005735	WGS-Qk11	resequencing
Cb-C	SAMN08005736	WGS-Qk12	resequencing
Cb-C	SAMN08005737	WGS-Qk13	resequencing
Cb-C	SAMN08005738	WGS-Qk14	resequencing
Cb-C	SAMN08005739	WGS-Qk15	resequencing
Cb-C	SAMN08005740	WGS-Qk16	resequencing
Cb-C	SAMN08005741	WGS-Qk17	resequencing
Cb-C	SAMN08005742	WGS-Qk18	resequencing
Cb-C	SAMN08005725	WGS-Qk1	resequencing
Cb-C	SAMN08005743	WGS-Qk19	resequencing
Cb-C	SAMN08005744	WGS-Qk20	resequencing
Cb-C	SAMN08005745	WGS-Qk21	resequencing
Cb-C	SAMN08005746	WGS-Qk22	resequencing
Cb-C	SAMN08005747	WGS-Qk23	resequencing

Cb-C	SAMN08005748	WGS-Qk24	resequencing
Cb-C	SAMN08005749	WGS-Qk25	resequencing
Cb-C	SAMN08005750	WGS-Qk26	resequencing
Cb-C	SAMN08005752	WGS-Qk28	resequencing
Cb-C	SAMN08005726	WGS-Qk2	resequencing
Cb-C	SAMN08005753	WGS-Qk29	resequencing
Cb-C	SAMN08005754	WGS-Qk30	resequencing
Cb-C	SAMN08005755	WGS-Qk31	resequencing
Cb-C	SAMN08005756	WGS-Qk32	resequencing
Cb-C	SAMN08005757	WGS-Qk33	resequencing
Cb-C	SAMN08005758	WGS-Qk34	resequencing
Cb-C	SAMN08005759	WGS-Qk35	resequencing
Cb-C	SAMN08005760	WGS-Qk36	resequencing
Cb-C	SAMN08005762	WGS-Qk38	resequencing
Cb-C	SAMN08005727	WGS-Qk3	resequencing
Cb-C	SAMN08005763	WGS-Qk39	resequencing
Cb-C	SAMN08005764	WGS-Qk40	resequencing
Cb-C	SAMN08005765	WGS-Qk41	resequencing
Cb-C	SAMN08005766	WGS-Qk42	resequencing
Cb-C	SAMN08005767	WGS-Qk43	resequencing
Cb-C	SAMN08005768	WGS-Qk44	resequencing
Cb-C	SAMN08005769	WGS-Qk45	resequencing
Cb-C	SAMN08005770	WGS-Qk46	resequencing
Cb-C	SAMN08005771	WGS-Qk47	resequencing
Cb-C	SAMN08005772	WGS-Qk48	resequencing
Cb-C	SAMN08005728	WGS-Qk4	resequencing
Cb-C	SAMN08005773	WGS-Qk49	resequencing
Cb-C	SAMN08005774	WGS-Qk50	resequencing
Cb-C	SAMN08005775	WGS-Qk51	resequencing
Cb-C	SAMN08005776	WGS-Qk52	resequencing
Cb-C	SAMN08005777	WGS-Qk53	resequencing
Cb-C	SAMN08005779	WGS-Qk55	resequencing
Cb-C	SAMN08005780	WGS-Qk56	resequencing
Cb-C	SAMN08005781	WGS-Qk57	resequencing
Cb-C	SAMN08005782	WGS-Qk58	resequencing
Cb-C	SAMN08005729	WGS-Qk5	resequencing

Cb-C	SAMN08005783	WGS-Qk59	resequencing
Cb-C	SAMN08005784	WGS-Qk60	resequencing
Cb-C	SAMN08005785	WGS-Qk61	resequencing
Cb-C	SAMN08005786	WGS-Qk62	resequencing
Cb-C	SAMN08005787	WGS-Qk63	resequencing
Cb-C	SAMN08005788	WGS-Qk64	resequencing
Cb-C	SAMN08005730	WGS-Qk6	resequencing
Cb-C	SAMN08005731	WGS-Qk7	resequencing
Cb-C	SAMN08005810	WGS-Qk86	resequencing
Cb-C	SAMN08005811	WGS-Qk87	resequencing
Cb-C	SAMN08005812	WGS-Qk88	resequencing
Cb-C	SAMN08005732	WGS-Qk8	resequencing
Cb-C	SAMN08005813	WGS-Qk89	resequencing
Cb-C	SAMN08005814	WGS-Qk90	resequencing
Cb-C	SAMN08005815	WGS-Qk91	resequencing
Cb-C	SAMN08005816	WGS-Qk92	resequencing
Cb-C	SAMN08005817	WGS-Qk93	resequencing
Cb-C	SAMN08005818	WGS-Qk94	resequencing
Cb-C	SAMN08005819	WGS-Qk95	resequencing
Cb-C	SAMN08005820	WGS-Qk96	resequencing
Cb-C	SAMN08005821	WGS-Qk97	resequencing
Cb-C	SAMN08005822	WGS-Qk98	resequencing
Cb-C	SAMN08005778	WGS-Qk54	resequencing
Cb-C	SAMN08005751	WGS-Qk27	resequencing
Cb-C	SAMN20065704	Aibaiyang	RNA-resequencing
Cb-C	SAMN20065705	Cixiyiyeqi	RNA-resequencing
Cb-C	SAMN20065706	Beimai No.4	RNA-resequencing
Cb-C	SAMN20065707	Huangqingke	RNA-resequencing
Cb-C	SAMN20065708	Zhijiangheshangtou	RNA-resequencing
Cb-C	SAMN20065709	Hamidamai	RNA-resequencing
Cb-C	SAMN20065710	Jinxingdamai	RNA-resequencing
Cb-C	SAMN20065711	Feite210	RNA-resequencing
Cb-C	SAMN20065712	Zhenongda No.2	RNA-resequencing
Cb-C	SAMN20065713	Zhenongda No.3	RNA-resequencing

Note: Wb-NE, wild barley from the Near East Fertile Crescent. Wb-T, wild barley from Tibetan Plateau. Cb-C, cultivated barley from China.Cb-NE, cultivated barley from the Near East Fertile Crescent.

Reference

Alexander D.H., Novembre J., Lange K. Fast model-based estimation of ancestry in unrelated individuals. *Genome Res.* **2009**,19,1655-1664. doi: 10.1101/gr.094052.109.

Danecek P., Auton A., Abecasis G.A., Albers C., Banks E., DePristo M., et al. The variant call format and VCFtools. *Bioinformatics*. **2011**,27,2156-2158. doi:10.1093/bioinformatics/btr330.

Malinsky M., Matschiner M., Svardal H. Dsuite - Fast D-statistics and related admixture evidence from VCF files. *Mol. Ecol. Resour.* **2020**,21,584-595. doi:10.1111/1755-0998.13265.

Purcell S., Neale B., Todd-Brown K., Thomas L., Ferreira M.A.R., Bender D., et al. PLINK: a tool set for whole-genome association and population-based linkage analyses. *Am. J. Hum. Genet.* **2007**,81,559-575. doi:10.1086/519795.

Yin C.B., Li H.H., Li S.S., Xu L.D., Zhao Z.G., and Wang J.K. Genetic dissection on rice grain shape by the two-dimensional image analysis in one *japonica* × *indica* population consisting of recombinant inbred lines. *Theor. Appl. Genet.* **2015**,128,1969-1986. doi:10.1007/s00122-015-2560-7.
